# Facility-level implementation strategies in early childhood education and care to enhance adherence to a provincial physical activity standard: Protocol for the Good Start Matters ATP+ randomised controlled trial

**DOI:** 10.1371/journal.pone.0329276

**Published:** 2025-08-12

**Authors:** Louise C. Mâsse, Chris Wright, E. Jean Buckler, Olivia De-Jongh González, Luke Wolfenden, Guy Faulkner, Valerie Carson, Viviene Temple, Mariana Brussoni, Sana Fakih, Karen Sauve, Iyoma Edache, Patti-Jean Naylor

**Affiliations:** 1 School of Population and Public Health, Faculty of Medicine, University of British Columbia, Vancouver, British Columbia, Canada; 2 BC Children’s Hospital Research Institute, Vancouver, British Columbia, Canada; 3 School of Exercise Science, Physical and Health Education, Faculty of Health, University of Victoria, Victoria, British Columbia, Canada; 4 Institute on Aging and Lifelong Health. University of Victoria, Victoria, British Columbia, Canada; 5 School of Medicine and Public Health, University of Newcastle, University Drive, Callaghan, New South Wales, Australia; 6 School of Kinesiology, University of British Columbia, University Blvd, Vancouver, British Columbia, Canada; 7 Faculty of Kinesiology, Sport, and Recreation, University of Alberta, Edmonton, Alberta, Canada; 8 Department of Pediatrics, University of British Columbia, Vancouver, British Columbia, Canada; 9 British Columbia Injury Research & Prevention Unit, Vancouver, British Columbia, Canada; 10 Child Health BC, Vancouver, British Columbia, Canada; PLOS: Public Library of Science, UNITED KINGDOM OF GREAT BRITAIN AND NORTHERN IRELAND

## Abstract

Early childhood education and care (ECEC) facilities represent an important setting to support the healthy development during early childhood through the promotion of active play (AP) and fundamental movement skills (FMS). A mandatory AP standard was enforced in 2017 for licensed ECEC centres in the province of British Columbia, Canada. In conjunction with the AP standard, a suite of capacity building resources were administered (Appetite to Play), which improved the knowledge and confidence of early childhood educators in implementing AP and FMS development activities. Child outcomes were not measured. With implementation of AP practices degrading over time, we set out to enhance existing ATP resources and developed an advanced mobile app-based professional development program title “Appetite to Play Plus (ATP+)”. ATP+ focuses on implementation strategies that integrate AP and FMS into scheduling and curriculum planning and on creating a supportive environment for AP in ECEC facilities. The purpose of the research is to investigate whether advanced mobile app-based training focused on implementation strategies and practical resources for early childhood educators and managers: 1) enhances adherence to the British Columbia Director of Licensing Standard of Practice for AP, and 2) improves 2.5- to 5.9-year-old children’s AP and FMS over a 12-week period. We hypothesise that centres randomised to the ATP+ intervention will: 1) demonstrate greater adherence to the AP standards in comparison to those assigned to the waitlist control group, and 2) children attending intervention centres will spend more time in AP while in care and have greater increases in their FMS scores. A hierarchical mixed effect model incorporating both fixed and random effects at the ECEC centre and child levels will test the impact of ATP + , while considering that children, educators and managers are clustered within ECEC centres.

**Trial registration:**

ClinicalTrials.gov NCT05669378

## Introduction

Active play (AP) in early childhood is an essential part of development [1–5]. This type of play has been positively associated with executive function, social interactions, academic achievement, cognition, language development, mental health, and physical literacy development in early childhood [6–12]. As 56% of Canadian children younger than five years are in early childhood education and care (ECEC), it is an important avenue for increasing AP in early childhood [13]. Evidence that children in this age group have insufficient motor skill proficiency and do not participate in sufficient physical activity while attending ECEC is concerning [14–16]. Early childhood is important for the development of foundational skills and habits that support physical activity participation throughout childhood and into adolescence and adulthood [11,17,18].

In 2017, the Government of British Columbia (BC) recognized the importance of both AP and the ECEC setting to children’s health and development by enacting a mandatory AP standard for licensed ECEC facilities [19–21]. The standard, officially titled ‘The Director of Licencing Standard of Practice - AP’, mandated: the amount of time spent each day in outdoor active play, written screen-time and AP policies, screen time and time spent sitting limits, implementation of facilitated and unfacilitated physical activities and the incorporation of fundamental movement skills (FMS) into routines [19–21]. As part of the rollout strategy, a capacity building intervention, Appetite to Play (https://appetitetoplay.com/), was launched to advance the knowledge and ability of early years providers to implement the AP standard [22–24]. Appetite to Play is a capacity-building approach incorporating a suite of evidence-informed implementation strategies aimed at enhancing the adoption of the AP standard and recommended practices for promoting healthy eating and AP in early years settings [22,23,25]. These are presented in detail elsewhere but include training (community-based and virtual workshops and asynchronous e-learning), resources (e.g., audit and planning tools, an activity and recipe bank, let’s make videos, etc.), community of practice, marketing and communications, and central technical support [22,23]. Scale-up was fully funded during the first 18 months after the release of the AP Standard and the training was provided in-person and online free of charge. Since February 2019, a nominal fee ($14–19) was charged for the training, and content delivery shifted to a primarily online model [19].

Research on Appetite to Play and the impact of the AP standard have demonstrated an increase in the knowledge, confidence and intention of educators to promote AP and healthy eating, and an increase in FMS practices [22,23,26]. Survey research demonstrated improved centre-level policies for AP and increased provision of daily AP and outdoor time, as well as a reduction in screen time [20]. However, following the withdrawal of the funding for Appetite to Play in 2019, survey research has indicated a return to pre-implementation of the AP Standard levels of policies and practices [19]. To date, research on the impact of Appetite to Play and the AP standard has focused on impact at the level of the early childhood educators and managers, and at the policy level of ECEC centres, but has not yet examined child-level outcomes, nor the interaction between managers and educators practices and child-level outcomes. To continue adherence to the standard and sustained use of resources, our research team developed an enhanced intervention called Appetite to Play Plus (ATP+). This intervention builds on the knowledge presented in the original Appetite to Play workshop. ATP+ enhances professional development (training) for ECEC managers and staff supports the application of facility level implementation strategies focused on integrating AP and FMS into scheduling, curriculum planning and creating a supportive environment for AP in ECEC [25]. This paper describes our plan to assess the efficacy of the ATP+ intervention in the context of a randomised control trial (RCT).

### Study aim and hypothesis

The purpose of this research is to investigate whether the enhanced training and resources to support the use/application of facility-level implementation strategies: 1) increases adherence to the BC Directors of Licensing Standard of Practice for AP, and 2) improves 2.5-5.9-year-old children’s FMS. It is hypothesized that centres randomised to the ATP+ intervention will, in comparison to those assigned to the waitlist control group, have greater adherence to the AP standard (time spent in and number of AP, FMS, and outdoor activities), and consequently children attending intervention centres will have greater improvements in their FMS scores over the intervention period. Secondary outcomes will include change in characteristics of providers (skills, self-efficacy, commitment to change), organizational processes (actions to prioritize AP and FMS), and in the environment. It is hypothesised that changes in the secondary outcomes will be higher in centres exposed to the ATP+ intervention as compared to those assigned to the waitlist control group.

## Methods and materials

### Study design

**Fig 1:**
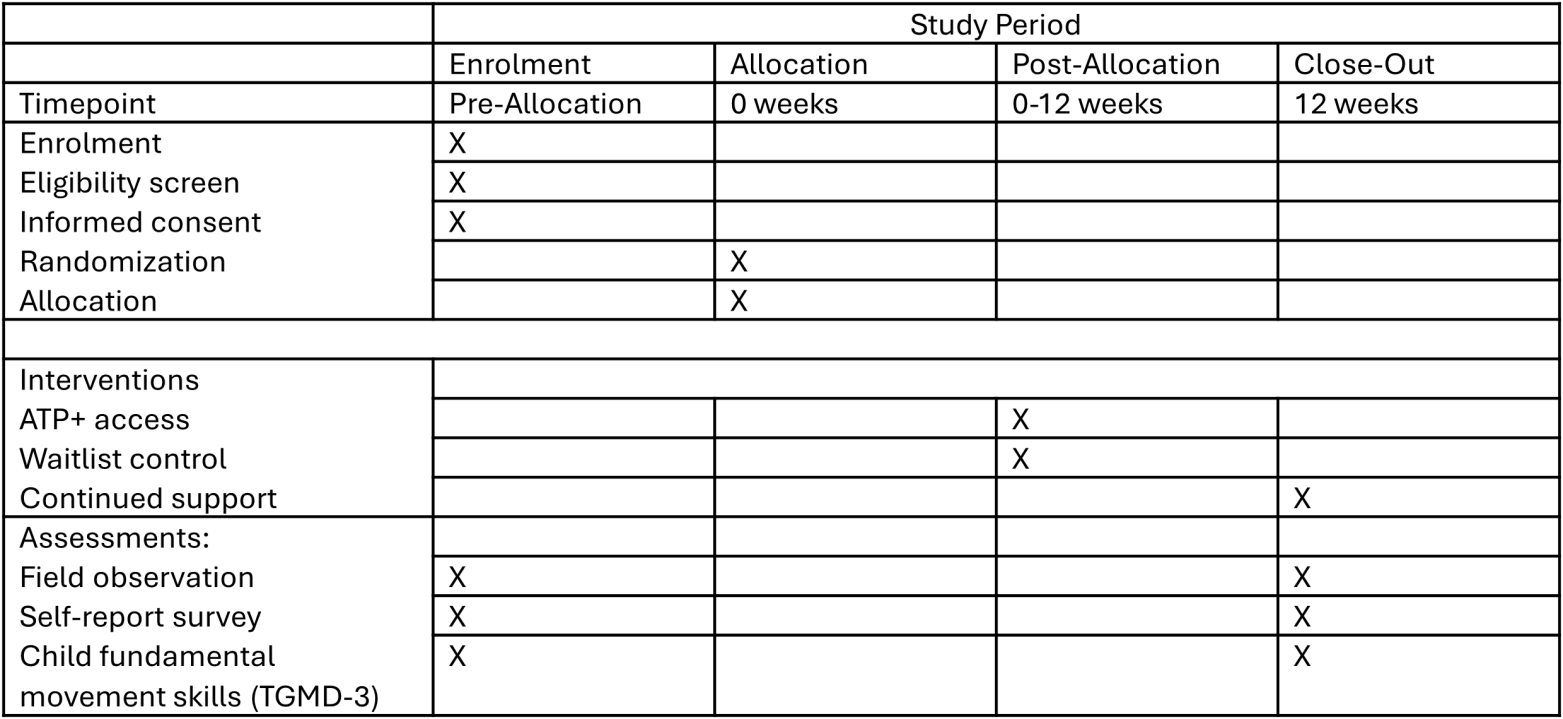
SPIRIT schedule of enrolment, interventions and assessments.

**Fig 2:**
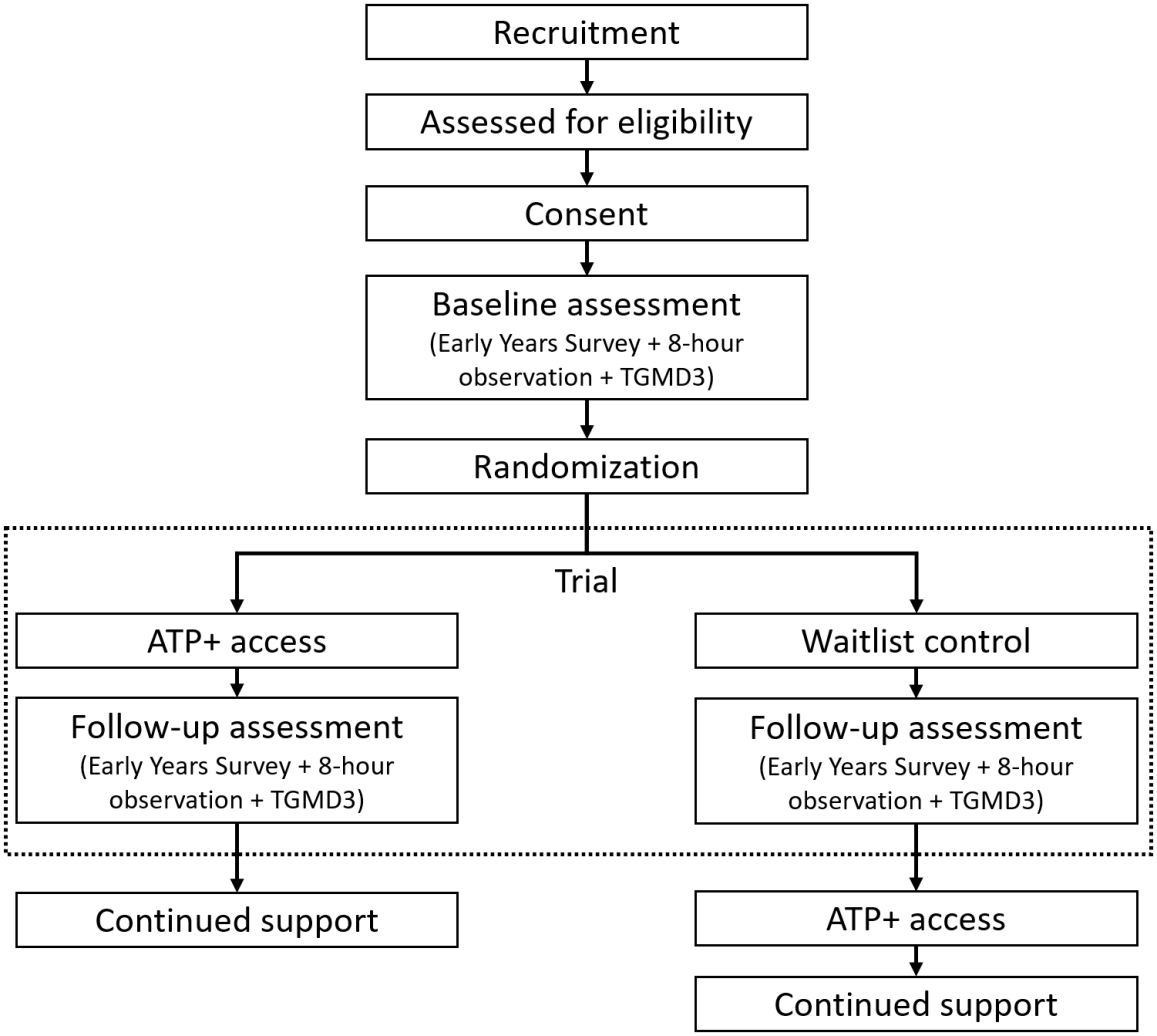
Study Flow Chart.

We will recruit 52 ECEC centres to participate in the RCT between 2023 and 2025 from the Metro Vancouver and Greater Victoria regions of BC, Canada. Recruitment began 10/10/2023, with our first data collection 08/11/2023. We anticipate recruitment will finish 15/05/2025. Data collection is anticipated to be completed 30/09/2025 and our results compiled by 01/03/2026. Centres will be recruited through an initial mail invitation and follow-up phone calls. Upon informed consent of centres meeting inclusion criteria, half will be randomised to receive the intervention immediately while the other half will receive the intervention three months post randomization and act as a standard of care waitlist control group. The experimental group will receive the ATP+ intervention, a capacity-building professional development opportunity designed to support ECEC providers in implementing the best practices for AP. The standard of care waitlist control group has access to publicly available Appetite to Play materials during the three-month waitlist period. The SPIRIT Schedule of enrolment, intervention and assessments is outlined in Fig 1, as well a visual of the study flowchart is presented in Figure 2.

The study is registered at ClinicalTrials.gov (trial ID - NCT05669378), and received ethical approval from the University of BC Behavioural Research Ethics Board (H18-01434).

### Study participants

Inclusion criteria for the ECEC centres are that they are located in the Metro Vancouver or Greater Victoria areas, provide care to at least ten children between the ages of 2.5 to 5.9 years, are licensed for group ECEC/multi-age facility by the local health authority, and provide care to children for more than four hours a day, five days per week. Family ECEC or in-home centres are ineligible to participate. As the ATP+ program requires that managers and educators work together to support AP, at least one manager and two educators who directly care for children that are 2.5 to 5.9 years of age must be willing to try out the ATP+ program and participate in its evaluation. If centres meet the inclusion criteria, the managers and educators must also meet additional inclusion criteria. Managers and educators at the centres must work full time (at least three full days per week), be 19 years or older, and hold Early Childhood Educator certifications. Managers and educators must also be fluent in English, both orally and in writing. Exclusion criteria for educators include if the individual is deemed to be completing administrative assistant duties only. Children at the centres will only be included in data collection specific to gross motor skills if their parents have voluntarily agreed for them to participate in the study and meet the aforementioned age criteria. A minimum of five parents of eligible children from one facility must consent for data collection to occur at such centre.

### Recruitment and consent

Recruitment of the ECEC centres will be conducted by research teams at the Universities of BC and Victoria from October 2023 to May 2025. Waves of letters and emails will be sent out to centres throughout the geographical regions stated in the inclusion criteria. Sending these initial letters and emails in waves, where 20–30 centres are invited simultaneously, allows for more efficient call backs to take place to understand if the centres are interested in participating and for scheduling the baseline data collection. Once centres have been deemed eligible to participate, paper and digital consent forms will be provided to participants. Specifically, managers and staff will receive printed consent forms, while parents/guardians will receive consent forms either on paper or online via REDCap hosted at BC Children's Hospital Research Institute (BCCHRI). Children whose parents have previously consented to participate in the study will also be provided the opportunity to provide verbal assent to participate in the gross motor skill activities on the day. Participation in the study is completely voluntary, and a participant may withdraw at any time without any consequences or any explanation. If a withdrawal does occur, the participant is able to choose to allow the team to retain their data collected to date or not. If the participant chooses to have their data destroyed, any analysis that migh have been conducted up to that point would remain intact due to the anonymous nature of the data.

### Data collection

All study outcomes will be collected at baseline and at follow-up, approximately three months later. This will be post-intervention for the experimental group and prior to the intervention for the waitlist control group.

### Primary outcomes

***Change in AP and FMS practices***: Child engagement in AP and FMS activities (i.e., time spent in AP, time spent in FMS, number of FMS activities, time spent outdoors, number of outdoor sessions, and an index on the number of best practices facility adheres to) will be measured with field observation and self-report. For field observations, we will use a modified version of the Environment and Policy Assessment and Observation (EPAO) tool [27], which captures type of AP and FMS (locomotor, agility, and/or object/control) activity, duration, location (indoor or outdoor), and intensity of movement. The field observations will initially be completed independently by two trained members of the research team and involve one observer once high intra-observer consistency is obtained. The observations will take place for eight hours of the ECEC centre’s operational times (8:30am-4:30 pm), to capture the activities when most children are present at the facility. The self-report evaluation will be based on questions from the Early Years Survey and the EPAO as a Self-Report instrument (EPAO-SR), adapted to specifically assess the practices targeted by the AP standards in BC, such as providing a minimum of 60 minutes outdoor AP time per day, at least 120 minutes of AP per day, and the use of both facilitated and unfacilitated AP activities that cover FMS [27].

***Change in FMS***: Child FMS will be assessed with the Test of Gross Motor Development version 3 (TGMD-3), a validated, norm-referenced tool for assessing 13 gross motor skills in both locomotor and object control categories [28]. A modified TGMD-3 protocol is commonly used in early childhood movement skill interventions [29]. In this study, we will evaluate six skills: gallop, hop, horizontal jump, two-hand catch, kick a stationary ball, and overhand throw. A composite score will be computed for locomotor and ball skills with a gross motor composite score computed for the analyses. The TGMD-3 performances will be videoed by the research team and scored at a later date. Two members of the research team will independently score the children on the TGMD-3 criteria and discrepancies will be discussed with a third reviewer.

### Secondary outcomes

***EPAO-SR tool***: This instrument will also capture: a) change in the characteristics of the providers (educators and managers) including skills, self-efficacy, and commitment to change facility practices to implement the AP standard; and b) changes in organizational processes implemented by managers including actions to prioritize AP (such as establishing a champion, modifying policies, setting goals and routines, routinizing processes, monitoring, etc.); and c) changes in the environment such as modifying the indoor or outdoor environment to support AP and FMS.

### Randomization

A computer-generated (www.sealedenvelope.com) randomization schedule will be used to allocate participants in blocks of two, four, six, or eight participants with a randomization ratio of 1:1. The allocation schedule will be concealed in the randomization module of REDCap and only assigned after informed consent and baseline assessments have been completed. Research team members and participants are blinded to allocation at baseline. They will not enter or modify the allocation schedule, as it will be entirely computer-generated. After randomization, participants and research team members will not be blinded to the allocation conditions because participants will know whether they receive the ATP+ intervention immediately or in 3 months, and team members will be tasked with providing intervention group participants access to the intervention. Allocation assignment will be concealed from the researchers at the analysis stage.

### Data confidentiality and anonymity

All data will be entered into REDCap hosted at the BCCHRI. REDCap is a secure, online software system created to facilitate data collection for research projects. It offers a user-friendly interface for validated data entry, comprehensive audit trails for monitoring data changes and exports, automated export features for easy data transfer to popular statistical programs, and methods for integrating and sharing data with external sources [30,31]. To protect participant anonymity, a unique identification number is given to each participant and names are stored in a different project. Participant confidentiality and the confidentiality of the data will be protected by having no participant or ECEC centre names on any of the data. Electronic files will be stored using a unique identification number on a secure network drive at the University of BC which will only be accessible to the investigators and research staff. Operating procedures will be documented in a procedural manual to standardize the administration of data collection methods, tracking procedures, and data entry methods into REDCap. Any adverse events will be reported to the University of BC Behavioural Research Ethics Board, and any changes to this protocol will be made to the clinical trials registry.

### Development of ATP+

ATP+ was developed by experts in the field of physical activity and AP for children in early childhood. The information in ATP+ builds on information from the Appetite to Play initiative. Appetite to Play was created as part of the Active People, Active Places strategy from the Government of BC to increase physical activity rates in a number of key focus areas from 2015–2025 [32]. Appetite to Play is a capacity-building intervention that utilizes implementation strategies, like providing accredited training and resources on physical and food literacy to early childhood educators, with the aim of enhancing children’s physical activity and nutritional habits [22,23]. It features an interactive website that offers users access to a wealth of resources, including physical activity suggestions and activities, knowledge translation articles and self-assessment and planning tools (*appetitetoplay.com*). Appetite to Play increased knowledge and confidence around AP [22] and implementation of the AP Standard increased the amount of time spent in outdoor play [20]. Barriers identified by educators were primarily less changeable characteristics of the setting such as the physical space, equipment and the attitudes and behaviours of other staff [24]. However, managers of these settings acknowledged that they had the ability to change some of these characteristics [24], which informed the development of ATP+ .

ATP+ involves training and resources in the application of facility-level implementation strategies such as integrating AP and FMS into scheduling, curriculum planning and creating a supportive environment for AP in ECEC facilities. ATP+ will target both managers and educators that are engaged with the children on a daily basis. Previous research has recommended this multi-level approach, with training to support policy and cultural change for managers and opportunities for professional development for educators [24]. Including multiple levels of staff within an organisation is an important factor in successful implementation strategies [33].

ATP+ is guided by Warrick’s (2009) change framework, aiding managers and educators in adopting and sustaining recommendedimplementation strategies related to the AP standards. ATP+ is a skill-building professional development course that provides resources and tools, including template forms, assignments with feedback, and ongoing virtual support through contact with a community of early years practitioners. Establishing ECEC-level policies to guide the use of implementation strategies for enhancing the implementation of the AP standards is a crucial initial step, although it is insufficient by itself [34,35]. Therefore, ATP+ enhances the Appetite to Play initiative capacity-building framework with additional training and resources to support application of set of facility-level implementation strategies that enhance continuous planning, monitoring and self-evaluation, while facilitating the institutionalization of AP standards for sustained implementation [36].

**Fig 3:**
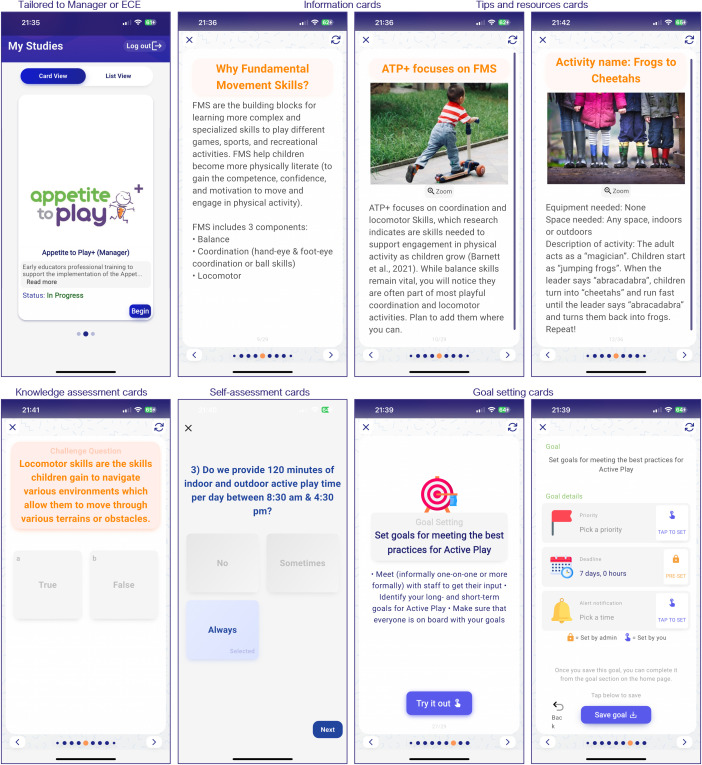
mHealth features of the ATP+ intervention in Pathverse.

Educators assigned to the intervention group will gain access to the ATP+ content through an app called Pathverse. Pathverse is a no-code app builder platform that supports mobile-health (mHealth) research [37]. The Pathverse platform consists of a web portal for researchers to create engaging mHealth interventions with “drag and drop” features instead of coding. The content is then instantly displayed on the Pathverse mobile app accessible to research participants. This app was chosen due to the user-friendly method of creating content and functionality from a research perspective. Pathverse will host information for both educators and managers, which takes approximately 30 minutes to review over three weeks (see Fig 3).

### ATP+ intervention

ATP+ includes three interactive e-learning training modules that integrate strategies for implementing the AP Standards and sustaining its implementation. Table 1 summarized the key strategies implemented and sustainability strategies that are integrated in ATP+ using Powell et al.’s [38] taxonomy. Briefly, the three e-learning modules:

**Table 1:** Description of ATP+ components with a list of implementation and sustainability strategies that are used.

ATP+ Component	Goals	Target M = Manager, C = Champion, E = Educator	Strategy (Powell et al, 2015)
**Week 1 Module: Let’s get started**			
Interactive e-learning	Refresh your knowledge about active play (AP), Fundamental Movement Skills (FMS), and physical literacy, and learn the best practices for AP and how they can be used to meet the BC Director of Licensing guidelines.	M/C/E	Distribute educational materials
Interactive e-learning	Understand your role in promoting the use of best practices for AP and ensuring their sustained implementation.	M/C/E	Revise professional roles
Interactive e-learning	Use the AP Standards Self-Assessment Tool to examine whether you and your facility are following best practices for AP.	M/C/E	Audit and provide feedback
Interactive e-learning	Prepare for change by setting goal(s) (i.e., observe children’s AP or outdoor time activities; create more AP opportunities; use the Activity Planner on the Appetite to Play website to select FMS; organize space and equipment).	E	Distribute educational materials Capture and share local knowledge
Build community of practice	Start implementing AP best practices by developing a weekly schedule that aligns with these guidelines.	M/C	Stage implementation scale up Create a learning collaborative
Interactive e-learning	Prepare for change by enlisting a champion to support implementation and establish processes to sustain it.	M/C	Identify and prepare champion
Build community of practice	Prepare for change by settings goal(s) (i.e., do an inventory of resources, policies and practices; review your current practices and seek input on what needs to be changed; ensure the weekly schedule and/or AP planning includes FMS-promoting activities).	M/C with input from E	Stage implementation scale up Capture and share knowledge Create a learning collaborative Conduct local consensus discussions
**Week 2 Module: Making changes**			
Interactive e-learning	Refresh your understanding of why coordination-focused FMS are essential, learn new coordination activities and adapt them to promote engagement and physical literacy, and discover how to create new invitations for AP by designing installations or adding equipment/loose parts.	M/C/E	Distribute AP educational materials
Build community of practice	Tailor goals for making change (i.e., try out 3 FMS coordination skills adapting them for various abilities and sharing your insights; create a new installation to invite coordination-focused AP and evaluate its effectiveness; revise your daily practices to align with these changes).	E	Change physical structure and equipment Capture and share knowledge
Interactive e-learning	Become familiar with implementation support strategies.	M/C	Distribute educational materials
Interactive e-learning	Use the self-assessment tool to evaluate whether your facility has processes in place to implement and sustain change.	M/C	Audit and provide feedback
Interactive e-learning	Learn strategies that facilitate implementation and establish processes that ensure its sustainability.	M/C	Distribute educational materials
Build community of practice	Tailor goals for making changes (i.e., adjust the weekly schedule to include coordination and locomotor activities; brainstorm ideas for excursions and outdoor time; encourage your staff to share their installation ideas).	M/C with input from E	Stage implementation scale up Conduct cyclical small tests of change Capture and share knowledge Conduct local consensus discussions
**Week 3 Module 3: Maintaining Changes**			
Interactive e-learning	Refresh your understanding of why locomotor FMS are essential, explore new locomotor activities to sharpen skills, and utilize a list of questions designed to support children’s abilities.	M/C/E	Distribute educational materials
Build community of practice	Tailor goals for maintaining change (i.e., try 3 FMS locomotor skills adapting them for various abilities and sharing your insights; create a new installation to invite locomotor-focused AP and evaluate its effectiveness; revise your daily practices to align with these changes).	E	Stage implementation scale up Change physical structure and equipment Capture and share knowledge
Interactive e-learning	Learn to develop processes that support implementation and sustain it over time.	M/C	Distribute educational materials
Build community of practice	Tailor goals for maintaining changes (i.e., use the monitoring tool and debrief with your staff to identify barriers and facilitators to implementation, as well as potential solutions to enhance compliance with AP standards).	M/C with input from E	Audit and provide feedback Purposely re-examine the implementation Stage implementation scale up Assess for readiness and identify barriers and facilitators
**Post-Module Support (weeks 1–52) and Recognition**			
Push notifications	Reminder of tasks for making and maintaining changes as well as reminder to use the self-assessment and evaluation tools. Each notification includes a FMS activity and tasks specific to role. Weekly notifications start week 1 until week 52.	M/C/E	Remind
Professional development	Professional development credits provided as an incentive for completing the ATP+ training.	M/C/E	Incentive

- Distribute educational materials to shape knowledge about: 1) why FMS are important; 2) what is AP and how can it support FMS development; 3) best practices for the implementation of AP Standards; 4) best strategies to support and sustain implementation.
- Provide audit and feedback tools to evaluate implementation of the best practices for AP and use of best strategies for sustaining the implementation of the AP standards.
- Support facilities in developing a formal implementation and sustainment plan (i.e., goal setting, timeframe & milestones, monitoring & discussion of progress) that promotes adaptability and stage the implementation of the AP Standards.
- Implement tools for monitoring the implementation of the AP standards as well as develop and organize a monitoring system for sustaining implementation.
- Identify and prepare champions (an individual within the organization who is enthusiastic and ready to support change) andestablish roles of managers, champions, and educators for the implementation and sustainment of the AP standards, ensuring everyone has a supportive and active role in both implementation and sustainment.

Two versions of the ATP+ program were created, tailored to the participant’s role at the facility (manager vs educator). While both programs have the same overall purpose and content, the strategies, tips and goals suggested are complimentary and focus on each participant’s specific role. In both versions of the ATP+ program, the content is separated into three distinct modules (see Table 1). Module 1 (“*Let’s get started*”) focuses on refreshing knowledge about AP, FMS, physical literacy, and the BC Director of Licensing AP Standards; learning best practices for AP, understanding roles of managers, champions, and educators in supporting AP; finding whether centres are using the best practices for AP; and starting to make changes with managers, champions, and educators aiming for different but complementary goals to align with their respective roles.

Module 2 (“*Making Changes*”) provides deeper learning related to teaching FMS with a particular focus on object-control/manipulation activities. It introduces new object-control/manipulation FMS activities, ways to adapt them for children with different abilities, and ideas to create invitations for AP. In Module 2, managers and champions learn the strategies for supporting implementation and how to maintain change over time. Both managers and champions assess whether they implement these support strategies and are provided with an action and communication plan for implementing the AP standards. At the end of the module, managers, champions, and educators plan to make changes by selecting goals that align with their roles.

Finally, Module 3 (“*Maintaining changes*”) focuses on expanding managers’ and educators’ learning about locomotor FMS and suggests new activities to promote these skills, along with ways to adapt these activities to suit the needs of 2.5-5.9-year-old children. In addition, managers and champions focus on specific tasks to integrate into schedules and routines to continue using the recommended implementation support strategies to support the AP standards. This module includes tools for monitoring and evaluation. The content differs for educators and managers/champions to better support each role.

The implementation of ATP+ is reinforced via push notifications. These are sent through thePathverse app and aim to: 1) develop and distribute AP materials and resources (e.g., tool box of ideas, resources for identifying new ideas, tools for planning AP activities); 2) remind all actors (managers, champions, and educators) about their roles and the actions they must initiate to implement the AP Standards and support its continued implementation; and 3) make the implementation and processes to sustain implementation dynamic and cyclical. Push notifications are initiated in week 1 and released weekly for a full year. The main purpose of these notifications is to provide ongoing support so the processes can all be restarted yearly with adaptation made possible. Implementation is also facilitated by building a community of practice within each centre to capture and share knowledge about implementation of the AP Standards and sustainment of the AP Standards over time.

In addition, all resources including the training materials, videos and links to external websites remain available in the Pathverse app for the duration of the study and beyond. Upon completion of the program, participants receive three professional development credit hours. Professional development hours are required for Early Childhood Educators to maintain their certification.

### Reporting

We will report study findings in accordance with CONSORT and STARI guidelines. We will submit final study results to a peer-reviewed scientific journal for publication and present findings at academic conferences. We will also report study results in the ClinicalTrials.gov registry.

### Sample size calculation

A sample of 79 educators per group, within 26 ECEC centres would be sufficient to detect a 15% improvement relative to control in AP minutes, given an ICC of 0.03, a design effect of 1.06 and an alpha of 0.05 with 80% power [39].

For child outcomes, a similar calculation was performed to estimate the number of children needed to detect a 0.5 standard deviation increase in FMS outcomes with 80% power and an alpha of 0.05. Assuming a 25% attrition rate and that 5 children participate per site at both time points, 92 children per group are needed, which translates to 36 centres. However, since the requirements for educator outcomes are more stringent, 52 centres will be recruited and randomized, and child-level data will be collected at all 52 centres.

### Data analysis

A hierarchical mixed effect model incorporating both fixed and random effects at the ECEC and child levels will test the impact of ATP+ on the primary and secondary outcomes, while accounting for the clustering of children, educators, and managers within ECEC centres). Following intention-to-treat principles, several analyses will be conducted with each of the primary and secondary outcomes. The corresponding levels of policy and practice variables will be used as the main independent variables, with child-level demographic variables (age, sex) included as covariates. Missing data patterns will be examined to determine proper imputation techniques to use before or during data analysis (e.g., multivariate normal imputation, chained equations, etc.).

## Discussion

The importance of AP in early childhood is well-established, with numerous studies highlighting its positive effects on physical, cognitive, social, and emotional development [6–12]. While policies have been put in place to increase the amount of AP time at early years centres, continual adherence to the standard and sustained use of available resources is necessary. This protocol will assess the potential of online professional development centred around implementation support strategies for changing and sustaining practices related to AP and consequently children’s AP while in care.

The use of an online professional development model has the potential to remove many barriers associated with traditional learning opportunities such as time, location and accessibility [40–42], as well as the flexibility of self-paced learning opportunities which suits educators [43]. However, the variability in educators’ competence and comfort with digital technologies needs to be taken into account [43], along with different learning styles that may not suit online learning platforms [42]. In addition, the potential attrition rate and lack of engagement by educators through the online platform due to a perceived lack of oversight is a limitation of the study [42].

The ATP+ intervention represents a significant advancement in promoting AP standards within early childhood settings through mobile app based professional development. By utilizing the Pathverse app, this intervention provides flexible, accessible, and self-paced professional development opportunities for educators and managers, effectively addressing common barriers associated with traditional training methods. The structured modules and focus on implementation strategies are designed to support centres and facilitate implementation and sustainment of change in AP practices, thereby benefiting children’s physical, cognitive, and social development. This is done through reflective practices and practical solutions to sustain change. However, potential challenges such as varying levels of digital competence among educators, differing learning preferences, and engagement issues must be considered. Addressing these limitations and continuously refining the intervention based on participant feedback will be essential for maximising its effectiveness and sustainability.

Furthermore, the feasibility of conducting the RCT within the specified timeframe and with the available resources appears efficacious, given the clear inclusion criteria, structured recruitment process and detailed data collection methods. Future research should focus on long-term outcomes and scalability to ensure the benefits of the ATP+ intervention can be realised across diverse early childhood settings.

## Supporting information

S1 File ATP+ Protocol. (PDF)

S2 File Childcare full REB submission H18-01434. (PDF)

S3 File Fillable-SPIRIT-Outcomes-2022-Checklist-with-SPIRIT-2013. (PDF)
