# Supplementary material for: Facility-level implementation strategies in early childhood education and care to enhance adherence to a provincial physical activity standard: Protocol for the Good Start Matters ATP+ randomised controlled trial
Source: PLoS One. 2025 Aug 12;20(8):e0329276. doi: 10.1371/journal.pone.0329276 (PMC12342287; doi:10.1371/journal.pone.0329276)
Supplement: S1 File — (PDF) [file pone.0329276.s001.pdf]

Date: 07/04/2025, 12:32:40 | Print | Close

undefinedundefined

The University of British Columbia
Office of Research Ethics
Behavioural Research Ethics Board
Suite 102, 6190 Agronomy Road
Vancouver, BC V6T 1Z3

H18-01434 **Child Care (Version 30.0)**

Principal Investigator: Louise C. Masse

1. Principal Investigator & Study Team - Human Ethics [View Form]

1.1. Principal Investigator	**Last Name**	**First Name**	**Employer.Name**	**Email**
	Masse	Louise C.	School of Population and Public Health	lmasse@bcchr.ubc.ca

Enter Principal Investigator's secondary appointments or affiliations (including Health Authorities), if applicable:	School of Population and Public Health

1.2. Primary Contact	**Last Name**	**First Name**	**Rank**
	de Jongh Gonzalez	Olivia	Post Doctoral Fellow

1.3A. Co-Investigators - Online Access

Last Name	First Name	Institution/Department	Rank	TCPS2
Weatherson	Katie	UBC/UBCO - Faculty of Health and Social Development/UBCO - School of Health and Exercise Science	M&P Staff	yes
Brussoni	Mariana	UBC/Medicine, Faculty of/Paediatrics	Professor	yes
Temple	Viviene A	University of Victoria	Non-UBC Faculty Member	
Carson	Valerie	Other/Other University/Hospital	Non-UBC Faculty Member	
McKay	Heather A.	UBC/Medicine, Faculty of/Family Practice	Professor	yes
Buckler	Jean	University of Victoria	Assistant Professor	yes
Wolfenden	Luke	Other/Other University/Hospital	Non-UBC Faculty Member	
Faulkner	Guy	UBC/Education/School of Kinesiology	Professor	yes
Naylor	Patti-Jean	University of Victoria	Professor	yes
McConnell-Nzunga	Jennifer	UBC/Medicine, Faculty of/School of Population and Public Health	Post Doctoral Fellow	yes
Lau	Erica Y	Other	Unspecified	yes

1.3B. Describe each Co-I's role in study, e.g. statistician, supervisor, adviser, student etc.

<table>
<tr><td>Ensure individual is entered in Box 1.3A</td><td></td></tr>
<tr><td>1.4A. Additional Study Team Members - Online Access</td><td>
<table>
<tr><th>Last Name</th><th>First Name</th><th>Institution/Department</th><th>Rank</th><th>TCPS2</th></tr>
<tr><td>Leon Elizalde</td><td>Angelica</td><td>Other</td><td>Unspecified</td><td>yes</td></tr>
<tr><td>Smith</td><td>Kate</td><td>UBC/Science</td><td>Undergraduate Student</td><td>yes</td></tr>
<tr><td>Scarr</td><td>Jennifer</td><td>Other/Other University/Hospital</td><td>Non-UBC Employee</td><td></td></tr>
<tr><td>Wright</td><td>Chris</td><td>University of Victoria</td><td>Non-UBC Faculty Member</td><td>yes</td></tr>
<tr><td>Marshall-beaucoup</td><td>Sophie</td><td>UBC/Medicine, Faculty of/School of Population and Public Health</td><td>Graduate Student</td><td>yes</td></tr>
<tr><td>Sauve</td><td>Karen</td><td>UBC/Medicine, Faculty of/School of Population and Public Health</td><td>Graduate Student</td><td>yes</td></tr>
<tr><td>de Jongh Gonzalez</td><td>Olivia</td><td>UBC/Medicine, Faculty of/School of Population and Public Health</td><td>Post Doctoral Fellow</td><td>yes</td></tr>
<tr><td>Pitblado</td><td>Mark kenneth, john</td><td>UBC/Science</td><td>M&P Staff</td><td>yes</td></tr>
<tr><td>Edache</td><td>Iyoma</td><td>UBC/Medicine, Faculty of</td><td>Graduate Student</td><td>yes</td></tr>
<tr><td>Hassani</td><td>Kasra</td><td>UBC/Medicine, Faculty of/BC Children’s Hospital Research Institute</td><td>Non-UBC Employee</td><td>yes</td></tr>
<tr><td>Deslippe</td><td>Alysha</td><td>UBC/Land and Food Systems</td><td>Sessional Instructor/Lecturer</td><td>yes</td></tr>
<tr><td>Tugault-Lafleur</td><td>Claire</td><td>Other</td><td>Unspecified</td><td></td></tr>
<tr><td>Li</td><td>Joy</td><td>UBC/Land and Food Systems</td><td>Undergraduate Student</td><td>yes</td></tr>
<tr><td>Dong</td><td>Amy</td><td>UBC/Medicine, Faculty of/School of Population and Public Health</td><td>Student Worker</td><td>yes</td></tr>
<tr><td>Villagomez Volkers</td><td>Paula</td><td>UBC/Medicine, Faculty of/School of Population and Public Health</td><td>Graduate Student</td><td>yes</td></tr>
</table>
</td></tr>
<tr><td>1.4B. Describe each Additional Study Team Members' role in study, e.g. staff, research assistant etc.</td><td></td></tr>
<tr><td>1.5A. Additional Study Team Members - No Online Access</td><td>
<table>
<tr><th>Last Name</th><th>First Name</th><th>Institution / Department</th><th>Rank / Job Title</th><th>Email Address</th></tr>
<tr><td>Nieuwenhuijs</td><td>Eva</td><td>UBC</td><td>research staff</td><td>evaspph@student.ubc.ca</td></tr>
<tr><td>Alexander</td><td>Megan</td><td>University of Victoria</td><td>research staff</td><td></td></tr>
<tr><td>Graydon</td><td>Emma</td><td>University of Victoria</td><td>research staff</td><td>emmagraydon@uvic.ca</td></tr>
<tr><td>Hall</td><td>Courtney</td><td>University of Victoria</td><td>research staff</td><td>chall625@uvic.ca</td></tr>
<tr><td>Shek</td><td>Natalie</td><td>UBC</td><td>research staff</td><td>natalieshekjh@gmail.com</td></tr>
<tr><td>Armeanu</td><td>Nicole</td><td>UBC</td><td>research staff</td><td>narmeanu@student.ubc.ca</td></tr>
<tr><td>Grant</td><td>Grace</td><td>University of Victoria</td><td>research staff</td><td></td></tr>
</table>
</td></tr>
<tr><td>1.5B. Describe each Additional Study Team Members' (no online access) role in study, e.g. external supervisor, consultant etc.</td><td>(PAA April 17, 2024) Four new research staff will start working on this project, and all of them will be involved in recruitment and data collection for centers, educators and parents:
1) Nicole Armeanu, TCPS2 certificate obtained on 7-10-2022
2) Natalie Shek, TCPS2 certificate obtained on 8-10-2022
3) Courtney Hall, TCPS2 certificate obtained on 29-9-2023
4) Emma Graydon, TCPS2 certificate obtained on 2690-2023</td></tr>
</table>

	April 30, 2024 Staff who no longer worked on the project were deleted from the application as requested by REB. Names are included here as these staff/students were on publications for this study which required that they be listed on the REB certificate (Karen Strange, Camilla Briggs, Kristin McIlhenney, Kayla Morton, Sana Fakih)<br><br>(PAA May 17, 2024)<br>Two new students have joined our team and both of them will be supporting recruitment and data collection for this project:<br>• Grace Grant, University of Victoria, Completed TCPS2 on 17 May, 2024<br>• Megan Alexander, University of Victoria, Completed TCPS2 on 25 April, 2024<br><br>PAA July 31, 2024: A new student, Eva Nieuwenhuijs, is joining our team and will support recruitment and data collection for this project. Eva completed the TCPS2 on July 17, 2024.
1.6. Tri Council Policy Statement (TCPS) Tutorial<br><br>*Have all research personnel completed the required TCPS2 tutorial:*	Yes
1.7. Project Title<br><br>*Enter the title of this research study as it will appear on the certificate. Title given **must match** the title on all study documents.*	Good Start Matters
1.8. Project Nickname<br><br>*Enter a nickname for this study. What would you like this study to be known as to the Principal Investigator and study team?*	Child Care
**2. Study Dates and Funding - Human Ethics** [View Form]	
You plan to start collecting data immediately after obtaining ethics and any other required approvals	no
You plan to start data collection at a later date i.e., 2 months or more after approvals are obtained. Click the calendar icon below to select the dates.<br><br>*Estimated start date:*	9/11/2019
2.1.B.<br><br>*Estimated end date:*	3/31/2025
2.2.A. Types of Funds<br><br>*Please select the applicable box(es) below*	Grant

to indicate the type(s) of funding you are receiving to conduct this research. ***You must then complete section 2.3 and/or section 2.4 for the name of the source of the funds to be listed on the certificate of approval.***	
2.2.B. *For Industry Sponsored studies, please provide a sponsor contact.*	
2.3.A. *Research Funding Application/Award Associated with the Study that was Submitted to the UBC Office of Research Ethics*	**UBC Number** / **Title** / **Sponsor**<br>F18-04316 / Evaluating the impact of a province-wide capacity building intervention on nutrition policies and practices in early years providers / Canadian Institutes of Health Research (CIHR)<br>F16-04356 / A good start matters: Do supportive childcare centre environments, policies and practices enhance physical activity? / Canadian Institutes of Health Research (CIHR)<br>F19-03402 / A Good Start Matters: Do provincial standards and capacity-building efforts influence childcare centre environments, policies and practices related to physical literacy in the early years? / Social Sciences and Humanities Research Council of Canada (SSHRC)<br>F22-01244 / Unraveling the complexity of familial influences that shape children's obesity-related behaviours over time / Michael Smith Health Research BC
2.3.B. *Which institution is administering the funds, if not UBC or UBC affiliated institution?*	
2.4.A. *Research Funding Application/Award Associated with the Study not listed in question 2.3.*	**UBC Number** **Title** **Sponsor**
2.4.B. *Please enter any applicable information about your funding which is not already shown in Box 2.3A or 2.4A (including funding applied for but not yet received).*	SSHRC funding was also received but the funds are allocated to Dr. Patti Jean Naylor from the University of Victoria.
2.5.A. *Is this a DHHS grant?*	no
2.5.B. *Please select the appropriate DHHS funding agency from the selection box.*	**DHHS Sponsor List:** **Order:** **Active:**
2.6. Study Related Conflict of Interest<br><br>*Conflicts of Interest (COIs) in research are situations where someone's personal*	no

interests (financial, career, or other) could compromise or could be perceived to compromise the objective conduct of research or integrity of the data. Conflicts of interest can arise naturally from an Investigator's engagement inside and outside the University, and the mere existence of a COI or the perception of a COI does not necessarily imply wrongdoing on anyone's part. Nonetheless, real and perceived COI must be recognized, disclosed, and assessed. This question asks Investigators to disclose COIs that may relate to the research study that is the subject of the REB application.<br><br>*Do the Principal Investigator, Co-Investigators and/or their related parties have any personal interest(s) that could compromise or reasonably be perceived to compromise the objective conduct of the research or the integrity of the data generated by the study? Personal interests may include business, commercial or financial interests, dual roles (e.g. PI and Doctor), as well as personal matters and career interests.*	
**4.A. Study Type - (Boxes 4.1 to 4.2C)** [View Form]	
4.1. Application Type<br><br>*Indicate whether your application is Clinical or Behavioural.*	Behavioural
4.2.A. *Institutions and Sites for Study (including study team members' institutional affiliations under which this research is being conducted)*	**Institution** / **Site**<br>UBC / Vancouver (excludes UBC Hospital)
4.2.B. *Non-UBC Institutions and Sites for*	**Institution** / **Site**

Study (including study team members' institutional affiliations under which this research is being conducted)	University of Victoria Main Campus
4.2.C. *Please enter any other locations where the research will be conducted under this Research Ethics Approval (e.g., Name of privately owned clinic, community centre, school, classroom, participant's home, in the field - provide details).*	The study will take place in childcare facilities throughout British Columbia. Since this is a behavioural study, REB approval was sought from UBC BREB.

4.B. Behavioural Study Type - (Boxes 4.2D to 4.6) [View Form]

	**Study Site:**	**Accessing Records or Charts:**	**Analysing Data or Utilizing Lab Space:**	**Recruiting Participants:**	**Team Member Affiliations:**
4.2.D. Roles of Study Sites and Institutions	UBC - Vancouver (excludes UBC Hospital)	no	yes	yes	yes
	University of Victoria - Main Campus	no	no	yes	yes

4.3.A. *If this proposal is closely linked to any other proposal previously/simultaneously submitted, enter the Institution or Health Authority name and associated Research Ethics Board study number of that proposal.*<br><br>*Institution Name:*	University of Victoria
REB study number:	BC16-128
4.3.B. *If applicable, please describe the relationship between this proposal and the previously/simultaneously submitted proposal listed above.*	The harmonized ethics application (BC16-128) was previously approved by University of Victoria REB. Currently, the PI from UBC (L. Masse) is taking the lead on the study for the data collection of groups 3, 4, and 6, thus, REB approval is sought from BREB. Given that the bulk of the funds is held at UBC and that the original application was too complex, I am requesting that the board of record for the component of this study be transferred to UBC.
4.3.C. *Have you received any information or are you aware of any rejection of this study by any Research Ethics Board? If yes, please provide known details and attach any available relevant documentation in Box 9.7.*	no

Please provide known details:	
4.4.A. *External peer review details:*	SSHRC<br>CIHR
4.4.B. *Internal (Institution or hospital) peer review details:*	N/A
4.4.C. *If this research proposal has not received any independent scientific/methodological peer review, explain why no review has taken place.*	N/A
Participant Vulnerability	Medium
Research Risk	Low
4.5.B. *Provide explanations for the assessments of research risk and participant vulnerability reported above.*	Young children (3-5 yrs) so this study was classifying as medium to the age of this children.
4.5.C. *Does your application fall under minimal risk (i.e., was it assigned an overall risk level of 1 or a blue box on the minimal risk matrix above)?*	yes
**4.C. Behavioural Study Type - (Boxes 4.7 to 4.8)** [View Form]	
4.7.A Creation of a Research Database or Registry<br><br>*Does this study involve the creation of a research database or registry with a local custodian for future unspecified research?*	no
4.7.B. *Is the purpose of this application exclusively to obtain approval for the creation of a research database or registry? [Note: if the creation of the database or registry is part of a bigger project also included in this application, you must answer "no" below].*	no
Survey Research<br><br>*Is this a **minimal risk***	no

study exclusively using a survey for data collection?	
Secondary Use<br><br>*Is this a **minimal risk** study exclusively analyzing previously collected data?*	no
**5. Summary of Study and Recruitment - Behavioural Study** [View Form]	
5.1.A. *Provide a brief statement about the project written in lay language. Do not exceed 100 words and do not cut and paste directly from the study proposal.*	We aim to 1) examine whether implementation of provincial guidelines and capacity-building interventions for physical activity (PA) and healthy eating (HE) impacts the environment, policies and practices in childcare settings and how implementation influences children's PA and HE. 2) To monitor the implementation processes, and examine influencing factors. This research will help to establish an understanding of current PA and HE environments, policies and staff practices in childcare settings to inform future policy and may also have implications for prevention of diseases associated with physical inactivity and unhealthy eating.
5.1.B. *Summarize the research proposal, including study purpose, hypothesis, study population, and research method.*	As a result of stakeholder consultation for the BC Provincial physical activity (PA) and healthy eating (HE) strategies, provincial practice guidelines targeting PA and HE in early years settings supported with capacity-building interventions (training, resources. support) were released/implemented in late 2016. These guidelines are voluntary and as such, childcare facilities and Early Years programs are not mandated to follow them. We are uniquely positioned to build upon this investment and evaluate the impact of the guidelines and capacity-building efforts (evaluating the level of uptake and implementation by childcare centres, family resources and Early Years Programs and their impact on policies and practices, and ultimately children's health).<br>Appetite to Play is the capacity building portion (training workshops and website) of the Early Years initiative. In February 2017, the contract for Appetite to Play was finalized.<br>Research objective(s) and question(s)<br>The primary research objective is to examine whether implementation of provincial childcare guidelines and capacity-building interventions for PA and HE impacts the environment, policies and practices in early years settings and how implementation influences children's PA and HE. The secondary objectives are to a) monitor the reach, dose and acceptance of training and resources, and b) examine the factors that influence implementation of the guidelines and predict higher implementation dose. The research questions are:<br><br>1) Assess whether the Appetite to Play Standards and supporting scale up intervention (training and resources) significantly change HE and PA policies of licensed childcare facilities and practices of providers over time. (AIM 1of CIHR with the addition of HE)<br><br>2) Identify (qualitatively and quantitatively) the hierarchy of factors that influence implementation of the Appetite to Play Standards and uptake of the multi-component scale up intervention. (AIM 2 of CIHR with the addition of HE)<br><br>3) To develop and pilot the content of the ATP+ program which complements the original ATP program which was offered in-person. This PAA request is targeting this aim.<br><br>4) To evaluate the efficacy of the ATP+ program on change in practices (i.e., more active play) and child outcomes (i.e., more physical activity and better fundamental movement skills).<br><br>The importance and contributions of the research This research will help to establish a baseline understanding of current PA and HE environments, policies and staff practices in early years settings prior to the release of provincial guidelines and capacity-building initiative (training, resources, support) for the early years. It will contribute to the understanding of the impact of policy and capacity-building and will help to inform future policy development and implementation in Canada in regards to the early years settings. It may also have direct implications for the prevention of diseases associated with physical inactivity, unhealthy eating and overweight/obesity.

	SUB-STUDY AIMs<br>With post-doctoral funding from the Michael Smith Foundation for Health Research, Dr. Olivia de Jongh Gonzalez will integrate a sub-study within the Good Start Matters! study. The original Appetite to Play initiative which we are supplementing with our ATP+ program is a provincial initiative that aims to engage children in active play and ensure that they adopt healthy eating habits. Dr. de Jongh Gonzalez program of research aims complement the suite of resources developed by Appetite to Play and add a critically needed focus on parenting – an aspect that Child Health BC and the Ministry of Health intends to expand upon. To address this needs, the sub-study has the following overall objective to conduct a mixed-methods randomized controlled trial to improve co-parenting practices and child obesity-related outcomes. The specific aims of this sub-study are to:<br><br>Sub-study AIM 1: To examine how usual parenting practices and variations between parents shape children's eating and PA behaviors.<br>Sub-study Aim 2: To determine how intra- and inter-parent variations in parenting practices influence: a) fluctuations in child behaviors over time as well as b) overall child health behaviors.<br>Sub-study Aim 3: To test the efficacy of a 3-months co-parenting practices intervention in: a) improving parenting practices (primary proximal outcome), b) reducing intra- and inter-parent variations in parenting practices (exploratory outcome), and c) improving child health behaviors related to active play and healthy dietary habits (primary distal outcome).
5.2. Inclusion Criteria<br><br>*Describe the participants being selected for this study, and list the criteria for their inclusion.*	Managers, staff and children in group childcare facilities (In harmonized UVic application managers, staff, and children were referred as groups 3, 4, and 6 respectively). This study targets group childcare facilities that provide care for children from infancy to school age (averaging 32 children per facilities) and account for 87% of the children in licensed care in BC. In June 2015, there were 3,044 licensed group childcare providers that provided care to 96,478 children across four service delivery areas.<br><br>Eligibility (AIMS 1 & 2): BC licenced childcare providers caring for children aged 30 months to 5 years are eligible. About 68% of the 3,044 licensed group childcare providers meet this criterion; 2,070 eligible facilities. Participants must work full time (defined as working at least 3 full days per week) as a Manager or Staff. Manager defined as Executive Director, or supervisor, or program manager.<br>Staff defined as Early Childhood Educator, instructor, or staff that cares for 30 months to 5 year old children.<br><br>Eligibility (AIM 3 & 4): Our primary sampling frame will be the childcare facilities that completed the 2022/23 EY Surveys. We will pilot our ATP+ program with three childcare centres within Vancouver in the summer 2022.<br>Given the intense nature of this Aim 4, and the cost of collecting onsite childcare-level and child-level data we will recruit 52 facilities from the the greater metropolitan area of Vancouver. In addition, facilities that provide care to at least 20 children (30 months to 5 year) will be targeted.<br>Children (ages 3 to 5 years old), who can provide objective data on PA and nutrition behaviours in the childcare setting through accelerometry, direct observation and fundamental movement skills (FMS) testing.<br><br>Eligibility (AIM 4 - substudy)<br>We aim to recruit 118 families (236 parents/legal guardian/primary caregivers) whose facilities are invited to participate in the ATP+ RCT. Eligible parents must: 1) Be a parent/legal guardian/caregiver with the primary custody of a 2.5-5-year-old child who attends a BC licensed childcare facility, and the child must be able to follow general nutrition and physical activity guidelines; 2) Be fluent in English both orally and in writing; 3) Enroll with a partner/co-parent/caregiver willing to participate in the parenting program with them (single parents/primary caregivers can still enroll in this program alone, or can choose to enlist 1 person who shares caregiving responsibilities with them (e.g., grand-parent, step-parent); and 4) Have a smartphone where each parent can individually receive notifications and be able to download and use the app on this smartphone. Specifically to participate in the parenting interviews, both parents/legal guardians/caregivers must be willing to be interviewed and must have downloaded and used the app.

	(February, 2024) Centers and participants located in Victoria that meet the rest of the above mentioned eligibility criterial will also be eligible to participate in the study.<br><br>(May 17, 2024) Specifically to participate in the parenting interviews sub-study, control group participants are also eligible to participate in the interviews if they meet the rest of the criteria previously described for the interview substudy except the requirement of having used the app as they are from the control group.<br><br>(March 4, 2025) ATP interviews: A total of 20 childcare educators and 10 managers who had access to the app as part of the randomized controlled trial (specifically those assigned to the intervention group) will be invited to participate in the interviews. A quota sampling approach will be used to ensure representation of both app users and non-users, allowing for an examination of perceived utility as well as barriers to use.
5.3. Exclusion Criteria<br><br>*Include details if otherwise eligible participants will be excluded due to other characteristics. If no exclusion criteria are applicable, enter n/a.*	This study excludes children in licensed family childcare.<br><br>(Aim 4 sub-study) If the family is currently participating in a pediatric weight management program or other nutrition program, or the child has any severe dietary or physical restriction that limit their ability to follow general nutritional or movement guidelines for 2-5-year-olds that will be delivered via the app.
5.4. Recruitment<br><br>*Provide a detailed description of the steps you will use to recruit participants. Include:*<br>*a) How will prospective participants be identified?*<br>*b) By what means will recruitment be done (e.g., public posting, direct contact, third party recruitment, etc.)?*<br>*c) Who will contact prospective participants?*<br>*d) If recruitment will occur in person, what sites will be used (e.g. doctor's office, hospital clinic, etc.)?*<br>*e) Attach all materials, including letters of initial contact, posters, scripts and advertisements, to Box 9.4.*	Data collection (AIMS 1 and 2): Licensed Child Care facilities will be identified via publically-available lists (e.g., BC ChildCare Map http://maps.gov.bc.ca/ess/hm/ccf and healthspace.ca /). In both the 2018/19 and 2019/20 survey waves, eligible childcare facilities will be sent via mail and email a preliminary notice (postcard) to alert them that our team will soon be inviting the managers and their staff to complete the EY survey and will included: a) a short description of the study; b) a link to our study website where they can sign-up to receive the survey online; and c) a date indicating when they can expect to receive the EY survey. These notices will be followed by an invitational package (mailed/emailed) detailing data collection procedures which includes: informed consent and a link to the online EY surveys (administrator/staff survey). Administrators or assistants will forward invitational emails to their staff. Those who agree to participate will receive an email link to the staff EY surveys. Research staff will be emailing and calling administrators and their assistants to follow-up and ensure an acceptable response rate. Administrators and staff surveys take about 20 minutes to complete on-line. Staff surveys include self-report of typical and “previous day” practices.<br><br>Note: In our original UVic harmonized application, used to collect the 2016/17 data, we originally planned to follow-up only those who signed up for the “longitudinal part of the study”. Given that there is such a high turnover of staff in these settings and that staff did not provide their contact information unless they opted to receive a nominal incentive we have no way of eliminating those who opted out as they completed the survey anonymously. As a results, we no longer ask staff to sign up for the longitudinal study but instead have identified this as a limitation of large scale evaluation (e.g., that those who completed the survey at each time point may not be the same people even though the sites are the same).<br><br>Two re-contact recruitment procedures were used to facilitate recruitment – these recontact procedures are no longer ongoing as these re-contacts have already happened, but are a part of the original UVic application:<br>1. Multi-age facilities meet our inclusion criterion but our skip patterns excluded them. As a result, the survey was amended, we resent the survey to multi-age facilities. See Multi-Age Facilites TTT<br>2. Managers were recontacted to respond to questions that were originally only asked to staff. Staff were recontacted to answer an additional 16 questions to align the “daily practices” questions with the beaseline data collection.<br><br>Data collection (AIM 3):

We will contact managers of childcare centres in the the greater metropolitan area of Vancouver using the public lists used for Aims 1 and 2 and that are conveniently close to BCCHR. We will send the manager an invitation email (with ECE consent form in attachment). If interested, managers will need to connect with their staff to see whether they are interested in participating and that they would need to share they names and emails with the research team. To enroll the manager and staff into the pilot, everyone (manager + 3 ECE) needs to sign the consent form and complete the demographic forms (same as previously approved for EY survey).

Data collection (AIM 4):
(old content approved)
We will contact managers of childcare centres in the greater metropolitan area of Vancouver using the public lists used for Aims 1 and 2. Facilities will receive an email or letter invitation describing the purpose of the study and inviting facility to try out the ATP+ program and to participate in the evaluation of the ATP+ program. Those who expressed an interest in the study will be invited to have a zoom meeting with our team so that we can provide the facility with more information about the study or alternatively they can access the presentation about the study on their own. Those who are interested to proceed with the study will be mailed or emailed an invitation package that includes an invitation letter and a copy of the consent. After they have had the opportunity to review everything they can move ahead with registering for the intervention. As for the recruitment of the parents, childcare providers will facilitate delivery of invitation packages to parents which may include having the staff giving the invitations to the parents directly, emailing the invitation, or allocating a time when one of our staff can deliver the invitation packages to parents and answer any questions they may have about enrolling their child into the ATP+ program evaluation.

(modifications on Nov 22, 2023) We will contact managers of childcare centres in the greater metropolitan area of Vancouver using the public lists used for Aims 1 and 2. Facilities will receive an email and letter invitation describing the purpose of the study and inviting facility to participate in the evaluation of the ATP+ program. The research staff will call the manager to assess initial interests in the study and schedule an in-person meeting after the manager had the time to share broadly the invitation and assess facility's willingness to proceed to an initial meeting. Those who expressed an interest in the study will be invited to have an in-person meeting with our team so that we can provide the facility with more information about the study. Research staff will meet with staff at the facility, do a brief introduction to the study, answer any of their questions and provide them an invitational package with a brochure and consent form. 1 to 2 weeks after, our staff will return to the facility to answer questions and collect consent forms (see section 6.6 to review consent process). As for the recruitment of the parents, childcare providers will facilitate delivery of invitation packages to parents which may include having the staff giving the invitations to the parents directly, emailing the invitation, or allocating a time when one of our staff can deliver the invitation packages to parents and answer any questions they may have about enrolling their child into the ATP+ program evaluation or the Parenting study.

Data collection for the sub-study (AIM 4):
As part of the ATP+ RCT parents of facilities were invited to receive the ATP+ training are asked to indicate whether they will consent to have their child be involved into the evaluation of the ATP+ program. A sub-set of these parents will be asked to participate into a second study – the parenting intervention where parents get to receive parenting tips to support active play or healthy dietary habits. When parents enroll for the evaluation of the parenting program, they have the option to select whether they want to complete only the quantitative assessment, or the qualitative interviews as well but these interviews will only enroll the first 15 families that select this component of the study). Parents will have the option to opt out of either study or to select which aspect the family wish to get involved. Parents learn about the ATP+ program evaluation and the parenting program by receiving an invitation package from the research which will be distributed either by the staff at the childcare facility, a research staff stationed at the childcare facility who would handout the packages to parents at the door and can answer questions about the study, via email sent by the childcare facility, or via our direct email and/or phone call if the parent has already registered for the ATP+ study and agreed to be contacted for future studies. In addition, a

pamphlet / advertisement with information about the parenting study will be distributed to centers for them to post it in the communications board, and the pamphlet will have a QR code which parents can scan to read the consent and enroll in the study if interested.
The invitational letter or email would include a copy of the consent form, and parents would be asked to review the information and to discuss the content of the package as a family given that enrollment into the parenting intervention as well as in the parenting interviews is designed to have both parents included in the study. If the family elects to register for the study, they can complete the registration either online or on paper. One parent (hereafter “the main parent”) would register themselves, consent and provide information about the other parent/caregiver (hereafter the co-parent) who has agreed to be contacted for this study. Registration by the main parent would trigger an email to the co-parent to determine whether they wish to enroll into the evaluation of the parenting intervention. It will be made clear that registering a partner for the intervention does not mean that the coparent has to agree our invitation to enroll as participation is completely voluntary. However, only for the qualitative interviews component, if one parent decides not to register, the whole family would not be eligible as this component examines co-parental interactions and the role each parent plays to support their child’s eating and physical activity behaviors and thus requires both parents to participate.

(Modifications to recruitment from February, 2024)
Eligible centers from Victoria, BC, will also be invited to participate in the study for Aim 4 main study and sub-study. Recruitment procedures will remain the same as the procedures described for centers recruited in the Greater Vancouver area.

(May 17, 2024) The sample size for the qualitative interviews for the parenting substudy was increased to 25 families instead of 15 families as we want to interview some control group families as well (initially we were including only intervention group families). Note that there will be no change in the interview guide, procedures for recruitment or interviewing these new families, or incentive. Everything will remain the same as the intervention group families interviewed, with the only difference that the interview guide contains a subset of questions regarding the perception of the intervention, which will not be asked to control group families as they are not appliable to them.

(Additional recruitment strategy - PAA Dec 20, 2024)
We have created an email invitation which would be sent to childcare facilities by the BC Director of Licensing Standard of Practice and mailing list that Child Health BC has. We are currently experiencing issues in recruiting childcare centers and we have talked with our partners on this project, Child Health BC, who indicated that an email from them and the BC Director of Licensing would likely not be considered as a spam email and may be read by those who receive it. These email invitations would be externally sent and include a link to our REDCap project for those interested. This is a new REDCap project created for this specific purpose, but it collects the same information we are already collecting in paper from the interested facilities that we are recruiting. When participants click on this link, prior to providing any data, they are shown the full consent that is currently REB-approved and that all facilities receive, and then they can register if interested. Once interested facilities complete this information in REDCap expressing their interest in our project, our team will contact them to assess their eligibility and move forward with enrollment if applicable, following the exact same processes that have been already described and approved for this project.

(ATP interviews, March 4, 2025) Qualitative interviews will be conducted with educators and managers after they used and/or had access to the ATP+ app for a period of 3-month. In the ATP+ consent that participants completed, participants were already informed that a subsample will be invited to qualitative interviews, so this new consent is for them to enroll in the interview. Prior to inviting any participants to the interview, we will assess whether the participants were assigned to the intervention condition receiving access to the ATP+ app at baseline, as this will determine their eligibility. All qualitative interviews will be conducted online using Zoom. Multiple modalities will be employed to invite educators and managers to engage in these interviews, including an email and/or printed letter sent to eligible educators and managers. Research staff will follow-up with either emails or calls to provide more information about the interviews and assess educators and managers’ interest in

	participating in this supplemental study. The emails/letters will be sent 2-3 weeks prior to the 3-month follow-up to allow for enough time for participants to review the consent form before the research team goes to the facility again. Interested participants will have the option to enroll in the interviews by completing the paper consent form or by consenting online via REDCap e-consent framework, which will provide the same detailed information as that shown in the printed consent. Eligible educators and managers who have already completed their follow-up assessments will also be invited to provide insights about the ATP+ program via emails and/or phone calls to discuss their interest in participating in the interview. If interested, they will be directed to the REDCap e-consent project for them to enroll online via REDCap e-consent framework.
5.5. Use of Records<br><br>*If existing records (e.g., health records, course grade sheets or other records/databases) will be used to access information about potential participants, please describe how permission to access this information, and to collect and use this information, will be obtained.*	As stated in section 5.4, we will also identify potential childcare facilities participants via publicly available online information such as but not limited to maps.gov.bc.ca and healthspace.ca. In our 2016/17 data collection, we collected enough information to identify which childcare facilities completed our survey. We will use this information to supplement our list of facilities. The data from all waves 2016/17, 2018/19, 2019/20, and 2022/23 will be linked but no identifiers will be stored with the data.
5.6. Summary of Procedures<br><br>*Describe briefly in a step-by-step manner what the researcher will be doing with participants, after they have been recruited and consented.*	MANAGERS AND STAFF SURVEYS (AIMS 1, 2 (quantitative), and 3)<br>All of our web-based survey will be administered using the Qualtrics platform at UBC (BCCHR). The questionnaires are specifically designed to measure PA and HE policies and practices in the early years setting. Managers will complete the Manager Questionnaire (Appendix EE), childcare staff will complete the Staff General Questionnaire (Appendix FF), and/or the Staff Today Questionnaire (Appendix GG), and short program staff will complete the Short Program Staff Questionnaire (Appendix HH), at a time and location most convenient for them. Participants will also be given the option to complete a printed version of the survey if they wish t and then mail/fax it back. Managers and staff complete these questionnaires as part of AIMS 1, 2, and 3. All these surveys were approved by the UVic Harmonized REB application. We will be amending these surveys to meet the objectives of the SSHRC and CIHR grants and modifications of these questionnaires will be submitted at a later date.<br><br>MANAGERS AND STAFFS SEMI-STRUCTURE INTERVIEWS (AIM 2 qualitative) The consenting participants will participate in telephone and/or in-person interviews. The interview dates/times will be scheduled according to the participants' availabilities and will last approximately 40 minutes. A trained researcher will facilitate the interview, following the interview scripts (Appendices AA). The researcher will take notes and/or audio record the interview, which will then be transcribed.<br><br>PILOT DATA COLLECTION (AIM 3)<br>After receiving all forms from the manager + 3 ECE from their facility, participants will receive access to the APT+ program with enrolment instructions via email. After competing the 3 online modules, we will schedule a 1-hour individual and/or focus-group interviews online via Zoom, according to the participants' availabilities so they can share their expert opinion about these resources. A trained researcher will facilitate the interviews, following the interview scripts. The researcher will take notes and/or audio record the interviews.<br><br>CHILD DATA COLLECTION (AIM 4)<br>AIM 4 involves a more intensive study on the consented childcare facility environments and the impact of changes in policies and practices on consented children's (ages 3-5 years old) PA and HE behaviours and includes the following components:<br>a) The Good Start Matters Equipment Environment Space observation sheet (Attached in Section 9.5) provides greater detail on the protocol that will be used. Briefly, this tool requires direct observation to assess facilities' environments, children's PA and nutrition

behaviours throughout the childcare day. The researchers will conduct 2 full days of observations in each childcare and children will wear the physical activity devices for 4 days.
b) Wearable devices: Children will wear two devices: 1) the AX3 Axivity accelerometer (Axivity, Newcastle Helix, UK) and 2) the GPS forerunner 230 watch (Garmin, KS) as GPS devices have higher accuracy to detect indoor and outdoor time of preschoolers.

An accelerometer (aka wrist monitor) is a small, non-invasive device that tracks PA levels. The consented children will wear one during childcare hours for three full days at each time period that they consent to. A trained researcher will put the accelerometers on the children in the morning and take them off at the end of the day.
c) Assessment of Fundamental Movement Skills (FMS): All children in childcare will participate in the FMS activities as a part of their PA for the day (approximately 1 hour each time period). Only the consented children's FMS will be assessed as part of the PA sub-study. The valid and reliable TGMD-3 tool (Appendix JJ) will be used, in which trained researchers will demonstrate various FMS to the children, and then video record the consented children doing them. Their skills will then be analyzed by a trained researcher. Video recordings of children doing the FMS testing will only be used for analysis only and will destroyed as soon as the skills have been analyzed.
d) Policy document: The staff will also be asked to provide the researcher with their facilities' policy documents, as outlined in the Good Start Matters Equipment Environment Space observation sheet (Attached in Section 9.5).
e) Some centres will also be asked to participate in a recording of lunchbox contents. A digital photo and paper recording of contents will be taken. Detailed protocol of this assessment can be found in Section 9.1 Lunchbox Dietary Data Collection Protocol, and associated forms in 9.5 entitled School Food Checklist.
f)Parents will be asked to complete a short survey about their child asking for demographic information, child's physical activity behaviour, and structured physical activities the child participates in, the associated form is in Section 9.5 entitled parent survey.
Note: All these child data collection procedures were approved in the UVic Harmonized REB application, except we added the GPS device as this was missing from our UVic application

THE INTERVENTION ATP+ (AIM 4):
(old content approved): After receiving consent from at least 60% of ECE (~1 manager + 2 staff) childcare centres will complete the baseline assessments at child and facility levels. Following, half of the centres will receive the ATP+ program immediately or 3 months after. Measures will be assessed again 3 months later.
A subset of managers and educators (~30) will participate in interviews to discuss their experiences in using the ATP+ program. We will randomly select participants and those who are selected will receive an invitation package and will be given the opportunity to participate in this optional component of the study. We will conduct qualitative interviews to understand how the Early Childcare Educators and managers are using the ATP+ intervention. The invitational package (will include a letter and consent form) and content of these qualitative interviews will be developed at a later time by a PhD student who has an interested in evaluating the effectiveness of the ATP+ intervention. This component of the study will be submitted at a later date to REB and no interviews and invitation will proceed until we have REB approval.

(modifications on Nov 22, 2023): Eligible and interested managers and educators would: 1) complete the online ATP+ program – where 26 facilities would start the program after the baseline assessments are completed and the other 26 facilities would receive the program 3 months later; and 2) complete online surveys at 2 time points (baseline and 3-month). The survey take about 30-35 minutes to complete. In addition, one staff would coordinate the 3-day data collection at their facility which would be done by our research staff and include: 1) Observing children's active play and the activities provided by the educators (1 full day, observation form is the document named "RCT(Aim4) Modified EPAO_v10" approved in the PAA of April 27, 2023); and 2) Administering a Test of Gross Motor Development (TGMD-3) that assess manipulative, locomotor and balancing skills and takes about 15-20 min to administer per children.
A subset of managers and educators (~30) will participate in interviews to discuss their experiences in using the ATP+ program. We will randomly select participants and those who

are selected will receive an invitation package and will be given the opportunity to participate in this optional component of the study. We will conduct qualitative interviews to understand how the Early Childcare Educators and managers are using the ATP+ intervention. The invitational package (will include a letter and consent form) and content of these qualitative interviews will be developed at a later time by a PhD student who has an interested in evaluating the effectiveness of the ATP+ intervention. This component of the study will be submitted at a later date to REB and no interviews and invitation will proceed until we have REB approval.

SUB-STUDY (AIM 4):
A Mixed Methods study will be integrated into the Good Start Matters ATP+ Randomized Controlled Trial (RCT). Specifically, parents of children attending the invited childcare centers will be invited to enroll into the Good Start Matters Parenting study. The Good Start Matters Parenting study is a 2-arms RCT where families are randomized into a control or an intervention condition. Intervention group families receive a parenting intervention though a mobile-health (mHealth) app, namely Pathverse, to promote positive feeding, activity and media parenting practices and support healthy child behaviors. Data collection occurs at baseline and at 10 weeks. After the 10 weeks assessment, control group families will get access to the app. Participants (2 parents or 2 primary caregivers) will complete 3 types of measurement: a) questionnaires inquiring about their usual behaviors and child behaviours, b) questionnaires inquiring about their behaviors in a particular day – an Ecological Momentary Assessment (EMA) of their behaviors every day during a 14-day period at each data collection time point (this is a short questionnaire that takes 5-10 minutes a day to complete. Please note that there is no cost to participants for using or downloading the app, and that any cost related to the app will be covered by the research team.); and c) a 1-hour Zoom interview (only a subset of 15 families) to inquire about their parent-coparent-child interactions and their experiences while using the app. Interviews will be done only at 10-weeks – not baseline- and will be recorded (we will schedule the interview according to the participants' availability and a trained staff will facilitate the interviews, following the interview scripts).
Pathverse is a platform that was designed to minimize the cost associated with developing an app. The app includes templates which app developers can use and our study will use the templates that have been already developed. It will cost us about $300 per month to use the app and this cost is associated with storing our data. Based on our initial look at the app, we may require some customization which would cost us about $2000. The research team does not have any financial interest in the platform nor would we get any revenue from using the platform.
The app-analytics data related to the Parenting intervention are stored on the Pathverse app whereas the data derived from the measurement tools are stored in REDCap. These app-analytics data derives from parental interactions with the app (i.e., the usability data related to parental use of the features used in the app while setting a goal in the app, completing a self-monitoring check-in in the app, interacting with other parents in the app, etc.). The data remain in the Pathverse platform for up to a 1 year after the study ends. The research team will extract all the data from the app at the end of the study.

Temporary change tor procedures (Starting Oct 13, 2023) ; Given that we are collecting repeated data for the parenting study, we need to send multiple automated survey invitations from REDCap. The REB has approved that we transfer parent email and cellphone from the REDCap Consent project to the Survey project so that we can the survey multiple times (email is needed to complete the survey more than 1 time and the phone is needed to complete the daily diary data). This transfer of data needs to be programmed by the REDCap team. We have submitted this request to the REDCap team in August and they anticipate having the programming done by Mid-November given that they are short staffed. We are requesting approval to ask parents to provide their emails and cellphones as part of the REDCap Survey project while we wait for the REDCap team to transfer this data from the Consent project. This is a temporary solution while we await for the programming to be complete. Note that this temporary approach does not change already approved REDCap dataset structures and data storage, we would only skip the data transfer process temporarily and parents would be asked twice to provide their email and phone number.

	(February, 2024) Eligible centers and participants from Victoria, BC, will also be invited to participate in the study, but procedures will be the same as those used with centers in the Greater Vancouver area.<br><br>(May 17, 2024) For the parenting interviews sub-study, we will increase our sample from 15 to approximately 25 families as we decided to interview 10 additional families from the control group (initially we had planned to interview only intervention group participants). Note that there will be no change in the interview guide, procedures for recruitment or interviewing these new families, or incentive. Everything will remain the same as the intervention group families interviewed, with the only difference that the interview guide contains a subset of questions regarding the perception of the intervention, which will not be asked to control group families as they are not applicable to them.<br><br>(Additional recruitment strategy - PAA Dec 20, 2024)<br>We have created an email invitation which would be sent to childcare facilities by the BC Director of Licensing Standard of Practice and mailing list that Child Health BC has. We are currently experiencing issues in recruiting childcare centers and we have talked with our partners on this project, Child Health BC, who indicated that an email from them and the BC Director of Licensing would likely not be considered as a spam email and may be read by those who receive it. These email invitations would be externally sent and include a link to our REDCap project for those interested. This is a new REDCap project created for this specific purpose, but it collects the same information we are already collecting in paper from the interested facilities that we are recruiting. When participants click on this link, prior to providing any data, they are shown the full consent that is currently REB-approved and that all facilities receive, and then they can register if interested. Once interested facilities complete this information in REDCap expressing their interest in our project, our team will contact them to assess their eligibility and move forward with enrollment if applicable, following the exact same processes that have been already described and approved for this project.<br><br>(ATP interviews, March 4, 2025) Qualitative interviews will be conducted with educators and managers after they used and/or had access to the ATP+ app for a period of 3-month. In the ATP+ consent that participants completed, participants were already informed that a subsample will be invited to qualitative interviews, so this new consent is for them to enroll in the interview. Prior to inviting any participants to the interview, we will assess whether the participants were assigned to the intervention condition receiving access to the ATP+ app at baseline, as this will determine their eligibility. All qualitative interviews will be conducted online using Zoom. Multiple modalities will be employed to invite educators and managers to engage in these interviews, including an email and/or printed letter sent to eligible educators and managers. Research staff will follow-up with either emails or calls to provide more information about the interviews and assess educators and managers' interest in participating in this supplemental study. The emails/letters will be sent 2-3 weeks prior to the 3-month follow-up to allow for enough time for participants to review the consent form before the research team goes to the facility again. Interested participants will have the option to enroll in the interviews by completing the paper consent form or by consenting online via REDCap e-consent framework, which will provide the same detailed information as that shown in the printed consent. Eligible educators and managers who have already completed their follow-up assessments will also be invited to provide insights about the ATP+ program via emails and/or phone calls to discuss their interest in participating in the interview. If interested, they will be directed to the REDCap e-consent project for them to enroll online via REDCap e-consent framework.
5.7. Research Types<br><br>*Select all that apply to your study. Please review the research methods descriptions before responding. If none*	Naturalistic Observation<br>Community Based Research (collaboration with community on design and methods)<br>Videotaping<br>Expert Interviews

apply, please select "None of these Methods"	

**6. Participant Information and Consent Process - Behavioural Study** [View Form]	
6.1. Time to Participate	Childcare staff (Groups 3 and 4) should take about 20 minutes to complete the surveys.<br>Children (groups 6) will wear the accelerometers from the time they arrive at the childcare facility and until they leave and the FMS test is expected to take 15 to 20 minutes to complete.<br>AIM 3: for the pilot, staff will spend 60-90 min/weeks for a total of 3 weeks on the online intervention plus 60 min on an interview.<br>Aim 4-part 1: staff will spend 30-40 min on the survey.<br>Aim 4 – Childhood educators and Managers will spend 90 min/weeks for a total of 3 weeks on the online intervention. They will spend about 45 minutes completing the questionnaire at baseline and again at 3-month.<br><br>Aim 4 - sub-study: Participants who volunteer for the evaluation of the parenting intervention will spend15-20 minutes on the intervention for 9 weeks, need 15-20 minutes at baseline and 10 weeks to complete the assessments. In addition, the Ecological Momentary Assessment is completed at baseline and at the 10 weeks mark, will take 5-10 min a day and is completed for a duration of 14 days at each occasion. Participants who volunteer to be interviewed will devote 60 minutes at the 10-weeks to complete 1 interview.<br><br>(ATP interviews, March 4, 2025) The ATP qualitative interviews will take between 45-60 minutes.
6.2. Risks and Mitigation	An observer will be in the childcare facility collecting data and the childcare staff might feel self-conscious about what they are doing in front of the data collector. There is no risk to managers and staff who are not abiding by the provincial guidelines as indicated in their survey responses and/or by our observations since these guidelines are voluntary and not mandated.<br>The consent process will minimize the risk as only those who are willing to have an observer in their setting will volunteer. The consent will emphasize that we are interested in the overall policies and practices of the childcare facility and that the observation is of the children’s PA and eating behaviours. All facility names will be coded and participants’ names will be removed from the surveys once they have received their gift cards. For those who are participating in the longitudinal study, their names will be coded and only used to re-contact them each year.<br>The key to addressing the issue is being empathetic and establishing trust through communication. If a childcare staff member feels uncomfortable we will remind them that we are observing the children’s PA and eating behaviours. If necessary we will not complete the observation. Our experience has been that the staff quickly forget that a member of the research team is in their facility as they are busy tending to the children’s’ needs and we make a point of being as unobtrusive as possible.<br>Safeguards taken to ensure voluntariness and minimize undue influence, coercion or potential harm:<br>The research team rather than the PI will recruit the Advisory Committee members. A presentation will be made to the Advisory Committee but individual invitations and consents will be sent out so there is no ‘group’ or ‘PI’ pressure and individual interviews will be conducted rather than a focus group so they have the ability to refuse rather than feel social pressure to participate.<br>The recruitment materials/consent forms will include the following clauses to ensure participant voluntariness and minimize undue influence: (1) the stakeholders/managers are not conducting the study; (2) relationships and employment will not be affected in any way whether managers/staff choose to participate or not; and (3) the stakeholders/managers will not be aware of who chooses to participate and who does not.<br>Another possible inconvenience includes the amount of time taken to complete the surveys and interviews. Some of the children may find the accelerometers (aka wrist monitor) slightly irritating to wear, but our experience is that most children do not even notice that they are wearing them after the initial novelty wears off. Childcare staff might feel self-conscious with an observer present.<br>The lunchbox observation may pose an increased risk to children with food allergies, therefore we will exclude all children with a reported food allergy in order to prevent any

	cross contamination that could occur.<br><br>There are no known risks to children participating in the evaluation of the ATP+ program. Some of the children may find the accelerometer (aka wrist monitor) slightly irritating to wear, but our experience is that most children do not even notice that they are wearing them after the initial novelty wears off. Parents and legal primary guardians who utilize the SMARTER parenting app will engage in more discussions about their parenting practices and for some parents this may helpful while for others this may take time and highlight areas where they may not agree with their partners, This may create some discomfort for some families and a list of resources will be provided to parents / legal guardians if feel they need more support.<br>Among the ATP+ course activities, participants will have the option to post comments on the discussion board and interact with fellow participants by liking or replying to the posts. Every post includes the student name, date and time. Although our expectation is that these interactions will remain positive and professional, it is possible for someone to post an inappropriate comment. To mitigate any negative interactions, staff members will monitor every week the discussion board by:<br>1. enabling the notifications feature to receive an email every time a new post is added; and<br>2. reviewing every week the content of the discussion board and act as a moderator to eliminate inappropriate comments.
6.3. Potential Benefits	To Participants: The stakeholders and delivery agents will get to be actively involved in the implementation process and evaluation of PA and HE guidelines. Childcare managers and staff and short program staff will be provided with a greater understanding and/or a heightened awareness of PA and HE policies and practices in their early-years facilities/programs. In late 2016 they will be given provincial practice guidelines targeting PA and HE in early-years settings with capacity-building interventions (training, resources, support). The children may also gain a heightened awareness of PA.<br>The evaluation of the ATP+ program and the SMARTER parenting intervention will help inform policy development and the implementation of early years initiatives in British Columbia and Canada, and may have direct implications for the prevention of diseases associated with unhealthy lifestyle behaviors.
6.4. Impacts on Community	Benefits to Society: Improving children's PA and eating habits in the early years has been shown to track into adolescence and adulthood. This research may therefore have direct implications on the prevention of diseases associated with physical inactivity, unhealthy eating and overweight/obesity such as cardiovascular disease, diabetes and certain cancers.<br>Benefits to the State of Knowledge: This research will provide a greater understanding of the current PA and HE environments, policies and staff practices in early years settings prior to the release of provincial guidelines and capacity-building initiative (training, resources, support). It will also evaluate the impact of the guidelines and capacity-building efforts. Broadly, it will help inform future policy development and implementation in Canada and efforts to change practices in regards to PA and HE in the early years settings.
6.5. Reimbursement and Incentives	Groups 3-5 will be offered a small incentive ($5 e-gift card) for participating in the surveys. They will have the option of writing their name and contact information at the end of the survey if they would like an e-gift card. The other groups will not receive an incentive. Participants in Groups 3-5 who do not complete the portion of the survey that asks if they would like an e-gift card or provide their contact information will not receive an e-card. If they complete the survey and have provided their contact information, but then would like their survey data removed, they will get to keep the e-gift card if it has already been sent. There will be no compensation for participating in the research (e.g., for transportation, parking, childcare, etc.)<br>For Group 6: Participating facilities will receive $80, staff and managers who complete the 45-min survey will receive $25 each time they complete the survey, and parents of children who participate in the study will receive $25 for each 3 days of data collection.<br><br>Aim 3: we will provide $150 to educators and managers who pilot the content of the ATP+ program.<br><br>Aim 4-part 1: managers and educators who choose to participate will receive $25 cash and be entered to win $100 cash (1 in 30 changes of winning as there will be a draw for every

	30 participants). All compensations will be sent via e-transfer. Any remuneration or compensation that are not claimed within 21 days of being sent via e-transfer to the participant will be forfeited.<br><br>(August 23, 2023 - Changes made)<br>Aim 4 (RCT that evaluates efficacy of ATP+) - Childcare facilities will receive $75 for facilitating the data collection at their sites, and educators and managers will receive $50 for completing the survey ($25 for the baseline assessment and $25 for the 3-month assessment), pro-rated for the components completed. Participants that complete both baseline and follow up surveys will be entered in a draw to win $100 (1 in 30 chances of winning as there will be a draw for every 30 participants). All compensations for participants recruited prior to August 23, 2023, are sent via e-transfer, and in these cases, compensations not claimed within 21 days were forfeit. All compensations for participants recruited after August 23, 2023 will be sent via Amazon.ca gift after the 3-month follow-up is completed. Finally, educators and managers who complete the on-line training will receive a certificate for 5 credit hours of professional development if they completed the old training in Canvas, and 3 credits if they completed the new training in the Pathverse app.<br><br>Aim 4 - sub-study (Parenting RCT): Families who enroll in the evaluation of the parenting intervention will receive up to $214 gift card for their participation ($107 for each participating parent/legal guardian/primary caregiver – pro-rated if not all assessment tools are completed). Each participating parent/legal guardian/primary caregiver is compensated as follow: 1) $40 for completing two 15-20-minues survey at baseline and at the 10 weeks ($20 for each survey per parent) and 2) $42 for completing two daily check-ins for 14 days. The daily check-ins take 5-10 minutes to complete and are completed at baseline and at the 10 weeks ($1.5 for each daily check-in completed). In addition, each parent who enroll in the parenting interviews substudy will receive in total $25 gift card ($50 per family) for completing one 1-hour interview. Finally, parents/legal guardian/primary caregivers who complete both baseline and follow up long surveys will be entered to win $100 gift card (1 in 30 chances of winning as there will be a draw for every 30 participants). All gift cards will be Amazon.ca gift cards and will be sent directly from Amazon. As no parents were recruited for the Parenting RCT before August 23, 2023 (date in which compensations were changed from e-transfer to gift card), the only compensation to be used with parents is Amazon gift cards.<br><br>(ATP interviews, March 4, 2025) Participants completing the ATP qualitative interviews will receive $30 gift card paid using the same procedure as for the ATP RCT. In addition, all who take part in the interviews will be entered to win a $100 gift card.
6.6. Obtaining Consent<br><br>*Include details of where and when consent will be obtained and how it will be documented.*	Groups 1 and 2 will be provided with a consent form to be signed prior to participating in an interview. In terms of the Capacity Building Tracking form that Delivery Agents (Group 2) are asked to complete, they will be instructed to only use meeting participants' general titles (e.g. manager, staff) and not specific names in their records, as we do not require this identifiable information (the purpose is to track the capacity building process). This is outlined in the tracking sheet and in the consent form. As such we will not seek third party informed consent.<br>Groups 3 and 4 will be provided with an implied consent in the email where they are given their online survey link. Managers and childcare staff who agree to participate in the longitudinal study (to complete a survey in years 2, 3, and 4 as well) will be presented with an implied consent and the survey link each year. Managers and childcare staff that are recruited for the year 3 survey will also be presented with an implied consent and survey link in year 3.<br>Group 5 will be presented with an implied consent in the email where they are given their online survey link in years 1, 2 and 3.<br>Group 6 – Two paths to recruitment to participate in the substudy will occur. Eligible childcare centres in the lower mainland will be contacted. Depending on if these childcare centres are single site organizations, or a larger childcare organization with multiple sites we will contact them directly or via the director/manager of the larger organization. If the

manager of the larger organization approved, we will then contact the individual single sites within the larger organization. This is outlined in 9.1 Action Steps Flow chart with R numbers. Once individual single sites have expressed interest, we will meet with managers or supervisors and staff to answer questions and provide consent documentation. Once sufficient staff (~80%) have consented, we will move to recruiting child participants. Research staff will provide the children's parents/guardians with a consent form to be signed prior to the children wearing accelerometers and completing the FMS testing. The children's assent will also be obtained prior to the FMS testing and each day prior to being fitted with the accelerometer. We will not ask the parents/guardians to provide consent to the facility observation as the observation will not be specific to individual children, but will instead be a survey of the program environment and practices at the facility. Due to the nature of this, we do not require consent as it will be impossible to single out child in the overall observation. This is noted in all of the child recruitment scripts which provides general information on the observations.

Parents/guardians of the children will be asked to consent at each measurement period (Fall of year 1, Spring of year 1, Fall of year 2, Spring of year 2). Data collection will include those children that initially agree to participate (Fall of year) in addition to children who did not consent initially but then consent at the subsequent time periods (i.e. Fall of year 2, Spring of year 1 and 2). Children will be asked to assent to the accelerometers each day they are put on, and each time they participate in FMS testing.

Group 8 Early Years providers that are interested will register for the Appetite to Play workshop on their own, and then will be offered a survey and consent form at the workshop. They may choose to sign the consent form and complete the surveys before and after the workshop and also self-select to be contacted for a qualitative interview or not.

The Training Coordinator at the YMCA works closely with the delivery agents and will send the email inviting them to participate in an interview. The email will contain the script attached in Appendix MMM. Delivery Agent Interview Invitation.

Withdrawal

Participants will be told in the consent letters/implied consent that they are able to withdraw from the research at any time. All consents say "If you do decide to participate, you may withdraw at any time without any consequences or any explanation." Participants are asked to contact the research team in person (during an interview) or by telephone/email (contact information is provided).

INTERVIEWS If a participant in Groups 1 or 2 or Pilot would like to withdraw from an interview after consenting but prior to it taking place, they would be asked to notify the corresponding researcher by telephone or email. If they would like to withdraw during the interview, they will be told that they can just notify the interviewer and they can have their data removed from the study.

SURVEYS Participants in groups 3-5 can stop completing the survey at any time; the research team would not know since the surveys are anonymous. Those who are completing the cross-sectional survey (years 1 and 3) will not be able to withdraw their survey data once submitted since there are no identifiers on the survey. However, participants who have submitted the survey and have included their name for the incentive and/or to participate in the longitudinal study and are therefore using an identifier on their survey (in years 2-4), can have their data removed upon request. If they have already been sent the e-gift card incentive they will be able to keep it.

PA/HE SUB-STUDY IN CHILDCARE FACILITIES If a child in Group 6 would like to withdraw from wearing the accelerometer or participating in the FMS testing, he/she would just have to tell the researcher or the childcare staff, or the parent/guardian could notify the corresponding researcher by telephone, email or a note sent by way of the childcare staff.

If participants in Groups 3-5 have identified themselves for the e-gift card and/or to participate in the longitudinal study and then wish to withdraw, they will be sent an email asking if their data could still be used and explaining that it would be anonymous in the results/final report. Participants in the longitudinal study (years 2-4) who are including their unique identifier on their surveys (given by the corresponding researcher) can request to have their data removed by emailing by the researcher. Anyone who wishes to have their data removed will be completely erased from the records. If participants have NOT identified their desire for an e-gift card or to participate in the longitudinal survey, we cannot identify their survey as it would be anonymous.

If a participant in Group 6 wishes to withdraw, the parent/guardian will be told on the consent form "If you choose to withdraw from the study, and do not want your accelerometry

and FMS data included, please contact the researcher." It will not be used in the analysis and will be destroyed. Upon request for interviews, surveys with participant's names included, accelerometry and FMS testing. It is logistically impossible to remove individual participant data.
AIM 3: eligible childcare centres in the Vancouver will be contacted. If the manager approves, they will receive a copy of the ECE consent form via email. Managers will invite their ECE to participate in the study by sharing the consent form and let them know that they would need to share their names and emails with the research team. Interested ECE will provide their contact information to the research team to receive an email with the link to the consent form via REDCap survey. If they provide consent, they will be ask some demographic questions (same questions as the previously approved for group 3 and 4) and will not include any other information (similar to the previous approval). Participants will have ample time to review the consent to participate in the pilot testing the content of the ATP+ program.

(old content approved)
Aim - for Aim 4: Those who expressed an interest in the study will be invited to have a zoom meeting with our team so that we can provide the facility with more information about the study or alternatively they can access the presentation about the study on their own. Those who are interested to proceed with the study will be mailed or emailed an invitation package that includes an invitation letter and a copy of the consent. After they have had the opportunity to review everything they can move ahead to be part of the evaluation of the ATP+ program. Registration is done online or on paper. The online/paper registration has the invitation, the consent form which participant need to review and consent to participate and then they can register. As for the recruitment of the parents, childcare providers will facilitate delivery of invitation packages (includes invitation letter and a copy of the consent form) to parents which may include having the staff giving the invitations to the parents directly, emailing the invitation, or allocating a time when one of our staff can deliver the invitation packages to parents and answer any questions they may have about enrolling their child into the ATP+ program evaluation. The invitation letter includes a an online link that parents can use to register their child. The link includes the invitation letter, the consent form which they complete online and if they consent they then register their child into the study. In addition, participants will have the option to fill out the paper forms of the consent form and contact information form which will be collected by research staff at a later date.

Aim 4 - substudy (Parenting RCT): If parents wish to enroll in the study they will need to access the registration site and consent for their participation from the email they received from the research team, or they can provide their consent in paper by signing the printed forms and giving the signed forms to our research staff. It will be made clear that they do not need to participate even though a person close to them provided us with their information. As the sub-study is a co-parenting intervention, we need to invite both parents but families can still proceed with the study if the person they listed as their partner for the intervention elect to not participate. We will also clarify that even if they sign the printed forms, we will assess their eligibility before enrolling them into the study, and they will be notified via email if they do not meet eligibility criteria.

(modifications from Nov 22, 2023)
Aim - for Aim 4: Those who expressed an interest in the study will be invited to have an in person meeting with our team so that we can provide the facility with more information about the study. At the initial meeting, each staff receives a brochure, invitation, consent form, and copy of the questionnaire. After staff and manager have had the opportunity to review the invitation materials (1-2 weeks after the initial meeting), the research staff will call the manager to assess initial interests in the study and schedule a second in-person meeting to clarify remaining question and collet the forms. Registration, consent and baseline survey are completed on paper but follow up survey is done either on paper or online via a REDCap URL sent directly to their email which is obtained during the consent process – this link includes a de-identified numeric code that is unique for each participant. As for the recruitment of the parents, childcare providers will facilitate delivery of invitation packages (includes invitation letter and a copy of the consent form) to parents which may include having the staff giving the invitations to the parents directly, emailing the invitation, or allocating a time when one of our staff can deliver the invitation packages to parents and

	answer any questions they may have about enrolling their child into the ATP+ or the Parenting programs. The invitation letter includes an online link that parents can use to register. The link includes the invitation letter, the consent form which they complete online (e-consent using REDCap framework) and if they consent they then register their child into the ATP and/or Parenting studies. In addition, participants will have the option to fill out the paper forms of the consent form and contact information form, which will be collected by research staff at a later date.<br><br>Aim 4 - substudy (Parenting RCT): Parents enroll for the Parenting study following the same process as for the ATP study. If parents wish to enroll in the Parenting study they will need to access the registration website and consent for their participation online, which can be done from the email they received from the research team, or by scanning a QR code that we print in the printed paper forms. When consenting online, parents consent via REDCap e-consent framework. Alternatively, parents can provide their consent in paper by signing the printed forms and giving the signed forms to our research staff, in which case we would manually add their information into REDCap. It will be made clear that they do not need to participate even though a person close to them provided us with their information. As the Parenting sub-study is a co-parenting intervention, we need to invite both parents but families can still proceed with the study if the person they listed as their partner for the intervention elect to not participate. We will also clarify that even if they sign the printed forms, we will assess their eligibility before enrolling them into the study, and they will be notified via email if they do not meet eligibility criteria.<br><br>(March 4, 2025) In the ATP+ consent that participants completed, participants were already informed that a subsample will be invited to qualitative interviews, so this new consent is for them to enroll in the interview. Multiple modalities will be employed to invite educators and managers to engage in these interviews, including an email and/or printed letter sent to eligible educators and managers. Research staff will follow-up with either emails or calls to provide more information about the interviews and assess educators and managers' interest in participating in this supplemental study. The emails/letters will be sent 2-3 weeks prior to the 3-month follow-up to allow for enough time for participants to review the consent form before the research team goes to the facility again. Interested participants will have the option to enroll in the interviews by completing the paper consent form or by consenting online via REDCap e-consent framework, which will provide the same detailed information as that shown in the printed consent. Eligible educators and managers who have already completed their follow-up assessments will also be invited to provide insights about the ATP+ program via emails and/or phone calls to discuss their interest in participating in the interview. If interested, they will be directed to the REDCap e-consent project for them to enroll online via REDCap e-consent framework.
6.6.A. Waiver of Consent	
6.7. Time to Decide	At least 24 hours prior to the procedure
6.8. Capacity to Consent	No

Will participants have the capacity to give fully informed consent on their own behalf?	
***6.8.A.** Provide details of the nature of the incapacity (for instance, young age, mental or physical condition).*	Young age
***6.8.B.** If a participant does not have the capacity to give fully informed consent, who will consent on their behalf? Ensure the relevant consent form (parent/caregiver, substitute decision maker, legally authorized representative) is attached to page 9.*	Group 6 – Parents/guardians of the children will be asked to consent at each measurement period (Fall of year 1, Spring of year 1, Fall of year 2, Spring of year 2). Data collection will include those children that initially agree to participate (Fall of year) in addition to children who did not consent initially but then consent at the subsequent time periods (i.e. Fall of year 2, Spring of year 1 and 2). Children will be asked to assent to the accelerometers each day they are put on, and each time they participate in FMS testing. We will also obtain signed or verbal assent for non-competent participants (children).
***6.8.C.** If a participant does not have the capacity to give fully informed consent, will they be able to give assent to participate?*	yes
***6.8.D.** If yes, explain how assent will be sought. Please be sure to attach copies of the assent form to page 9.*	We will obtain signed or verbal assent for non-competent participants (children)
6.9. Ongoing Consent	
6.10. Provisions for Consent (e.g., special assistance, Braille, translations/translator)	
6.11. Restrictions on Disclosure	
**7. Number of Participants - Behavioural Study** [View Form]	
7.1. External Approvals<br><br>***A.** Other Institutions:*	no
***B.** Please select "Add" to enter the name of the institution and attach the approval letter if received.*	**Name of Institution**
***C.** Other Jurisdiction or Country (if "NO," go to 7.1.G):*	no
***D.** Please select "Add" to enter the name of the jurisdiction or country and if you have already received approval attach the approval letter.*	**Name of Jurisdiction or Country**

E. *Has a Request for Ethics Approval been submitted to the institution or responsible authority in the other jurisdiction or country? (**Append a copy of any such document to this application once it is received**).*	no
F. *If a Request for Approval has **not been** submitted, provide the reasons below:*	
G. *Does this research focus on Indigenous peoples, communities or organizations?*	no
G.1.A. *Will the research be conducted on Indigenous reserves, Métis settlement(s), or lands governed under a self-government agreement or an Inuit or First Nations land claims agreement?*	
If yes, please provide details:	
G.1.B. *Do any of the criteria for participation include membership in an Indigenous community, group of communities, or organization, including urban Indigenous populations?*	
If yes, please provide details:	
G.1.C. *Does the research seek input from participants regarding a community's cultural heritage, artifacts, traditional knowledge or unique characteristics?*	
If yes, please provide details:	
G.1.D. *Will Indigenous identity or membership in an Indigenous community be used as a variable for the purposes of analysis?*	
If yes, please provide details:	
G.1.E. *Will the results of the research refer to Indigenous communities,*	

peoples, language, history or culture?	
If yes, please provide details:	
G.2. Community Engagement<br><br>***G.2.A.** If you answered yes to questions a), b), c), d), or e), have you initiated or do you intend to initiate an engagement process with the Indigenous collective, community or communities for this study?*	
***G.2.B.** If you answered "Yes" to question G.2.A., describe the process that you have followed or will follow with respect to community engagement. Include the role or position of those consulted, including their names if appropriate. Attach any documentation of consultations (i.e. formal research agreement, letter of approval, email communications, etc.) below.*	
Attachment:	
G.3. No community consultation or engagement<br><br>*If you answered "no" to question G.2.A., briefly describe why community engagement will not be sought and how you can conduct a study that respects Indigenous communities and participants in the absence of community engagement.*	
***H.** Registration for Publication of Clinical Trials.*	yes
If 'Yes', click 'Add' to enter the following information.	**Has it been registered?** / **Indicate the Authorized Registry used:** / **Enter your Clinical Trial unique identifier:**<br>yes / ClinicalTrials.gov / NCT05669378<br>yes / ClinicalTrials.gov / NCT05802160

7.2. Number of Participants<br><br>***A.*** *How many participants will take part in the entire study (i.e., world-wide)?*	3000
B. *How many participants will take part at institutions covered by this Research Ethics Approval?*	3000
7.3. Principal Investigator and Research Team Experience	The principal investigators and co-investigators have full research qualifications and have managed many community-based PA and HE research projects with young children. They offer expertise in implementation science, PA and HE measurement, policy assessment, childcare and school-based interventions and knowledge translation, all of which support success of the project.
**8. Confidentiality - Behavioural Study** [View Form]	
8.1. Security of Data During the Course of the Study	Hard copies of the data (i.e. surveys, data collection sheets, interview notes), video files and audio files will be stored in a locked file cabinet in a locked room at UVic/UBC. Electronic files of de-identified data will be stored on a secure network drive at UVic/UBC which is accessible only to the investigators and research team. Electronic surveys will be kept in Qualtrics.<br><br>The parent, child and educator data will be be collected in REDCap in a different project (i.e.. REDCap “Childcare Providers" and "RedCap "Childcare Families"). As the ID, email address, and child first name need to be in the “Childcare Families" , we will work with the REDCap team to write a DET (data entry transfer) to securely move information from the REDCap “RedCap "Childcare Contact" project into the Redcap "Childcare Providers" and "Childcare Families" data projects. We have used this strategy to separate the contact information in previous REB approved study. The email addresses are used to send the questionnaire and other study reminders. The children's first names will be used because it is likely that multiple children from the same family will attend the same childcare centre.<br><br>The videos of children performing FMS activities will be used to assess the FMS skills after the fact. The audiotapes of the interviews will be used to transcribe the interviews so that they can be themed. The data (from surveys, interviews, observations, accelerometers and FMS testing) will be used to prepare a report for stakeholders, and all participating groups upon request. Academic papers may be published. Presentations may be made to stakeholders and at academic conferences.<br><br>After the workshops, the de-identified surveys and consent forms are separated by the regional trainers. The surveys are mailed to the YMCA of Greater Vancouver where they are transferred to Jennifer McConnell-Nzunga at UBC/BCCHR offices in person during bi-weekly meetings. The surveys have to be processed at UBC because they have the OMR survey scanning software used to extract the data from the surveys. The surveys do not contain names just ID codes.<br>The consents are mailed to UVic by the workshop trainers where they are scanned and uploaded to the password protected UVic Research Netdrive.<br><br>The paper-based workshop surveys are currently being stored and managed at UBC/BCCHR. The paper surveys are scanned first through Remark Office OMR software to read the responses into a de-identified dataset that is saved on the UVic Research Netdrive, and second they are scanned and saved as PDFs to the UVic Research NetDrive. Then we will securely shred the paper copies. We are expecting 5,000 participants in the Appetite to Play Workshops and the pre-workshop survey is 4 pages and the post workshop survey is 6 pages resulting in up to 50,000 pages of survey data to store.<br><br>We wish to have identifiable data stored on the UBC secure servers, specifically the place of work of participants who completed an Appetite to Play Workshop in person or online (Groups 8 and 9) who provided this information.

We previously used UBC Qualtics for the EY (Early Yearys) surveys. To streamline the RCT data collection as part of Aim 4 all data is now collected in a single platform - REDCap. To ensure that the data remains protected, we will follow a similar previously approved strategy in which we will collect the surveys data in a project that is independent of the project that collects the contact information and consent.

As part of the evaluation of the ATP+ RCT intervention, we will collect web-analytic data. The intervention is delivered via the UBC Canvas Catalogue site (for the centers recruited before August 23, 2023) and via Pathverse app for the new centers recruited after August 21, 2023). All participants recruited before August 23, 2023, will get access to the ATP+ intervention via Canvas even if they access the intervention after August 23, 2023. All participants recruited after August 23, 2023, will get access to the ATP+ intervention via Pathverse app. Thus, there may be a period of about 3 months in which both intervention platforms are available, but each participant would only have access to one platform, depending on their recruitment date.
The extraction of data from CANVAS and Pathverse includes the modules the participants have completed, date of completion, and user name (in Pathverse, this is not an identifier but an id generated by us and based on participant id number to connect the usability data with REDCap. In CANVAS, this is participant email but after this merging process, the email will be immediately eliminated.

The Pathverse platform was developed by researchers at the University of Victoria, app-analytics data is stored in Canada, and process for storing and securing the data has been approved by Canadian REB (see detailed privacy policy included in attachment “2022-04-05_Pathverse Privacy Policy.docx”). This platform will be used for both ATP+ and Parenting RCTs.
Participants will enter an ID number into the app and this is the only code that will allow us to connect the app-analytics data from Pathverse with the data we collect in REDCap. Lesson completion data collected through Pathverse will be stored on Amazon servers in Canada. Although these data are stored in Canadian servers, Amazon is a US-owned and operated company. As such, there is a possibility that the app usability data may be accessed without our knowledge or consent by the U.S. government, in compliance with the U.S. Freedom Act. Data will be deleted from these servers after three years. Goal setting data that is added by the user is stored on the device and is strictly for display purposes. Therefore, uninstalling the app will permanently delete all data related to them.

Sub-study (AIM 4) Parenting RCT - Consent and contact information for subject who volunteer to sign up for the parenting sub-study will have their identifiers and contact information stored into REDCAP. All data will be collected in REDCap except for the app-analytics data and the project data will be stored in an independent project (a procedure we have used and that has been approved in the past by the REB). For the parenting sub-study the intervention will be delivered by the Pathverse platform.

We will use Twillo to send participants the REDCap link via text messages to complete the Ecological Momentary Assessment (EMA). REDCap sends the text messages through this third-party web service (Twilio (www.twilio.com)) but no data is collected via Twilio. All text messages will be routed through Twilio's Canadian servers. However, REDCap ensures that text message transcriptions do not stay in Twilio's logs but are removed shortly after being completed. This is done for security and privacy concerns (e.g., HIPAA), in which participants' phone numbers do not get permanently logged on Twilio's servers but instead remain securely in REDCaP. Thus, Twillo is an add-on to RedCap that allows us to send text message notifications to participants. We need to send text messages for this study because we will collect intensive longitudinal data on a daily basis for a period of 14-days at baseline and at 10 weeks. Given the importance of our participants to complete the EMA check-in (short survey) right when the link is sent to them, we are using Twillo to more effectively communicate with them and ensure they see the link at the moment is sent to them as email communications do not work well to collect just-in-time data. In addition, to be able to send daily EMA survey links to participants via text message, we need to store the participant’s cellphone number in the same REDCap project where the EMA survey is stored, but no analysis dataset will contain the cellphone number or any other personal identifiers.

	(June 21, 2023 PAA) Our study needs parents to complete questionnaires at 2 time points. REDCap allows us to send the first questionnaire link using an Alert from the Consent project without saving the email in the Survey project. However, the time 2 questionnaire cannot be triggered by the completion of the first questionnaire or by using an Alert from the Consent project; it requires saving the email in the Survey project. Specifically, to be able to redirect parents to the time 2 questionnaire within the Survey project, we need to use an Automated Survey Invitation (ASI) instead of an Alert. However, it is only possible to send an ASI from the project where the questionnaire is located, which in our case is the Survey project. Thus, we require the email address to be stored in the Survey project. In this REB submission, we are requesting approval to store the participant email with the parenting survey in the REDCap Survey project. To maintain confidentiality of the data, data extraction will be done without the identifier.<br><br>(June 29, 2023 PAA) Our current data architecture in REDCap includes, among others, 2 separate projects storing the results from our short and long surveys, namely EMA survey and Full survey respectively. REB previously approved storing cellphone with EMA survey, and email with Full survey. As previously mentioned, we require storing these identifier since REDCap ASIs can only be triggered if some contact information is available (and we extensively use ASIs in our study since we collect repeated daily data for a long period of time - 14 days at baseline and 14 at follow up, in addition to full baseline and follow up surveys). In this amendment, we request to combine the 2 survey projects in a single survey project, where both cellphone and email would be stored with this single survey instead of one per survey. All remaining personal information will be stored in different REDCap project and not in the survey project. For monitoring purposes (eg, randomization, reminders, incentives), we need to know when participants have completed each assessment and how many tools have been completed or still missing. However, if the survey completion information resides in multiple projects, we would only be able to monitor participants' progress by downloading survey information from each project, merging them, running a report outside of REDCap, and uploading the merged datasets back into the monitoring project. While this would not combine survey responses with identifiers, having all survey responses in a single REDCap project would reduce error, increase efficiency, and minimize the information we download and merge, which is a continuous process for the duration of the study. Note that for monitoring purposes, we only need to extract time stamps and number of surveys completed from the survey project but no survey response data, so confidentiality of participants' responses would be maintained. Likewise, for data analysis purposes, we don't need to extract any identifiers from the survey project as participants can be linked with their participant id number protecting their anonymity.<br><br>(August 23, 2023 PAA) As requested by the REDCap team, a summary of all REDCap projects involved in both RCTs (ATP+ & Parenting) as well as the information transferred between projects is summarized in the document named "RCT(Aim4)_REDCap_v1".<br><br>(Oct 19, 2023 PAA) The REDCap team has requested additional detail in our application. For Aim 4, when participants provide their consent online, it means the consent is completed via REDCap e-consent framework. In addition, when variables are transferred via survey distribution link, the value of the variable itself is included in the link, and participants are redirected to the destination REDCap project when they click on the link. Finally, while the electronic data transferred is being programmed, we have duplicated projects in REDCap to implement alternative data transfer processes detailed in our document named RCT(Aim4)REDCap_v2.
8.2. Access to Data	Only the research team members (listed on the REB certificate) will have access to the data. They will be made aware that the information is confidential and that data security must be maintained at all times.<br>Only the principal investigator (Masse) and the research coordinator (Buckler) will have access to identifiable (childcare site of participants from workshops) data stored on both UVic and UBC secure servers.
8.3. Protection of Personal Information	Consents of Group 8 – the forms include the study ID code to be able to match the consent form with the surveys that include no other ID's. The consent with name/IDs and the workshop surveys with IDs only are only together very briefly before they are separated into 2 piles and sent to 2 different locations which minimizes the chance of them being matched

	up outside of the study.<br><br>INTERVIEWS<br>For participants in Groups 1 and 2, data will be themed and all identifying information and features will be removed or changed in quotes in any report, manuscript or presentation. Each year a new recruitment script will be sent out to all stakeholders and delivery agents (regardless of prior participation) and as such, names will not be used to re-contact initial participants in subsequent years.<br><br>SURVEYS<br>Participants in Groups 3, 4 and 5 taking part in the longitudinal survey will be given unique numeric identifiers to be used in years 2, 3 and 4 of the study. Their names and contact information will be required to re-contact them each year, however, only the researcher contacting them will use this information. When the researcher contacts them in years 2, 3 and 4, they will provide the participant with their unique code which the participant can include on the survey to keep it confidential.<br>The contact information provided by survey respondents to receive an e-gift card will not be kept with the survey data. All survey data will be de-identified.<br><br>PA/HE SUB-STUDY IN CHILDCARE FACILITIES<br>Participants in Group 6 will be given unique numeric identifiers for accelerometry and FMS testing, which will be used on all of their data. In each of the subsequent data collection periods after the initial one, the child will be identified by their code (e.g. for FMS testing, a white board will be placed in front of the camera before each consented child does the skill, and the board will say the skill name and the child's code).<br><br>Once the children are fitted with the accelerometers, it is not obvious who is wearing them because they are small and generally worn on top of the pants but under a shirt. Being videotaped during the FMS activities will not be obvious because it is difficult to tell if the camera is on or off. It will be on a tripod pointed at the activity area and turned on when a consented child is performing their activity in the area. Video recordings of children doing the FMS activities will only for analysis and will be destroyed as soon as the skills have been analyzed.<br><br>Groups 1, 2, 6, and those in 3-5 who provide their contact information in the survey, will not be anonymous. Participants in Groups 3-5 who complete the surveys without including their contact information will be anonymous. The participants will be anonymous in the dissemination of results.<br>NEW: Registration lists from workshops will be provided to the UBC PI (Masse) and research coordinator (Buckler) with participant name removed, only the location of the participant's work will be stored on UBC servers. This data will only be used to determine the impact of the workshop on improvements in policies and practices in childcare centres participating in the child participant data collection (Group 6) and relates directly to Aim 1 and 2 of the CIHR grant.<br>We will include a note on the discussion board to indicate that posting is not anonymous: "All posting are NOT anonymous, please do not reply to this post if you want to remain anonymous".<br><br>(Aim 4) In REDCap projects we store data needed to communicate with the facilities, and which include identifiers such as the contact persons of the facility, either managers or someone else, phone numbers, emails, and facility address. When each staff from the facility consents, as shown in our consent forms, we also collect identifier information (eg, full name, phone, email, gender) which is again stored in REDCap.<br><br>(Aim 4 substudy) The phone numbers are used to send EMA text messages via Twilio – the REDCap approved provider for sending text messages to participants.
8.4. Transfer of Data<br><br>*Will any data be transferred (made available) to persons or agencies outside the lead*	no

University or Health Authority?	
If yes, describe in detail what information will be released, to whom, how the data will be transferred, how and where it will be stored and what safeguards will be used to protect the identity of participants and the privacy of their data. Attach the data transfer agreement if applicable.	With respects to data collected under groups 3, 4, and 6 no data will be transferred outside of the University.
8.5. Retention and Destruction of Data	All data will be retained by Dr. Masse on UBC secure servers for a minimum of five years following the last publication, after which point it will be destroyed. Hard copies of data will be shredded. Computer files and electronic surveys will be deleted. Video tapes and audio tapes will be erased. Video recordings of children doing the FMS skills will only be used for analysis and will be destroyed as soon as the skills have been analyzed.
8.6. Future Use of Data	With respects to data collected for groups 3, 4, and 6, there are currently no plans to use the data in the future. Since continuous monitoring can be of use for gaining a greater understanding of the data, we will ensure that the data is collected in way that it can be continuously linked and participants will be informed that this is our intention.<br>Data may be posted in an online repository in line with open science policies, and consent forms reflect this.
8.7. Feedback to Participants<br><br>*Please provide information regarding your plans for communicating study results to participants. See the guidance notes for more information and respond to the bullet points as needed.*	We intend to communicate our findings to stakeholders, childcare centres that participate in our study and if appropriate to parents.

9. Documentation - Behavioural Study [View Form]

9.1. Research Proposal

Document Name	Version	Date	Password (if applicable)	
CIHR funded grant	4	Tuesday, March 4, 2025		[View]
Parenting Protocol	2	Thursday, April 27, 2023		[View]
Parenting Protocol	1	Friday, October 28, 2022		[View]
CIHR funded grant	3	Friday, October 28, 2022		[View]
Flow Chart with R numbers	2	Wednesday, January 22, 2020		[View]
Research Protocol Approved by UVic	3	Wednesday, January 22, 2020		[View]
School Lunchbox Checklist Protocol	1	Tuesday, January 14, 2020		[View]
A. Appendices List	2	Tuesday, September 10, 2019		[View]
SSHRC Funded proposal	1	Wednesday, June 13, 2018		[View]

9.2. Documentation of Consent	**Document Name**	**Version**	**Date**	**Password (if applicable)**	
	ATP interviews consent	1	Tuesday, March 4, 2025		[View]
	ATP interviews invitation	1	Tuesday, March 4, 2025		[View]
	RCT(Aim4_SubStudy)_Parent consents_v7.3	7.3	Wednesday, February 7, 2024		[View]
	RCT(Aim4)_ECE consent_v7.3	7.3	Wednesday, February 7, 2024		[View]
	RCT(Aim4_SubStudy)_Parent consents_v3	3	Wednesday, March 15, 2023		[View]
	RCT(Aim4)_ECE consent_v4	4	Wednesday, March 15, 2023		[View]
	RCT(Aim4)_invitation and consent form qualitaive_v1	2	Friday, October 28, 2022		[View]
	Time 3 EY Survey consent and invitation	2	Thursday, September 15, 2022		[View]
	Time 3 Consent and initial invitation CLEAN VERSION	1	Thursday, September 15, 2022		[View]
	Pilot ATP+ ECE consent	1	Wednesday, June 15, 2022		[View]
	Child Consent Years 1 and 2	8	Thursday, February 27, 2020		[View]
	ECE Consent	4	Thursday, February 20, 2020		[View]
	I. Manager_Recruitment Script, Consent, Survey Link_Years 1 and 3	4	Tuesday, September 10, 2019		[View]
	N. Short Program Staff Recruitment Script, Consent, Survey Link Year 1	4	Tuesday, September 10, 2019		[View]
	E. Delivery Agent_Consent_Years 1-4	4	Tuesday, September 10, 2019		[View]
	C. Stakeholder_Consent_Years 1-4	4	Tuesday, September 10, 2019		[View]
	M. Childcare Staff Recruitment Script, Consent, Survey Link Years 1 and 3	4	Tuesday, September 10, 2019		[View]
	K. Manager Recruitment Script, Consent, Survey Link Longitudinal Study	4	Tuesday, September 10, 2019		[View]
	S. SP Staff_Recruitment Script, Consent, Survey Link_Longitudinal Study_v4	4	Tuesday, September 10, 2019		[View]
	NNN. Delivery Agent Interview Consent Years 1-4	2	Tuesday, September 10, 2019		[View]
	Q. Childcare Staff Recruitment Script, Consent, Survey Link Longitudinal Study	4	Tuesday, September 10, 2019		[View]

	Document Name	Version	Date	Password (if applicable)	
	OOO. Virtual Workshop Consent	1	Wednesday, October 3, 2018		[View]
	LLL. Workshop Participant Interview Consent Form	1	Wednesday, January 31, 2018		[View]
	VV. Workshop Participant Consent Form v2 for ethics	2	Tuesday, December 5, 2017		[View]
	HHH. E-Module Consent	1	Tuesday, December 5, 2017		[View]
	I. Manager_Recruitment Script, Consent, Survey Link_Years 1 and 3_v3	3	Tuesday, March 7, 2017		[View]

9.3. Documentation of Assent

Document Name	Version	Date	Password (if applicable)	
Child Assent Years 1 and 2	2	Wednesday, January 15, 2020		[View]

9.4. Advertisement to Recruit Participants *(Ads, Posters, letters of initial contact, etc).*

Document Name	Version	Date	Password (if applicable)	
RCT(Aim4_SubStudy)_parenting_advertisement_v3	3	Wednesday, August 23, 2023		[View]
RCT(Aim4)_Advertisement to recruit_v1	1	Wednesday, January 11, 2023		[View]

9.5. Questionnaire, Questionnaire Consent Cover Letter, Tests, Interview Scripts, etc.

Document Name	Version	Date	Password (if applicable)	
ATP interview guide	1	Thursday, March 13, 2025		[View]
RCT(Aim4)_Interview script for parents_v3	3	Wednesday, August 23, 2023		[View]
RCT(SubStudy)_Parenting Measures_v4	4	Thursday, April 27, 2023		[View]
RCT(Aim4) Modified EPAO_v10	10	Thursday, April 27, 2023		[View]
RCT(SubStudy)_Parenting Measures_v2	2	Friday, October 28, 2022		[View]
RCT(Aim4)_Interview script for parents_v1	1	Friday, October 28, 2022		[View]
Good Start Matters Equipment Environment Space	3	Friday, October 28, 2022		[View]
Time 3 EY Survey	1	Thursday, September 15, 2022		[View]
Pilot ATP+ interview	1	Wednesday, June 15, 2022		[View]
Early Years survey invitation email - Observation	2	Thursday, March 5, 2020		[View]
Parent survey invitation email	2	Thursday, March 5, 2020		[View]
EY Surveys Centre collection	2	Thursday, February 20,		[View]

		2020	
Childcare Centre Screening Questions	1	Thursday, February 20, 2020	[View]
Parent Survey	1	Thursday, January 16, 2020	[View]
School Food Checklist	1	Wednesday, January 8, 2020	[View]
QQQ. Virtual Workshop Post-Survey	1	Thursday, October 3, 2019	[View]
RRR. EY Surveys_2018_19_ver_20_01172019	1	Thursday, January 17, 2019	[View]
PPP. Virtual Workshop Pre Survey	1	Wednesday, October 3, 2018	[View]
AA. Delivery Agent_Interview Schedule_Years 1-4_v2	2	Friday, March 9, 2018	[View]
GGG. E-Learning Module Post-PA Survey_v2	2	Wednesday, January 31, 2018	[View]
JJJ. EY Participant Pre-Workshop Survey_NonECE	1	Wednesday, January 31, 2018	[View]
KKK. EY Participant Post-Workshop Survey_NonECE	1	Wednesday, January 31, 2018	[View]
EEE. E-Learning Module Pre-PA Survey_V2	2	Wednesday, January 31, 2018	[View]
XX. Workshop Participant Interview Schedule_v3	3	Wednesday, January 31, 2018	[View]
FFF.E-Learning Module Post-HE Survey_v2	2	Wednesday, January 31, 2018	[View]
DDD. E-Learning Module Pre-HE Survey_v2	2	Wednesday, January 31, 2018	[View]
DD. Delivery Agent Tracking tools v2	2	Tuesday, December 5, 2017	[View]
PP. EY Participant Post-Workshop Survey_v3formatted	3	Tuesday, December 5, 2017	[View]
III. 2 page non-standard ATP Post-Workshop Survey	1	Tuesday, December 5, 2017	[View]
RR. EY Trainer Pre-Workshop Surveyv3formatted	3	Tuesday, December 5, 2017	[View]
TT. EY Trainer Post-Workshop Survey v3formatted	3	Tuesday, December 5, 2017	[View]
NN. EY Participant Pre-Workshop Survey_v3 formatted	3	Tuesday, December 5, 2017	[View]
CCC. Family Child Care Questionnaire v2 2017-05-15	2	Monday, May 15, 2017	[View]

	HH. Short Program Staff Questionnaire	1	Tuesday, March 7, 2017		[View]
	FF. Childcare Staff General Questionnaire_Word Format	1	Tuesday, March 7, 2017		[View]
	EE. Manager Questionnaire_Word Format	1	Tuesday, March 7, 2017		[View]
	GG. Childcare Staff Today Questionnaire_Word Format	1	Tuesday, March 7, 2017		[View]
	BB. Stakeholder_Interview Transcript Review Script_Years 1-4_v1	1	Thursday, May 12, 2016		[View]
	CC. Delivery Agent_Interview Transcript Review Script_Years 1-4_v1	1	Thursday, May 12, 2016		[View]
	Z. Stakeholder_Interview Schedule_Years 1-4_v1	1	Thursday, May 12, 2016		[View]

9.6. Letter of Initial Contact	**Document Name**	**Version**	**Date**	**Password (if applicable)**	
	External Email Invitation	1	Friday, December 20, 2024		[View]
	RCT(Aim4_SubStudy)_Initial Invitations_v5	5	Tuesday, February 7, 2023		[View]
	RCT(Aim4_SubStudy)_Initial Invitations_v1	1	Friday, October 28, 2022		[View]
	Pilot ATP+ invitation	1	Wednesday, June 15, 2022		[View]
	Manager Multi Site Invitation Letter	3	Thursday, February 20, 2020		[View]
	Single Site Invitation Letter	3	Thursday, February 20, 2020		[View]
	Child Recruitment Script	3	Wednesday, January 22, 2020		[View]
	KK. Childcare Staff Sub-Study letter of information	2	Tuesday, July 31, 2018		[View]
	OOO. Staff and Manager Interview Recruitment	1	Tuesday, July 31, 2018		[View]
	SSS. Manager Recruitment Postcard	1	Tuesday, July 31, 2018		[View]
	PPP. Child Care Centre Sub-Study Invitational package	1	Tuesday, July 31, 2018		[View]
	H. Manager Initial Recruitment Script Years 1 and 3 v2	2	Tuesday, July 31, 2018		[View]
	MMM. Delivery Agent Interview Invitation	1	Friday, March 9, 2018		[View]
	ZZ. Sample promotional scripts for Family Child Care facilities	2	Tuesday, April 11, 2017		[View]
	AAA. Family Child Care Recruitment Script and Survey Link V2	2	Tuesday, April 11, 2017		[View]
	YY. Direct mail out letter to Group facilities (w paper survey April 2017)	1	Tuesday, April 11, 2017		[View]
	D. Delivery Agent_Recruitment Script_Years 1-4_v1	1	Thursday, May 12, 2016		[View]
	B. Stakeholder_Recruitment Script_Years 1-4_v1	1	Thursday, May 12, 2016		[View]
	X. Child_Recruitment Script_Spring Years 1 and 2_v2	2	Tuesday, May 10, 2016		[View]

	Y. Child_Recruitment Script_Fall Year 2_v2	2	Tuesday, May 3, 2016		[View]
9.7. Other Documents	**Document Name**	**Version**	**Date**	**Password (if applicable)**	
	RCT(Aim4)_REDCap_v4	4	Friday, December 20, 2024		[View]
	RCT(Aim4)_REDCap_v3	3	Wednesday, November 22, 2023		[View]
	RCT(Aim4)_Brochure_ATP_v1	1	Wednesday, August 23, 2023		[View]
	RCT(Aim4_SubStudy)_Parenting communications_v4	4	Wednesday, August 23, 2023		[View]
	RCT(Aim4_SubStudy)_Parenting Pathverse intervention_v2	2	Thursday, April 27, 2023		[View]
	ATP+ communications_v2	2	Tuesday, February 7, 2023		[View]
	RCT(Aim4)_Interview for parents_other documents_v2	1	Friday, October 28, 2022		[View]
	RCT(SubStudy)_Parenting Intervention_v1	1	Friday, October 28, 2022		[View]
	RCT(Aim4_SubStudy)_ATP+ Presentation_v1	1	Friday, October 28, 2022		[View]
	RCT(Aim4_SubStudy)_Other Docs_v2	2	Friday, October 28, 2022		[View]
	RCT(Aim4_SubStudy)_ATP+ Presentation_v1	1	Friday, October 28, 2022		[View]
	Pathverse Privacy Policy	1	Friday, October 28, 2022		[View]
	RCT(Aim4) Safe Research Plan_v1	1	Friday, October 28, 2022		[View]
	Time 3 EY Survey other documents		Thursday, September 15, 2022		[View]
	ATP+ intervention	1	Wednesday, June 15, 2022		[View]
	Pilot ATP+ other documents	1	Wednesday, June 15, 2022		[View]
	Family incentive email	1	Thursday, February 27, 2020		[View]
	Educator incentive email	1	Thursday, February 27, 2020		[View]
	Educators missing item reminder scripts - email and phone	1	Thursday, February 27, 2020		[View]
	Parents missing item reminder scripts - email and phone	1	Thursday, February 27, 2020		[View]
	Multi Site Follow Up Call Script	2	Wednesday, January 22, 2020		[View]
	Single Site Follow Up Call Script	2	Wednesday, January 22, 2020		[View]
	Multi Site Obtaining Contact info call script	1	Friday, January 10, 2020		[View]

	Schedule Meeting Call script	1	Friday, January 10, 2020	[View]
	TTT. Invitation Multi-Age Facilities_01172019	1	Thursday, January 17, 2019	[View]
	UUU. Invitation retake_01172019	1	Thursday, January 17, 2019	[View]
	J. Manager Reminder Script Years 1 and 3	3	Friday, December 7, 2018	[View]
	BBB. Family Child Care Reminder Script_Years 1 and 3	1	Tuesday, April 11, 2017	[View]
	R. Childcare Staff_Reminder Script_Longitudinal Study_v3	3	Tuesday, March 7, 2017	[View]
	P. SP Staff_Reminder Script_Year 1_v3	3	Tuesday, March 7, 2017	[View]
	LL. Phone call reminder script	1	Tuesday, March 7, 2017	[View]
	L. Manager_Reminder Script_Longitudinal Study_v3 DO NOT USE	3	Tuesday, March 7, 2017	[View]
	MM. EY phone call followup email script	1	Tuesday, March 7, 2017	[View]
	O. Childcare Staff_Reminder Script_Years 1 and 3_v3	3	Tuesday, March 7, 2017	[View]
	T. SP Staff_Reminder Script_Longitudinal Study_v3	3	Tuesday, March 7, 2017	[View]
	F. Stakeholder_Reminder Script_Years 1-4_v2	2	Thursday, May 12, 2016	[View]
	JJ. TGMD-3 Skill Scoring Sheet	1	Thursday, May 12, 2016	[View]
	G. Delivery Agent_Reminder Script_Years 1-4_v2	2	Tuesday, May 3, 2016	[View]
9.8. Websites and Social Media				

10. Fee for Service - Behavioural Study [View Form]

How to submit<br><br>*Please indicate which of the following methods of payment will be used for this application:*	
Please wait for the invoice from the UBC Behavioural Research Ethics Board (BREB) to submit payment. The invoice will detail payment instructions and wire transfer information.<br><br>***Contact information regarding where to send the invoice.***	

12. Save Application - Human Ethics [View Form]

Print Close
