# Supplementary material for: Facility-level implementation strategies in early childhood education and care to enhance adherence to a provincial physical activity standard: Protocol for the Good Start Matters ATP+ randomised controlled trial
Source: PLoS One. 2025 Aug 12;20(8):e0329276. doi: 10.1371/journal.pone.0329276 (PMC12342287; doi:10.1371/journal.pone.0329276)
Supplement: S2 File — (PDF) [file pone.0329276.s002.pdf]

Certificate of Ethical Approval for Harmonized Minimal Risk Behavioural Study

UBC Behavioural Research Ethics Board

The University of British Columbia
Behavioural Research Ethics Board
#102, 6190 Agronomy Road
Vancouver, BC V6T 1Z3

Also reviewed and approved by:

- **University of Victoria**

**Principal Investigator:**	**Primary Appointment:**	**Board of Record REB Number:**	**UBC REB Number:**
Louise C. Masse	UBC/Medicine, Faculty of/School of Population and Public Health	**Board of Record:** UBC Behavioural Research Ethics Board	H18-01434

Study Title:
Early Years Capacity-Building Evaluation

Study Approved: September 11, 2019 **Expiry Date: September 11, 2020**

**Research Team Members:**	Katie Weatherson<br>Viviene A Temple<br>Heather A. McKay<br>Yuen Yan Lau<br>Luke Wolfenden<br>Patti-Jean Naylor<br>Jennifer McConnell-Nzunga<br>Erica Y Lau
**Sponsoring Agencies:**	- Canadian Institutes of Health Research (CIHR) - "A good start matters: Do supportive childcare centre environments, policies and practices enhance physical activity?"

Documents included in this approval:

Document Name	Version	Date
**Protocol:**		
SSHRC Funded proposal	1	June 13, 2018
CIHR funded grant	1	June 13, 2018
A. Appendices List	2	September 10, 2019
Research Protocol Approved by UVic	1	August 8, 2019
**Consent Forms:**		
M. Childcare Staff Recruitment Script, Consent, Survey Link Years 1 and 3	4	September 10, 2019
LLL. Workshop Participant Interview Consent Form	1	January 31, 2018
NNN. Delivery Agent Interview Consent Years 1-4	2	September 10, 2019
I. Manager_Recruitment Script, Consent, Survey Link_Years 1 and 3	4	September 10, 2019
HHH. E-Module Consent	1	December 5, 2017
S. SP Staff_Recruitment Script, Consent, Survey	4	September

	Link_Longitudinal Study_v4		10, 2019
	Q. Childcare Staff Recruitment Script, Consent, Survey Link Longitudinal Study	4	September 10, 2019
	OOO. Virtual Workshop Consent	1	October 3, 2018
	N. Short Program Staff Recruitment Script, Consent, Survey Link Year 1	4	September 10, 2019
	I. Manager_Recruitment Script, Consent, Survey Link_Years 1 and 3_v3	3	March 7, 2017
	E. Delivery Agent_Consent_Years 1-4	4	September 10, 2019
	C. Stakeholder_Consent_Years 1-4	4	September 10, 2019
	V. Child Consent Years 1 and 2	4	September 10, 2019
	VV. Workshop Participant Consent Form v2 for ethics	2	December 5, 2017
	K. Manager Recruitment Script, Consent, Survey Link Longitudinal Study	4	September 10, 2019
	**Assent Forms:**		
	W. Child_Verbal Assent Script_Years 1 and 2_v1	1	May 12, 2016
	**Questionnaire, Questionnaire Cover Letter, Tests:**		
	BB. Stakeholder_Interview Transcript Review Script_Years 1-4_v1	1	May 12, 2016
	RR. EY Trainer Pre-Workshop Surveyv3formatted	3	December 5, 2017
	DDD. E-Learning Module Pre-HE Survey_v2	2	January 31, 2018
	XX. Workshop Participant Interview Schedule_v3	3	January 31, 2018
	CC. Delivery Agent_Interview Transcript Review Script_Years 1-4_v1	1	May 12, 2016
	KKK. EY Participant Post-Workshop Survey_NonECE	1	January 31, 2018
	PPP. Virtual Workshop Pre Survey	1	October 3, 2018
	EE. Manager Questionnaire_Word Format	1	March 7, 2017
	PP. EY Participant Post-Workshop Survey_v3formatted	3	December 5, 2017
	QQQ. Virtual Workshop Post-Survey	1	October 3, 2019
	EEE. E-Learning Module Pre-PA Survey_V2	2	January 31, 2018
	GG. Childcare Staff Today Questionnaire_Word Format	1	March 7, 2017
	HH. Short Program Staff Questionnaire	1	March 7, 2017
	DD. Delivery Agent Tracking tools v2	2	December 5, 2017
	CCC. Family Child Care Questionnaire v2 2017-05-15	2	May 15, 2017
	AA. Delivery Agent_Interview Schedule_Years 1-4_v2	2	March 9, 2018
	II. Childcare Facilty Observation Tool	1	May 12, 2016
	Z. Stakeholder_Interview Schedule_Years 1-4_v1	1	May 12, 2016
	GGG. E-Learning Module Post-PA Survey_v2	2	January 31, 2018

	III. 2 page non-standard ATP Post-Workshop Survey	1	December 5, 2017
	FFF.E-Learning Module Post-HE Survey_v2	2	January 31, 2018
	NN. EY Participant Pre-Workshop Survey_v3 formatted	3	December 5, 2017
	JJJ. EY Participant Pre-Workshop Survey_NonECE	1	January 31, 2018
	FF. Childcare Staff General Questionnaire_Word Format	1	March 7, 2017
	RRR. EY Surveys_2018_19_ver_20_01172019	1	January 17, 2019
	TT. EY Trainer Post-Workshop Survey v3formatted	3	December 5, 2017
	**Letter of Initial Contact:**		
	Y. Child_Recruitment Script_Fall Year 2_v2	2	May 3, 2016
	SSS. Manager Recruitment Postcard	1	July 31, 2018
	H. Manager Initial Recruitment Script Years 1 and 3 v2	2	July 31, 2018
	U. Child Recruitment Script Fall Year 1	2	July 31, 2018
	OOO. Staff and Manager Interview Recruitment	1	July 31, 2018
	PPP. Child Care Centre Sub-Study Invitational package	1	July 31, 2018
	D. Delivery Agent_Recruitment Script_Years 1-4_v1	1	May 12, 2016
	MMM. Delivery Agent Interview Invitation	1	March 9, 2018
	YY. Direct mail out letter to Group facilities (w paper survey April 2017)	1	April 11, 2017
	X. Child_Recruitment Script_Spring Years 1 and 2_v2	2	May 10, 2016
	B. Stakeholder_Recruitment Script_Years 1-4_v1	1	May 12, 2016
	AAA. Family Child Care Recruitment Script and Survey Link V2	2	April 11, 2017
	ZZ. Sample promotional scripts for Family Child Care facilities	2	April 11, 2017
	KK. Childcare Staff Sub-Study letter of information	2	July 31, 2018
	**Other Documents:**		
	BBB. Family Child Care Reminder Script_Years 1 and 3	1	April 11, 2017
	R. Childcare Staff_Reminder Script_Longitudinal Study_v3	3	March 7, 2017
	LL. Phone call reminder script	1	March 7, 2017
	T. SP Staff_Reminder Script_Longitudinal Study_v3	3	March 7, 2017
	P. SP Staff_Reminder Script_Year 1_v3	3	March 7, 2017
	TTT. Invitation Multi-Age Facilities_01172019	1	January 17, 2019
	L. Manager_Reminder Script_Longitudinal Study_v3 DO NOT USE	3	March 7, 2017
	UUU. Invitation retake_01172019	1	January 17, 2019
	O. Childcare Staff_Reminder Script_Years 1 and 3_v3	3	March 7, 2017
	J. Manager Reminder Script Years 1 and 3	3	December 7, 2018
	JJ. TGMD-3 Skill Scoring Sheet	1	May 12, 2016

	F. Stakeholder_Reminder Script_Years 1-4_v2	2	May 12, 2016
	MM. EY phone call followup email script	1	March 7, 2017
	G. Delivery Agent_Reminder Script_Years 1-4_v2	2	May 3, 2016

This ethics approval applies to research ethics issues only and does not include provision for any administrative approvals required from individual institutions before research activities can commence.

The Board of Record (as noted above) has reviewed and approved this study in accordance with the requirements of the Tri-Council Policy Statement: Ethical Conduct for Research Involving Humans (TCPS2, 2014).

The "Board of Record" is the Research Ethics Board delegated by the participating REBs involved in a harmonized study to facilitate the ethics review and approval process.

The application for ethical review and the document(s) listed above have been reviewed and the procedures were found to be acceptable on ethical grounds for research involving human subjects.

This study has been approved either by the Board of Record's full REB or by an authorized delegated reviewer.

UBC Behavioural Research Ethics Board

The University of British Columbia
Behavioural Research Ethics Board
#102, 6190 Agronomy Road
Vancouver, BC V6T 1Z3

Certificate of Ethical Approval: Renewal for Harmonized Minimal Risk Behavioural Study

Also reviewed and approved by:

- **University of Victoria**

Principal Investigator:	Primary Appointment:	Board of Record REB Number:	REB Number:
Louise C. Masse	UBC/Medicine, Faculty of/School of Population and Public Health		H18-01434

**Study Title:** Early Years Capacity-Building Evaluation	
**Approval Date: July 27, 2020**	**Expiry Date: July 27, 2021**
**Research Team Members:**	Katie Weatherson<br>Viviene A Temple<br>Heather A. McKay<br>Luke Wolfenden<br>Patti-Jean Naylor<br>Jennifer McConnell-Nzunga<br>Erica Y Lau
**Sponsoring Agencies:**	- Canadian Institutes of Health Research (CIHR) - "A good start matters: Do supportive childcare centre environments, policies and practices enhance physical activity?"<br>- Canadian Institutes of Health Research (CIHR) - "Evaluating the impact of a province-wide capacity building intervention on nutrition policies and practices in early years providers "<br>- Social Sciences and Humanities Research Council of Canada (SSHRC) - "A Good Start Matters: Do provincial standards and capacity-building efforts influence childcare centre environments, policies and practices related to physical literacy in the early years?"
**Documents included in this approval:**	N/A

This ethics approval applies to research ethics issues only and does not include provision for any administrative approvals required from individual institutions before research activities can commence.

The Board of Record (as noted above) has reviewed and approved this study in accordance with the requirements of the Tri-Council Policy Statement: Ethical Conduct for Research Involving Humans (TCPS2, 2014).

The "Board of Record" is the Research Ethics Board delegated by the participating REBs involved in a harmonized study to facilitate the ethics review and approval process.

The application for ethical review and the document(s) listed above have been reviewed and the procedures were found to be acceptable on ethical grounds for research involving human subjects.

This study has been approved either by the Board of Record's full REB or by an authorized delegated reviewer.

UBC Behavioural Research Ethics Board

Certificate of Ethical Approval: Renewal for Harmonized Minimal Risk Behavioural Study

The University of British Columbia
Behavioural Research Ethics Board
#102, 6190 Agronomy Road
Vancouver, BC V6T 1Z3

Also reviewed and approved by:

- **University of Victoria**

Principal Investigator:	Primary Appointment:	Board of Record REB Number:	REB Number:
Louise C. Masse	UBC/Medicine, Faculty of/School of Population and Public Health		H18-01434

**Study Title:** Early Years Capacity-Building Evaluation	
**Approval Date: July 26, 2021**	**Expiry Date: July 26, 2022**
**Research Team Members:**	Katie Weatherson<br>Viviene A Temple<br>Heather A. McKay<br>Luke Wolfenden<br>Patti-Jean Naylor<br>Jennifer McConnell-Nzunga<br>Erica Y Lau
**Sponsoring Agencies:**	- Canadian Institutes of Health Research (CIHR) - "A good start matters: Do supportive childcare centre environments, policies and practices enhance physical activity?"<br>- Canadian Institutes of Health Research (CIHR) - "Evaluating the impact of a province-wide capacity building intervention on nutrition policies and practices in early years providers "<br>- Social Sciences and Humanities Research Council of Canada (SSHRC) - "A Good Start Matters: Do provincial standards and capacity-building efforts influence childcare centre environments, policies and practices related to physical literacy in the early years?"
**Documents included in this approval:**	N/A

This ethics approval applies to research ethics issues only and does not include provision for any administrative approvals required from individual institutions before research activities can commence.

The Board of Record (as noted above) has reviewed and approved this study in accordance with the requirements of the Tri-Council Policy Statement: Ethical Conduct for Research Involving Humans (TCPS2, 2018).

The "Board of Record" is the Research Ethics Board delegated by the participating REBs involved in a harmonized study to facilitate the ethics review and approval process.

The application for ethical review and the document(s) listed above have been reviewed and the procedures were found to be acceptable on ethical grounds for research involving human subjects.

This study has been approved either by the Board of Record's full REB or by an authorized delegated reviewer.

REBC

Certificate of Ethical Approval: Renewal for Harmonized Minimal Risk Behavioural Study

UBC Behavioural Research Ethics Board

The University of British Columbia
Behavioural Research Ethics Board
#102, 6190 Agronomy Road
Vancouver, BC V6T 1Z3

Also reviewed and approved by:

- **University of Victoria**

**Principal Investigator:**	**Primary Appointment:**	**Board of Record REB Number:**	**REB Number: H18-01434**
Louise C. Masse	UBC/Medicine, Faculty of/School of Population and Public Health		PAA #: H18-01434-A013

**Study Title:**<br>Early Years Capacity-Building Evaluation	
**Approval Date: June 15, 2022**	**Expiry Date: June 15, 2023**
**Research Team Members:**	Katie Weatherson<br>Mariana Brussoni<br>Viviene A Temple<br>Valerie Carson<br>Heather A. McKay<br>Luke Wolfenden<br>Guy Faulkner<br>Patti-Jean Naylor<br>Jennifer McConnell-Nzunga<br>Erica Y Lau
**Sponsoring Agencies:**	- Canadian Institutes of Health Research (CIHR) - "A good start matters: Do supportive childcare centre environments, policies and practices enhance physical activity?"<br>- Canadian Institutes of Health Research (CIHR) - "Evaluating the impact of a province-wide capacity building intervention on nutrition policies and practices in early years providers "<br>- Social Sciences and Humanities Research Council of Canada (SSHRC) - "A Good Start Matters: Do provincial standards and capacity-building efforts influence childcare centre environments, policies and practices related to physical literacy in the early years?"
**Documents included in this approval:**	N/A

This ethics approval applies to research ethics issues only and does not include provision for any administrative approvals required from individual institutions before research activities can commence.

The Board of Record (as noted above) has reviewed and approved this study in accordance with the requirements of the Tri-Council Policy Statement: Ethical Conduct for Research Involving Humans (TCPS2, 2018).

The "Board of Record" is the Research Ethics Board delegated by the participating REBs involved in a harmonized study to facilitate the ethics review and approval process.

The application for ethical review and the document(s) listed above have been reviewed and the

procedures were found to be acceptable on ethical grounds for research involving human subjects.
**This study has been approved either by the Board of Record's full REB or by an authorized delegated reviewer.**

REBC

UBC Behavioural Research Ethics Board

The University of British Columbia
Behavioural Research Ethics Board
#102, 6190 Agronomy Road
Vancouver, BC V6T 1Z3

Certificate of Ethical Approval: Renewal for Harmonized Minimal Risk Behavioural Study

Also reviewed and approved by:

- **University of Victoria**

**Principal Investigator:**	**Primary Appointment:**	**Board of Record REB Number:**	**REB Number: H18-01434**
Louise C. Masse	UBC/Medicine, Faculty of/School of Population and Public Health		PAA #: H18-01434-A020

**Study Title:**<br>Good Start Matters	
**Approval Date: June 8, 2023**	**Expiry Date: June 8, 2024**
**Research Team Members:**	Katie Weatherson<br>Mariana Brussoni<br>Viviene A Temple<br>Valerie Carson<br>Heather A. McKay<br>Luke Wolfenden<br>Guy Faulkner<br>Patti-Jean Naylor<br>Jennifer McConnell-Nzunga<br>Erica Y Lau
**Sponsoring Agencies:**	- Canadian Institutes of Health Research (CIHR) - "A good start matters: Do supportive childcare centre environments, policies and practices enhance physical activity?"<br>- Canadian Institutes of Health Research (CIHR) - "Evaluating the impact of a province-wide capacity building intervention on nutrition policies and practices in early years providers "<br>- Michael Smith Health Research BC - "Unraveling the complexity of familial influences that shape children's obesity-related behaviours over time"<br>- Social Sciences and Humanities Research Council of Canada (SSHRC) - "A Good Start Matters: Do provincial standards and capacity-building efforts influence childcare centre environments, policies and practices related to physical literacy in the early years?"
**Documents included in this approval:**	N/A

This ethics approval applies to research ethics issues only and does not include provision for any administrative approvals required from individual institutions before research activities can commence.

The Board of Record (as noted above) has reviewed and approved this study in accordance with the requirements of the Tri-Council Policy Statement: Ethical Conduct for Research Involving Humans (TCPS2, 2018).

The "Board of Record" is the Research Ethics Board delegated by the participating REBs involved in a

harmonized study to facilitate the ethics review and approval process.
The application for ethical review and the document(s) listed above have been reviewed and the procedures were found to be acceptable on ethical grounds for research involving human subjects.
**This study has been approved either by the Board of Record's full REB or by an authorized delegated reviewer.**

UBC Behavioural Research Ethics Board

Certificate of Ethical Approval: Renewal for Harmonized Minimal Risk Behavioural Study

The University of British Columbia
Behavioural Research Ethics Board
#102, 6190 Agronomy Road
Vancouver, BC V6T 1Z3

Also reviewed and approved by:

- **University of Victoria**

**Principal Investigator:**	**Primary Appointment:**	**Board of Record REB Number:**	**REB Number:** **H18-01434**
Louise C. Masse	UBC/Medicine, Faculty of/School of Population and Public Health		PAA #: H18-01434-A031

**Study Title:**<br>Good Start Matters	
**Approval Date: May 15, 2024**	**Expiry Date: May 15, 2025**
**Research Team Members:**	Katie Weatherson<br>Mariana Brussoni<br>Viviene A Temple<br>Valerie Carson<br>Heather A. McKay<br>Jean Buckler<br>Luke Wolfenden<br>Guy Faulkner<br>Patti-Jean Naylor<br>Jennifer McConnell-Nzunga<br>Erica Y Lau
**Sponsoring Agencies:**	- Canadian Institutes of Health Research (CIHR) - "A good start matters: Do supportive childcare centre environments, policies and practices enhance physical activity?"<br>- Canadian Institutes of Health Research (CIHR) - "Evaluating the impact of a province-wide capacity building intervention on nutrition policies and practices in early years providers "<br>- Michael Smith Health Research BC - "Unraveling the complexity of familial influences that shape children's obesity-related behaviours over time"<br>- Social Sciences and Humanities Research Council of Canada (SSHRC) - "A Good Start Matters: Do provincial standards and capacity-building efforts influence childcare centre environments, policies and practices related to physical literacy in the early years?"
**Documents included in this approval:**	N/A
**Document(s) acknowledged with this submission:**	<u>**Summary Report(s)**</u><br>There are no items to display

This ethics approval applies to research ethics issues only and does not include provision for any administrative approvals required from individual institutions before research activities can commence.

The Board of Record (as noted above) has reviewed and approved this study in accordance with the most recent requirements of the Tri-Council Policy Statement: Ethical Conduct for Research Involving

Humans (TCPS2).<br><br>The "Board of Record" is the Research Ethics Board delegated by the participating REBs involved in a harmonized study to facilitate the ethics review and approval process.
The application for ethical review and the document(s) listed above have been reviewed and the procedures were found to be acceptable on ethical grounds for research involving human subjects.
**This study has been approved either by the Board of Record’s full REB or by an authorized delegated reviewer.**

Date: 5/5/2025, 2:20:14 PM | Print | Close

undefinedundefined

The University of British Columbia
Office of Research Ethics
Behavioural Research Ethics Board
Suite 102, 6190 Agronomy Road
Vancouver, BC V6T 1Z3

H18-01434 Child Care (Version 30.0)

Principal Investigator: Louise C. Masse

1. Principal Investigator & Study Team - Human Ethics [View Form]

1.1. Principal Investigator	**Last Name**	**First Name**	**Employer.Name**	**Email**
	Masse	Louise C.	School of Population and Public Health	lmasse@bcchr.ubc.ca

Enter Principal Investigator's secondary appointments or affiliations (including Health Authorities), if applicable:	School of Population and Public Health

1.2. Primary Contact	**Last Name**	**First Name**	**Rank**
	de Jongh Gonzalez	Olivia	Post Doctoral Fellow

1.3A. Co-Investigators - Online Access

Last Name	First Name	Institution/Department	Rank	TCPS2
Weatherson	Katie	UBC/UBCO - Faculty of Health and Social Development/UBCO - School of Health and Exercise Science	M&P Staff	yes
Brussoni	Mariana	UBC/Medicine, Faculty of/Paediatrics	Professor	yes
Temple	Viviene A	University of Victoria	Non-UBC Faculty Member	
Carson	Valerie	Other/Other University/Hospital	Non-UBC Faculty Member	
McKay	Heather A.	UBC/Medicine, Faculty of/Family Practice	Professor	yes
Buckler	Jean	University of Victoria	Assistant Professor	yes
Wolfenden	Luke	Other/Other University/Hospital	Non-UBC Faculty Member	
Faulkner	Guy	UBC/Education/School of Kinesiology	Professor	yes
Naylor	Patti-Jean	University of Victoria	Professor	yes
McConnell-Nzunga	Jennifer	UBC/Medicine, Faculty of/School of Population and Public Health	Post Doctoral Fellow	yes
Lau	Erica Y	Other	Unspecified	yes

1.3B. Describe each Co-I's role in study, e.g. statistician, supervisor, adviser, student etc.

Ensure individual is entered in Box 1.3A	
1.4A. Additional Study Team Members - Online Access	**Last Name** / **First Name** / **Institution/Department** / **Rank** / **TCPS2** (see table below)
1.4B. Describe each Additional Study Team Members' role in study, e.g. staff, research assistant etc.	
1.5A. Additional Study Team Members - No Online Access	(see table below)
1.5B. Describe each Additional Study Team Members' (no online access) role in study, e.g. external supervisor, consultant etc.	(PAA April 17, 2024) Four new research staff will start working on this project, and all of them will be involved in recruitment and data collection for centers, educators and parents:<br>1) Nicole Armeanu, TCPS2 certificate obtained on 7-10-2022<br>2) Natalie Shek, TCPS2 certificate obtained on 8-10-2022<br>3) Courtney Hall, TCPS2 certificate obtained on 29-9-2023<br>4) Emma Graydon, TCPS2 certificate obtained on 2690-2023<br><br>April 30, 2024 Staff who no longer worked on the project were deleted from the application

1.4A. Additional Study Team Members - Online Access

Last Name	First Name	Institution/Department	Rank	TCPS2
Leon Elizalde	Angelica	Other	Unspecified	yes
Smith	Kate	UBC/Science	Undergraduate Student	yes
Scarr	Jennifer	Other/Other University/Hospital	Non-UBC Employee	
Wright	Chris	University of Victoria	Non-UBC Faculty Member	yes
Marshall-beaucoup	Sophie	UBC/Medicine, Faculty of/School of Population and Public Health	Graduate Student	yes
Sauve	Karen	UBC/Medicine, Faculty of/Physical Therapy	Assistant Professor of Teaching	yes
de Jongh Gonzalez	Olivia	UBC/Medicine, Faculty of/School of Population and Public Health	Post Doctoral Fellow	yes
Pitblado	Mark kenneth, john	UBC/Science	M&P Staff	yes
Edache	Iyoma	UBC/Medicine, Faculty of	Graduate Student	yes
Hassani	Kasra	UBC/Medicine, Faculty of/BC Children’s Hospital Research Institute	Non-UBC Employee	yes
Deslippe	Alysha	UBC/Land and Food Systems	Graduate Student	yes
Tugault-Lafleur	Claire	Other	Unspecified	
Li	Joy	UBC/Land and Food Systems	Undergraduate Student	yes
Dong	Amy	UBC/Medicine, Faculty of/School of Population and Public Health	Graduate Student	yes
Villagomez Volkers	Paula	UBC/Medicine, Faculty of/School of Population and Public Health	Graduate Student	yes

1.5A. Additional Study Team Members - No Online Access

Last Name	First Name	Institution / Department	Rank / Job Title	Email Address
Nieuwenhuijs	Eva	UBC	research staff	evaspph@student.ubc.ca
Alexander	Megan	University of Victoria	research staff	
Graydon	Emma	University of Victoria	research staff	emmagraydon@uvic.ca
Hall	Courtney	University of Victoria	research staff	chall625@uvic.ca
Shek	Natalie	UBC	research staff	natalieshekjh@gmail.com
Armeanu	Nicole	UBC	research staff	narmeanu@student.ubc.ca
Grant	Grace	University of Victoria	research staff	

	as requested by REB. Names are included here as these staff/students were on publications for this study which required that they be listed on the REB certificate (Karen Strange, Camilla Briggs, Kristin McIlhenney, Kayla Morton, Sana Fakih)<br><br>(PAA May 17, 2024)<br>Two new students have joined our team and both of them will be supporting recruitment and data collection for this project:<br>• Grace Grant, University of Victoria, Completed TCPS2 on 17 May, 2024<br>• Megan Alexander, University of Victoria, Completed TCPS2 on 25 April, 2024<br><br>PAA July 31, 2024: A new student, Eva Nieuwenhuijs, is joining our team and will support recruitment and data collection for this project. Eva completed the TCPS2 on July 17, 2024.
1.6. Tri Council Policy Statement (TCPS) Tutorial<br><br>*Have all research personnel completed the required TCPS2 tutorial:*	Yes
1.7. Project Title<br><br>*Enter the title of this research study as it will appear on the certificate. Title given **must match** the title on all study documents.*	Good Start Matters
1.8. Project Nickname<br><br>*Enter a nickname for this study. What would you like this study to be known as to the Principal Investigator and study team?*	Child Care
**2. Study Dates and Funding - Human Ethics** [View Form]	
You plan to start collecting data immediately after obtaining ethics and any other required approvals	no
You plan to start data collection at a later date i.e., 2 months or more after approvals are obtained. Click the calendar icon below to select the dates.<br><br>*Estimated start date:*	9/11/2019
2.1.B.<br><br>*Estimated end date:*	3/31/2025
2.2.A. Types of Funds<br><br>*Please select the applicable box(es) below to indicate the type(s) of*	Grant

funding you are receiving to conduct this research. ***You must then complete section 2.3 and/or section 2.4 for the name of the source of the funds to be listed on the certificate of approval.***	
2.2.B. *For Industry Sponsored studies, please provide a sponsor contact.*	
2.3.A. *Research Funding Application/Award Associated with the Study that was Submitted to the UBC Office of Research Ethics*	**UBC Number** / **Title** / **Sponsor**<br>F18-04316 / Evaluating the impact of a province-wide capacity building intervention on nutrition policies and practices in early years providers / Canadian Institutes of Health Research (CIHR)<br>F16-04356 / A good start matters: Do supportive childcare centre environments, policies and practices enhance physical activity? / Canadian Institutes of Health Research (CIHR)<br>F19-03402 / A Good Start Matters: Do provincial standards and capacity-building efforts influence childcare centre environments, policies and practices related to physical literacy in the early years? / Social Sciences and Humanities Research Council of Canada (SSHRC)<br>F22-01244 / Unraveling the complexity of familial influences that shape children's obesity-related behaviours over time / Michael Smith Health Research BC
2.3.B. *Which institution is administering the funds, if not UBC or UBC affiliated institution?*	
2.4.A. *Research Funding Application/Award Associated with the Study not listed in question 2.3.*	**UBC Number** **Title** **Sponsor**
2.4.B. *Please enter any applicable information about your funding which is not already shown in Box 2.3A or 2.4A (including funding applied for but not yet received).*	SSHRC funding was also received but the funds are allocated to Dr. Patti Jean Naylor from the University of Victoria.
2.5.A. *Is this a DHHS grant?*	no
2.5.B. *Please select the appropriate DHHS funding agency from the selection box.*	**DHHS Sponsor List:** **Order:** **Active:**
2.6. Study Related Conflict of Interest<br><br>*Conflicts of Interest (COIs) in research are situations where someone's personal interests (financial,*	no

career, or other) could compromise or could be perceived to compromise the objective conduct of research or integrity of the data. Conflicts of interest can arise naturally from an Investigator's engagement inside and outside the University, and the mere existence of a COI or the perception of a COI does not necessarily imply wrongdoing on anyone's part. Nonetheless, real and perceived COI must be recognized, disclosed, and assessed. This question asks Investigators to disclose COIs that may relate to the research study that is the subject of the REB application.<br><br>*Do the Principal Investigator, Co-Investigators and/or their related parties have any personal interest(s) that could compromise or reasonably be perceived to compromise the objective conduct of the research or the integrity of the data generated by the study? Personal interests may include business, commercial or financial interests, dual roles (e.g. PI and Doctor), as well as personal matters and career interests.*	
**4.A. Study Type - (Boxes 4.1 to 4.2C)** [View Form]	
4.1. Application Type<br><br>*Indicate whether your application is Clinical or Behavioural.*	Behavioural
***4.2.A.** Institutions and Sites for Study (including study team members' institutional affiliations under which this research is being conducted)*	**Institution** / **Site**<br>UBC / Vancouver (excludes UBC Hospital)
***4.2.B.** Non-UBC Institutions and Sites for Study (including study*	**Institution** / **Site**<br>University of Victoria / Main Campus

team members' institutional affiliations under which this research is being conducted)	
4.2.C. *Please enter any other locations where the research will be conducted under this Research Ethics Approval (e.g., Name of privately owned clinic, community centre, school, classroom, participant's home, in the field - provide details).*	The study will take place in childcare facilities throughout British Columbia. Since this is a behavioural study, REB approval was sought from UBC BREB.

4.B. Behavioural Study Type - (Boxes 4.2D to 4.6) [View Form]

4.2.D. Roles of Study Sites and Institutions

Study Site:	Accessing Records or Charts:	Analysing Data or Utilizing Lab Space:	Recruiting Participants:	Team Member Affiliations:
UBC - Vancouver (excludes UBC Hospital)	no	yes	yes	yes
University of Victoria - Main Campus	no	no	yes	yes

4.3.A. *If this proposal is closely linked to any other proposal previously/simultaneously submitted, enter the Institution or Health Authority name and associated Research Ethics Board study number of that proposal.*<br><br>*Institution Name:*	University of Victoria
REB study number:	BC16-128
4.3.B. *If applicable, please describe the relationship between this proposal and the previously/simultaneously submitted proposal listed above.*	The harmonized ethics application (BC16-128) was previously approved by University of Victoria REB. Currently, the PI from UBC (L. Masse) is taking the lead on the study for the data collection of groups 3, 4, and 6, thus, REB approval is sought from BREB. Given that the bulk of the funds is held at UBC and that the original application was too complex, I am requesting that the board of record for the component of this study be transferred to UBC.
4.3.C. *Have you received any information or are you aware of any rejection of this study by any Research Ethics Board? If yes, please provide known details and attach any available relevant documentation in Box 9.7.*	no
Please provide known details:	

4.4.A. *External peer review details:*	SSHRC<br>CIHR
4.4.B. *Internal (Institution or hospital) peer review details:*	N/A
4.4.C. *If this research proposal has not received any independent scientific/methodological peer review, explain why no review has taken place.*	N/A
Participant Vulnerability	Medium
Research Risk	Low
4.5.B. *Provide explanations for the assessments of research risk and participant vulnerability reported above.*	Young children (3-5 yrs) so this study was classifying as medium to the age of this children.
4.5.C. *Does your application fall under minimal risk (i.e., was it assigned an overall risk level of 1 or a blue box on the minimal risk matrix above)?*	yes
**4.C. Behavioural Study Type - (Boxes 4.7 to 4.8)** [View Form]	
4.7.A Creation of a Research Database or Registry<br><br>*Does this study involve the creation of a research database or registry with a local custodian for future unspecified research?*	no
4.7.B. *Is the purpose of this application exclusively to obtain approval for the creation of a research database or registry? [Note: if the creation of the database or registry is part of a bigger project also included in this application, you must answer "no" below].*	no
Survey Research<br><br>*Is this a* ***minimal risk*** *study exclusively using a*	no

survey for data collection?	
Secondary Use<br><br>*Is this a **minimal risk** study exclusively analyzing previously collected data?*	no
**5. Summary of Study and Recruitment - Behavioural Study** [View Form]	
5.1.A. *Provide a brief statement about the project written in lay language. Do not exceed 100 words and do not cut and paste directly from the study proposal.*	We aim to 1) examine whether implementation of provincial guidelines and capacity-building interventions for physical activity (PA) and healthy eating (HE) impacts the environment, policies and practices in childcare settings and how implementation influences children's PA and HE. 2) To monitor the implementation processes, and examine influencing factors. This research will help to establish an understanding of current PA and HE environments, policies and staff practices in childcare settings to inform future policy and may also have implications for prevention of diseases associated with physical inactivity and unhealthy eating.
5.1.B. *Summarize the research proposal, including study purpose, hypothesis, study population, and research method.*	As a result of stakeholder consultation for the BC Provincial physical activity (PA) and healthy eating (HE) strategies, provincial practice guidelines targeting PA and HE in early years settings supported with capacity-building interventions (training, resources. support) were released/implemented in late 2016. These guidelines are voluntary and as such, childcare facilities and Early Years programs are not mandated to follow them. We are uniquely positioned to build upon this investment and evaluate the impact of the guidelines and capacity-building efforts (evaluating the level of uptake and implementation by childcare centres, family resources and Early Years Programs and their impact on policies and practices, and ultimately children's health).<br>Appetite to Play is the capacity building portion (training workshops and website) of the Early Years initiative. In February 2017, the contract for Appetite to Play was finalized.<br>Research objective(s) and question(s)<br>The primary research objective is to examine whether implementation of provincial childcare guidelines and capacity-building interventions for PA and HE impacts the environment, policies and practices in early years settings and how implementation influences children's PA and HE. The secondary objectives are to a) monitor the reach, dose and acceptance of training and resources, and b) examine the factors that influence implementation of the guidelines and predict higher implementation dose. The research questions are:<br><br>1) Assess whether the Appetite to Play Standards and supporting scale up intervention (training and resources) significantly change HE and PA policies of licensed childcare facilities and practices of providers over time. (AIM 1of CIHR with the addition of HE)<br><br>2) Identify (qualitatively and quantitatively) the hierarchy of factors that influence implementation of the Appetite to Play Standards and uptake of the multi-component scale up intervention. (AIM 2 of CIHR with the addition of HE)<br><br>3) To develop and pilot the content of the ATP+ program which complements the original ATP program which was offered in-person. This PAA request is targeting this aim.<br><br>4) To evaluate the efficacy of the ATP+ program on change in practices (i.e., more active play) and child outcomes (i.e., more physical activity and better fundamental movement skills).<br><br>The importance and contributions of the research This research will help to establish a baseline understanding of current PA and HE environments, policies and staff practices in early years settings prior to the release of provincial guidelines and capacity-building initiative (training, resources, support) for the early years. It will contribute to the understanding of the impact of policy and capacity-building and will help to inform future policy development and implementation in Canada in regards to the early years settings. It may also have direct implications for the prevention of diseases associated with physical inactivity, unhealthy eating and overweight/obesity.

	SUB-STUDY AIMs<br>With post-doctoral funding from the Michael Smith Foundation for Health Research, Dr. Olivia de Jongh Gonzalez will integrate a sub-study within the Good Start Matters! study. The original Appetite to Play initiative which we are supplementing with our ATP+ program is a provincial initiative that aims to engage children in active play and ensure that they adopt healthy eating habits. Dr. de Jongh Gonzalez program of research aims complement the suite of resources developed by Appetite to Play and add a critically needed focus on parenting – an aspect that Child Health BC and the Ministry of Health intends to expand upon. To address this needs, the sub-study has the following overall objective to conduct a mixed-methods randomized controlled trial to improve co-parenting practices and child obesity-related outcomes. The specific aims of this sub-study are to:<br><br>Sub-study AIM 1: To examine how usual parenting practices and variations between parents shape children's eating and PA behaviors.<br>Sub-study Aim 2: To determine how intra- and inter-parent variations in parenting practices influence: a) fluctuations in child behaviors over time as well as b) overall child health behaviors.<br>Sub-study Aim 3: To test the efficacy of a 3-months co-parenting practices intervention in: a) improving parenting practices (primary proximal outcome), b) reducing intra- and inter-parent variations in parenting practices (exploratory outcome), and c) improving child health behaviors related to active play and healthy dietary habits (primary distal outcome).
5.2. Inclusion Criteria<br><br>*Describe the participants being selected for this study, and list the criteria for their inclusion.*	Managers, staff and children in group childcare facilities (In harmonized UVic application managers, staff, and children were referred as groups 3, 4, and 6 respectively). This study targets group childcare facilities that provide care for children from infancy to school age (averaging 32 children per facilities) and account for 87% of the children in licensed care in BC. In June 2015, there were 3,044 licensed group childcare providers that provided care to 96,478 children across four service delivery areas.<br><br>Eligibility (AIMS 1 & 2): BC licenced childcare providers caring for children aged 30 months to 5 years are eligible. About 68% of the 3,044 licensed group childcare providers meet this criterion; 2,070 eligible facilities. Participants must work full time (defined as working at least 3 full days per week) as a Manager or Staff. Manager defined as Executive Director, or supervisor, or program manager.<br>Staff defined as Early Childhood Educator, instructor, or staff that cares for 30 months to 5 year old children.<br><br>Eligibility (AIM 3 & 4): Our primary sampling frame will be the childcare facilities that completed the 2022/23 EY Surveys. We will pilot our ATP+ program with three childcare centres within Vancouver in the summer 2022.<br>Given the intense nature of this Aim 4, and the cost of collecting onsite childcare-level and child-level data we will recruit 52 facilities from the the greater metropolitan area of Vancouver. In addition, facilities that provide care to at least 20 children (30 months to 5 year) will be targeted.<br>Children (ages 3 to 5 years old), who can provide objective data on PA and nutrition behaviours in the childcare setting through accelerometry, direct observation and fundamental movement skills (FMS) testing.<br><br>Eligibility (AIM 4 - substudy)<br>We aim to recruit 118 families (236 parents/legal guardian/primary caregivers) whose facilities are invited to participate in the ATP+ RCT. Eligible parents must: 1) Be a parent/legal guardian/caregiver with the primary custody of a 2.5-5-year-old child who attends a BC licensed childcare facility, and the child must be able to follow general nutrition and physical activity guidelines; 2) Be fluent in English both orally and in writing; 3) Enroll with a partner/co-parent/caregiver willing to participate in the parenting program with them (single parents/primary caregivers can still enroll in this program alone, or can choose to enlist 1 person who shares caregiving responsibilities with them (e.g., grand-parent, step-parent); and 4) Have a smartphone where each parent can individually receive notifications and be able to download and use the app on this smartphone. Specifically to participate in the parenting interviews, both parents/legal guardians/caregivers must be willing to be interviewed and must have downloaded and used the app.

	(February, 2024) Centers and participants located in Victoria that meet the rest of the above mentioned eligibility criterial will also be eligible to participate in the study.<br><br>(May 17, 2024) Specifically to participate in the parenting interviews sub-study, control group participants are also eligible to participate in the interviews if they meet the rest of the criteria previously described for the interview substudy except the requirement of having used the app as they are from the control group.<br><br>(March 4, 2025) ATP interviews: A total of 20 childcare educators and 10 managers who had access to the app as part of the randomized controlled trial (specifically those assigned to the intervention group) will be invited to participate in the interviews. A quota sampling approach will be used to ensure representation of both app users and non-users, allowing for an examination of perceived utility as well as barriers to use.
5.3. Exclusion Criteria<br><br>*Include details if otherwise eligible participants will be excluded due to other characteristics. If no exclusion criteria are applicable, enter n/a.*	This study excludes children in licensed family childcare.<br><br>(Aim 4 sub-study) If the family is currently participating in a pediatric weight management program or other nutrition program, or the child has any severe dietary or physical restriction that limit their ability to follow general nutritional or movement guidelines for 2-5-year-olds that will be delivered via the app.
5.4. Recruitment<br><br>*Provide a detailed description of the steps you will use to recruit participants. Include:*<br>*a) How will prospective participants be identified?*<br>*b) By what means will recruitment be done (e.g., public posting, direct contact, third party recruitment, etc.)?*<br>*c) Who will contact prospective participants?*<br>*d) If recruitment will occur in person, what sites will be used (e.g. doctor's office, hospital clinic, etc.)?*<br>*e) Attach all materials, including letters of initial contact, posters, scripts and advertisements, to Box 9.4.*	Data collection (AIMS 1 and 2): Licensed Child Care facilities will be identified via publically-available lists (e.g., BC ChildCare Map http://maps.gov.bc.ca/ess/hm/ccf and healthspace.ca /). In both the 2018/19 and 2019/20 survey waves, eligible childcare facilities will be sent via mail and email a preliminary notice (postcard) to alert them that our team will soon be inviting the managers and their staff to complete the EY survey and will included: a) a short description of the study; b) a link to our study website where they can sign-up to receive the survey online; and c) a date indicating when they can expect to receive the EY survey. These notices will be followed by an invitational package (mailed/emailed) detailing data collection procedures which includes: informed consent and a link to the online EY surveys (administrator/staff survey). Administrators or assistants will forward invitational emails to their staff. Those who agree to participate will receive an email link to the staff EY surveys. Research staff will be emailing and calling administrators and their assistants to follow-up and ensure an acceptable response rate. Administrators and staff surveys take about 20 minutes to complete on-line. Staff surveys include self-report of typical and “previous day” practices.<br><br>Note: In our original UVic harmonized application, used to collect the 2016/17 data, we originally planned to follow-up only those who signed up for the “longitudinal part of the study”. Given that there is such a high turnover of staff in these settings and that staff did not provide their contact information unless they opted to receive a nominal incentive we have no way of eliminating those who opted out as they completed the survey anonymously. As a results, we no longer ask staff to sign up for the longitudinal study but instead have identified this as a limitation of large scale evaluation (e.g., that those who completed the survey at each time point may not be the same people even though the sites are the same).<br><br>Two re-contact recruitment procedures were used to facilitate recruitment – these recontact procedures are no longer ongoing as these re-contacts have already happened, but are a part of the original UVic application:<br>1. Multi-age facilities meet our inclusion criterion but our skip patterns excluded them. As a result, the survey was amended, we resent the survey to multi-age facilities. See Multi-Age Facilites TTT<br>2. Managers were recontacted to respond to questions that were originally only asked to staff. Staff were recontacted to answer an additional 16 questions to align the “daily practices” questions with the beaseline data collection.<br><br>Data collection (AIM 3):<br>We will contact managers of childcare centres in the the greater metropolitan area of

Vancouver using the public lists used for Aims 1 and 2 and that are conveniently close to BCCHR. We will send the manager an invitation email (with ECE consent form in attachment). If interested, managers will need to connect with their staff to see whether they are interested in participating and that they would need to share they names and emails with the research team. To enroll the manager and staff into the pilot, everyone (manager + 3 ECE) needs to sign the consent form and complete the demographic forms (same as previously approved for EY survey).

Data collection (AIM 4):
(old content approved)
We will contact managers of childcare centres in the greater metropolitan area of Vancouver using the public lists used for Aims 1 and 2. Facilities will receive an email or letter invitation describing the purpose of the study and inviting facility to try out the ATP+ program and to participate in the evaluation of the ATP+ program. Those who expressed an interest in the study will be invited to have a zoom meeting with our team so that we can provide the facility with more information about the study or alternatively they can access the presentation about the study on their own. Those who are interested to proceed with the study will be mailed or emailed an invitation package that includes an invitation letter and a copy of the consent. After they have had the opportunity to review everything they can move ahead with registering for the intervention. As for the recruitment of the parents, childcare providers will facilitate delivery of invitation packages to parents which may include having the staff giving the invitations to the parents directly, emailing the invitation, or allocating a time when one of our staff can deliver the invitation packages to parents and answer any questions they may have about enrolling their child into the ATP+ program evaluation.

(modifications on Nov 22, 2023) We will contact managers of childcare centres in the greater metropolitan area of Vancouver using the public lists used for Aims 1 and 2. Facilities will receive an email and letter invitation describing the purpose of the study and inviting facility to participate in the evaluation of the ATP+ program. The research staff will call the manager to assess initial interests in the study and schedule an in-person meeting after the manager had the time to share broadly the invitation and assess facility’s willingness to proceed to an initial meeting. Those who expressed an interest in the study will be invited to have an in-person meeting with our team so that we can provide the facility with more information about the study. Research staff will meet with staff at the facility, do a brief introduction to the study, answer any of their questions and provide them an invitational package with a brochure and consent form. 1 to 2 weeks after, our staff will return to the facility to answer questions and collect consent forms (see section 6.6 to review consent process). As for the recruitment of the parents, childcare providers will facilitate delivery of invitation packages to parents which may include having the staff giving the invitations to the parents directly, emailing the invitation, or allocating a time when one of our staff can deliver the invitation packages to parents and answer any questions they may have about enrolling their child into the ATP+ program evaluation or the Parenting study.

Data collection for the sub-study (AIM 4):
As part of the ATP+ RCT parents of facilities were invited to receive the ATP+ training are asked to indicate whether they will consent to have their child be involved into the evaluation of the ATP+ program. A sub-set of these parents will be asked to participate into a second study – the parenting intervention where parents get to receive parenting tips to support active play or healthy dietary habits. When parents enroll for the evaluation of the parenting program, they have the option to select whether they want to complete only the quantitative assessment, or the qualitative interviews as well but these interviews will only enroll the first 15 families that select this component of the study). Parents will have the option to opt out of either study or to select which aspect the family wish to get involved. Parents learn about the ATP+ program evaluation and the parenting program by receiving an invitation package from the research which will be distributed either by the staff at the childcare facility, a research staff stationed at the childcare facility who would handout the packages to parents at the door and can answer questions about the study, via email sent by the childcare facility, or via our direct email and/or phone call if the parent has already registered for the ATP+ study and agreed to be contacted for future studies. In addition, a pamphlet / advertisement with information about the parenting study will be distributed to

centers for them to post it in the communications board, and the pamphlet will have a QR code which parents can scan to read the consent and enroll in the study if interested.
The invitational letter or email would include a copy of the consent form, and parents would be asked to review the information and to discuss the content of the package as a family given that enrollment into the parenting intervention as well as in the parenting interviews is designed to have both parents included in the study. If the family elects to register for the study, they can complete the registration either online or on paper. One parent (hereafter "the main parent") would register themselves, consent and provide information about the other parent/caregiver (hereafter the co-parent) who has agreed to be contacted for this study. Registration by the main parent would trigger an email to the co-parent to determine whether they wish to enroll into the evaluation of the parenting intervention. It will be made clear that registering a partner for the intervention does not mean that the coparent has to agree our invitation to enroll as participation is completely voluntary. However, only for the qualitative interviews component, if one parent decides not to register, the whole family would not be eligible as this component examines co-parental interactions and the role each parent plays to support their child's eating and physical activity behaviors and thus requires both parents to participate.

(Modifications to recruitment from February, 2024)
Eligible centers from Victoria, BC, will also be invited to participate in the study for Aim 4 main study and sub-study. Recruitment procedures will remain the same as the procedures described for centers recruited in the Greater Vancouver area.

(May 17, 2024) The sample size for the qualitative interviews for the parenting substudy was increased to 25 families instead of 15 families as we want to interview some control group families as well (initially we were including only intervention group families). Note that there will be no change in the interview guide, procedures for recruitment or interviewing these new families, or incentive. Everything will remain the same as the intervention group families interviewed, with the only difference that the interview guide contains a subset of questions regarding the perception of the intervention, which will not be asked to control group families as they are not appliable to them.

(Additional recruitment strategy - PAA Dec 20, 2024)
We have created an email invitation which would be sent to childcare facilities by the BC Director of Licensing Standard of Practice and mailing list that Child Health BC has. We are currently experiencing issues in recruiting childcare centers and we have talked with our partners on this project, Child Health BC, who indicated that an email from them and the BC Director of Licensing would likely not be considered as a spam email and may be read by those who receive it. These email invitations would be externally sent and include a link to our REDCap project for those interested. This is a new REDCap project created for this specific purpose, but it collects the same information we are already collecting in paper from the interested facilities that we are recruiting. When participants click on this link, prior to providing any data, they are shown the full consent that is currently REB-approved and that all facilities receive, and then they can register if interested. Once interested facilities complete this information in REDCap expressing their interest in our project, our team will contact them to assess their eligibility and move forward with enrollment if applicable, following the exact same processes that have been already described and approved for this project.

(ATP interviews, March 4, 2025) Qualitative interviews will be conducted with educators and managers after they used and/or had access to the ATP+ app for a period of 3-month. In the ATP+ consent that participants completed, participants were already informed that a subsample will be invited to qualitative interviews, so this new consent is for them to enroll in the interview. Prior to inviting any participants to the interview, we will assess whether the participants were assigned to the intervention condition receiving access to the ATP+ app at baseline, as this will determine their eligibility. All qualitative interviews will be conducted online using Zoom. Multiple modalities will be employed to invite educators and managers to engage in these interviews, including an email and/or printed letter sent to eligible educators and managers. Research staff will follow-up with either emails or calls to provide more information about the interviews and assess educators and managers' interest in participating in this supplemental study. The emails/letters will be sent 2-3 weeks prior to the

	3-month follow-up to allow for enough time for participants to review the consent form before the research team goes to the facility again. Interested participants will have the option to enroll in the interviews by completing the paper consent form or by consenting online via REDCap e-consent framework, which will provide the same detailed information as that shown in the printed consent. Eligible educators and managers who have already completed their follow-up assessments will also be invited to provide insights about the ATP+ program via emails and/or phone calls to discuss their interest in participating in the interview. If interested, they will be directed to the REDCap e-consent project for them to enroll online via REDCap e-consent framework.
5.5. Use of Records<br><br>*If existing records (e.g., health records, course grade sheets or other records/databases) will be used to access information about potential participants, please describe how permission to access this information, and to collect and use this information, will be obtained.*	As stated in section 5.4, we will also identify potential childcare facilities participants via publicly available online information such as but not limited to maps.gov.bc.ca and healthspace.ca. In our 2016/17 data collection, we collected enough information to identify which childcare facilities completed our survey. We will use this information to supplement our list of facilities. The data from all waves 2016/17, 2018/19, 2019/20, and 2022/23 will be linked but no identifiers will be stored with the data.
5.6. Summary of Procedures<br><br>*Describe briefly in a step-by-step manner what the researcher will be doing with participants, after they have been recruited and consented.*	MANAGERS AND STAFF SURVEYS (AIMS 1, 2 (quantitative), and 3)<br>All of our web-based survey will be administered using the Qualtrics platform at UBC (BCCHR). The questionnaires are specifically designed to measure PA and HE policies and practices in the early years setting. Managers will complete the Manager Questionnaire (Appendix EE), childcare staff will complete the Staff General Questionnaire (Appendix FF), and/or the Staff Today Questionnaire (Appendix GG), and short program staff will complete the Short Program Staff Questionnaire (Appendix HH), at a time and location most convenient for them. Participants will also be given the option to complete a printed version of the survey if they wish t and then mail/fax it back. Managers and staff complete these questionnaires as part of AIMS 1, 2, and 3. All these surveys were approved by the UVic Harmonized REB application. We will be amending these surveys to meet the objectives of the SSHRC and CIHR grants and modifications of these questionnaires will be submitted at a later date.<br><br>MANAGERS AND STAFFS SEMI-STRUCTURE INTERVIEWS (AIM 2 qualitative) The consenting participants will participate in telephone and/or in-person interviews. The interview dates/times will be scheduled according to the participants' availabilities and will last approximately 40 minutes. A trained researcher will facilitate the interview, following the interview scripts (Appendices AA). The researcher will take notes and/or audio record the interview, which will then be transcribed.<br><br>PILOT DATA COLLECTION (AIM 3)<br>After receiving all forms from the manager + 3 ECE from their facility, participants will receive access to the APT+ program with enrolment instructions via email. After competing the 3 online modules, we will schedule a 1-hour individual and/or focus-group interviews online via Zoom, according to the participants' availabilities so they can share their expert opinion about these resources. A trained researcher will facilitate the interviews, following the interview scripts. The researcher will take notes and/or audio record the interviews.<br><br>CHILD DATA COLLECTION (AIM 4)<br>AIM 4 involves a more intensive study on the consented childcare facility environments and the impact of changes in policies and practices on consented children's (ages 3-5 years old) PA and HE behaviours and includes the following components:<br>a) The Good Start Matters Equipment Environment Space observation sheet (Attached in Section 9.5) provides greater detail on the protocol that will be used. Briefly, this tool requires direct observation to assess facilities' environments, children's PA and nutrition behaviours throughout the childcare day. The researchers will conduct 2 full days of

observations in each childcare and children will wear the physical activity devices for 4 days.
b) Wearable devices: Children will wear two devices: 1) the AX3 Axivity accelerometer (Axivity, Newcastle Helix, UK) and 2) the GPS forerunner 230 watch (Garmin, KS) as GPS devices have higher accuracy to detect indoor and outdoor time of preschoolers.

An accelerometer (aka wrist monitor) is a small, non-invasive device that tracks PA levels. The consented children will wear one during childcare hours for three full days at each time period that they consent to. A trained researcher will put the accelerometers on the children in the morning and take them off at the end of the day.
c) Assessment of Fundamental Movement Skills (FMS): All children in childcare will participate in the FMS activities as a part of their PA for the day (approximately 1 hour each time period). Only the consented children's FMS will be assessed as part of the PA sub-study. The valid and reliable TGMD-3 tool (Appendix JJ) will be used, in which trained researchers will demonstrate various FMS to the children, and then video record the consented children doing them. Their skills will then be analyzed by a trained researcher. Video recordings of children doing the FMS testing will only be used for analysis only and will destroyed as soon as the skills have been analyzed.
d) Policy document: The staff will also be asked to provide the researcher with their facilities' policy documents, as outlined in the Good Start Matters Equipment Environment Space observation sheet (Attached in Section 9.5).
e) Some centres will also be asked to participate in a recording of lunchbox contents. A digital photo and paper recording of contents will be taken. Detailed protocol of this assessment can be found in Section 9.1 Lunchbox Dietary Data Collection Protocol, and associated forms in 9.5 entitled School Food Checklist.
f)Parents will be asked to complete a short survey about their child asking for demographic information, child's physical activity behaviour, and structured physical activities the child participates in, the associated form is in Section 9.5 entitled parent survey.
Note: All these child data collection procedures were approved in the UVic Harmonized REB application, except we added the GPS device as this was missing from our UVic application

THE INTERVENTION ATP+ (AIM 4):
(old content approved): After receiving consent from at least 60% of ECE (~1 manager + 2 staff) childcare centres will complete the baseline assessments at child and facility levels. Following, half of the centres will receive the ATP+ program immediately or 3 months after. Measures will be assessed again 3 months later.
A subset of managers and educators (~30) will participate in interviews to discuss their experiences in using the ATP+ program. We will randomly select participants and those who are selected will receive an invitation package and will be given the opportunity to participate in this optional component of the study. We will conduct qualitative interviews to understand how the Early Childcare Educators and managers are using the ATP+ intervention. The invitational package (will include a letter and consent form) and content of these qualitative interviews will be developed at a later time by a PhD student who has an interested in evaluating the effectiveness of the ATP+ intervention. This component of the study will be submitted at a later date to REB and no interviews and invitation will proceed until we have REB approval.

(modifications on Nov 22, 2023): Eligible and interested managers and educators would: 1) complete the online ATP+ program – where 26 facilities would start the program after the baseline assessments are completed and the other 26 facilities would receive the program 3 months later; and 2) complete online surveys at 2 time points (baseline and 3-month). The survey take about 30-35 minutes to complete. In addition, one staff would coordinate the 3-day data collection at their facility which would be done by our research staff and include: 1) Observing children's active play and the activities provided by the educators (1 full day, observation form is the document named "RCT(Aim4) Modified EPAO_v10" approved in the PAA of April 27, 2023); and 2) Administering a Test of Gross Motor Development (TGMD-3) that assess manipulative, locomotor and balancing skills and takes about 15-20 min to administer per children.
A subset of managers and educators (~30) will participate in interviews to discuss their experiences in using the ATP+ program. We will randomly select participants and those who are selected will receive an invitation package and will be given the opportunity to

participate in this optional component of the study. We will conduct qualitative interviews to understand how the Early Childcare Educators and managers are using the ATP+ intervention. The invitational package (will include a letter and consent form) and content of these qualitative interviews will be developed at a later time by a PhD student who has an interested in evaluating the effectiveness of the ATP+ intervention. This component of the study will be submitted at a later date to REB and no interviews and invitation will proceed until we have REB approval.

SUB-STUDY (AIM 4):
A Mixed Methods study will be integrated into the Good Start Matters ATP+ Randomized Controlled Trial (RCT). Specifically, parents of children attending the invited childcare centers will be invited to enroll into the Good Start Matters Parenting study. The Good Start Matters Parenting study is a 2-arms RCT where families are randomized into a control or an intervention condition. Intervention group families receive a parenting intervention though a mobile-health (mHealth) app, namely Pathverse, to promote positive feeding, activity and media parenting practices and support healthy child behaviors. Data collection occurs at baseline and at 10 weeks. After the 10 weeks assessment, control group families will get access to the app. Participants (2 parents or 2 primary caregivers) will complete 3 types of measurement: a) questionnaires inquiring about their usual behaviors and child behaviours, b) questionnaires inquiring about their behaviors in a particular day – an Ecological Momentary Assessment (EMA) of their behaviors every day during a 14-day period at each data collection time point (this is a short questionnaire that takes 5-10 minutes a day to complete. Please note that there is no cost to participants for using or downloading the app, and that any cost related to the app will be covered by the research team.); and c) a 1-hour Zoom interview (only a subset of 15 families) to inquire about their parent-coparent-child interactions and their experiences while using the app. Interviews will be done only at 10-weeks – not baseline- and will be recorded (we will schedule the interview according to the participants' availability and a trained staff will facilitate the interviews, following the interview scripts).
Pathverse is a platform that was designed to minimize the cost associated with developing an app. The app includes templates which app developers can use and our study will use the templates that have been already developed. It will cost us about $300 per month to use the app and this cost is associated with storing our data. Based on our initial look at the app, we may require some customization which would cost us about $2000. The research team does not have any financial interest in the platform nor would we get any revenue from using the platform.
The app-analytics data related to the Parenting intervention are stored on the Pathverse app whereas the data derived from the measurement tools are stored in REDCap. These app-analytics data derives from parental interactions with the app (i.e., the usability data related to parental use of the features used in the app while setting a goal in the app, completing a self-monitoring check-in in the app, interacting with other parents in the app, etc.). The data remain in the Pathverse platform for up to a 1 year after the study ends. The research team will extract all the data from the app at the end of the study.

Temporary change tor procedures (Starting Oct 13, 2023) ; Given that we are collecting repeated data for the parenting study, we need to send multiple automated survey invitations from REDCap. The REB has approved that we transfer parent email and cellphone from the REDCap Consent project to the Survey project so that we can the survey multiple times (email is needed to complete the survey more than 1 time and the phone is needed to complete the daily diary data). This transfer of data needs to be programmed by the REDCap team. We have submitted this request to the REDCap team in August and they anticipate having the programming done by Mid-November given that they are short staffed. We are requesting approval to ask parents to provide their emails and cellphones as part of the REDCap Survey project while we wait for the REDCap team to transfer this data from the Consent project. This is a temporary solution while we await for the programming to be complete. Note that this temporary approach does not change already approved REDCap dataset structures and data storage, we would only skip the data transfer process temporarily and parents would be asked twice to provide their email and phone number.

	(February, 2024) Eligible centers and participants from Victoria, BC, will also be invited to participate in the study, but procedures will be the same as those used with centers in the Greater Vancouver area.<br><br>(May 17, 2024) For the parenting interviews sub-study, we will increase our sample from 15 to approximately 25 families as we decided to interview 10 additional families from the control group (initially we had planned to interview only intervention group participants). Note that there will be no change in the interview guide, procedures for recruitment or interviewing these new families, or incentive. Everything will remain the same as the intervention group families interviewed, with the only difference that the interview guide contains a subset of questions regarding the perception of the intervention, which will not be asked to control group families as they are not applicable to them.<br><br>(Additional recruitment strategy - PAA Dec 20, 2024)<br>We have created an email invitation which would be sent to childcare facilities by the BC Director of Licensing Standard of Practice and mailing list that Child Health BC has. We are currently experiencing issues in recruiting childcare centers and we have talked with our partners on this project, Child Health BC, who indicated that an email from them and the BC Director of Licensing would likely not be considered as a spam email and may be read by those who receive it. These email invitations would be externally sent and include a link to our REDCap project for those interested. This is a new REDCap project created for this specific purpose, but it collects the same information we are already collecting in paper from the interested facilities that we are recruiting. When participants click on this link, prior to providing any data, they are shown the full consent that is currently REB-approved and that all facilities receive, and then they can register if interested. Once interested facilities complete this information in REDCap expressing their interest in our project, our team will contact them to assess their eligibility and move forward with enrollment if applicable, following the exact same processes that have been already described and approved for this project.<br><br>(ATP interviews, March 4, 2025) Qualitative interviews will be conducted with educators and managers after they used and/or had access to the ATP+ app for a period of 3-month. In the ATP+ consent that participants completed, participants were already informed that a subsample will be invited to qualitative interviews, so this new consent is for them to enroll in the interview. Prior to inviting any participants to the interview, we will assess whether the participants were assigned to the intervention condition receiving access to the ATP+ app at baseline, as this will determine their eligibility. All qualitative interviews will be conducted online using Zoom. Multiple modalities will be employed to invite educators and managers to engage in these interviews, including an email and/or printed letter sent to eligible educators and managers. Research staff will follow-up with either emails or calls to provide more information about the interviews and assess educators and managers' interest in participating in this supplemental study. The emails/letters will be sent 2-3 weeks prior to the 3-month follow-up to allow for enough time for participants to review the consent form before the research team goes to the facility again. Interested participants will have the option to enroll in the interviews by completing the paper consent form or by consenting online via REDCap e-consent framework, which will provide the same detailed information as that shown in the printed consent. Eligible educators and managers who have already completed their follow-up assessments will also be invited to provide insights about the ATP+ program via emails and/or phone calls to discuss their interest in participating in the interview. If interested, they will be directed to the REDCap e-consent project for them to enroll online via REDCap e-consent framework.
5.7. Research Types<br><br>*Select all that apply to your study. Please review the research methods descriptions before responding. If none*	Naturalistic Observation<br>Community Based Research (collaboration with community on design and methods)<br>Videotaping<br>Expert Interviews

apply, please select "None of these Methods"	
**6. Participant Information and Consent Process - Behavioural Study** [View Form]	
6.1. Time to Participate	Childcare staff (Groups 3 and 4) should take about 20 minutes to complete the surveys.<br>Children (groups 6) will wear the accelerometers from the time they arrive at the childcare facility and until they leave and the FMS test is expected to take 15 to 20 minutes to complete.<br>AIM 3: for the pilot, staff will spend 60-90 min/weeks for a total of 3 weeks on the online intervention plus 60 min on an interview.<br>Aim 4-part 1: staff will spend 30-40 min on the survey.<br>Aim 4 – Childhood educators and Managers will spend 90 min/weeks for a total of 3 weeks on the online intervention. They will spend about 45 minutes completing the questionnaire at baseline and again at 3-month.<br><br>Aim 4 - sub-study: Participants who volunteer for the evaluation of the parenting intervention will spend15-20 minutes on the intervention for 9 weeks, need 15-20 minutes at baseline and 10 weeks to complete the assessments. In addition, the Ecological Momentary Assessment is completed at baseline and at the 10 weeks mark, will take 5-10 min a day and is completed for a duration of 14 days at each occasion. Participants who volunteer to be interviewed will devote 60 minutes at the 10-weeks to complete 1 interview.<br><br>(ATP interviews, March 4, 2025) The ATP qualitative interviews will take between 45-60 minutes.
6.2. Risks and Mitigation	An observer will be in the childcare facility collecting data and the childcare staff might feel self-conscious about what they are doing in front of the data collector. There is no risk to managers and staff who are not abiding by the provincial guidelines as indicated in their survey responses and/or by our observations since these guidelines are voluntary and not mandated.<br>The consent process will minimize the risk as only those who are willing to have an observer in their setting will volunteer. The consent will emphasize that we are interested in the overall policies and practices of the childcare facility and that the observation is of the children’s PA and eating behaviours. All facility names will be coded and participants’ names will be removed from the surveys once they have received their gift cards. For those who are participating in the longitudinal study, their names will be coded and only used to re-contact them each year.<br>The key to addressing the issue is being empathetic and establishing trust through communication. If a childcare staff member feels uncomfortable we will remind them that we are observing the children’s PA and eating behaviours. If necessary we will not complete the observation. Our experience has been that the staff quickly forget that a member of the research team is in their facility as they are busy tending to the children’s’ needs and we make a point of being as unobtrusive as possible.<br>Safeguards taken to ensure voluntariness and minimize undue influence, coercion or potential harm:<br>The research team rather than the PI will recruit the Advisory Committee members. A presentation will be made to the Advisory Committee but individual invitations and consents will be sent out so there is no ‘group’ or ‘PI’ pressure and individual interviews will be conducted rather than a focus group so they have the ability to refuse rather than feel social pressure to participate.<br>The recruitment materials/consent forms will include the following clauses to ensure participant voluntariness and minimize undue influence: (1) the stakeholders/managers are not conducting the study; (2) relationships and employment will not be affected in any way whether managers/staff choose to participate or not; and (3) the stakeholders/managers will not be aware of who chooses to participate and who does not.<br>Another possible inconvenience includes the amount of time taken to complete the surveys and interviews. Some of the children may find the accelerometers (aka wrist monitor) slightly irritating to wear, but our experience is that most children do not even notice that they are wearing them after the initial novelty wears off. Childcare staff might feel self-conscious with an observer present.<br>The lunchbox observation may pose an increased risk to children with food allergies, therefore we will exclude all children with a reported food allergy in order to prevent any

	cross contamination that could occur.<br><br>There are no known risks to children participating in the evaluation of the ATP+ program. Some of the children may find the accelerometer (aka wrist monitor) slightly irritating to wear, but our experience is that most children do not even notice that they are wearing them after the initial novelty wears off. Parents and legal primary guardians who utilize the SMARTER parenting app will engage in more discussions about their parenting practices and for some parents this may helpful while for others this may take time and highlight areas where they may not agree with their partners, This may create some discomfort for some families and a list of resources will be provided to parents / legal guardians if feel they need more support.<br>Among the ATP+ course activities, participants will have the option to post comments on the discussion board and interact with fellow participants by liking or replying to the posts. Every post includes the student name, date and time. Although our expectation is that these interactions will remain positive and professional, it is possible for someone to post an inappropriate comment. To mitigate any negative interactions, staff members will monitor every week the discussion board by:<br>1. enabling the notifications feature to receive an email every time a new post is added; and<br>2. reviewing every week the content of the discussion board and act as a moderator to eliminate inappropriate comments.
6.3. Potential Benefits	To Participants: The stakeholders and delivery agents will get to be actively involved in the implementation process and evaluation of PA and HE guidelines. Childcare managers and staff and short program staff will be provided with a greater understanding and/or a heightened awareness of PA and HE policies and practices in their early-years facilities/programs. In late 2016 they will be given provincial practice guidelines targeting PA and HE in early-years settings with capacity-building interventions (training, resources, support). The children may also gain a heightened awareness of PA.<br>The evaluation of the ATP+ program and the SMARTER parenting intervention will help inform policy development and the implementation of early years initiatives in British Columbia and Canada, and may have direct implications for the prevention of diseases associated with unhealthy lifestyle behaviors.
6.4. Impacts on Community	Benefits to Society: Improving children's PA and eating habits in the early years has been shown to track into adolescence and adulthood. This research may therefore have direct implications on the prevention of diseases associated with physical inactivity, unhealthy eating and overweight/obesity such as cardiovascular disease, diabetes and certain cancers.<br>Benefits to the State of Knowledge: This research will provide a greater understanding of the current PA and HE environments, policies and staff practices in early years settings prior to the release of provincial guidelines and capacity-building initiative (training, resources, support). It will also evaluate the impact of the guidelines and capacity-building efforts. Broadly, it will help inform future policy development and implementation in Canada and efforts to change practices in regards to PA and HE in the early years settings.
6.5. Reimbursement and Incentives	Groups 3-5 will be offered a small incentive ($5 e-gift card) for participating in the surveys. They will have the option of writing their name and contact information at the end of the survey if they would like an e-gift card. The other groups will not receive an incentive. Participants in Groups 3-5 who do not complete the portion of the survey that asks if they would like an e-gift card or provide their contact information will not receive an e-card. If they complete the survey and have provided their contact information, but then would like their survey data removed, they will get to keep the e-gift card if it has already been sent. There will be no compensation for participating in the research (e.g., for transportation, parking, childcare, etc.)<br>For Group 6: Participating facilities will receive $80, staff and managers who complete the 45-min survey will receive $25 each time they complete the survey, and parents of children who participate in the study will receive $25 for each 3 days of data collection.<br><br>Aim 3: we will provide $150 to educators and managers who pilot the content of the ATP+ program.<br><br>Aim 4-part 1: managers and educators who choose to participate will receive $25 cash and be entered to win $100 cash (1 in 30 changes of winning as there will be a draw for every

	30 participants). All compensations will be sent via e-transfer. Any remuneration or compensation that are not claimed within 21 days of being sent via e-transfer to the participant will be forfeited.<br><br>(August 23, 2023 - Changes made)<br>Aim 4 (RCT that evaluates efficacy of ATP+) - Childcare facilities will receive $75 for facilitating the data collection at their sites, and educators and managers will receive $50 for completing the survey ($25 for the baseline assessment and $25 for the 3-month assessment), pro-rated for the components completed. Participants that complete both baseline and follow up surveys will be entered in a draw to win $100 (1 in 30 chances of winning as there will be a draw for every 30 participants). All compensations for participants recruited prior to August 23, 2023, are sent via e-transfer, and in these cases, compensations not claimed within 21 days were forfeit. All compensations for participants recruited after August 23, 2023 will be sent via Amazon.ca gift after the 3-month follow-up is completed. Finally, educators and managers who complete the on-line training will receive a certificate for 5 credit hours of professional development if they completed the old training in Canvas, and 3 credits if they completed the new training in the Pathverse app.<br><br>Aim 4 - sub-study (Parenting RCT): Families who enroll in the evaluation of the parenting intervention will receive up to $214 gift card for their participation ($107 for each participating parent/legal guardian/primary caregiver – pro-rated if not all assessment tools are completed). Each participating parent/legal guardian/primary caregiver is compensated as follow: 1) $40 for completing two 15-20-minues survey at baseline and at the 10 weeks ($20 for each survey per parent) and 2) $42 for completing two daily check-ins for 14 days. The daily check-ins take 5-10 minutes to complete and are completed at baseline and at the 10 weeks ($1.5 for each daily check-in completed). In addition, each parent who enroll in the parenting interviews substudy will receive in total $25 gift card ($50 per family) for completing one 1-hour interview. Finally, parents/legal guardian/primary caregivers who complete both baseline and follow up long surveys will be entered to win $100 gift card (1 in 30 chances of winning as there will be a draw for every 30 participants). All gift cards will be Amazon.ca gift cards and will be sent directly from Amazon. As no parents were recruited for the Parenting RCT before August 23, 2023 (date in which compensations were changed from e-transfer to gift card), the only compensation to be used with parents is Amazon gift cards.<br><br>(ATP interviews, March 4, 2025) Participants completing the ATP qualitative interviews will receive $30 gift card paid using the same procedure as for the ATP RCT. In addition, all who take part in the interviews will be entered to win a $100 gift card.
6.6. Obtaining Consent<br><br>*Include details of where and when consent will be obtained and how it will be documented.*	Groups 1 and 2 will be provided with a consent form to be signed prior to participating in an interview. In terms of the Capacity Building Tracking form that Delivery Agents (Group 2) are asked to complete, they will be instructed to only use meeting participants' general titles (e.g. manager, staff) and not specific names in their records, as we do not require this identifiable information (the purpose is to track the capacity building process). This is outlined in the tracking sheet and in the consent form. As such we will not seek third party informed consent.<br>Groups 3 and 4 will be provided with an implied consent in the email where they are given their online survey link. Managers and childcare staff who agree to participate in the longitudinal study (to complete a survey in years 2, 3, and 4 as well) will be presented with an implied consent and the survey link each year. Managers and childcare staff that are recruited for the year 3 survey will also be presented with an implied consent and survey link in year 3.<br>Group 5 will be presented with an implied consent in the email where they are given their online survey link in years 1, 2 and 3.<br>Group 6 – Two paths to recruitment to participate in the substudy will occur. Eligible childcare centres in the lower mainland will be contacted. Depending on if these childcare centres are single site organizations, or a larger childcare organization with multiple sites we will contact them directly or via the director/manager of the larger organization. If the

manager of the larger organization approved, we will then contact the individual single sites within the larger organization. This is outlined in 9.1 Action Steps Flow chart with R numbers. Once individual single sites have expressed interest, we will meet with managers or supervisors and staff to answer questions and provide consent documentation. Once sufficient staff (~80%) have consented, we will move to recruiting child participants. Research staff will provide the children's parents/guardians with a consent form to be signed prior to the children wearing accelerometers and completing the FMS testing. The children's assent will also be obtained prior to the FMS testing and each day prior to being fitted with the accelerometer. We will not ask the parents/guardians to provide consent to the facility observation as the observation will not be specific to individual children, but will instead be a survey of the program environment and practices at the facility. Due to the nature of this, we do not require consent as it will be impossible to single out child in the overall observation. This is noted in all of the child recruitment scripts which provides general information on the observations.
Parents/guardians of the children will be asked to consent at each measurement period (Fall of year 1, Spring of year 1, Fall of year 2, Spring of year 2). Data collection will include those children that initially agree to participate (Fall of year) in addition to children who did not consent initially but then consent at the subsequent time periods (i.e. Fall of year 2, Spring of year 1 and 2). Children will be asked to assent to the accelerometers each day they are put on, and each time they participate in FMS testing.

Group 8 Early Years providers that are interested will register for the Appetite to Play workshop on their own, and then will be offered a survey and consent form at the workshop. They may choose to sign the consent form and complete the surveys before and after the workshop and also self-select to be contacted for a qualitative interview or not.
The Training Coordinator at the YMCA works closely with the delivery agents and will send the email inviting them to participate in an interview. The email will contain the script attached in Appendix MMM. Delivery Agent Interview Invitation.
Withdrawal
Participants will be told in the consent letters/implied consent that they are able to withdraw from the research at any time. All consents say "If you do decide to participate, you may withdraw at any time without any consequences or any explanation." Participants are asked to contact the research team in person (during an interview) or by telephone/email (contact information is provided).
INTERVIEWS If a participant in Groups 1 or 2 or Pilot would like to withdraw from an interview after consenting but prior to it taking place, they would be asked to notify the corresponding researcher by telephone or email. If they would like to withdraw during the interview, they will be told that they can just notify the interviewer and they can have their data removed from the study.
SURVEYS Participants in groups 3-5 can stop completing the survey at any time; the research team would not know since the surveys are anonymous. Those who are completing the cross-sectional survey (years 1 and 3) will not be able to withdraw their survey data once submitted since there are no identifiers on the survey. However, participants who have submitted the survey and have included their name for the incentive and/or to participate in the longitudinal study and are therefore using an identifier on their survey (in years 2-4), can have their data removed upon request. If they have already been sent the e-gift card incentive they will be able to keep it.
PA/HE SUB-STUDY IN CHILDCARE FACILITIES If a child in Group 6 would like to withdraw from wearing the accelerometer or participating in the FMS testing, he/she would just have to tell the researcher or the childcare staff, or the parent/guardian could notify the corresponding researcher by telephone, email or a note sent by way of the childcare staff.
If participants in Groups 3-5 have identified themselves for the e-gift card and/or to participate in the longitudinal study and then wish to withdraw, they will be sent an email asking if their data could still be used and explaining that it would be anonymous in the results/final report. Participants in the longitudinal study (years 2-4) who are including their unique identifier on their surveys (given by the corresponding researcher) can request to have their data removed by emailing by the researcher. Anyone who wishes to have their data removed will be completely erased from the records. If participants have NOT identified their desire for an e-gift card or to participate in the longitudinal survey, we cannot identify their survey as it would be anonymous.
If a participant in Group 6 wishes to withdraw, the parent/guardian will be told on the consent form "If you choose to withdraw from the study, and do not want your accelerometry

and FMS data included, please contact the researcher." It will not be used in the analysis and will be destroyed. Upon request for interviews, surveys with participant's names included, accelerometry and FMS testing. It is logistically impossible to remove individual participant data.
AIM 3: eligible childcare centres in the Vancouver will be contacted. If the manager approves, they will receive a copy of the ECE consent form via email. Managers will invite their ECE to participate in the study by sharing the consent form and let them know that they would need to share their names and emails with the research team. Interested ECE will provide their contact information to the research team to receive an email with the link to the consent form via REDCap survey. If they provide consent, they will be ask some demographic questions (same questions as the previously approved for group 3 and 4) and will not include any other information (similar to the previous approval). Participants will have ample time to review the consent to participate in the pilot testing the content of the ATP+ program.

(old content approved)
Aim - for Aim 4: Those who expressed an interest in the study will be invited to have a zoom meeting with our team so that we can provide the facility with more information about the study or alternatively they can access the presentation about the study on their own. Those who are interested to proceed with the study will be mailed or emailed an invitation package that includes an invitation letter and a copy of the consent. After they have had the opportunity to review everything they can move ahead to be part of the evaluation of the ATP+ program. Registration is done online or on paper. The online/paper registration has the invitation, the consent form which participant need to review and consent to participate and then they can register. As for the recruitment of the parents, childcare providers will facilitate delivery of invitation packages (includes invitation letter and a copy of the consent form) to parents which may include having the staff giving the invitations to the parents directly, emailing the invitation, or allocating a time when one of our staff can deliver the invitation packages to parents and answer any questions they may have about enrolling their child into the ATP+ program evaluation. The invitation letter includes a an online link that parents can use to register their child. The link includes the invitation letter, the consent form which they complete online and if they consent they then register their child into the study. In addition, participants will have the option to fill out the paper forms of the consent form and contact information form which will be collected by research staff at a later date.

Aim 4 - substudy (Parenting RCT): If parents wish to enroll in the study they will need to access the registration site and consent for their participation from the email they received from the research team, or they can provide their consent in paper by signing the printed forms and giving the signed forms to our research staff. It will be made clear that they do not need to participate even though a person close to them provided us with their information. As the sub-study is a co-parenting intervention, we need to invite both parents but families can still proceed with the study if the person they listed as their partner for the intervention elect to not participate. We will also clarify that even if they sign the printed forms, we will assess their eligibility before enrolling them into the study, and they will be notified via email if they do not meet eligibility criteria.

(modifications from Nov 22, 2023)
Aim - for Aim 4: Those who expressed an interest in the study will be invited to have an in person meeting with our team so that we can provide the facility with more information about the study. At the initial meeting, each staff receives a brochure, invitation, consent form, and copy of the questionnaire. After staff and manager have had the opportunity to review the invitation materials (1-2 weeks after the initial meeting), the research staff will call the manager to assess initial interests in the study and schedule a second in-person meeting to clarify remaining question and collet the forms. Registration, consent and baseline survey are completed on paper but follow up survey is done either on paper or online via a REDCap URL sent directly to their email which is obtained during the consent process – this link includes a de-identified numeric code that is unique for each participant. As for the recruitment of the parents, childcare providers will facilitate delivery of invitation packages (includes invitation letter and a copy of the consent form) to parents which may include having the staff giving the invitations to the parents directly, emailing the invitation, or allocating a time when one of our staff can deliver the invitation packages to parents and

	answer any questions they may have about enrolling their child into the ATP+ or the Parenting programs. The invitation letter includes an online link that parents can use to register. The link includes the invitation letter, the consent form which they complete online (e-consent using REDCap framework) and if they consent they then register their child into the ATP and/or Parenting studies. In addition, participants will have the option to fill out the paper forms of the consent form and contact information form, which will be collected by research staff at a later date.<br><br>Aim 4 - substudy (Parenting RCT): Parents enroll for the Parenting study following the same process as for the ATP study. If parents wish to enroll in the Parenting study they will need to access the registration website and consent for their participation online, which can be done from the email they received from the research team, or by scanning a QR code that we print in the printed paper forms. When consenting online, parents consent via REDCap e-consent framework. Alternatively, parents can provide their consent in paper by signing the printed forms and giving the signed forms to our research staff, in which case we would manually add their information into REDCap. It will be made clear that they do not need to participate even though a person close to them provided us with their information. As the Parenting sub-study is a co-parenting intervention, we need to invite both parents but families can still proceed with the study if the person they listed as their partner for the intervention elect to not participate. We will also clarify that even if they sign the printed forms, we will assess their eligibility before enrolling them into the study, and they will be notified via email if they do not meet eligibility criteria.<br><br>(March 4, 2025) In the ATP+ consent that participants completed, participants were already informed that a subsample will be invited to qualitative interviews, so this new consent is for them to enroll in the interview. Multiple modalities will be employed to invite educators and managers to engage in these interviews, including an email and/or printed letter sent to eligible educators and managers. Research staff will follow-up with either emails or calls to provide more information about the interviews and assess educators and managers' interest in participating in this supplemental study. The emails/letters will be sent 2-3 weeks prior to the 3-month follow-up to allow for enough time for participants to review the consent form before the research team goes to the facility again. Interested participants will have the option to enroll in the interviews by completing the paper consent form or by consenting online via REDCap e-consent framework, which will provide the same detailed information as that shown in the printed consent. Eligible educators and managers who have already completed their follow-up assessments will also be invited to provide insights about the ATP+ program via emails and/or phone calls to discuss their interest in participating in the interview. If interested, they will be directed to the REDCap e-consent project for them to enroll online via REDCap e-consent framework.
6.6.A. Waiver of Consent	
6.7. Time to Decide	At least 24 hours prior to the procedure
6.8. Capacity to Consent	No

Will participants have the capacity to give fully informed consent on their own behalf?	
6.8.A. *Provide details of the nature of the incapacity (for instance, young age, mental or physical condition).*	Young age
6.8.B. *If a participant does not have the capacity to give fully informed consent, who will consent on their behalf? Ensure the relevant consent form (parent/caregiver, substitute decision maker, legally authorized representative) is attached to page 9.*	Group 6 – Parents/guardians of the children will be asked to consent at each measurement period (Fall of year 1, Spring of year 1, Fall of year 2, Spring of year 2). Data collection will include those children that initially agree to participate (Fall of year) in addition to children who did not consent initially but then consent at the subsequent time periods (i.e. Fall of year 2, Spring of year 1 and 2). Children will be asked to assent to the accelerometers each day they are put on, and each time they participate in FMS testing. We will also obtain signed or verbal assent for non-competent participants (children).
6.8.C. *If a participant does not have the capacity to give fully informed consent, will they be able to give assent to participate?*	yes
6.8.D. *If yes, explain how assent will be sought. Please be sure to attach copies of the assent form to page 9.*	We will obtain signed or verbal assent for non-competent participants (children)
6.9. Ongoing Consent	
6.10. Provisions for Consent (e.g., special assistance, Braille, translations/translator)	
6.11. Restrictions on Disclosure	
**7. Number of Participants - Behavioural Study** [View Form]	
7.1. External Approvals<br><br>***A.*** *Other Institutions:*	no
B. *Please select "Add" to enter the name of the institution and attach the approval letter if received.*	**Name of Institution**
C. *Other Jurisdiction or Country (if "NO," go to 7.1.G):*	no
D. *Please select "Add" to enter the name of the jurisdiction or country and if you have already received approval attach the approval letter.*	**Name of Jurisdiction or Country**

E. *Has a Request for Ethics Approval been submitted to the institution or responsible authority in the other jurisdiction or country? (**Append a copy of any such document to this application once it is received**).*	no
F. *If a Request for Approval has **not been** submitted, provide the reasons below:*	
G. *Does this research focus on Indigenous peoples, communities or organizations?*	no
G.1.A. *Will the research be conducted on Indigenous reserves, Métis settlement(s), or lands governed under a self-government agreement or an Inuit or First Nations land claims agreement?*	
If yes, please provide details:	
G.1.B. *Do any of the criteria for participation include membership in an Indigenous community, group of communities, or organization, including urban Indigenous populations?*	
If yes, please provide details:	
G.1.C. *Does the research seek input from participants regarding a community's cultural heritage, artifacts, traditional knowledge or unique characteristics?*	
If yes, please provide details:	
G.1.D. *Will Indigenous identity or membership in an Indigenous community be used as a variable for the purposes of analysis?*	
If yes, please provide details:	
G.1.E. *Will the results of the research refer to Indigenous communities,*	

peoples, language, history or culture?	
If yes, please provide details:	
G.2. Community Engagement<br><br>***G.2.A.*** *If you answered yes to questions a), b), c), d), or e), have you initiated or do you intend to initiate an engagement process with the Indigenous collective, community or communities for this study?*	
G.2.B. *If you answered "Yes" to question G.2.A., describe the process that you have followed or will follow with respect to community engagement. Include the role or position of those consulted, including their names if appropriate. Attach any documentation of consultations (i.e. formal research agreement, letter of approval, email communications, etc.) below.*	
Attachment:	
G.3. No community consultation or engagement<br><br>*If you answered "no" to question G.2.A., briefly describe why community engagement will not be sought and how you can conduct a study that respects Indigenous communities and participants in the absence of community engagement.*	
H. *Registration for Publication of Clinical Trials.*	yes
If 'Yes', click 'Add' to enter the following information.	**Has it been registered?** / **Indicate the Authorized Registry used:** / **Enter your Clinical Trial unique identifier:**<br>yes / ClinicalTrials.gov / NCT05669378<br>yes / ClinicalTrials.gov / NCT05802160

7.2. Number of Participants<br><br>***A.*** *How many participants will take part in the entire study (i.e., world-wide)?*	3000
B. *How many participants will take part at institutions covered by this Research Ethics Approval?*	3000
7.3. Principal Investigator and Research Team Experience	The principal investigators and co-investigators have full research qualifications and have managed many community-based PA and HE research projects with young children. They offer expertise in implementation science, PA and HE measurement, policy assessment, childcare and school-based interventions and knowledge translation, all of which support success of the project.
**8. Confidentiality - Behavioural Study** [View Form]	
8.1. Security of Data During the Course of the Study	Hard copies of the data (i.e. surveys, data collection sheets, interview notes), video files and audio files will be stored in a locked file cabinet in a locked room at UVic/UBC. Electronic files of de-identified data will be stored on a secure network drive at UVic/UBC which is accessible only to the investigators and research team. Electronic surveys will be kept in Qualtrics.<br><br>The parent, child and educator data will be be collected in REDCap in a different project (i.e.. REDCap “Childcare Providers" and "RedCap "Childcare Families"). As the ID, email address, and child first name need to be in the “Childcare Families" , we will work with the REDCap team to write a DET (data entry transfer) to securely move information from the REDCap “RedCap "Childcare Contact" project into the Redcap "Childcare Providers" and "Childcare Families" data projects. We have used this strategy to separate the contact information in previous REB approved study. The email addresses are used to send the questionnaire and other study reminders. The children's first names will be used because it is likely that multiple children from the same family will attend the same childcare centre.<br><br>The videos of children performing FMS activities will be used to assess the FMS skills after the fact. The audiotapes of the interviews will be used to transcribe the interviews so that they can be themed. The data (from surveys, interviews, observations, accelerometers and FMS testing) will be used to prepare a report for stakeholders, and all participating groups upon request. Academic papers may be published. Presentations may be made to stakeholders and at academic conferences.<br><br>After the workshops, the de-identified surveys and consent forms are separated by the regional trainers. The surveys are mailed to the YMCA of Greater Vancouver where they are transferred to Jennifer McConnell-Nzunga at UBC/BCCHR offices in person during bi-weekly meetings. The surveys have to be processed at UBC because they have the OMR survey scanning software used to extract the data from the surveys. The surveys do not contain names just ID codes.<br>The consents are mailed to UVic by the workshop trainers where they are scanned and uploaded to the password protected UVic Research Netdrive.<br><br>The paper-based workshop surveys are currently being stored and managed at UBC/BCCHR. The paper surveys are scanned first through Remark Office OMR software to read the responses into a de-identified dataset that is saved on the UVic Research Netdrive, and second they are scanned and saved as PDFs to the UVic Research NetDrive. Then we will securely shred the paper copies. We are expecting 5,000 participants in the Appetite to Play Workshops and the pre-workshop survey is 4 pages and the post workshop survey is 6 pages resulting in up to 50,000 pages of survey data to store.<br><br>We wish to have identifiable data stored on the UBC secure servers, specifically the place of work of participants who completed an Appetite to Play Workshop in person or online (Groups 8 and 9) who provided this information.

We previously used UBC Qualtics for the EY (Early Yearys) surveys. To streamline the RCT data collection as part of Aim 4 all data is now collected in a single platform - REDCap. To ensure that the data remains protected, we will follow a similar previously approved strategy in which we will collect the surveys data in a project that is independent of the project that collects the contact information and consent.

As part of the evaluation of the ATP+ RCT intervention, we will collect web-analytic data. The intervention is delivered via the UBC Canvas Catalogue site (for the centers recruited before August 23, 2023) and via Pathverse app for the new centers recruited after August 21, 2023). All participants recruited before August 23, 2023, will get access to the ATP+ intervention via Canvas even if they access the intervention after August 23, 2023. All participants recruited after August 23, 2023, will get access to the ATP+ intervention via Pathverse app. Thus, there may be a period of about 3 months in which both intervention platforms are available, but each participant would only have access to one platform, depending on their recruitment date.
The extraction of data from CANVAS and Pathverse includes the modules the participants have completed, date of completion, and user name (in Pathverse, this is not an identifier but an id generated by us and based on participant id number to connect the usability data with REDCap. In CANVAS, this is participant email but after this merging process, the email will be immediately eliminated.

The Pathverse platform was developed by researchers at the University of Victoria, app-analytics data is stored in Canada, and process for storing and securing the data has been approved by Canadian REB (see detailed privacy policy included in attachment “2022-04-05_Pathverse Privacy Policy.docx”). This platform will be used for both ATP+ and Parenting RCTs.
Participants will enter an ID number into the app and this is the only code that will allow us to connect the app-analytics data from Pathverse with the data we collect in REDCap. Lesson completion data collected through Pathverse will be stored on Amazon servers in Canada. Although these data are stored in Canadian servers, Amazon is a US-owned and operated company. As such, there is a possibility that the app usability data may be accessed without our knowledge or consent by the U.S. government, in compliance with the U.S. Freedom Act. Data will be deleted from these servers after three years. Goal setting data that is added by the user is stored on the device and is strictly for display purposes. Therefore, uninstalling the app will permanently delete all data related to them.

Sub-study (AIM 4) Parenting RCT - Consent and contact information for subject who volunteer to sign up for the parenting sub-study will have their identifiers and contact information stored into REDCAP. All data will be collected in REDCap except for the app-analytics data and the project data will be stored in an independent project (a procedure we have used and that has been approved in the past by the REB). For the parenting sub-study the intervention will be delivered by the Pathverse platform.

We will use Twillo to send participants the REDCap link via text messages to complete the Ecological Momentary Assessment (EMA). REDCap sends the text messages through this third-party web service (Twilio (www.twilio.com)) but no data is collected via Twilio. All text messages will be routed through Twilio's Canadian servers. However, REDCap ensures that text message transcriptions do not stay in Twilio's logs but are removed shortly after being completed. This is done for security and privacy concerns (e.g., HIPAA), in which participants' phone numbers do not get permanently logged on Twilio's servers but instead remain securely in REDCaP. Thus, Twillo is an add-on to RedCap that allows us to send text message notifications to participants. We need to send text messages for this study because we will collect intensive longitudinal data on a daily basis for a period of 14-days at baseline and at 10 weeks. Given the importance of our participants to complete the EMA check-in (short survey) right when the link is sent to them, we are using Twillo to more effectively communicate with them and ensure they see the link at the moment is sent to them as email communications do not work well to collect just-in-time data. In addition, to be able to send daily EMA survey links to participants via text message, we need to store the participant’s cellphone number in the same REDCap project where the EMA survey is stored, but no analysis dataset will contain the cellphone number or any other personal identifiers.

	(June 21, 2023 PAA) Our study needs parents to complete questionnaires at 2 time points. REDCap allows us to send the first questionnaire link using an Alert from the Consent project without saving the email in the Survey project. However, the time 2 questionnaire cannot be triggered by the completion of the first questionnaire or by using an Alert from the Consent project; it requires saving the email in the Survey project. Specifically, to be able to redirect parents to the time 2 questionnaire within the Survey project, we need to use an Automated Survey Invitation (ASI) instead of an Alert. However, it is only possible to send an ASI from the project where the questionnaire is located, which in our case is the Survey project. Thus, we require the email address to be stored in the Survey project. In this REB submission, we are requesting approval to store the participant email with the parenting survey in the REDCap Survey project. To maintain confidentiality of the data, data extraction will be done without the identifier.<br><br>(June 29, 2023 PAA) Our current data architecture in REDCap includes, among others, 2 separate projects storing the results from our short and long surveys, namely EMA survey and Full survey respectively. REB previously approved storing cellphone with EMA survey, and email with Full survey. As previously mentioned, we require storing these identifier since REDCap ASIs can only be triggered if some contact information is available (and we extensively use ASIs in our study since we collect repeated daily data for a long period of time - 14 days at baseline and 14 at follow up, in addition to full baseline and follow up surveys). In this amendment, we request to combine the 2 survey projects in a single survey project, where both cellphone and email would be stored with this single survey instead of one per survey. All remaining personal information will be stored in different REDCap project and not in the survey project. For monitoring purposes (eg, randomization, reminders, incentives), we need to know when participants have completed each assessment and how many tools have been completed or still missing. However, if the survey completion information resides in multiple projects, we would only be able to monitor participants' progress by downloading survey information from each project, merging them, running a report outside of REDCap, and uploading the merged datasets back into the monitoring project. While this would not combine survey responses with identifiers, having all survey responses in a single REDCap project would reduce error, increase efficiency, and minimize the information we download and merge, which is a continuous process for the duration of the study. Note that for monitoring purposes, we only need to extract time stamps and number of surveys completed from the survey project but no survey response data, so confidentiality of participants' responses would be maintained. Likewise, for data analysis purposes, we don't need to extract any identifiers from the survey project as participants can be linked with their participant id number protecting their anonymity.<br><br>(August 23, 2023 PAA) As requested by the REDCap team, a summary of all REDCap projects involved in both RCTs (ATP+ & Parenting) as well as the information transferred between projects is summarized in the document named "RCT(Aim4)_REDCap_v1".<br><br>(Oct 19, 2023 PAA) The REDCap team has requested additional detail in our application. For Aim 4, when participants provide their consent online, it means the consent is completed via REDCap e-consent framework. In addition, when variables are transferred via survey distribution link, the value of the variable itself is included in the link, and participants are redirected to the destination REDCap project when they click on the link. Finally, while the electronic data transferred is being programmed, we have duplicated projects in REDCap to implement alternative data transfer processes detailed in our document named RCT(Aim4)REDCap_v2.
8.2. Access to Data	Only the research team members (listed on the REB certificate) will have access to the data. They will be made aware that the information is confidential and that data security must be maintained at all times.<br>Only the principal investigator (Masse) and the research coordinator (Buckler) will have access to identifiable (childcare site of participants from workshops) data stored on both UVic and UBC secure servers.
8.3. Protection of Personal Information	Consents of Group 8 – the forms include the study ID code to be able to match the consent form with the surveys that include no other ID's. The consent with name/IDs and the workshop surveys with IDs only are only together very briefly before they are separated into 2 piles and sent to 2 different locations which minimizes the chance of them being matched

	up outside of the study.<br><br>INTERVIEWS<br>For participants in Groups 1 and 2, data will be themed and all identifying information and features will be removed or changed in quotes in any report, manuscript or presentation. Each year a new recruitment script will be sent out to all stakeholders and delivery agents (regardless of prior participation) and as such, names will not be used to re-contact initial participants in subsequent years.<br><br>SURVEYS<br>Participants in Groups 3, 4 and 5 taking part in the longitudinal survey will be given unique numeric identifiers to be used in years 2, 3 and 4 of the study. Their names and contact information will be required to re-contact them each year, however, only the researcher contacting them will use this information. When the researcher contacts them in years 2, 3 and 4, they will provide the participant with their unique code which the participant can include on the survey to keep it confidential.<br>The contact information provided by survey respondents to receive an e-gift card will not be kept with the survey data. All survey data will be de-identified.<br><br>PA/HE SUB-STUDY IN CHILDCARE FACILITIES<br>Participants in Group 6 will be given unique numeric identifiers for accelerometry and FMS testing, which will be used on all of their data. In each of the subsequent data collection periods after the initial one, the child will be identified by their code (e.g. for FMS testing, a white board will be placed in front of the camera before each consented child does the skill, and the board will say the skill name and the child's code).<br><br>Once the children are fitted with the accelerometers, it is not obvious who is wearing them because they are small and generally worn on top of the pants but under a shirt. Being videotaped during the FMS activities will not be obvious because it is difficult to tell if the camera is on or off. It will be on a tripod pointed at the activity area and turned on when a consented child is performing their activity in the area. Video recordings of children doing the FMS activities will only for analysis and will be destroyed as soon as the skills have been analyzed.<br><br>Groups 1, 2, 6, and those in 3-5 who provide their contact information in the survey, will not be anonymous. Participants in Groups 3-5 who complete the surveys without including their contact information will be anonymous. The participants will be anonymous in the dissemination of results.<br>NEW: Registration lists from workshops will be provided to the UBC PI (Masse) and research coordinator (Buckler) with participant name removed, only the location of the participant's work will be stored on UBC servers. This data will only be used to determine the impact of the workshop on improvements in policies and practices in childcare centres participating in the child participant data collection (Group 6) and relates directly to Aim 1 and 2 of the CIHR grant.<br>We will include a note on the discussion board to indicate that posting is not anonymous: "All posting are NOT anonymous, please do not reply to this post if you want to remain anonymous".<br><br>(Aim 4) In REDCap projects we store data needed to communicate with the facilities, and which include identifiers such as the contact persons of the facility, either managers or someone else, phone numbers, emails, and facility address. When each staff from the facility consents, as shown in our consent forms, we also collect identifier information (eg, full name, phone, email, gender) which is again stored in REDCap.<br><br>(Aim 4 substudy) The phone numbers are used to send EMA text messages via Twilio – the REDCap approved provider for sending text messages to participants.
8.4. Transfer of Data<br><br>*Will any data be transferred (made available) to persons or agencies outside the lead*	no

University or Health Authority?	
If yes, describe in detail what information will be released, to whom, how the data will be transferred, how and where it will be stored and what safeguards will be used to protect the identity of participants and the privacy of their data. Attach the data transfer agreement if applicable.	With respects to data collected under groups 3, 4, and 6 no data will be transferred outside of the University.
8.5. Retention and Destruction of Data	All data will be retained by Dr. Masse on UBC secure servers for a minimum of five years following the last publication, after which point it will be destroyed. Hard copies of data will be shredded. Computer files and electronic surveys will be deleted. Video tapes and audio tapes will be erased. Video recordings of children doing the FMS skills will only be used for analysis and will be destroyed as soon as the skills have been analyzed.
8.6. Future Use of Data	With respects to data collected for groups 3, 4, and 6, there are currently no plans to use the data in the future. Since continuous monitoring can be of use for gaining a greater understanding of the data, we will ensure that the data is collected in way that it can be continuously linked and participants will be informed that this is our intention.<br>Data may be posted in an online repository in line with open science policies, and consent forms reflect this.
8.7. Feedback to Participants<br><br>*Please provide information regarding your plans for communicating study results to participants. See the guidance notes for more information and respond to the bullet points as needed.*	We intend to communicate our findings to stakeholders, childcare centres that participate in our study and if appropriate to parents.

9. Documentation - Behavioural Study [View Form]

9.1. Research Proposal

Document Name	Version	Date	Password (if applicable)	
CIHR funded grant	4	Tuesday, March 4, 2025		[View]
Parenting Protocol	2	Thursday, April 27, 2023		[View]
Parenting Protocol	1	Friday, October 28, 2022		[View]
CIHR funded grant	3	Friday, October 28, 2022		[View]
Research Protocol Approved by UVic	3	Wednesday, January 22, 2020		[View]
Flow Chart with R numbers	2	Wednesday, January 22, 2020		[View]
School Lunchbox Checklist Protocol	1	Tuesday, January 14, 2020		[View]
A. Appendices List	2	Tuesday, September 10, 2019		[View]
SSHRC Funded proposal	1	Wednesday, June 13, 2018		[View]

9.2. Documentation of Consent	Document Name	Version	Date	Password (if applicable)	
	ATP interviews consent	1	Tuesday, March 4, 2025		[View]
	ATP interviews invitation	1	Tuesday, March 4, 2025		[View]
	RCT(Aim4_SubStudy)_Parent consents_v7.3	7.3	Wednesday, February 7, 2024		[View]
	RCT(Aim4)_ECE consent_v7.3	7.3	Wednesday, February 7, 2024		[View]
	RCT(Aim4_SubStudy)_Parent consents_v3	3	Wednesday, March 15, 2023		[View]
	RCT(Aim4)_ECE consent_v4	4	Wednesday, March 15, 2023		[View]
	RCT(Aim4)_invitation and consent form qualitaive_v1	2	Friday, October 28, 2022		[View]
	Time 3 EY Survey consent and invitation	2	Thursday, September 15, 2022		[View]
	Time 3 Consent and initial invitation CLEAN VERSION	1	Thursday, September 15, 2022		[View]
	Pilot ATP+ ECE consent	1	Wednesday, June 15, 2022		[View]
	Child Consent Years 1 and 2	8	Thursday, February 27, 2020		[View]
	ECE Consent	4	Thursday, February 20, 2020		[View]
	C. Stakeholder_Consent_Years 1-4	4	Tuesday, September 10, 2019		[View]
	I. Manager_Recruitment Script, Consent, Survey Link_Years 1 and 3	4	Tuesday, September 10, 2019		[View]
	N. Short Program Staff Recruitment Script, Consent, Survey Link Year 1	4	Tuesday, September 10, 2019		[View]
	E. Delivery Agent_Consent_Years 1-4	4	Tuesday, September 10, 2019		[View]
	M. Childcare Staff Recruitment Script, Consent, Survey Link Years 1 and 3	4	Tuesday, September 10, 2019		[View]
	K. Manager Recruitment Script, Consent, Survey Link Longitudinal Study	4	Tuesday, September 10, 2019		[View]
	S. SP Staff_Recruitment Script, Consent, Survey Link_Longitudinal Study_v4	4	Tuesday, September 10, 2019		[View]
	NNN. Delivery Agent Interview Consent Years 1-4	2	Tuesday, September 10, 2019		[View]
	Q. Childcare Staff Recruitment Script, Consent, Survey Link Longitudinal Study	4	Tuesday, September 10, 2019		[View]

	Document Name	Version	Date	Password (if applicable)	
	OOO. Virtual Workshop Consent	1	Wednesday, October 3, 2018		[View]
	LLL. Workshop Participant Interview Consent Form	1	Wednesday, January 31, 2018		[View]
	HHH. E-Module Consent	1	Tuesday, December 5, 2017		[View]
	VV. Workshop Participant Consent Form v2 for ethics	2	Tuesday, December 5, 2017		[View]
	I. Manager_Recruitment Script, Consent, Survey Link_Years 1 and 3_v3	3	Tuesday, March 7, 2017		[View]

9.3. Documentation of Assent	Document Name	Version	Date	Password (if applicable)	
	Child Assent Years 1 and 2	2	Wednesday, January 15, 2020		[View]

***9.4. Advertisement to Recruit Participants** (Ads, Posters, letters of initial contact, etc).*	Document Name	Version	Date	Password (if applicable)	
	RCT(Aim4_SubStudy)_parenting_advertisement_v3	3	Wednesday, August 23, 2023		[View]
	RCT(Aim4)_Advertisement to recruit_v1	1	Wednesday, January 11, 2023		[View]

9.5. Questionnaire, Questionnaire Consent Cover Letter, Tests, Interview Scripts, etc.	Document Name	Version	Date	Password (if applicable)	
	ATP interview guide	1	Thursday, March 13, 2025		[View]
	RCT(Aim4)_Interview script for parents_v3	3	Wednesday, August 23, 2023		[View]
	RCT(SubStudy)_Parenting Measures_v4	4	Thursday, April 27, 2023		[View]
	RCT(Aim4) Modified EPAO_v10	10	Thursday, April 27, 2023		[View]
	RCT(SubStudy)_Parenting Measures_v2	2	Friday, October 28, 2022		[View]
	RCT(Aim4)_Interview script for parents_v1	1	Friday, October 28, 2022		[View]
	Good Start Matters Equipment Environment Space	3	Friday, October 28, 2022		[View]
	Time 3 EY Survey	1	Thursday, September 15, 2022		[View]
	Pilot ATP+ interview	1	Wednesday, June 15, 2022		[View]
	Parent survey invitation email	2	Thursday, March 5, 2020		[View]
	Early Years survey invitation email - Observation	2	Thursday, March 5, 2020		[View]
	EY Surveys Centre collection	2	Thursday, February 20,		[View]

		2020	
Childcare Centre Screening Questions	1	Thursday, February 20, 2020	[View]
Parent Survey	1	Thursday, January 16, 2020	[View]
School Food Checklist	1	Wednesday, January 8, 2020	[View]
QQQ. Virtual Workshop Post-Survey	1	Thursday, October 3, 2019	[View]
RRR. EY Surveys_2018_19_ver_20_01172019	1	Thursday, January 17, 2019	[View]
PPP. Virtual Workshop Pre Survey	1	Wednesday, October 3, 2018	[View]
AA. Delivery Agent_Interview Schedule_Years 1-4_v2	2	Friday, March 9, 2018	[View]
JJJ. EY Participant Pre-Workshop Survey_NonECE	1	Wednesday, January 31, 2018	[View]
GGG. E-Learning Module Post-PA Survey_v2	2	Wednesday, January 31, 2018	[View]
DDD. E-Learning Module Pre-HE Survey_v2	2	Wednesday, January 31, 2018	[View]
KKK. EY Participant Post-Workshop Survey_NonECE	1	Wednesday, January 31, 2018	[View]
XX. Workshop Participant Interview Schedule_v3	3	Wednesday, January 31, 2018	[View]
FFF.E-Learning Module Post-HE Survey_v2	2	Wednesday, January 31, 2018	[View]
EEE. E-Learning Module Pre-PA Survey_V2	2	Wednesday, January 31, 2018	[View]
DD. Delivery Agent Tracking tools v2	2	Tuesday, December 5, 2017	[View]
III. 2 page non-standard ATP Post-Workshop Survey	1	Tuesday, December 5, 2017	[View]
RR. EY Trainer Pre-Workshop Surveyv3formatted	3	Tuesday, December 5, 2017	[View]
PP. EY Participant Post-Workshop Survey_v3formatted	3	Tuesday, December 5, 2017	[View]
TT. EY Trainer Post-Workshop Survey v3formatted	3	Tuesday, December 5, 2017	[View]
NN. EY Participant Pre-Workshop Survey_v3 formatted	3	Tuesday, December 5, 2017	[View]
CCC. Family Child Care Questionnaire v2 2017-05-15	2	Monday, May 15, 2017	[View]

	EE. Manager Questionnaire_Word Format	1	Tuesday, March 7, 2017		[View]
	GG. Childcare Staff Today Questionnaire_Word Format	1	Tuesday, March 7, 2017		[View]
	FF. Childcare Staff General Questionnaire_Word Format	1	Tuesday, March 7, 2017		[View]
	HH. Short Program Staff Questionnaire	1	Tuesday, March 7, 2017		[View]
	CC. Delivery Agent_Interview Transcript Review Script_Years 1-4_v1	1	Thursday, May 12, 2016		[View]
	BB. Stakeholder_Interview Transcript Review Script_Years 1-4_v1	1	Thursday, May 12, 2016		[View]
	Z. Stakeholder_Interview Schedule_Years 1-4_v1	1	Thursday, May 12, 2016		[View]
9.6. Letter of Initial Contact	**Document Name**	**Version**	**Date**	**Password (if applicable)**	
	External Email Invitation	1	Friday, December 20, 2024		[View]
	RCT(Aim4_SubStudy)_Initial Invitations_v5	5	Tuesday, February 7, 2023		[View]
	RCT(Aim4_SubStudy)_Initial Invitations_v1	1	Friday, October 28, 2022		[View]
	Pilot ATP+ invitation	1	Wednesday, June 15, 2022		[View]
	Single Site Invitation Letter	3	Thursday, February 20, 2020		[View]
	Manager Multi Site Invitation Letter	3	Thursday, February 20, 2020		[View]
	Child Recruitment Script	3	Wednesday, January 22, 2020		[View]
	OOO. Staff and Manager Interview Recruitment	1	Tuesday, July 31, 2018		[View]
	KK. Childcare Staff Sub-Study letter of information	2	Tuesday, July 31, 2018		[View]
	SSS. Manager Recruitment Postcard	1	Tuesday, July 31, 2018		[View]
	PPP. Child Care Centre Sub-Study Invitational package	1	Tuesday, July 31, 2018		[View]
	H. Manager Initial Recruitment Script Years 1 and 3 v2	2	Tuesday, July 31, 2018		[View]
	MMM. Delivery Agent Interview Invitation	1	Friday, March 9, 2018		[View]
	YY. Direct mail out letter to Group facilities (w paper survey April 2017)	1	Tuesday, April 11, 2017		[View]
	ZZ. Sample promotional scripts for Family Child Care facilities	2	Tuesday, April 11, 2017		[View]
	AAA. Family Child Care Recruitment Script and Survey Link V2	2	Tuesday, April 11, 2017		[View]
	D. Delivery Agent_Recruitment Script_Years 1-4_v1	1	Thursday, May 12, 2016		[View]
	B. Stakeholder_Recruitment Script_Years 1-4_v1	1	Thursday, May 12, 2016		[View]
	X. Child_Recruitment Script_Spring Years 1 and 2_v2	2	Tuesday, May 10, 2016		[View]

	Y. Child_Recruitment Script_Fall Year 2_v2	2	Tuesday, May 3, 2016		[View]
9.7. Other Documents	**Document Name**	**Version**	**Date**	**Password (if applicable)**	
	RCT(Aim4)_REDCap_v4	4	Friday, December 20, 2024		[View]
	RCT(Aim4)_REDCap_v3	3	Wednesday, November 22, 2023		[View]
	RCT(Aim4)_Brochure_ATP_v1	1	Wednesday, August 23, 2023		[View]
	RCT(Aim4_SubStudy)_Parenting communications_v4	4	Wednesday, August 23, 2023		[View]
	RCT(Aim4_SubStudy)_Parenting Pathverse intervention_v2	2	Thursday, April 27, 2023		[View]
	ATP+ communications_v2	2	Tuesday, February 7, 2023		[View]
	RCT(Aim4)_Interview for parents_other documents_v2	1	Friday, October 28, 2022		[View]
	RCT(SubStudy)_Parenting Intervention_v1	1	Friday, October 28, 2022		[View]
	RCT(Aim4_SubStudy)_ATP+ Presentation_v1	1	Friday, October 28, 2022		[View]
	RCT(Aim4_SubStudy)_Other Docs_v2	2	Friday, October 28, 2022		[View]
	RCT(Aim4_SubStudy)_ATP+ Presentation_v1	1	Friday, October 28, 2022		[View]
	Pathverse Privacy Policy	1	Friday, October 28, 2022		[View]
	RCT(Aim4) Safe Research Plan_v1	1	Friday, October 28, 2022		[View]
	Time 3 EY Survey other documents		Thursday, September 15, 2022		[View]
	Pilot ATP+ other documents	1	Wednesday, June 15, 2022		[View]
	ATP+ intervention	1	Wednesday, June 15, 2022		[View]
	Parents missing item reminder scripts - email and phone	1	Thursday, February 27, 2020		[View]
	Educator incentive email	1	Thursday, February 27, 2020		[View]
	Family incentive email	1	Thursday, February 27, 2020		[View]
	Educators missing item reminder scripts - email and phone	1	Thursday, February 27, 2020		[View]
	Multi Site Follow Up Call Script	2	Wednesday, January 22, 2020		[View]
	Single Site Follow Up Call Script	2	Wednesday, January 22, 2020		[View]
	Multi Site Obtaining Contact info call script	1	Friday, January 10, 2020		[View]

	Schedule Meeting Call script	1	Friday, January 10, 2020	[View]
	TTT. Invitation Multi-Age Facilities_01172019	1	Thursday, January 17, 2019	[View]
	UUU. Invitation retake_01172019	1	Thursday, January 17, 2019	[View]
	J. Manager Reminder Script Years 1 and 3	3	Friday, December 7, 2018	[View]
	BBB. Family Child Care Reminder Script_Years 1 and 3	1	Tuesday, April 11, 2017	[View]
	R. Childcare Staff_Reminder Script_Longitudinal Study_v3	3	Tuesday, March 7, 2017	[View]
	P. SP Staff_Reminder Script_Year 1_v3	3	Tuesday, March 7, 2017	[View]
	LL. Phone call reminder script	1	Tuesday, March 7, 2017	[View]
	L. Manager_Reminder Script_Longitudinal Study_v3 DO NOT USE	3	Tuesday, March 7, 2017	[View]
	MM. EY phone call followup email script	1	Tuesday, March 7, 2017	[View]
	O. Childcare Staff_Reminder Script_Years 1 and 3_v3	3	Tuesday, March 7, 2017	[View]
	T. SP Staff_Reminder Script_Longitudinal Study_v3	3	Tuesday, March 7, 2017	[View]
	JJ. TGMD-3 Skill Scoring Sheet	1	Thursday, May 12, 2016	[View]
	F. Stakeholder_Reminder Script_Years 1-4_v2	2	Thursday, May 12, 2016	[View]
	G. Delivery Agent_Reminder Script_Years 1-4_v2	2	Tuesday, May 3, 2016	[View]
9.8. Websites and Social Media				

10. Fee for Service - Behavioural Study [View Form]

How to submit<br><br>*Please indicate which of the following methods of payment will be used for this application:*	
Please wait for the invoice from the UBC Behavioural Research Ethics Board (BREB) to submit payment. The invoice will detail payment instructions and wire transfer information.<br><br>***Contact information regarding where to send the invoice.***	

12. Save Application - Human Ethics [View Form]

Print Close

Protocol version 4 (latest version approved by REB)

Notes for REB: new sections are highlighted in yellow

Protocol changes are highlighted in yellow. Change in green were added to address REB comments.

Title: A Good Start Matters: Do supportive childcare centre environments, policies and practices enhance physical activity?

Co-Principal Investigators: Louise Masse & PJ Naylor
Co-Investigators: Mariana Brussoni, Valerie Carson, Guy Faulkner, Erica Lau, Luke Wolfenden,

Lay title: A Good Start Matters: Do provincial physical activity guidelines influence childcare centre policies and practices and promote health in the early years?

Lay abstract (2000 characters): 2273
The early years of life are critical to establishing healthy behaviours. Sadly, the majority of preschool age children are not meeting both Canadian physical activity and screen time guidelines and have poor motor skills. Childcare centres are important settings as more than half of children attend childcare and spend a large proportion of their days and weeks there. Yet there are no Canadian guidelines and few provincial guidelines that govern the physical activity environment of childcare settings. In recognition of this gap, and the importance of the preschool years, the BC government and partners have developed Active Play Standards and communication and capacity-building interventions to support implementation of the Standards. Our research group is ready to take advantage of this opportunity to increase our understanding about the effect of supporting these Standards with communication and capacity-building strategies on the policies and practices of childcare providers and the health behaviours of children while in their care. In addition, we will explore the hierarchy of factors (individual, childcare settings and regional) that facilitate or hinder full implementation of the Standards. To meet these research goals, we will conduct province-wide surveys assessing the policies, practices and environments of licensed childcare providers before and after implementation of the province-wide communication and capacity-building interventions are initiated. We will also interview childcare providers in all regions of BC to gain more in-depth understanding of the factors that influence implementation of the AP Standards. We will develop an intervention to augment the existing resources to support full implementation and sustainment of the AP Standards. Following pilot testing and expert engagement of the intervention, we will evaluate the efficacy of this intervention in a sub-set of centres to assess the impact on childcare physical activity practices and child level physical activity, sedentary behaviour and motor skill competencies. The findings will inform future policies and enhance our understanding of how policy action supported by communication and capacity-building strategies influences the environment of childcare centres.

Institution paid: University of British Columbia

Project descriptors: early years, child health, childcare, physical activity, guidelines, policy action, implementation, knowledge translation, population-based interventions

Areas of research: Population Health, Health Research

Classification: 1) Population health; 2) Health Promotion

Themes: Social/Cultural/Environmental/Population Health

Suggested institutes: must choose 1, but can list up to 4: Population and Public Health, Institution of Nutrition, Metabolism and Diabetes or Child and Youth Health

Are gender considerations taken into account? Yes
Are sex considerations taken into account? Yes – sex differences in FMS

We propose to examine whether implementation of the Active Play Standards in childcare settings leads to improvements in children's physical activity and motor competencies while in care. Sex differences in physical activity do exist; however, the literature is mixed on whether these manifest during the early years or later in childhood and early in adolescence. We plan to treat sex as a covariate and have accounted for it in our sample size calculations.

In addition, as part of our field observations and qualitative analyses, we plan to examine whether providers' practices are influenced by gender norms (e.g., whether providers encourage girls and boys to participate in different activities). These in-depth analyses will be guided by Bottorf et al.'s (2011) social construction of gender framework and will begin exploration of gender norms in the childcare settings.

Budget (total amount requested):

Suggested Reviewers
Patricia Doyle - University of Calgary pdoyleba@ucalgary.ca
Stephen Kelder – US **Steven**.H.**Kelder**@uth.tmc
Stuart Trost – US s.trost@qut.edu.au
Diane Ward
Nicola Rodgers – Australia
Susie Kreimler – Europe
Jamie Mandingo
Erin Hobin – PE policy

Reviewers to not send to:
Darren Warburton
Shannon Bredin

ABSTRACT

Background: The early years are critical for establishing positive physical activity (PA) behaviours and fundamental movement skills (FMS) that track across the lifespan. Sadly, many Canadian children are not active enough to achieve health or developmental benefits. Childcare centres are focal settings for intervention, as more than half of Canadian children (5 years and younger) attend for approximately 30 hours per week. The early years are a recognized priority within the BC provincial PA strategy (Active People, Active Places). Consequently, the BC Director of Licensing released the Active Play (AP) Standard in June 2016 and the BC government is funding a $1.075M multi-component scale up strategy that integrates both communication and capacity-building interventions. We are uniquely positioned to build upon this investment and evaluate implementation of the AP Standards by childcare providers and their impact on children's health.

Purpose: The purpose of this study is to evaluate the province-wide implementation of the AP Standards by BC licensed childcare providers serving 30 months to school aged children. The **specific aims** are to:

1) Assess the impact of the AP Standards and supportive multi-component scale up strategy in changing policies of childcare facilities and practices of providers over time (minutes per day of: outdoor AP; combined indoor and outdoor AP; screen time; prolonged sitting time; and FMS activities).
2) Quantitatively and qualitatively identify the hierarchy of factors that influence implementation of the AP Standards to gain a better understanding of care provider, organizational and environmental factors that influence implementation (changes in policies and practices).
3) Develop and pilot an intervention [called Appetite to Play+ (ATP+)] to augment existing resources to support full implementation and sustainment of the AP Standards within childcare facilities.
4) Test the efficacy of ATP+ in: a) changing childcare practices (i.e., meet or exceed the AP Standard); b) improving child level outcomes (i.e., have better PA, sedentary behaviours and FMS profiles).

Methodology: This study will use a mixed methods concurrent nested design (QUANT + Qual). The quantitative component employs a prospective study design; collecting data before and 2 years after the start of the scale up intervention. In preparation for this work, our team is currently collecting the pre-implementation data (Fall-Winter 2016/17) using the Early Years (EY) surveys (N=~850). As part of this study, we plan on re-administering the EY surveys in 2018/19 and 2022/23 with projected overlap across data collection cycles (N=~425). To gain a more in-depth understanding of the factors that influence full implementation of the AP Standards, we will conduct qualitative interviews with 60 informants at 30 licensed facilities.

Prior to testing the efficacy of ATP+, the ATP+ intervention will be piloted among 3 to 5 childcare facilities to evaluate whether the strategies implemented in ATP+ support implementation of the AP standards.

Finally, to test the efficacy of ATP+ we will conduct a randomized control trial with 52 facilities – where 26 facilities will receive the intervention and the remaining will be assigned to a waitlist control group and will receive the intervention at 3-month post randomization. We will collect: a) childcare level data among 156 – 208 staff and/or managers assuming 3 to 4 staff at each facility participate in the intervention. At the childcare level, we will collect change in practices related to the AP standards; and

b) child level data among 280-416 children assuming that 5 to 8 children participate in the data collection at each site. At the child level, we will measure indoor and outdoor light and moderate-vigorous PA, sedentary behaviours (screen time and prolonged sitting) with accelerometry, GPS and observations as well as measuring FMS with a standardized tool.

Research team: Our team has over a decade of experience conducting PA research with young children with expertise in implementation science, PA measurement, policy assessment, childcare and school-based interventions and knowledge translation, all of which support success of the proposed study.

Significance: There are no Canadian guidelines and few provincial guidelines that govern the PA, sedentary behaviours and FMS environment in childcare settings. This research will inform future policy development and implementation in Canada.

1. CONCEPT

SPECIFIC AIMS and HYPOTHESES

Physical activity (PA), reduced sedentary behaviour and motor skill development in early childhood are associated with a number of developmental and health benefits.[1,2] Yet, many Canadian children are not active enough to achieve health or developmental benefits; only 15% of 3-4 year-olds and 5% of 5 year-olds meet both Canadian PA and screen time guidelines[3] and their motor skill levels are below norms.[4,5]

Early childhood represents a critical window of opportunity[6,7] when population-based strategies focused on changing policies and practices of childcare providers are essential for promoting PA and motor skill competencies in young children.[8,9] Findings from randomized controlled trials (RCTs) conducted in childcare settings support policy action and have informed best practices in childcare settings.[9-13] However, we know little about how to scale up knowledge from RCTs into population level impact as few interventions have been scaled up and fewer have leveraged ‘natural’ implementation experiments to research.[8,9] Effective scale up strategies are needed to ensure current population-based approaches targeting childcare settings positively impact child outcomes.[8,9]

In June 2016, the Director of Licensing in British Columbia (BC) released the Standard of Active Play (**AP Standards**) for licensed childcare facilities and, in parallel, the BC Ministry of Health is investing $1.075 M in an evidence-based multi-component scale up intervention to support implementation of the AP Standards. This provides a unique opportunity to capitalize on this investment and conduct a “real world” implementation trial to advance knowledge of strategies that support policy action.

In this project, **our overall objective** is to evaluate implementation and impact of the AP Standards and supporting multi-component scale up intervention, which integrates both communication and capacity-building support interventions, on BC licensed childcare providers.

Our specific **AIMS** are to:
1) Assess whether the AP Standards and supporting scale up intervention significantly change policies of licensed childcare facilities and practices of providers over time.
2) Identify the hierarchy of factors that influence implementation of the AP Standards and uptake of the multi-component scale up intervention.
3) Develop and pilot an intervention [called Appetite to Play+ (ATP+)] to augment existing resources to support full implementation and sustainment of the AP Standards within childcare facilities.
4) Test the efficacy of ATP+ in: a) changing childcare practices (i.e., meet or exceed the AP Standard); b) improving child level outcomes (i.e., have better PA, sedentary behaviours and FMS profiles).

<u>Hypothesis 1</u>: In the first 2 years, implementation of AP Standards will result in a 10% increase per year in the proportion of licensed childcare facilities that have policies and provide: ≥60 min/day of outdoor active play; 120 min/day of combined indoor and outdoor active play; daily Fundamental Movement Skills (FMS) activities, limit screen use ≤ 30 min/day and restrict sitting time to ≤ 60 min at a time.

<u>Hypothesis 2 (see Figure 1)</u>: Implementation of the AP Standards will be influenced by: ***Attributes of the Standards and intervention*** – moderated by adaptability/ triability, complexity, observability and relative advantage; ***Characteristics of providers*** – greater among those who have higher behavioural capacity, self-efficacy and commitment to trying and changing

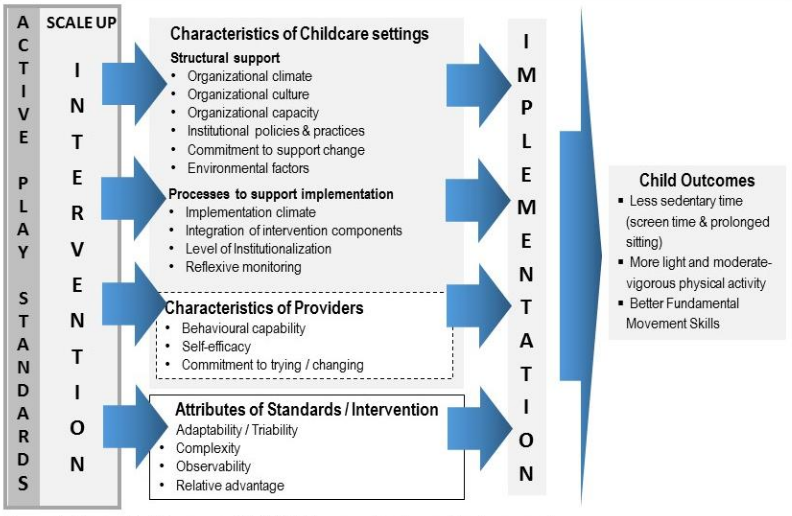

Figure 1: Theoretical framework highlighting contributions to implementation

their practices; and ***Characteristics of childcare settings*** – greater in facilities with better structural support (better organizational climate, culture, capacity and environment; supportive policies and practices; a commitment to support change) and processes to support implementation (supportive implementation climate, integration of intervention components, higher level of institutionalizations and higher reflexive monitoring).

Hypothesis 3: None stated as this is a formative aim.

Hypothesis 4: Childcare centre randomized to the ATP+ intervention will, in comparison to those assigned to the waitlist control group see the following improvements, at 3-month: a) improve their practices with respect to the AP Standards and will increase their , with increases of at least 15 minutes per day of outdoor active play, 15 minutes of indoor active play, and daily Fundamental Movement Skills (FMS) activities, compared to centres who are randomized to the control waitlist. Further, children attending those centres randomized to the intervention will demonstrate better FMS scores (overall, locomotor and object control skills), more min/day of light and moderate/vigorous PA and less sedentary time (prolonged sitting and screen time) compared to those attending control centres.

Our hypotheses are informed by our previous theoretically grounded implementation framework.[14-19] It includes four theoretical concepts from the *Diffusion of Innovations:*[20] 1) attributes of the Standards and intervention, 2) characteristics of providers, 3) linkage systems to technical support and programs, and 4) level of institutionalization (change in policies and practices). It also integrates *Social Cognitive Theory*[21,22] where childcare providers' commitment to trying/changing their practices is influenced dynamically by both characteristics of the providers and the socio-environmental context of childcare settings. From *Organizational Theory*,[23] we incorporate organizational climate, culture, and capacity as well as inter-organizational collaborations and integration of intervention components. Finally, based on Lau[24] and Damschroder,[25] we added implementation processes identified as key but currently understudied.

This project has several **novel aspects**, including: 1) expanding knowledge of strategies that support scaling up evidence-based policies and practices at the population level (an understudied priority);[8] 2) examining how system-level processes affects childcare environments and outcomes; and 3) advancing implementation science from a real world perspective.

SUB-STUDY AIMs

With post-doctoral funding from the Michael Smith Foundation for Health Research, Dr. Olivia de Jongh Gonzalez will integrate a sub-study within the Good Start Matters! study. The original Appetite to Play initiative which we are supplementing with our ATP+ program is a provincial initiative that aims to engage children in active play and ensure that they adopt healthy eating habits. Dr. de Jongh Gonzalez program of research aims complement the suite of resources developed by Appetite to Play and add a critically needed focus on parenting – an aspect that Child Health BC and the Ministry of Health intends to expand upon. To address this needs, the sub-study has the following overall objective to conduct a mixed-methods randomized controlled trial to improve co-parenting practices and child obesity-related outcomes. The specific aims of this sub-study are to:

Sub-study AIM 1: To examine how usual parenting practices and variations between parents shape children's eating and PA behaviors.

Sub-study Aim 2: To determine how intra- and inter-parent variations in parenting practices influence: a) fluctuations in child behaviors over time as well as b) overall child health behaviors.

Sub-study Aim 3: To test the efficacy of a 3-months co-parenting practices intervention in: a) improving parenting practices (primary proximal outcome), b) reducing intra- and inter-parent variations in parenting practices (exploratory outcome), and c) improving child health behaviors related to active play and healthy dietary habits (primary distal outcome).

Note about the sub-study: The expanded protocol is added into rise (filename: Protocol_SubStudy_Gonzalez_v1.doc). The sub-study aims, hypothesis, study design, eligibility of participants, sample size calculation, and data collection methods are integrated into the main study protocol. More details about the full study are in the appendix including description of the intervention,

measures, analyses, among other sections. The sections added here serve to highlight how the sub-study integrates seamlessly with the original Good Start Matters project.

BACKGROUND AND RATIONALE

More PA, reduced sedentary behaviours and FMS are critical to early child development and health.[1,2] Higher levels of PA and reduced sedentary behaviours are associated with a number of sustained developmental and health benefits in early childhood that track into adulthood, including: improved measures of adiposity,[1,2,26]cardiovascular risk factors (blood pressure and lipid profiles)[1,2,27] and bone health,[1,2,28,29] as well as motor skill acquisition,[1,2,27,30-34] and psycho-social,[1,35-37] cognitive and language development.[38-41]

PA, motor skill development and play are mutually supportive in the early years[5,42-44] and promote physical literacy "the motivation confidence, physical competence, knowledge and understanding …leading to lifelong PA engagement".[45] Exposure to a broad variety of PA opportunities supports skill acquisition[46] while motor competence promotes engagement in PA and perceptions of competence[55]. Fine and gross motor skill competence predicts a child's engagement in physically active pursuits[47-49] over the long-term.[33,47] *Thus, the early years represent a critical window of opportunity[1,2] when effective population-level strategies, if implemented, can foster motor skill development and enduring PA habits.*

Childcares are ideal settings for enhancing PA and motor skills in the early years.[40,41] In Canada, 50% of children younger than 5 years attend a licensed childcare facility for about 30 hours per week.[42] Here, early learning professionals and practitioners, physical and social environments and policies and practices shape children's PA habits and motor skills.[43] About 43% of children's PA is predicted by the childcare attended.[44] As young children do not control their environments or related choices,[45] designing supportive physical and social environments is an important shared responsibility for decision makers and caregivers. *Implementation of effective PA policy and practice interventions is key to enhancing the childcare environment and increasing PA and motor skill competencies in the early years.[46-49]*

Policy interventions are important. Recent reviews found that PA interventions conducted in childcare settings have been effective in increasing PA in the early years, [8-12] especially if those interventions included at least 30 minutes of structured PA and were delivered by skilled personnel.[8,9] This evidence supports policy action. As demonstrated in other areas (tobacco control,[50] dental health,[51] seat belt usage[52] and PA in school context[53]), policy interventions have tremendous potential to influence behavior and impact people at a relatively low cost (they are scalable).[8]

In childcare specifically, facility-level policies have positively impacted children's behaviours.[54,55] For instance, staff supervision policies related to media use was associated with reduced screen-time.[55] A US-based evaluation of the impact of PA policies for childcare facilities in New York showed that policy action combined with extensive training and technical support significantly improved childcare settings' PA practices.[56] For each additional teacher trained the association increased by 9% and the odds of complying with an additional regulation increased 3.6 times[56] for every staff person enrolled in PA training. Similarly, implementation support (from managers) enhanced the likelihood of fully implementing policies or practices in Australia.[57] *Emerging evidence suggests that policy action supported by capacity-building can increase implementation of policies in childcare settings.[14,56-58]*

Studies about impact of provincial/state childcare PA policies are rare. In 2013, 54% of provinces/territories in Canada had legislative regulations to address the outdoor play environment and PA in childcare.[59] Most regulations focused on the provision of equipment, opportunities for outdoor play and adequacy of physical spaces, except one that specified a daily PA time requirement for children.[59] In the US, most state policies have provisions for the supervision in outdoor play areas and adequate play space per child but only 36/50 specified daily outdoor PA; only 9 set specific minimum times for children to be outdoors each day.[60,61] *To date in Canada, no studies have assessed the impact of provincial level PA policies on PA within childcare settings.*

The study of implementation is timely; knowledge of effective interventions to enhance implementation of policies and programs in childcare settings is lacking.[8,13] To realize their potential impact, policies must be implemented at scale.[62] However, implementation varies widely despite best practice recommendations. For instance, only 14% of US childcare facilities provided the

recommended 120 min/day of PA, 60% had no written policy and in 20% children were seated for more than 30 minutes duration.[63,64] In an Australian study, half of childcare facilities had written policies and 40-56% provided daily FMS activities.[65] Despite progress in some countries, there is a notable gap between having supportive policies in the childcare setting and successfully implementing them.[13,66] It is not possible to develop effective standardized implementation protocols to support broad scale uptake of policy action without adequate evidence.

Current scale-up and implementation frameworks reflect the importance of strengthening organizational capacity and supporting implementation of polices and best practices,[25,62,67,68] as implementation in youth-serving organizations and childcare facilities are influenced by organizational capacity and quality of support systems.[24,57] *However, we still know little about the factors that influence implementation of policy supported by capacity-building, although such strategies are recommended in current scale-up frameworks.*[8,25,62,69,70]

ANTICIPATED OUTCOMES / SIGNIFICANCE

Understanding how to achieve wide scale implementation of policy actions at the population level is an important research and 'real world' priority.[8] Two recent bodies of evidence suggest that: 1) addressing PA and FMS in early childhood is important, and 2) that intervening with policies and practices in the childcare setting is needed.[9,13] However, we know little about how best to achieve implementation of AP Standards at scale in the childcare setting.

Our project will provide invaluable information about implementation support strategies and their impact on childcare PA policies and practices. Importantly, we aim to leverage a substantial government investment by conducting a natural experiment that examines the impact of AP Standards and supportive multi-component scale-up intervention to enhance the meagre evidence-base in this area. This will help researchers identify where implementation models and measures developed in other sectors can be modified for early years settings. Outcomes will help decision makers tailor their investments and activities to promote the outcome they hope to achieve, ideally physically literate children. In addition, our results have the potential to reshape FMS and PA habits of preschoolers; critical to early child development and health outcomes in the earlier years and later.[1,2] Finally, our findings will inform other jurisdictions in Canada who plan to employ similar population-based policy strategies.

2. APPROACH AND METHODS

STUDY DESIGN AND TIMELINE

To address Aims 1 and 2, we will use a mixed methods concurrent nested design (QUANT + Qual).

Quantitative: We will conduct a prospective study to monitor changes and factors that influence implementation of the AP Standards in childcare settings. Importantly, resources have already been committed to collect **baseline data** from all BC licensed group childcare providers in 2016/17 (using our Early Years Surveys [EY Surveys] see measures) before the start of the $1.075M multi-component scale up intervention which includes both communication and capacity-building support interventions. We will re-administer the EY Surveys at the end of 2018/19 and again in 2022/23 to all eligible licensed childcare facilities and providers in BC (see timeline).

Qualitative: We will conduct 60 informant interviews from 30 licensed childcare facilities (half in 2019~~8~~ and half in 2023~~19~~) to gain in-depth understanding of the facilitators and barriers to implementation.

Given the pandemic, we changed the design for Aim 3 and added Aim 4. As the initial AP Standard support resources developed by Child Health BC – the Appetite to Play (ATP) – were no longer providing in-person training, our ability to examine the effect of the implementation of this initiative on childcare-level and child-level outcomes became limited. As such, our team developed the Appetite to Play+ (ATP+) program to augment the original ATP programs and address the weaknesses our team identified in the evaluation of the original ATP program (e.g., Tugault-Lafleur et al, submitted found that centre did not utilize processes for sustaining change over time). The ATP+ program features a series of online courses and regular virtual support for childcare educators and managers to facilitate change in processes and practices to institutionalize the AP Standards over time. We now have the opportunity to evaluate the efficacy of the ATP+ intervention through a randomized controlled trial and

given our involvement with the team who is managing the ATP initiative, we will be in a position to implement this program should the results of this study support its wide use.

We plan to first pilot the ATP+ program with three to five childcare centres by conducting qualitative interviews to gather feedback from educators and managers from those sites. Following, this initial round of review, 52 childcare centres within the Lower Mainland will be randomized to ATP+ or randomized to the waitlist control ATP+ to collect: a) childcare level data among 156 – 208 staff and/or managers assuming 3 to 4 staff at each facility participate in the intervention. At the childcare level, we will collect change in practices related to the AP standards; and b) child level data among 280-416 children assuming that 5 to 8 children participate in the data collection at each site. The data collection will occur over (2022/2023). Using permuted block randomization, centres will be randomized to receive the intervention or to a waitlist control. Measures will be assessed at baseline and 3 months. The control group will receive the ATP+ intervention at 3 months ~~and will be assessed at baseline, 3-month, and 6-month~~. Note: this design will allow our team to test our original hypothesis as well as answer additional questions (test the efficacy of ATP+ program).

Overview of sub-study design and how it is integrated into Aim 4 of the original study

As detailed in AIM 4, The Good Start Matters! study randomizes 52 childcare centers as follow: half receive the Appetite to Play+ (ATP+) intervention after they complete the baseline assessment, and the other half are waitlisted to receive the ATP+ intervention at 3-month. As part of the ATP+ RCT, data are collected at the childcare level (~156 childcare providers – 3 per centers) and at the child level (~280-416 children – 5-8 children per centers). The SMARTER co-parenting intervention will be integrated into the original ATP+ RCT as follow: a) the food co-parenting practice intervention will be offered to 118 families (236 parents/legal guardian/primary caregivers) whose facilities are waitlisted to receive the ATP+ intervention at baseline, and b) the physical activity co-parenting practice intervention will be offered at 3-month to parents whose facilities received the ATP+ intervention at baseline. As the ATP+ intervention intervenes at the childcare level (i.e., focuses on changing practices related to the Active Play provincial guidelines), it does NOT affect the co-parenting interventions, which means that parents randomized into each arm of the study can serve as controls for this post-doctoral sub-study.

OVERVIEW OF MULTI-COMPONENT SCALE-UP INTERVENTION / AP STANDARDS

As shown in Figure 2, the BC childcare multi-component scale up intervention is guided broadly by:

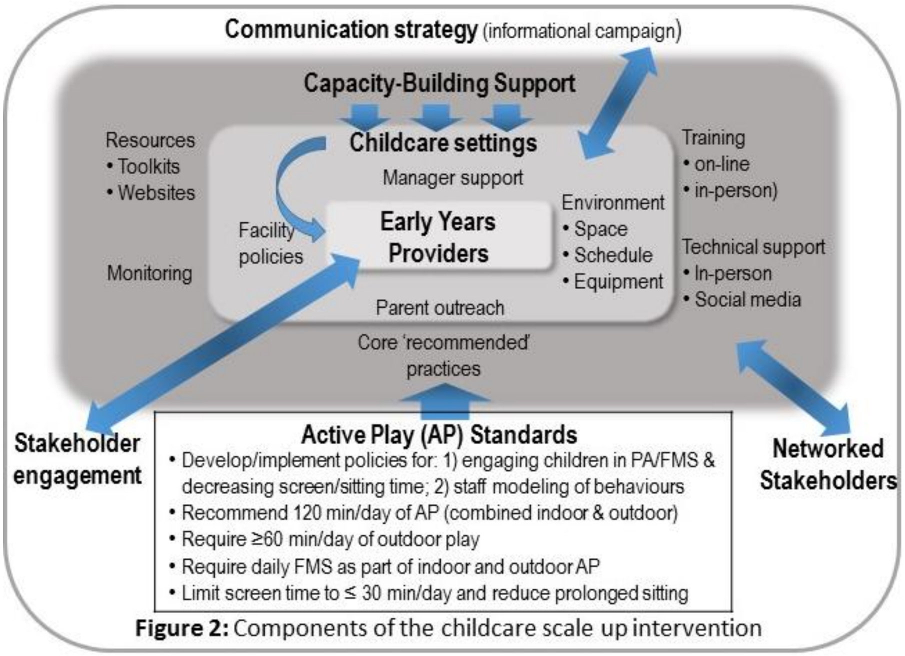

Figure 2: Components of the childcare scale up intervention

1) Socio-ecological theory[71] at the macro/systems' level, policies, networked stakeholders, communication (i.e., informational campaign promoting the AP Standards) and a capacity-building support interventions. These elements are integrated to facilitate change at the meso and micro levels – meaning changes at the facility and provider levels.[72] Ultimately, the scale up intervention aims to change organizational infrastructure and childcare providers' practices to influence PA and FMS competence.

2) Simmons'[67] scale up and Fixsen's[69,70] implementation framework core elements are integrated: coordinated efforts and messaging (aligning resources with AP Standards), linked networks (provincial stakeholders, regional licensing officers council and local childcare and PA agencies), early engagement

and input from childcare providers (through extensive consultations and involvement of an Early Years PA advisory team), promoting adaptability (choice-based resources toolkits), providing training (on-line and in-person) and technical support (site visits, proactive social media and regional experts), feedback mechanisms, and integrating delivery within existing channels (childcare resource and referral agencies, Head start, regional health authorities, local recreation and sport organizations).

Broadly, the ATP intervention is a combination of policy action with supportive dissemination strategies. Within the broader scale up intervention, the communication and capacity-building support interventions , have been designed collaboratively by Child Health BC in partnership with stakeholders and the Early Years PA Advisory Committee and researchers. The capacity building support intervention includes: web-site support, resource toolkits, pro-active social media, decentralized technical support using a train the trainer and regionalized approach, fixed guidelines but adaptable toolkits and monitoring through licensing visits and self-audit tools. The intervention strategies in these components are evidence-based,[8,13,62,70,73] and have gone through an extensive vetting process (through focus groups and surveys of 269 childcare providers).

ATP+ is informed by Warrick's (2009) implementation framework to help managers and educators implement best practices for the AP Standards. The ATP+ offers skill building professional development courses, resources and tools through template forms, assignments and feedback, and ongoing virtual support and connection to a community of practice of early years educators. Developing childcare level policies that guide the use of best practices for AP Standards provides the initial step needed to change practices, though we know that this is not enough. **(**Flaspohler et al., 2012; Mâsse et al, 2012; Wolfenden et al., 2020**)** The ATP+ thus builds upon the ATP to offer a capacity building framework for continuous monitoring and evaluation to facilitate operationally institutionalizing the AP Standards for sustainment. (Mâsse, McKay, Valente, Brant, Naylor AJPM, 2012).

PARTICIPANTS

This study targets group childcare facilities that provide care for children from infancy to school age (averaging 32 children per facilities) and account for 87% of the children in licensed care in BC. In June 2015, there were 3,044 licensed group childcare providers that provided care to 96,478 children across four service delivery areas (see below).

**Service delivery area**	# providers	# children
Interior (Kootenays, Okanagan, Thompson/Cariboo/Shuswap)	412	14,069
Coast/Fraser (E/N/S Fraser, Vancouver/Richmond, Coast/North Shore)	1,949	57,817
Vancouver Island (S/N Vancouver Island)	499	18,839
North (Northwest, North Central, Northeast)	184	5,753

AIMS 1 and 2: ELIGIBILITY / SAMPLE SIZE / DATA COLLECTION /MEASURES

Eligibility (AIMS 1 & 2): BC licenced childcare providers caring for children aged 30 months to 5 years are eligible. About 68% of the 3,044 licensed group childcare providers meet this criterion; 2,070 eligible facilities. We anticipate that 850 licensed childcare providers will complete the EY surveys in 2016; based on previous surveys.[74,75] We will conduct surveys again in 2017/18 and 2018/19 (see timeline) recruiting from the same eligibility pool to document implementation over time. Based on our previous work, we project a 50% overlap between years (N=450)[74] – meeting our sample requirement. Participation will be voluntary, so we will use post-stratification weights (based on census data) to weigh the sample and adjust for bias associated with voluntary participation.

Sample Size (AIMS 1 & 2): *Quantitative* - Using the most stringent requirement, we require pre and post-data from 368 childcare facilities to have 80% power to detect a 10% difference in the proportions of childcare facilities that significantly improve their policies and practices. This assumes the proportion at baseline is 0.35 using an α=.05 and 2-sided test (sampling increased to allow for stratified analyses as differences may exists [e.g., by low versus moderate/high income neighborhood]. We further adjusted the sample size to account for the design effect [DEFF= 1+ $\delta(n_p\text{-}1)$, where n_p = average number of providers per facility and δ = intra-facilities correlation]. Assuming an average of 2 providers per facility and an intra-facility correlation of 0.10 resulted in a DEFF = 1.10. This increased the sample size to

N=405 facilities with data in both 2016/17 and 2018/19 (collected with SSHRC funding) as well as 2016/17 and 2022/23.

Qualitative (AIM 2): Based on our previous research and recommendations for reaching theoretical saturation,[76] we estimate a sample size of 15 childcare facilities with 2 informants resulting in 30 interviews in each cycle of data collection 2017/18 and 2018/19 (total of 60 informant interviews).

Data Collection (AIMS 1 & 2): *Quantitative* - In both the 2018/19 (collected with SSHRC funding) and 2022/23 survey waves, eligible childcare facilities will be sent a preliminary notice which will be followed by an invitational package detailing data collection procedures which includes: informed consent and a link to the online EY surveys (administrator/staff survey). Administrators or assistants will forward invitational emails to their staff. Those who agree to participate will receive an email link to the staff EY surveys. Research staff will be emailing and calling administrators and their assistants to follow-up and ensure an acceptable response rate. Administrators and staff surveys take about 20 minutes to complete on-line. Staff surveys include self-report of typical and "previous day" practices. All participants will be compensated.

Qualitative (AIM 2): Facilities that take part in the EY surveys will be purposely selected to ensure variability in implementation of the AP Standards and representation from each service delivery region. We will conduct semi-structured interviews with at least 1 administrator and 1 staff.

Quantitative measures (EY-Surveys AIMS 1 & 2): We will collect information from both administrators and staff using an enhanced version of our EY follow-up surveys to assess implementation and factors that influence implementation (see list in Figure 1).

- **Implementation of AP Standards (Policies and Practices):** Our EY Surveys adapted the reliable childcare self-report Environment and Policy Assessment and Observation tool (EPAO-SR)[77] and Dodds' items (Intraclass correlation ICC from .55 to .93).[78] The "administrator EY survey" asks about the written or unwritten PA policies and the "staff EY survey" asks about typical and previous day practices related to min/day of indoor and outdoor play time and un-facilitated and facilitated play that incorporates FMS activities.
- **Attributes of AP Standards and intervention:** *Adaptability/ triability, complexity, observability, and relative advantage* measured with our 8-item scales modified for the childcare setting (α=.92 & supported by factor analysis[79]).
- **Characteristics of providers:** *Behavioural capability:* New items measuring providers' knowledge and skills. *Self-efficacy:* adapting our 8-item scale to assess confidence in implementing the AP Standards and using components of the scale up intervention, dedicating time to meet expectations and ability to engage children in activities. (α=.86 supported by factor analysis).[79] *Commitment to trying / changing practices:* New items assessing whether they are implementing and (if not) could in the next 3 months implement the AP Standards by: providing more than 60 min/day of outdoor time, 120 min/day of active play and daily FMS activities and by limiting screen time to 30 min/day or less and limiting prolonged sitting to 60 min at a time.
- **Characteristics of childcare settings – Structural Support:** *Organizational climate:* Using a 10-item semantic differential scale based on Hoy's measure (collegial, supportive, conciliatory, friendly, warm, open, welcoming, accepting of change, accommodating, trusting) (α=0.94).[80-83] *Organizational culture:* New items assessing whether innovation and change is encouraged/rewarded. *Organizational capacity:* structural resources for implementation [e.g., equipment, space, staff] and *Institutional policies and practices* with the EPAO-SR tool.[77] *Commitment to support change:* New items assessing both leadership support for changing policies and practices and organizational readiness (not open, hesitant, planning, and taken steps to make changes; partially implementing and fully implementing the changes). *Environmental factors:* New items measuring organizational size and SES of the community.
- **Characteristics of childcare settings – Processes to support implementation**: *Implementation climate:* New items assessing tension to change, compatibility with role and relative priority to change. *Integration of intervention components*: a) attended training (Yes/No item); b) links to provincial support modeled after the Alberta Heart Health project 2004 linkage questions[84] based on Valente's[85] and Robinson's[86] connectedness questions; and c) use of provincial resources/programs modeled after Steckler et al.'s[87] current use items to classify providers into 5 levels of users (non-

users to high users). *Level of Institutionalization*: EPAO-SR tool[77] to assess change in policies and practices and new items for plans to maintain change assessing sustainability (retraining of new staff). *Reflexive monitoring:* New items assessing the extent to which as a group they have institutionalized processes for evaluating their implementation efforts.

Qualitative semi-structured interviews (AIM 2): We will develop guiding questions based on our theoretical framework (see Figure 1) to probe about the factors that enhance or hinder implementation of the AP Standards. Interviews will be digitally recorded and take about 45 to 60 minutes.

AIMS 3&4: ELIGIBILITY / SAMPLE SIZE / DATA COLLECTION / MEASURES

Eligibility (AIMS 3 &4): Our primary sampling frame will be the childcare facilities that completed the 2022/23 EY Surveys. We will pilot our ATP+ intervention with three childcare centres within Vancouver in the summer 2022.

Given the intense nature of Aim 4 and the cost of collecting onsite childcare-level and child-level data, we will recruit 52 facilities from within the greater metropolitan area of Vancouver and Victoria. In addition, facilities that provide care to at least 20 children (30 months to 5 year) will be targeted.

Sample size

AIM 3: Part 1 (Developmental phase): 3 to 5 childcare provider sites will be used to evaluate the functionality of the ATP+ program. Completed as part of the development of the program. **Part 2 (Evaluation phase).** A total of 20 childcare educators and 10 managers who had access to the app as part of the randomized controlled trial (specifically those assigned to the intervention group) will be invited to participate in the interviews. A quota sampling approach will be used to ensure representation of both app users and non-users, allowing for an examination of perceived utility as well as barriers to use.

AIM 4: *Childcare outcomes*: To detect an increase in Active Play practices of 15 min/day at 3-month between the ATP+ group versus the control group with 80% power and at an alpha of 0.05 requires 62 childcare providers per groups. Assuming that 3 childcare providers participate in the intervention and an intra-facility correlation of 0.03 (based on published data[88]) resulted in a design effect (DEFF) = 1.06 which further increased the number of childcare providers to 66. Assuming a conservative attrition of 20% further increases the sample size of 79 childcare providers per groups or 26 sites per groups assuming that the study enrolls 3 childcare providers per sites. Therefore, to address the childcare level outcomes, this study requires that 52 sites be randomized to the intervention or waitlist control intervention group. ***Child outcomes***: A similar computation was conducted to estimate the number of children needed to detect a ½ increase in standard deviation for the FMS outcomes or a 15 min/day in Active Play time with 80% power at an alpha of 0.05. Assuming a 25% attrition and that 5 children per sites participate in the study at both time point would require 92 children per group or 36 sites. Given that the childcare outcomes are more stringent 52 sites will be randomized.

Data collection

AIMS 3: (Part 1: Development phase) Qualitative interviews will be conducted with childcare educators and managers following completion of the pilot ATP+ intervention online modules. Qualitative feedback will be used to adapt and revise the ATP+ intervention content and delivery prior to the full RCT study. We will use focus-groups and/or individual interviews to collect the qualitative data depending on the preferences of the facility. The interviews will be conducted online and will done in Zoom using the UBC campus-wide login in Zoom. To safeguard the identity of the participants during these interviews the following procedures will be implemented: 1) access to the session will be password protected; 2) participant will login into a waiting room and will be let in the zoom meeting by the research staff; 3) the link to the meeting will be send directly to the participants and will not be available on any public sites; 4) meetings will not be hosted on personal meeting ID; 5) the screen sharing option will not be enabled to participants; 6) the meeting will be locked once it starts so that no

other participants can enter the meeting; and 7) the video function will be disabled to maintain the participant privacy. At the invitation stage, participants will be instructed to use an alternate name in their log-in ID as a way to protect their identity and to turn off the video function. Participants will be notified when the recoding is going to start in zoom and their consent to having the conversation recorded before the interview begin. (**Part 2: Evaluation phase**) Similar to part 1, qualitative interviews will be conducted with educators and managers after they used and/or had access to the ATP+ app for a period of 3-month. In the ATP+ consent that participants completed, participants were already informed that a subsample will be invited to qualitative interviews, so this new consent is for them to enroll in the interview. Prior to inviting any participants to the interview, we will assess whether the participants were assigned to the intervention condition receiving access to the ATP+ app at baseline, as this will determine their eligibility. All qualitative interviews will be conducted online using Zoom following similar processes as discussed in Part 1. Multiple modalities will be employed to invite educators and managers to engage in these interviews, including an email and/or printed letter sent to eligible educators and managers. Research staff will follow-up with either emails or calls to provide more information about the interviews and assess educators and managers' interest in participating in this supplemental study. The emails/letters will be sent 2-3 weeks prior to the 3-month follow-up to allow for enough time for participants to review the consent form before the research team goes to the facility again. Interested participants will have the option to enroll in the interviews by completing the paper consent form or by consenting online via REDCap e-consent framework, which will provide the same detailed information as that shown in the printed consent. Eligible educators and managers who have already completed their follow-up assessments will also be invited to provide insights about the ATP+ program via emails and/or phone calls to discuss their interest in participating in the interview. If interested, they will be directed to the REDCap e-consent project for them to enroll online via REDCap e-consent framework.

AIM 4: Facilities will receive an email or letter invitation describing the purpose of the study and inviting facility to try out the ATP+ program and to participate in the evaluation of the ATP+ program. Those who expressed an interest in the study will be invited to have a zoom meeting with our team so that we can provide the facility with more information about the study or alternatively they can access the presentation about the study on their own. Those who are interested to proceed with the study will be mailed or emailed an invitation package that includes an invitation letter and a copy of the consent. After they have had the opportunity to review everything they can move ahead to be part of the evaluation of the ATP+ program. Registration is done online. The online registration has the invitation, the consent form which participant need to review and consent to participate and then they can register. As for the recruitment of the parents, childcare providers will facilitate delivery of invitation packages (includes invitation letter and a copy of the consent form) to parents which may include having the staff giving the invitations to the parents directly, emailing the invitation, or allocating a time when one of our staff can deliver the invitation packages to parents and answer any questions they may have about enrolling their child into the ATP+ program evaluation. The invitation letter includes a an online link that parents can use to register their child. The link includes the invitation letter, the consent form which they complete online and if they consent they then register their child into the study. Children, whose parents consent for their participation, will wear an accelerometer and GPS device for 3 days from the time they arrive at the facility to departure. On those same days, staff will conduct field observations. To account for weather effects, we will ensure that each facility observation set includes at least 1 non-rainy day (no rain from 9:30–11:00 am & 1:00–3:00 pm). We will collect data in units of 2 facilities at a time to match data collection days of low and high implementers. Matching will account for our stratification variables and proximity of the facilities to ensure the 2 facilities are subject to the same inclement weather. At their last visit, staff will collect the FMS data with the Test of Gross Motor Development (TGMD-3) which takes 15-20 minutes to administer. Finally, we will re-administer a shorter version of the EY Surveys to capture changes since the last cycle of data collection.

SUB-STUDY ELIGIBILITY/SAMPLE SIZE/DATA COLLECTION

Eligibility (all aims of sub-study): In total 118 families (236 parents/legal guardians) of 30 months to 5-year-old children whose facilities have expressed an interest to receive the ATP+ training will be invited to enroll in the evaluation of the SMARTER co-parenting app. Eligibility include being: 1) the

parent/legal guardian/primary caregiver of a 30 months to 5-year-old children; 2) be fluent in English both orally and in writing; 3) having one participating adults who has the primary custody of the participating child; and 4) be in a family where two parents/legal guardians/primary caregivers are willing to try out the SMARTER co-parenting app and be involved in its evaluation. A single parent / legal guardian can still enroll in the evaluation of the SMARTER co-parenting app by enlisting someone they share caregiving responsibilities (e.g., grand-parent, close friend, etc..) meaning interact with their child often. Only two parents / legal guardians /primary caregivers per children can enroll in the evaluation of the SMARTER co-parenting app.

Sample size (all aims of sub-study): Sub-study AIM 3a primary proximal outcomes (parenting practices) were used for the sample size computation for the sub-study. The RCT co-parenting practice intervention will have 80% power at an alpha of 0.05 to detect group difference of 0.5 on a 5-point Likert scale with a SD=0.8 (meaning a 10% change) in parenting practices when the sample size of each group is 41 families (total n=82). A total of 118 families (~236 parents) will be enrolled anticipating a 30% of attrition and missing data based. The sample size calculation is based on family and not on individual family members to allow examination of the intervention on both parents and examine gender difference. Note that a sub-set of families (~10 = 20 parents or until theoretical saturation is reached) will be asked to participate into qualitative interview at baseline, 3- month, and 6-month.

Data collection: As part of the ATP+ RCT parents of facilities who agree to receive the ATP+ training are asked to indicate whether they will consent to have their child be involved into the evaluation of the ATP+ program. A sub-set of these parents who already receive an invitation to enroll their children into the evaluation of the ATP+ program will be asked to participate into an optional study the evaluation of the SMARTER co-parenting app where parents get to receive parenting support for active play or healthy dietary habits. Parents will have the option to opt out of either study or to select which aspect the family wish to get involved. Parents learn about the ATP+ program evaluation by receiving an invitation package from the research which will be distributed either by the staff at the childcare facility, a research staff stationed at the childcare facility who would handout the packages to parents at the door and can answer questions about the study, or via email sent by the childcare facility. The invitational letter or email would include an invitation letter with a copy of the consent form, parents would be asked to review the information and to discuss the content of the package as a family given that enrollment into the SMARTER co-parenting app is designed to have both parents included in the app. If the family elects to register for the study, they would complete the registration online. One parent would be asked to register themselves and the participating parents. At that point, the main parent would consent and provide information about the person who has agreed to be contacted for this study. Registration by the main parent would trigger an email to the co-parent to determine whether they wish to enroll into the evaluation of the SMARTER co-parenting app. It will be made clear that registering a partner for the intervention does not mean that the parents has to agree our invitation to enroll as participation is completely voluntary.

Measures

AIM 3: (PART 1: Development phase): The qualitative semi-structured interviews will be conducted using an interview script developed for the pilot study. Questions are designed to gather feedback regarding the content, relevance and value of the content, clarity and ease of use of the online intervention, benefit of the course assignments, and feasibility of completion of the course. **(PART 2: Evaluation phase):** The qualitative semi-structured interviews will be conducted using an interview script develop to: 1) understand how centres support active play and fundamental movement skills; 2) their ability to implement active play and fundamental movement skills activities; 3) the extent to which the ATP+ have supported their practices and changed what they do to support active play and development of fundamental movement skills, and 4) overall impression of the ATP+ app and functionality that are found to be helpful.

(AIM 4): Child's Indoor and Outdoor Sedentary, Light and Moderate-Vigorous PA will be measured using direct measurement via wearable devices as well as field observations.

- **Wearable devices***:* Children will wear two devices: 1) the AX3 ~~GT3X+~~ accelerometer (Axivity device Actigraph, Pensocola, FL) extensively validated to measure PA in children;[89-91] and 2) the GPS

forerunner 230 watch (Garmin, KS) as GPS devices have higher accuracy to detect indoor and outdoor time of preschoolers (accuracy of .89 to detect outdoor time).[92]
The AX3 accelerometer is a small (4.6cm x 3.3cm x 1.5cm; 19 grams) tri-axial monitor designed to assess human motion (0.25–2.5 Hz range) and filter non-human motion. The device will be worn on children's non-dominant wrist. Children will wear the device for 4 weekdays as it provides adequate ICC (>=.70) in preschool children.[93] Data will be collected in 15-second epochs and processed with preschoolers' cut-.[94,95] In all analyses, wear time will be entered as a covariate to adjust for variations in time spent at the childcare.
The GPS forerunner 230 watch records longitude and latitude with a 2-3 metre accuracy. The GPS device will be affixed to a belt worn under clothes. Data will be collected in 1 second epoch. Indoor and outdoor time will be assessed by computing the signal-to-noise ratio (SNR or strength of the signal), where SNR >250 has been validated to assess outdoor time with 90% accuracy.[96]
We will follow the protocol we used in our CIHR playability study (Mâsse/Brussoni), the AX3 and GPS data will be merged and processed using the GGIR processing package in the R software.

- **Field observations** will supplement the wearable tools and utilize the field component of the EPAO measure; integrating observations of active and sedentary opportunities (ICC = .67 and 1.00 respectively).[97] EPAO observational tool will be expanded to document location of the activities (indoor/outdoor), intensity of the activity (sedentary, light or moderate-vigorous), types of activities (teacher led or not, FMS activity or not) and whether practices are influenced by gender norms. Observations will yield both childcare and child indices.
- **Shorter EY Survey** will be completed at baseline and 3-month~~, and 6-month for the waitlist control group~~ to collect childcare level data regarding self-report typical and previous day practices related to min/day of indoor and outdoor play time and un-facilitated and facilitated play that incorporates FMS activities.

Fundamental Movement Skills (FMS) will be assessed with the validated Test of Gross Motor Development (TGMD-3 a norm-referenced tool for assessing 13 gross motor skills - locomotor (run, gallop, hop, leap, horizontal jump and slide) and object control (strike, dribble, kick, catch, throw, and roll a ball) (test retest r .85 to .91 and $\alpha \geq .90$). The measure takes 15-20 min per child to administer and provides standardized and percentile composite scores for the locomotor, object control, and gross motor quotient (GMQ) composites.

DATA ANALYSES

Quantitative data. To address **AIM 1**, we will use longitudinal multi-level mixed effects dichotomous and polytomous logistic regressions to model changes in policies and practices triggered by the AP Standards. These analyses will quantify implementation 1 and 2 years following our initial assessment (2016/17). A mixed effects model can account for the clustering of the data and the unbalanced nature of the dataset (facilities and providers who completed 2016/17, 2018/19 (Collected with SSHRC funding) and/or 2022/23 EY surveys). In separate analyses we will examine change in: 1) policy strength (i.e., no policy, written policy without or with specificity) and 2) practices whether they meet minimum AP Standards [≥60 min/d of outdoor active play; ≥120 min/d of active play indoor and outdoor; daily FMS, screen use ≤ 30 min/d and prolonged sitting ≤ 60 min at a time].

To address **AIM 2**, longitudinal analyses from AIM 1 will serve as the base model where all the variables hypothesized to influence change (see Figure 1) will be entered as independent variables (i.e., attributes of the AP Standards and intervention, characteristics of providers, characteristics of childcare settings include both structural support and processes to support implementation). This analysis will take into account time dependency in the independent variables. In addition, childcare demographic characteristics will be entered as covariates (e.g., census variables linked by postal code such as urbanicity, % of visible minority and % of population with post-secondary education around the facility and using childcare facilities demographic variables such as size and number of staff among others).

To address **AIM 3** **(PART 1: Developmental phase)** which will pilot the ATP+ intervention, the information in the interviews will be used to modify / improve the functionality of the ATP+. The information collected as part of these interview will be summarized. Note that a thematic analysis is not

required as the interviews are conducted as a formative evaluation and the results of these interviews will not be published.

To address **AIM 4**, we will use hierarchical mixed models that incorporate both fixed and random effects at the childcare and child level and account for design effects (clustering of children within childcares). Several analyses will be conducted using the following as dependent variables: FMS scores and percent observations of daily average (min/day) of indoor and outdoor sedentary and light and moderate-vigorous PA. The corresponding level of policy and practice variables will be used as the main independent variables as well as including child level demographic variables (age, sex) as covariates.

Qualitative analyses (AIM 2): Administrators and staff transcripts from the same facilities will be linked to enable childcare unit analyses. Techniques from constructivist grounded theory approach (see Charmaz)[98-100] will guide the initial analyses using NVivo-10 software. Our analyses will also be guided by Straus and Corbin[101] procedures and will integrate Knalf and Ayres[102] procedures to ensure the childcare unit is accounted in the analyses. Our analytical plan will first analyze the transcripts of individuals (administrators and providers individually) and then shift our focus to analyzing the childcare as a unit.[102] This sequence consists of shifting emphasis from within and across individuals to a series of constant comparative analysis within and across childcare facilities.

AIM 3 (PART 2: Evaluation phase) will evaluate the perceived utility and barriers of ATP+ in supporting active play and the development of fundamental movement skills using a thematic analysis in the NVivo-10 software.

Mixed-method data integration: Data integration will begin after we have analyzed both the quantitative and qualitative data. This will require that both the qualitative and quantitative data be systematically integrated and contrasted in both the analysis and interpretation stages with more in-depth analyses using our theoretical framework (Figure 1) as an explicit theoretical lens. In addition, we will explore whether providers' practices and child preferences for activity types are influenced by gender norms in the childcare facilities using the social construction of gender framework.[103]

CHALLENGES & MITIGATION STRATEGIES

Impact of seasonality on study outcomes. Seasonality can influence providers' practices and child outcomes. For AIMS 1 and 2, we mitigate this by assessing both previous days' practices and typical practices and ask respondents to report previous day weather (used as confounder in analyses). In addition, we standardized the time when the EY surveys are administered (collected in the same months across years). The design for AIM 3 was changed as COVID-19 make disrupted the scale-up of the ATP initiative in the province and as a result our team could not look as to whether the strategies implemented into ATP impact child level outcomes. To address this issue our team developed the ATP+ to recheck the original hypotheses of this study but we were now in a position to conduct an experimental study which resulted into a stronger design.

Delays in intervention delivery. The primary components of the scale up intervention were developed in 2015/16 and have undergone extensive pilot testing. It was a priority to ensure availability of the multi-component intervention when the AP Standards were fully enacted in June 2017. The expected start date for the scale up intervention is early 2017 but in real world trials implementation delays are common. Our data collection can easily accommodate a 6-month delay in start date and if longer we can shift our EY surveys by a year without implications to our study.

KNOWLEDGE TRANSLATION PLAN

We have strong relationships with government and stakeholders to undertake this study (see letters of support). We have translated research findings to inform policies (school PA policies Mâsse/Naylor) and to evaluate the effectiveness of provincial scale-up strategies (Action Schools!BC scaled to 1600 schools in BC Mâsse/Naylor).[15,79] In addition, Naylor serves on the Early Years PA and Healthy Eating Advisory Committee of the provincial PA Strategy and the sub-committee which informed the AP Standards and the multi-component scale up intervention. As such, ongoing knowledge exchange will occur between researchers and early years stakeholders and decision-makers. As a committee member, Naylor will ensure that evaluation is a standing item on the agenda for the Early Years advisory committee that meets quarterly. We will have a direct line of communication to discuss findings from

our evaluations. Regional reports will be prepared for licensing officers. Most importantly this research will produce tangible knowledge exchange opportunities. We have planned for wider involvement of decision makers in our study as shown in our timeline, where they provide input to our instrumentation and help us develop knowledge translation products. In addition, to formally sharing our research findings with the scientific community (through presentations and publications), we will go beyond academic "currency" to inform practice and policy by sharing findings using News Flashes, Community Reports, infographics, short videos, public and stakeholder symposia, panel discussions and presentations at the Ministry Health policy rounds.

3. EXPERTISE, EXPERIENCE AND RESOURCES

RESEARCHERS

Mâsse (PI): Professor, School of Population and Public Health, UBC, MSFHR Senior Scholar. I have >20y experience in leading an active prevention research program with expertise in PA, evaluation of population-based interventions including policy evaluations enacted at the state/provincial level and the hierarchy of factors that influence implementation of interventions and policies, and measurement of PA. Mâsse will oversee all scientific and administrative aspects of this project. (40% effort time)

Naylor (co-PI) Professor, School of Exercise Science, Physical & Health Education, UVic. Naylor has extensive expertise in implementation science, setting-based interventions (childcare, schools, recreational centres), knowledge exchange and scale up/capacity-building interventions related to PA. Naylor will co-lead recruitment, design of study instrument and data collection. (35% effort time)

Brussoni (Co-A) Associate Professor, Pediatrics and School of Population and Public Health, UBC MSFHR scholar. Expertise in active play, developing playground guidelines to stimulate active and risky play and mixed methodology. She will provide a key role in measuring environmental factors in the childcare settings that influence child outcomes (PA, sedentary behaviours and FMS). (5-20% effort)

Carson (Co-A) Assistant Professor, Faculty of Physical Education and Recreation, U of A. CIHR New Investigator Salary Awardee. She has expertise in PA /Sedentary behaviours in early years and is currently funded by CIHR to evaluate the Alberta accreditation standards that address PA for childcare settings. She will guide assessment of behaviours and inform instrumentation. (5-20% effort)

Faulkner (Co-A) Professor School of Kinesiology, UBC, CIHR-PHAC Chair in Applied Public Health with extensive experience in the evaluation of large-scale provincial and national PA interventions and policies. He will guide implementation tools and refinement of the evaluation model. (5-20% effort)

Lau (Co-A) CIHR post-doctoral fellow, School of Kinesiology UBC. Emerging expertise in implementation sciences and dissemination of interventions in youth serving settings. She will provide input on factors that influence implementation and indirectly impact child outcomes. (5-20% effort)

Temple (Co-A) Professor, School of Exercise Science, Physical & Health Education, UVic with expertise in FMS, PA, interventions in childcare settings and conducting longitudinal studies in young children. She will guide assessment of FMS, provide key input on instrumentation and intervention evaluation in childcare. (5-20% effort)

Wolfenden (Co-A) Associate Professor and Brawn Career Development Fellow, University of Newcastle Australia is an internationally-recognized implementation scientist and public health researcher with experience in conducting large-scale randomized controlled trials in community settings including a number of large implementation trials in the childcare setting aimed at changing PA of preschoolers. He will provide key input on instrumentation related to assessing policies and practices in the childcare settings as well as factors that influence implementation. (5-20% effort)

EXPERIENCE/EXPERTISE

This Project is founded on the extensive stakeholder partnerships, expertise and experiences of the research team. It extends the established research programs of the PI (Mâsse) and co-PI (Naylor), who have a strong history of collaboration and research productivity – collectively Mâsse/Naylor have been awarded over $9.5M in project funds as PI/co-PI and over $19M as co-applicants. It builds on the recognized expertise and productivity of the co-applicants (Brussoni/Carson/Faulkner/Lau/Temple/Wolfenden hold >$16M in project funds as PI). Related to this Project, we have:

- *Advanced knowledge of scale up and implementation sciences* – Mâsse/Naylor published the 1st studies which identified factors that influenced implementation of provincial initiatives after scale-up;[15,79] Mâsse/Naylor/Faulkner/Wolfenden/Temple conducted implementation studies in childcare / youth based organizations; [8,17,104-110] Mâsse/Naylor/Lau/ Wolfenden informed implementation sciences through syntheses (Cochrane review) and expert driven processes.[9,13,14,24,73,111,112]

- *Evaluated policy implementation* – Mâsse identified factors that impact policy implementation[113] – Mâsse published 1st conceptual framework for evaluating impact of state/provincial PA policies;[19] Mâsse/Naylor tracked change in state/provincial policies;[74,114] Mâsse/Naylor/Faulkner/Wolfenden assessed the impact of policies on child outcomes;[65,115-119] Wolfenden developed measured that assess policies and practices in childcare settings.[78]

- *Led other early years PA, sedentary behaviours, active play and FMS work* – Brussoni/Carson/ Faulkner contributed to PA, sedentary behaviours and active play guidelines for early years and children;[120-124] Naylor/Carson/Temple assessed prevalence of PA, sedentary behaviours and FMS in childcare[3,125-127] Naylor/Temple conducted 1st Canadian longitudinal cohort K-Gr3 that measured PA and FMS and showed object and locomotor FMS skills below norms.[7] VC conducted the 1st study in Canada that objectively measured PA and sedentary behaviours in childcare;[126] Naylor/Temple/ Wolfenden developed interventions to improve FMS and PA in childcare;[4,48,128-131] Brussoni examined environmental factors related to active play in childcare;[132,133] Naylor/Carson/Temple studied developmental and health outcomes associations with PA and FMS.[1,2,5,7,134,134]

- *Methodological expertise in:* qualitative methods (Mâsse/Naylor/Brussoni), psychometrics (Mâsse) and mixed-effects models (Mâsse/Carson/Faulkner/Wolfenden); measuring PA/sedentary behaviours (Mâsse/Naylor/Carson/Temple, Wolfenden) and FMS (Naylor/Temple).

RESOURCES

Mâsse's (nominated PI) research unit at the BC Children's Hospital Research Institute is:
- Equipped with a *Survey Laboratory* to automate collection and analysis of email, web and scannable surveys; and *Field Measurement Tools* (70 accelerometers, portable scales and stadiometers – provided in-kind with CFI funds to establish the Childhood Obesity Prevention Unit).
- Supported by a research manager (D Mount) who connects staff and trainees across research group and helps bring on-board new research staff; financial manager (M Gottenbos) who manages CIHR budgets and financial reporting; and well-established research support for ethics, grants administration, finance, communications, IMIT, facilities, Research Education, Research Development.
- Physically located in a dynamic environment that fosters collaborations and interactions among faculty, trainees and research staff; provides educational opportunities to research staff and trainees (eg, ethics application, budgeting, project management, communication skills, data management and analysis); provides travel awards for trainees ($1500/yr); has a dedicated IT research support team that offers training and support in REDCap, SPPS and STATA; and provides biostatistical services.

Naylor's (co-PI) CFI funded Chronic Disease Prevention Knowledge Exchange research unit provides:
- A dynamic environment for Faculty, research trainees and coordinators including a meeting facility equipped with presentation and video-conferencing technologies, a dedicated secure research local area network, hardware and software for on-line surveying, qualitative and quantitative analysis and analysis of accelerometry data and field measurement tools (50 accelerometers, portable scales and stadiometers, and hard drives for storing video data).
- Geographic proximity to the BC Ministries of Health and Community, Sport and Culture allowing for continuous knowledge exchange with policy-makers.

This project presents considerable opportunities for student training and mentoring of first time investigator (Lau). Trainees will also have excellent networking opportunities locally and internationally within our team and our respective networks. Trainees will be co-supervised to gain transdisciplinary knowledge to identify mechanisms or levers that accelerate change, or influence responsiveness, to new policy – an important next step to influence policies and practices of childcare settings.

Reference List

(1) Timmons BW, Leblanc AG, Carson V et al. Systematic review of physical activity and health in the early years (aged 0-4 years). *Appl Physiol Nutr Metab* 2012;37:773-792.
(2) Leblanc AG, Spence JC, Carson V et al. Systematic review of sedentary behaviour and health indicators in the early years (aged 0-4 years). *Appl Physiol Nutr Metab* 2012;37:753-772.
(3) Colley RC, Garriguet D, Adamo KB et al. Physical activity and sedentary behavior during the early years in Canada: a cross-sectional study. *Int J Behav Nutr Phys Act* 2013;10:54.
(4) Adamo KB, Wilson S, Harvey AL et al. Does Intervening in Childcare Settings Impact Fundamental Movement Skill Development? *Med Sci Sports Exerc* 2016;48:926-932.
(5) Crane JR, Naylor PJ, Cook R, Temple VA. Do Perceptions of Competence Mediate The Relationship Between Fundamental Motor Skill Proficiency and Physical Activity Levels of Children in Kindergarten? *J Phys Act Health* 2015;12:954-961.
(6) Goldfield GS, Harvey A, Grattan K, Adamo KB. Physical activity promotion in the preschool years: a critical period to intervene. *Int J Environ Res Public Health* 2012;9:1326-1342.
(7) LeGear M, Greyling L, Sloan E et al. A window of opportunity? Motor skills and perceptions of competence of children in kindergarten. *Int J Behav Nutr Phys Act* 2012;9:29.
(8) Wolfenden L, Finch M, Wyse R, Clinton-McHarg T, Yoong SL. Time to focus on implementation: the need to re-orient research on physical activity in childcare services. *Aust N Z J Public Health* 2016;40:209-210.
(9) Finch M, Jones J, Yoong S, Wiggers J, Wolfenden L. Effectiveness of centre-based childcare interventions in increasing child physical activity: a systematic review and meta-analysis for policymakers and practitioners. *Obes Rev* 2016;17:412-428.
(10) Ward DS. Physical activity in young children: the role of child care. *Med Sci Sports Exerc* 2010;42:499-501.
(11) Ward DS, Vaughn A, McWilliams C, Hales D. Interventions for increasing physical activity at child care. *Med Sci Sports Exerc* 2010;42:526-534.
(12) Kreichauf S, Wildgruber A, Krombholz H et al. Critical narrative review to identify educational strategies promoting physical activity in preschool. *Obes Rev* 2012;13 Suppl 1:96-105.
(13) Wolfenden L, Jones J, Williams CM et al. Strategies to improve the implementation of healthy eating, physical activity and obesity prevention policies, practices or programmes within childcare services. *Cochrane Database Syst Rev* 2016;10:CD011779.
(14) Naylor PJ, Nettlefold L, Race D et al. Implementation of school based physical activity interventions: a systematic review. *Prev Med* 2015;72:95-115.
(15) Naylor PJ, McKay HA, Valente M, Masse LC. A mixed-methods exploration of implementation of a comprehensive school healthy eating model one year after scale-up. *Public Health Nutr* 2016;19:924-934.
(16) Masse LC, McKay H, Valente M, Brant R, Naylor PJ. Physical activity implementation in schools: a 4-year follow-up. *Am J Prev Med* 2012;43:369-377.
(17) McKay HA, Macdonald HM, Nettlefold L, Masse LC, Day M, Naylor PJ. Action Schools! BC implementation: from efficacy to effectiveness to scale-up. *Br J Sports Med* 2015;49:210-218.
(18) Mâsse LC, Frosh MM, Chriqui JF et al. Development of a School Nutrition-Environment State Policy Classification System (SNESPCS). *American Journal of Preventive Medicine* 2007;33:S277-S291.

(19) Mâsse LC, Chriqui JF, Igoe JF et al. Development of a Physical Education-Related State Policy Classification System (PERSPCS). *American Journal of Preventive Medicine* 2007;33:S264-S276.
(20) Rogers EM. *Diffusion of Innovations*. 5th Edition ed. New York: Free Press, 2003.
(21) Bandura A. *Social foundations of thought and action: A social cognitive theory*. New Jersey: Prentice Hall, Inc., 1986.
(22) Bandura A. Health promotion by social cognitive means. *Health Educ Behav* 2004;31:143-164.
(23) Steckler A, Goodman RM, Kegler MC. Mobilizing organizations for health enhancement: Theories of organizational change. In: Glanz K, Rimer BK, Lewis BM, eds. *Health Behavior and Health Education*. 2nd ed. San Francisco: Jossey-Bass; 2002;335-360.
(24) Lau EY, Wandersman AH, Pate RR. Factors influencing implementation of youth physical activity interventions:An expert perspective. Translational Journal of the American College of Sports Medicine 1[7], 60-70. 2016.
(25) Damschroder LJ, Aron DC, Keith RE, Kirsh SR, Alexander JA, Lowery JC. Fostering implementation of health services research findings into practice: a consolidated framework for advancing implementation science. *Implement Sci* 2009;4:50.
(26) Janz KF, Kwon S, Letuchy EM et al. Sustained effect of early physical activity on body fat mass in older children. *Am J Prev Med* 2009;37:35-40.
(27) Haga M. Physical fitness in children with high motor competence is different from that in children with low motor competence. *Phys Ther* 2009;89:1089-1097.
(28) Janz KF, Gilmore JM, Levy SM, Letuchy EM, Burns TL, Beck TJ. Physical activity and femoral neck bone strength during childhood: the Iowa Bone Development Study. *Bone* 2007;41:216-222.
(29) Janz KF, Letuchy EM, Eichenberger Gilmore JM et al. Early physical activity provides sustained bone health benefits later in childhood. *Med Sci Sports Exerc* 2010;42:1072-1078.
(30) Duncan MJ, Stanley M. Functional movement is negatively associated with weight status and positively associated with physical activity in british primary school children. *J Obes* 2012;2012:697563.
(31) Graf C, Koch B, Falkowski G et al. Effects of A School-Based Intervention on BMI and Motor Abilities in Childhood. *J Sports Sci Med* 2005;4:291-299.
(32) Hardy LL, King L, Kelly B, Farrell L, Howlett S. Munch and Move: evaluation of a preschool healthy eating and movement skill program. *Int J Behav Nutr Phys Act* 2010;7:80.
(33) Barnett L, Hinkley T, Okely AD, Salmon J. Child, family and environmental correlates of children's motor skill proficiency. *J Sci Med Sport* 2013;16:332-336.
(34) Robinson LE, Stodden DF, Barnett LM et al. Motor Competence and its Effect on Positive Developmental Trajectories of Health. *Sports Med* 2015;45:1273-1284.
(35) Burdette HL, Whitaker RC. Resurrecting free play in young children: looking beyond fitness and fatness to attention, affiliation, and affect. *Arch Pediatr Adolesc Med* 2005;159:46-50.
(36) Piek JP, Baynam GB, Barrett NC. The relationship between fine and gross motor ability, self-perceptions and self-worth in children and adolescents. *Hum Mov Sci* 2006;25:65-75.
(37) Skinner RA, Piek JP. Psychosocial implications of poor motor coordination in children and adolescents. *Hum Mov Sci* 2001;20:73-94.
(38) Fedewa AL, Ahn S. The effects of physical activity and physical fitness on children's achievement and cognitive outcomes: a meta-analysis. *Res Q Exerc Sport* 2011;82:521-535.
(39) Hillman CH, Schott N. Fitness and cognitive performance in childhood. Zeitschrift Fur Sportpsychologie 20[1], 33-41. 2013.

(40) Leonard HC, Hill EL. Review: the impact of motor development on typical and atypical social cognition and language:a systematic review. Child and Adolescent Mental Health 19[3], 163-170. 2014.

(41) Carson V, Hunter S, Kuzik N et al. Systematic review of physical activity and cognitive development in early childhood. *J Sci Med Sport* 2016;19:573-578.

(42) Williams HG, Pfeiffer KA, O'Neill JR et al. Motor skill performance and physical activity in preschool children. *Obesity (Silver Spring)* 2008;16:1421-1426.

(43) Stodden D, Langendorfer S, Roberton MA. The association between motor skill competence and physical fitness in young adults. *Res Q Exerc Sport* 2009;80:223-229.

(44) Riethmuller AM, Jones R, Okely AD. Efficacy of interventions to improve motor development in young children: a systematic review. *Pediatrics* 2009;124:e782-e792.

(45) International Physical Literacy Association. Canada's Physical Literacy Consensus Statement. 2014.

(46) Hardy LL, O'Hara BJ, Rogers K, St GA, Bauman A. Contribution of organized and nonorganized activity to children's motor skills and fitness. *J Sch Health* 2014;84:690-696.

(47) Henrique RS, Re AH, Stodden DF et al. Association between sports participation, motor competence and weight status: A longitudinal study. *J Sci Med Sport* 2016;19:825-829.

(48) Temple M, Robinson JC. A systematic review of interventions to promote physical activity in the preschool setting. *J Spec Pediatr Nurs* 2014;19:274-284.

(49) Piek JP, Barrett NC, Smith LM, Rigoli D, Gasson N. Do motor skills in infancy and early childhood predict anxious and depressive symptomatology at school age? *Hum Mov Sci* 2010;29:777-786.

(50) National Cancer Institute. Evaluating ASSIST A blueprint for understanding state-level tobacco control. Stillman FA, Schmitt CL, editors. Toboacco Control Monograph No. 17. 2007. Bethesda, MD, US Department of Health and Human Services, National Institutes of Health, National Cancer Institute.

(51) Kumar JV, Moss ME. Fluorides in dental public health programs. *Dent Clin North Am* 2008;52:387-401, vii.

(52) Houston DJ, Richardson LE, Jr. Getting Americans to buckle up: the efficacy of state seat belt laws. *Accid Anal Prev* 2005;37:1114-1120.

(53) Taber DR, Chriqui JF, Perna FM, Powell LM, Slater SJ, Chaloupka FJ. Association between state physical education (PE) requirements and PE participation, physical activity, and body mass index change. *Prev Med* 2013;57:629-633.

(54) LaRowe TL, Tomayko EJ, Meinen AM, Hoiting J, Saxler C, Cullen B. Active Early: one-year policy intervention to increase physical activity among early care and education programs in Wisconsin. *BMC Public Health* 2016;16:607.

(55) Erinosho T, Hales D, Vaughn A, Mazzucca S, Ward DS. Impact of Policies on Physical Activity and Screen Time Practices in 50 Child-Care Centers in North Carolina. *J Phys Act Health* 2016;13:59-66.

(56) Kakietek J, Dunn L, O'Dell SA, Jernigan J, Kettel KL. Training and technical assistance for compliance with beverage and physical activity components of New York City's regulations for early child care centers. *Prev Chronic Dis* 2014;11:E177.

(57) Wolfenden L, Finch M, Nathan N et al. Factors associated with early childhood education and care service implementation of healthy eating and physical activity policies and practices in Australia: a cross-sectional study. *Transl Behav Med* 2015;5:327-334.

(58) Benjamin Neelon SE, Finkelstein J, Neelon B, Gillman MW. Evaluation of a Physical Activity Regulation for Child Care in Massachusetts. *Child Obes* 2016.

(59) Childcare Resource and Research Unit. Provincial/territorial requirements in regulated childcare for physical activity, outdoor space and time. 2013.

(60) Kaphingst KM, Story M. Child care as an untapped setting for obesity prevention: state child care licensing regulations related to nutrition, physical activity, and media use for preschool-aged children in the United States. *Prev Chronic Dis* 2009;6:A11.

(61) Stephens RL, Xu Y, Lesesne CA et al. Relationship between child care centers' compliance with physical activity regulations and children's physical activity, New York City, 2010. *Prev Chronic Dis* 2014;11:E179.

(62) Durlak JA, DuPre EP. Implementation matters: a review of research on the influence of implementation on program outcomes and the factors affecting implementation. *Am J Community Psychol* 2008;41:327-350.

(63) Sisson SB, Campbell JE, May KB et al. Assessment of food, nutrition, and physical activity practices in Oklahoma child-care centers. *J Acad Nutr Diet* 2012;112:1230-1240.

(64) McWilliams C, Ball SC, Benjamin SE, Hales D, Vaughn A, Ward DS. Best-practice guidelines for physical activity at child care. *Pediatrics* 2009;124:1650-1659.

(65) Wolfenden L, Neve M, Farrell L, Lecathelinais C, Sutherland R, Bell C et al. How supportive are childcare service policies and practices to child physical activity? Journal of Science and Medicine in Sport 12, e169-e170. 2010.

(66) Lessard L, Lesesne C, Kakietek J et al. Measurement of compliance with New York City's regulations on beverages, physical activity, and screen time in early child care centers. *Prev Chronic Dis* 2014;11:E183.

(67) Simmons R, Shiffman J. Scaling up health service innovations: A framework for action. In: Simmons R, Fajans P, Ghiron L, eds. *Scaling up health service delivery: From pilot innvoations to policies and programmes*. Geneva, Switzerland: World Health Organization; 2007;1-30.

(68) Damschroder LJ, Goodrich DE, Robinson CH, Fletcher CE, Lowery JC. A systematic exploration of differences in contextual factors related to implementing the MOVE! weight management program in VA: a mixed methods study. *BMC Health Serv Res* 2011;11:248.

(69) Fixsen DL, Naoom SF, Blase KA, Friedman RM, Wallace F. *Implementation Research: A Synthesis of the Literature*. University of South Florida, 2005.

(70) Fixsen DL, Blase KA, Horner R, Sugai G. Scaling up evidence-based practices in education. Brief #1. 2009. Chapel Hill, The University of North Carolina.

(71) Bronfenbrenner U. Toward an experimental ecology of human development. *American Psychologist* 1977;32:513-531.

(72) Moore GF, Moore L, Murphy S. Facilitating adherence to physical activity: exercise professionals' experiences of the National Exercise Referral Scheme in Wales: a qualitative study. *BMC Public Health* 2011;11:935.

(73) Milat AJ, Newson R, King L et al. A guide to scaling up population health interventions. *Public Health Res Pract* 2016;26:e2611604.

(74) Watts AW, Saewyc E, Naylor P.J., Mâsse LC. Impact of nutrition and physical activity policies on the school environment: Case study in British Columbia. *Int J Behav Nutr Phys Act* 2014;14:50.

(75) Naylor PJ, Temple VA. Addressing physical activity and obesity in the preschool year (0-5): A literature review and environmental scan of better practices and recommendations for an

implementation strategy in BC. Report to the Early Year PA and HE Advisory Committee, editor. 2011. Victoria (British Columbia).
(76) Morse JM. Determining sample size. *Qualitative Health Research* 2000;10:3-5.
(77) Ward DS, Mazzucca S, McWilliams C, Hales D. Use of the Environment and Policy Evaluation and Observation as a Self-Report Instrument (EPAO-SR) to measure nutrition and physical activity environments in child care settings: validity and reliability evidence. *Int J Behav Nutr Phys Act* 2015;12:124.
(78) Dodds P, Wyse R, Jones J et al. Validity of a measure to assess healthy eating and physical activity policies and practices in Australian childcare services. *BMC Public Health* 2014;14:572.
(79) Mâsse LC, McKay H, Valente M, Brant R, Naylor PJ. Physical activity implementation in schools: a 4-year follow-up. *Am J Prev Med* 2012;43:369-377.
(80) Hoy WK, Tarter CJ, Kottkamp RB. The organizational climate description questionnaire for elementary schools. *Open Schools/Healthy Schools: Measuring Organizational Climate*. Newbury Park: Sage Publications; 1991;25-45.
(81) Hoy WK, Hannum JW. Middle school climate: An empirical assessment of organizational health and student achievement. *Educational Administration Quarterly* 1997;33:290-311.
(82) Hoy WK. Measuring the Health of the School Climate: A Conceptual Framework. *NASSP bulletin* 1992;76:74.
(83) Hoy WK. The Development of the Organizational Climate Index for High Schools: Its Measure and Relationship to Faculty Trust. *High School Journal* 2002;86:38-50.
(84) Alberta Cancer Foundation. An inventory of Canadian chronic disease and population health data. 2007.
(85) Valente TW, Unger JB, Ritt-Olson A, Cen SY, Anderson JC. The interaction of curriculum type and implementation method on 1-year smoking outcomes in a school-based prevention program. *Health Educ Res* 2006;21:315-324.
(86) Robinson K, Elliott SJ, Driedger SM et al. Using linking systems to build capacity and enhance dissemination in heart health promotion: a Canadian multiple-case study. *Health Educ Res* 2005;20:499-513.
(87) Steckler A, Goodman RM, McLeroy KR, Davis S, Koch G. Measuring the diffusion of innovative health promotion programs. *Am J Health Promot* 1992;6:214-224.
(88) Murray DM, Stevens J, Hannan PJ et al. School-level intraclass correlation for physical activity in sixth grade girls. *Med Sci Sports Exerc* 2006;38:926-936.
(89) Evenson KR, Catellier DJ, Gill K, Ondrak KS, McMurray RG. Calibration of two objective measures of physical activity for children. *J Sports Sci* 2008;26:1557-1565.
(90) Trost SG, Way R, Okely AD. Predictive validity of three ActiGraph energy expenditure equations for children. *Med Sci Sports Exerc* 2006;38:380-387.
(91) Trost SG, Loprinzi PD, Moore R, Pfeiffer KA. Comparison of accelerometer cut points for predicting activity intensity in youth. *Med Sci Sports Exerc* 2011;43:1360-1368.
(92) Tandon PS, Saelens BE, Zhou C, Kerr J, Christakis DA. Indoor versus outdoor time in preschoolers at child care. *Am J Prev Med* 2013;44:85-88.
(93) Addy CL, Trilk JL, Dowda M, Byun W, Pate RR. Assessing preschool children's physical activity: how many days of accelerometry measurement. *Pediatr Exerc Sci* 2014;26:103-109.
(94) Trost SG, Fees BS, Haar SJ, Murray AD, Crowe LK. Identification and validity of accelerometer cut-points for toddlers. *Obesity (Silver Spring)* 2012;20:2317-2319.

(95) Pate RR, Almeida MJ, McIver KL, Pfeiffer KA, Dowda M. Validation and calibration of an accelerometer in preschool children. *Obesity (Silver Spring)* 2006;14:2000-2006.
(96) Kerr J, Norman GJ, Godbole S, Raab F, Demchak B, Patrick K. Validating GPS data with the PALMS system to detect different active transportation modes. *Medicine & Science in Sports & Excercise* 2012;44:S25-S29.
(97) Ward D, Hales D, Haverly K et al. An instrument to assess the obesogenic environment of child care centers. *Am J Health Behav* 2008;32:380-386.
(98) Charmaz K. *Constructing grounded theory: A practical guide through qualitative analysis*. London, England: Sage Publications, 2003.
(99) Charmaz K. Shifting the grounds: Constructivist rounded theory methods. In: Morse JM, Stern PN, Corbin J, Bowers B, Charmaz K, Clarke AE, eds. *Developing Grounded Theory: The Second Generation*. Walnut Creek: Left Coast Press; 2009;127-154.
(100) Charmaz K. *Constructing grounded theory*. 2nd edition ed. Los Angeles, CA: Sage Publications Inc., 2014.
(101) Corbin J, Strauss A. *Basics of qualitative research: Techniques and procedures for developing grounded theory*. 3rd edition ed. Thousand Oaks, CA: Sage Publications, 2008.
(102) Knalf AK, Ayres L. Managing large qualitative data sets in family research. *Jounral of Family Nursing* 1996;2:350-364.
(103) Bottorf J, Oliffe JL, Kelly MT, Chambers NA. Approaches to examining gender relations in health research. In: Greaves L, Oliffe JL, eds. *Designing and conducting gender, sex, and health research*. Thousand Oak (CA): Sage; 2011;175-188.
(104) Yoong SL, Williams CM, Finch M et al. Childcare service centers' preferences and intentions to use a web-based program to implement healthy eating and physical activity policies and practices: a cross-sectional study. *J Med Internet Res* 2015;17:e108.
(105) Finch M, Wolfenden L, Morgan PJ, Freund M, Jones J, Wiggers J. A cluster randomized trial of a multi-level intervention, delivered by service staff, to increase physical activity of children attending center-based childcare. *Prev Med* 2014;58:9-16.
(106) Finch M, Yoong SL, Thomson RJ et al. A pragmatic randomised controlled trial of an implementation intervention to increase healthy eating and physical activity-promoting policies, and practices in centre-based childcare services: study protocol. *BMJ Open* 2015;5:e006706.
(107) Naylor PJ, Macdonald HM, Zebedee JA, Reed KE, McKay HA. Lessons learned from Action Schools! BC--an 'active school' model to promote physical activity in elementary schools. *J Sci Med Sport* 2006;9:413-423.
(108) Naylor PJ, Macdonald HM, Warburton DE, Reed KE, McKay HA. An active school model to promote physical activity in elementary schools: Action Schools! BC. *Br J Sports Med* 2008;42:338-343.
(109) Naylor PJ, Temple VA. Enhancing the capacity to facilitate physical activity in home-based child care settings. *Health Promot Pract* 2013;14:30-37.
(110) Ramanathan S, Allison KR, Faulkner G, Dwyer JJ. Challenges in assessing the implementation and effectiveness of physical activity and nutrition policy interventions as natural experiments. *Health Promot Int* 2008;23:290-297.
(111) Lau EY, Saunders RP, Pate RR. Factors Influencing Implementation of a Physical Activity Intervention in Residential Children's Homes. *Prev Sci* 2016.

(112) Lau E.Y., Saunders RP, Beets WM, Cai B, Pate RR. Factors influencing implementation of a preschool-based physical activity intervention. *Health Education Research (Accepted)* 2016.
(113) Naiman DI, Leatherdale ST, Gotay C, Masse LC. School factors associated with the provision of physical education and levels of physical activity among elementary school students in Ontario. *Can J Public Health* 2015;106:e290-e296.
(114) Mâsse LC, Perna F, Agurs-Collins T, Chriqui JF. Change in school nutrition-related laws from 2003 to 2008: evidence from the school nutrition-environment state policy classification system. *Am J Public Health* 2013;103:1597-1603.
(115) Mâsse LC, de Niet JE, Watts A, Naylor PJ, Saewyc E. Associations between school food environment student's consumption and body mass index of Canadian adolescents. *International Journal of Behavioral Nutrition and Physical Activity* 2014;14:50.
(116) Perna FM, Oh A, Chriqui JF et al. The association of state law to physical education time allocation in US public schools. *Am J Public Health* 2012;102:1594-1599.
(117) Hennessy E, Agurs-Collins T, Massse LC, Moser R, Perna F. State-level school competitive food and beverage laws are associated with children's weight status. *Journal of School Health* 2014;84:609-616.
(118) Stone MR, Faulkner GE, Zeglen-Hunt L, Bonne JC. The Daily Physical Activity (DPA) policy in Ontario: is it working? an examination using accelerometry-measured physical activity data. *Can J Public Health* 2012;103:170-174.
(119) Leatherdale ST, Manske S, Faulkner G, Arbour K, Bredin C. A multi-level examination of school programs, policies and resources associated with physical activity among elementary school youth in the PLAY-ON study. *Int J Behav Nutr Phys Act* 2010;7:6.
(120) Tremblay MS, Leblanc AG, Carson V et al. Canadian Sedentary Behaviour Guidelines for the Early Years (aged 0-4 years). *Appl Physiol Nutr Metab* 2012;37:370-391.
(121) Tremblay MS, Leblanc AG, Carson V et al. Canadian Physical Activity Guidelines for the Early Years (aged 0-4 years). *Appl Physiol Nutr Metab* 2012;37:345-369.
(122) Brussoni M, Gibbons R, Gray C et al. What is the Relationship between Risky Outdoor Play and Health in Children? A Systematic Review. *Int J Environ Res Public Health* 2015;12:6423-6454.
(123) Gray C, Gibbons R, Larouche R et al. What Is the Relationship between Outdoor Time and Physical Activity, Sedentary Behaviour, and Physical Fitness in Children? A Systematic Review. *Int J Environ Res Public Health* 2015;12:6455-6474.
(124) Tremblay MS, Gray C, Babcock S et al. Position Statement on Active Outdoor Play. *Int J Environ Res Public Health* 2015;12:6475-6505.
(125) Temple VA. Child's play: Physical activity and the early years. In: Singleton E, Varpalotai A, eds. *Pedagogy in Motion: Establishing a community of inquiry for studies in human movement*. London, Ontario: The Althouse Press; 2012.
(126) Kuzik N, Clark D, Ogden N, Harber V, Carson V. Physical activity and sedentary behaviour of toddlers and preschoolers in child care centres in Alberta, Canada. *Can J Public Health* 2015;106:e178-e183.
(127) Carson V, Tremblay MS, Spence JC, Timmons BW, Janssen I. The Canadian Sedentary Behaviour Guidelines for the Early Years (zero to four years of age) and screen time among children from Kingston, Ontario. *Paediatr Child Health* 2013;18:25-28.
(128) Temple VA, O'Connor JP. Active Care. A Meaningful Movement Program for Children in Care. 2002. Melbourne, VIC.

(129) Temple VA, Preece A. Healthy Opportunities for Preschoolers. A Resource for Early Learning and Care Providers. A Reseource of LEAP BC. 2007. Vancouver, BC. 2010 Legacies Now.

(130) Temple VA, Preece A. Healthy Opportunities for Preschoolers Family Resource. A Resource of Leap BC. 2007. Vancouver, BC. 2010 Legacies Now.

(131) Bell R, Gibbons S, Temple VA. Fundamental Motor Skills: Active Start & FUNdamentals. A Handbook for Generalists and Physical Education Teachers,as well as Others Tasked with Teaching Motor Skill Development. 2008. Ottawa, ON:PHE Canada.

(132) Herrington S, Brussoni M. Beyond Physical Activity: The Importance of Play and Nature-Based Play Spaces for Children's Health and Development. *Curr Obes Rep* 2015;4:477-483.

(133) Herrington S, Brunelle S, Brussoni M. Outdoor play spaces in Canada: As if if chidlren mattered (In Press). In: Waller Tea, ed. *International handbool of outdoor play and learning*. Thousand Oak (CA): Sage; 2016.

(134) Carson V, Hunter S, Kuzik N et al. Systematic review of physical activity and cognitive development in early childhood. *J Sci Med Sport* 2016;19:573-578.

Protocol version 1 (first version approved by REB)

Title: A Good Start Matters: Do supportive childcare centre environments, policies and practices enhance physical activity?
Co-Principal Investigators: Louise Masse & PJ Naylor
Co-Investigators: Mariana Brussoni, Valerie Carson, Guy Faulkner, Erica Lau, Luke Wolfenden,

Lay title: A Good Start Matters: Do provincial physical activity guidelines influence childcare centre policies and practices and promote health in the early years?

Lay abstract (2000 characters): 1975
The early years of life are critical to establishing healthy behaviours. Sadly, the majority of preschool age children are not meeting both Canadian physical activity and screen time guidelines and have poor motor skills. Childcare centres are important settings as more than half of children attend childcare and spend a large proportion of their days and weeks there. Yet there are no Canadian guidelines and few provincial guidelines that govern the physical activity environment of childcare settings. In recognition of this gap, and the importance of the preschool years, the BC government and partners have developed Active Play Standards and communication and capacity-building interventions to support implementation of the Standards. Our research group is ready to take advantage of this opportunity to increase our understanding about the effect of supporting these Standards with communication and capacity-building strategies on the policies and practices of childcare providers and the health behaviours of children while in their care. In addition, we will explore the hierarchy of factors (individual, childcare settings and regional) that facilitate or hinder full implementation of the Standards. To meet these research goals, we will conduct province-wide surveys assessing the policies, practices and environments of licensed childcare providers before and after implementation of the province-wide communication and capacity-building interventions are initiated. We will also interview childcare providers in all regions of BC to gain more in-depth understanding of the factors that influence implementation of the AP Standards. In a sub-set of centres, we will also measure children's physical activity, sedentary behaviour and motor skill competencies. The findings will inform future policies and enhance our understanding of how policy action supported by communication and capacity-building strategies influences the environment of childcare centres.

Institution paid: University of British Columbia

Project descriptors: early years, child health, childcare, physical activity, guidelines, policy action, implementation, knowledge translation, population-based interventions

Areas of research: Population Health, Health Research

Classification: 1) Population health; 2) Health Promotion

Themes: Social/Cultural/Environmental/Population Health

Suggested institutes: must choose 1, but can list up to 4: Population and Public Health, Institution of Nutrition, Metabolism and Diabetes or Child and Youth Health

Are gender considerations taken into account? Yes
Are sex considerations taken into account? Yes – sex differences in FMS

We propose to examine whether implementation of the Active Play Standards in childcare settings leads to improvements in children's physical activity and motor competencies while in care. Sex differences in physical activity do exist; however, the literature is mixed on whether these manifest during the early years or later in childhood and early in adolescence. We plan to treat sex as a covariate and have accounted for it in our sample size calculations.
In addition, as part of our field observations and qualitative analyses, we plan to examine whether providers' practices are influenced by gender norms (e.g., whether providers encourage girls and boys to

participate in different activities). These in-depth analyses will be guided by Bottorf et al.'s (2011) social construction of gender framework and will begin exploration of gender norms in the childcare settings.

Budget (total amount requested):

Suggested Reviewers
Patricia Doyle - University of Calgary pdoyleba@ucalgary.ca
Stephen Kelder – US **Steven**.H.**Kelder**@uth.tmc
Stuart Trost – US s.trost@qut.edu.au
Diane Ward
Nicola Rodgers – Australia
Susie Kreimler – Europe
Jamie Mandingo
Erin Hobin – PE policy

Reviewers to not send to:
Darren Warburton
Shannon Bredin

ABSTRACT 3500 / 3497

Background: The early years are critical for establishing positive physical activity (PA) behaviours and fundamental movement skills (FMS) that track across the lifespan. Sadly, many Canadian children are not active enough to achieve health or developmental benefits. Childcare centres are focal settings for intervention, as more than half of Canadian children (5 years and younger) attend for approximately 30 hours per week. The early years are a recognized priority within the BC provincial PA strategy (Active People, Active Places). Consequently, the BC Director of Licensing released the Active Play (AP) Standard in June 2016 and the BC government is funding a $1.075M multi-component scale up strategy that integrates both communication and capacity-building interventions. We are uniquely positioned to build upon this investment and evaluate implementation of the AP Standards by childcare providers and their impact on children's health.

Purpose: The purpose of this study is to evaluate the province-wide implementation of the AP Standards by BC licensed childcare providers serving 30 months to school aged children. The **specific aims** are to:

1) Assess the impact of the AP Standards and supportive multi-component scale up strategy in changing policies of childcare facilities and practices of providers over time (minutes per day of: outdoor AP; combined indoor and outdoor AP; screen time; prolonged sitting time; and FMS activities).
2) Quantitatively and qualitatively identify the hierarchy of factors that influence implementation of the AP Standards to gain a better understanding of care provider, organizational and environmental factors that influence implementation (changes in policies and practices).
3) Examine whether children in childcare settings that meet or exceed the AP Standard have better PA, sedentary behaviours and FMS profiles.

Methodology: This study will use a mixed methods concurrent nested design (QUANT + Qual). The quantitative component employs a prospective study design; collecting data before and 2 years after the start of the scale up intervention. In preparation for this work, our team is currently collecting the pre-implementation data (Fall-Winter 2016/17) using the Early Years (EY) surveys (N=~850). As part of this study, we plan on re-administering the EY surveys in 2017/18 and 2018/19 with projected overlap across data collection cycles (N=~425). To gain a more in-depth understanding of the factors that influence full implementation of the AP Standards, we will conduct qualitative interviews with 60 informants at 30 licensed facilities. Finally, we plan on collecting child level data among 650 preschoolers from 65 facilities stratified by level of implementation of the AP Standards; measuring indoor and outdoor light and moderate-vigorous PA, sedentary behaviours (screen time and prolonged sitting) with accelerometry, GPS and observations as well as measuring FMS with a standardized tool.

Research team: Our team has over a decade of experience conducting PA research with young children with expertise in implementation science, PA measurement, policy assessment, childcare and school-based interventions and knowledge translation, all of which support success of the proposed study.

Significance: There are no Canadian guidelines and few provincial guidelines that govern the PA, sedentary behaviours and FMS environment in childcare settings. This research will inform future policy development and implementation in Canada.

1. CONCEPT

SPECIFIC AIMS and HYPOTHESES

Physical activity (PA), reduced sedentary behaviour and motor skill development in early childhood are associated with a number of developmental and health benefits.[1,2] Yet, many Canadian children are not active enough to achieve health or developmental benefits; only 15% of 3-4 year olds and 5% of 5 year olds meet both Canadian PA and screen time guidelines[3] and their motor skill levels are below norms.[4,5]

Early childhood represents a critical window of opportunity[6,7] when population-based strategies focused on changing policies and practices of childcare providers are essential for promoting PA and motor skill competencies in young children.[8,9] Findings from randomized controlled trials (RCTs) conducted in childcare settings support policy action and have informed best practices in childcare settings.[9-13] However, we know little about how to scale up knowledge from RCTs into population level impact as few interventions have been scaled up and fewer have leveraged 'natural' implementation experiments to research.[8,9] Effective scale up strategies are needed to ensure current population-based approaches targeting childcare settings positively impact child outcomes.[8,9]

In June 2016, the Director of Licensing in British Columbia (BC) released the Standard of Active Play (**AP Standards**) for licensed childcare facilities and, in parallel, the BC Ministry of Health is investing $1.075 M in an evidence-based multi-component scale up intervention to support implementation of the AP Standards. This provides a unique opportunity to capitalize on this investment and conduct a "real world" implementation trial to advance knowledge of strategies that support policy action.

In this project, **our overall objective** is to evaluate implementation and impact of the AP Standards and supporting multi-component scale up intervention, which integrates both communication and capacity-building support interventions, on BC licensed childcare providers.

Our specific **AIMS** are to:
1) Assess whether the AP Standards and supporting scale up intervention significantly change policies of licensed childcare facilities and practices of providers over time.
2) Identify the hierarchy of factors that influence implementation of the AP Standards and uptake of the multi-component scale up intervention.
3) Determine whether children in BC licensed childcare centers that meet or exceed the AP Standards have better health behaviours and outcomes.

Hypothesis 1: In the first 2 years, implementation of AP Standards will result in a 10% increase per year in the proportion of licensed childcare facilities that have policies and provide: ≥60 min/day of outdoor active play; 120 min/day of combined indoor and outdoor active play; daily Fundamental Movement Skills (FMS) activities, limit screen use ≤ 30 min/day and restrict sitting time to ≤ 60 min at a time.

Hypothesis 2 (see Figure 1): Implementation of the AP Standards will be influenced by: ***Attributes of the Standards and intervention*** – moderated by adaptability/ triability, complexity, observability and relative advantage; ***Characteristics of providers*** – greater among those who have higher behavioural capacity, self-efficacy and commitment to trying and changing their practices; and ***Characteristics of childcare settings*** – greater in facilities with better structural support (better organizational climate, culture, capacity and environment; supportive policies and practices;

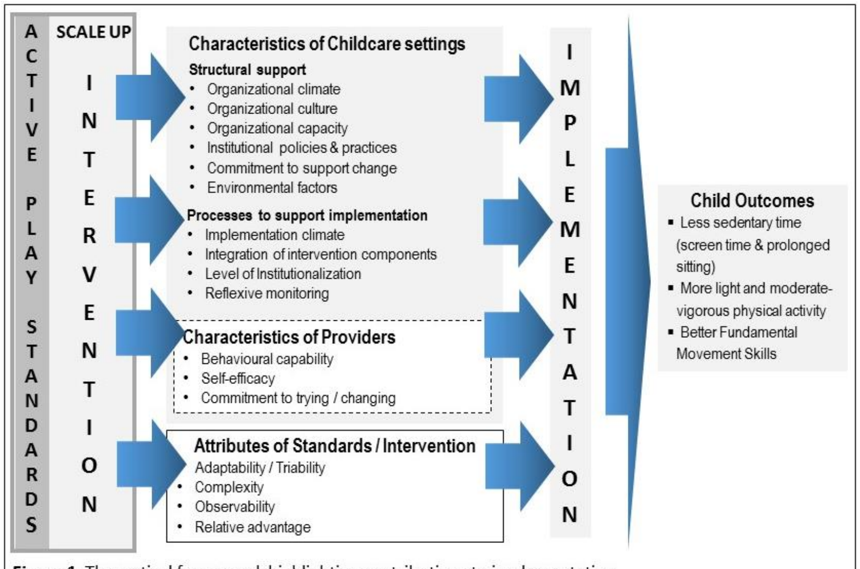

Figure 1: Theoretical framework highlighting contributions to implementation

a commitment to support change) and processes to support implementation (supportive implementation climate, integration of intervention components, higher level of institutionalizations and higher reflexive monitoring).

Hypothesis 3: There will be a dose response relationship in which higher implementation of the AP Standards will result in better FMS scores (overall, locomotor and object control skills), more min/day of light and moderate/vigorous PA and less sedentary time (prolonged sitting and screen time).

Our hypotheses are informed by our previous theoretically grounded implementation framework.[14-19] It includes four theoretical concepts from the *Diffusion of Innovations:*[20] 1) attributes of the Standards and intervention, 2) characteristics of providers, 3) linkage systems to technical support and programs, and 4) level of institutionalization (change in policies and practices). It also integrates *Social Cognitive Theory*[21,22] where childcare providers' commitment to trying/changing their practices is influenced dynamically by both characteristics of the providers and the socio-environmental context of childcare settings. From *Organizational Theory*,[23] we incorporate organizational climate, culture, and capacity as well as inter-organizational collaborations and integration of intervention components. Finally, based on Lau[24] and Damschroder,[25] we added implementation processes identified as key but currently understudied.

This project has several **novel aspects**, including: 1) expanding knowledge of strategies that support scaling up evidence-based policies and practices at the population level (an understudied priority);[8] 2) examining how system-level processes affects childcare environments and outcomes; and 3) advancing implementation science from a real world perspective.

BACKGROUND AND RATIONALE

More PA, reduced sedentary behaviours and FMS are critical to early child development and health.[1,2] Higher levels of PA and reduced sedentary behaviours are associated with a number of sustained developmental and health benefits in early childhood that track into adulthood, including: improved measures of adiposity,[1,2,26]cardiovascular risk factors (blood pressure and lipid profiles)[1,2,27] and bone health,[1,2,28,29] as well as motor skill acquisition,[1,2,27,30-34] and psycho-social,[1,35-37] cognitive and language development.[38-41]

PA, motor skill development and play are mutually supportive in the early years[5,42-44] and promote physical literacy "the motivation confidence, physical competence, knowledge and understanding …leading to lifelong PA engagement".[45] Exposure to a broad variety of PA opportunities supports skill acquisition[46] while motor competence promotes engagement in PA and perceptions of competence[55]. Fine and gross motor skill competence predicts a child's engagement in physically active pursuits[47-49] over the long-term.[33,47] *Thus, the early years represent a critical window of opportunity*[1,2] *when effective population-level strategies, if implemented, can foster motor skill development and enduring PA habits.*

Childcares are ideal settings for enhancing PA and motor skills in the early years.[40,41] In Canada, 50% of children younger than 5 years attend a licensed childcare facility for about 30 hours per week.[42] Here, early learning professionals and practitioners, physical and social environments and policies and practices shape children's PA habits and motor skills.[43] About 43% of children's PA is predicted by the childcare attended.[44] As young children do not control their environments or related choices,[45] designing supportive physical and social environments is an important shared responsibility for decision makers and caregivers. *Implementation of effective PA policy and practice interventions is key to enhancing the childcare environment and increasing PA and motor skill competencies in the early years.*[46-49]

Policy interventions are important. Recent reviews found that PA interventions conducted in childcare settings have been effective in increasing PA in the early years, [8-12] especially if those interventions included at least 30 minutes of structured PA and were delivered by skilled personnel.[8,9] This evidence supports policy action. As demonstrated in other areas (tobacco control,[50] dental health,[51] seat belt usage[52] and PA in school context[53]), policy interventions have tremendous potential to influence behavior and impact people at a relatively low cost (they are scalable).[8]

In childcare specifically, facility-level policies have positively impacted children's behaviours.[54,55] For instance, staff supervision policies related to media use was associated with reduced screen-time.[55] A US-based evaluation of the impact of PA policies for childcare facilities in New York showed that

policy action combined with extensive training and technical support significantly improved childcare settings' PA practices.[56] For each additional teacher trained the association increased by 9% and the odds of complying with an additional regulation increased 3.6 times[56] for every staff person enrolled in PA training. Similarly, implementation support (from managers) enhanced the likelihood of fully implementing policies or practices in Australia.[57] *Emerging evidence suggests that policy action supported by capacity-building can increase implementation of policies in childcare settings.*[14,56-58]

Studies about impact of provincial/state childcare PA policies are rare. In 2013, 54% of provinces/territories in Canada had legislative regulations to address the outdoor play environment and PA in childcare.[59] Most regulations focused on the provision of equipment, opportunities for outdoor play and adequacy of physical spaces, except one that specified a daily PA time requirement for children.[59] In the US, most state policies have provisions for the supervision in outdoor play areas and adequate play space per child but only 36/50 specified daily outdoor PA; only 9 set specific minimum times for children to be outdoors each day.[60,61] *To date in Canada, no studies have assessed the impact of provincial level PA policies on PA within childcare settings.*

The study of implementation is timely; knowledge of effective interventions to enhance implementation of policies and programs in childcare settings is lacking.[8,13] To realize their potential impact, policies must be implemented at scale.[62] However, implementation varies widely despite best practice recommendations. For instance, only 14% of US childcare facilities provided the recommended 120 min/day of PA, 60% had no written policy and in 20% children were seated for more than 30 minutes duration.[63,64] In an Australian study, half of childcare facilities had written policies and 40-56% provided daily FMS activities.[65] Despite progress in some countries, there is a notable gap between having supportive policies in the childcare setting and successfully implementing them.[13,66] It is not possible to develop effective standardized implementation protocols to support broad scale uptake of policy action without adequate evidence.

Current scale-up and implementation frameworks reflect the importance of strengthening organizational capacity and supporting implementation of polices and best practices,[25,62,67,68] as implementation in youth-serving organizations and childcare facilities are influenced by organizational capacity and quality of support systems.[24,57] *However, we still know little about the factors that influence implementation of policy supported by capacity-building, although such strategies are recommended in current scale-up frameworks.*[8,25,62,69,70]

ANTICIPATED OUTCOMES / SIGNIFICANCE

Understanding how to achieve wide scale implementation of policy actions at the population level is an important research and 'real world' priority.[8] Two recent bodies of evidence suggest that: 1) addressing PA and FMS in early childhood is important, and 2) that intervening with policies and practices in the childcare setting is needed.[9,13] However, we know little about how best to achieve implementation of AP Standards at scale in the childcare setting.

Our project will provide invaluable information about implementation support strategies and their impact on childcare PA policies and practices. Importantly, we aim to leverage a substantial government investment by conducting a natural experiment that examines the impact of AP Standards and supportive multi-component scale-up intervention to enhance the meagre evidence-base in this area. This will help researchers identify where implementation models and measures developed in other sectors can be modified for early years settings. Outcomes will help decision makers tailor their investments and activities to promote the outcome they hope to achieve, ideally physically literate children. In addition, our results have the potential to reshape FMS and PA habits of preschoolers; critical to early child development and health outcomes in the earlier years and later.[1,2] Finally, our findings will inform other jurisdictions in Canada who plan to employ similar population-based policy strategies.

2. APPROACH AND METHODS

STUDY DESIGN AND TIMELINE

To address Aims 1 and 2, we will use a mixed methods concurrent nested design (QUANT + Qual).

Quantitative: We will conduct a 2-year prospective study to monitor changes and factors that influence implementation of the AP Standards in childcare settings. Importantly, resources have already been committed to collect **baseline data** from all BC licensed group childcare providers in 2016/17 (using our

Early Years Surveys [EY Surveys] see measures) before the start of the $1.075M multi-component scale up intervention which includes both communication and capacity-building support interventions. We will re-administer the EY Surveys at the end of 2017/18 and again in 2018/19 to all eligible licensed childcare facilities and providers in BC (see timeline).

Qualitative: We will conduct 60 informant interviews from 30 licensed childcare facilities (half in 2018 and half in 2019) to gain in-depth understanding of the facilitators and barriers to implementation.

In Aim 3, we plan to collect child-level data among 650 children (aged 30 months to 5 year) from 65 facilities in 2019/20.

TIMELINE						
**Components of BC childcare scale up intervention** [a]	**2016**	**2017**	**2018**	**2019**	**2020**	**2021**
• AP Standards released June 2016 [b]						
• Communication intervention (Informational campaign)						
• Capacity-building support intervention						
**Duration of proposed study**		**Year 1**	**Year 2**	**Year 3**	**Year 4**	**Year 5**
Early Years Surveys (All BC licenced facilities N=~850) (Aims 1 & 2)						
Qualitative interviews 30 facilities/60 informants (Aim 2)						
Child (N=650) and childcare settings (N=65) observations (Aim 3)						
Data processing and analyses (Aims 1, 2 and 3)						
Decision maker input / knowledge transfer						

[a] In 2018 the BC Director of Licensing will start reporting on compliance with the AP Standards
[b] Ministry of Health has allocated $1.075M in 2017/18 and funding for other years not yet allocated

OVERVIEW OF MULTI-COMPONENT SCALE-UP INTERVENTION / AP STANDARDS

As shown in Figure 2, the BC childcare multi-component scale up intervention is guided broadly by:

1) Socio-ecological theory[71] at the macro/systems' level, policies, networked stakeholders, communication (i.e., informational campaign promoting the AP Standards) and a capacity-building support interventions. These elements are integrated to facilitate change at the meso and micro levels – meaning changes at the facility and provider levels.[72] Ultimately, the scale up intervention aims to change organizational infrastructure and childcare providers' practices to influence PA and FMS competence.

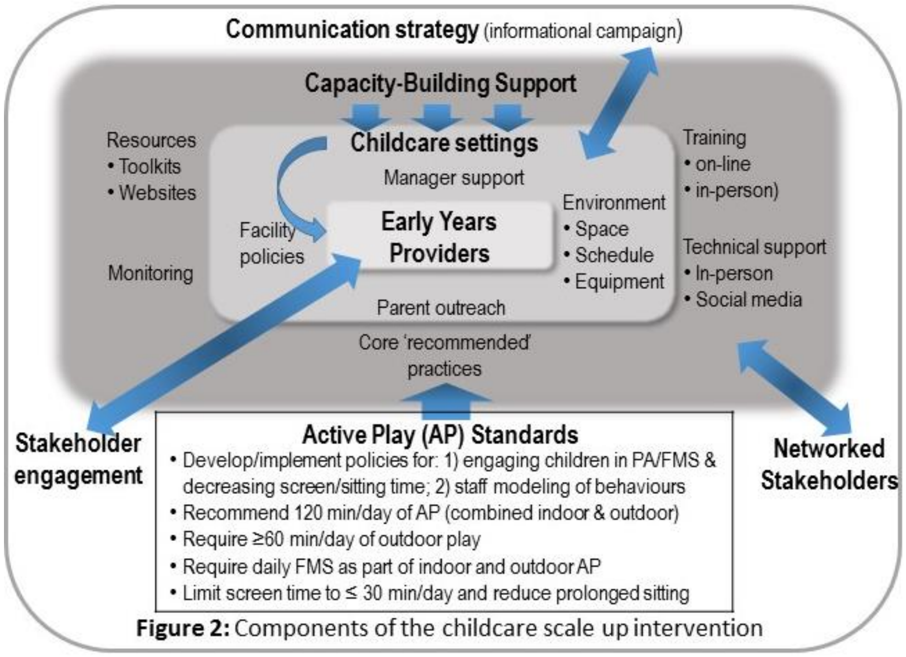

Figure 2: Components of the childcare scale up intervention

2) Simmons'[67] scale up and Fixsen's[69,70] implementation framework core elements are integrated: coordinated efforts and messaging (aligning resources with AP Standards), linked networks (provincial stakeholders, regional licensing officers council and local childcare and PA agencies), early engagement and input from childcare providers (through extensive consultations and involvement of an Early Years PA advisory team), promoting adaptability (choice-based resources toolkits), providing training (on-line and in-person) and technical support (site visits, proactive social media and regional experts), feedback mechanisms, and integrating delivery within existing channels (childcare resource and referral agencies, Head start, regional health authorities, local recreation and sport organizations).

Broadly, the intervention is a combination of policy action with supportive dissemination strategies. Within the broader scale up intervention, the communication and capacity-building support interventions (called Appetite to Play), have been designed collaboratively by Child Health BC in partnership with stakeholders and the Early Years PA Advisory Committee and researchers. The capacity building support intervention includes: web-site support, resource toolkits, pro-active social media, decentralized technical support using a train the trainer and regionalized approach, fixed guidelines but adaptable toolkits and monitoring through licensing visits and self-audit tools. The intervention strategies in these components are evidence-based,[8,13,62,70,73] and have gone through an extensive vetting process (through focus groups and surveys of 269 childcare providers).

PARTICIPANTS

This study targets group childcare facilities that provide care for children from infancy to school age (averaging 32 children per facilities) and account for 87% of the children in licensed care in BC. In June 2015, there were 3,044 licensed group childcare providers that provided care to 96,478 children across four service delivery areas (see below).

**Service delivery area**	# providers	# children
Interior (Kootenays, Okanagan, Thompson/Cariboo/Shuswap)	412	14,069
Coast/Fraser (E/N/S Fraser, Vancouver/Richmond, Coast/North Shore)	1,949	57,817
Vancouver Island (S/N Vancouver Island)	499	18,839
North (Northwest, North Central, Northeast)	184	5,753

AIMS 1 and 2: ELIGIBILITY / SAMPLE SIZE / DATA COLLECTION /MEASURES

Eligibility (AIMS 1 & 2): BC licenced childcare providers caring for children aged 30 months to 5 years are eligible. About 68% of the 3,044 licensed group childcare providers meet this criterion; 2,070 eligible facilities. We anticipate that 850 licensed childcare providers will complete the EY surveys in 2016; based on previous surveys.[74,75] We will conduct surveys again in 2017/18 and 2018/19 (see timeline) recruiting from the same eligibility pool to document implementation over time. Based on our previous work, we project a 50% overlap between years (N=450)[74] – meeting our sample requirement. Participation will be voluntary, so we will use post-stratification weights (based on census data) to weigh the sample and adjust for bias associated with voluntary participation.

Sample Size (AIMS 1 & 2): *Quantitative* - Using the most stringent requirement, we require pre and post-data from 368 childcare facilities to have 80% power to detect a 10% difference in the proportions of childcare facilities that significantly improve their policies and practices. This assumes the proportion at baseline is 0.35 using an α=.05 and 2 sided test (sampling increased to allow for stratified analyses as differences may exists [e.g., by low versus moderate/high income neighborhood]. We further adjusted the sample size to account for the design effect [DEFF= $1+ \delta(n_p-1)$, where n_p = average number of providers per facility and δ = intra-facilities correlation]. Assuming an average of 2 providers per facility and an intra-facility correlation of 0.10 resulted in a DEFF = 1.10. This increased the sample size to N=405 facilities with data in both 2016 and 2017/18 as well as 2016 and 2018/19.

Qualitative (AIM 2): Based on our previous research and recommendations for reaching theoretical saturation,[76] we estimate a sample size of 15 childcare facilities with 2 informants resulting in 30 interviews in each cycle of data collection 2017/18 and 2018/19 (total of 60 informant interviews).

Data Collection (AIMS 1 & 2): *Quantitative* - In both the 2017/18 and 2018/19 survey waves, eligible childcare facilities will be sent a preliminary notice which will be followed by an invitational package detailing data collection procedures which includes: informed consent and a link to the online EY surveys (administrator/staff survey). Administrators or assistants will forward invitational emails to their staff. Those who agree to participate will receive an email link to the staff EY surveys. Research staff will be emailing and calling administrators and their assistants to follow-up and ensure an acceptable response rate. Administrators and staff surveys take about 20 minutes to complete on-line. Staff surveys include self-report of typical and “previous day” practices. All participants will be compensated.

Qualitative (AIM 2): Facilities that take part in the EY surveys will be purposely selected to ensure variability in implementation of the AP Standards and representation from each service delivery region. We will conduct semi-structured interviews with at least 1 administrator and 1 staff.

Quantitative measures (EY-Surveys AIMS 1 & 2): We will collect information from both administrators and staff using an enhanced version of our EY follow-up surveys to assess implementation and factors that influence implementation (see list in Figure 1).

- **Implementation of AP Standards (Policies and Practices):** Our EY Surveys adapted the reliable childcare self-report Environment and Policy Assessment and Observation tool (EPAO-SR)[77] and Dodds' items (Intraclass correlation ICC from .55 to .93).[78] The "administrator EY survey" asks about the written or unwritten PA policies and the "staff EY survey" asks about typical and previous day practices related to min/day of indoor and outdoor play time and un-facilitated and facilitated play that incorporates FMS activities.
- **Attributes of AP Standards and intervention:** *Adaptability/ triability, complexity, observability, and relative advantage* measured with our 8-item scales modified for the childcare setting (α=.92 & supported by factor analysis[79]).
- **Characteristics of providers:** *Behavioural capability:* New items measuring providers' knowledge and skills. *Self-efficacy:* adapting our 8-item scale to assess confidence in implementing the AP Standards and using components of the scale up intervention, dedicating time to meet expectations and ability to engage children in activities. (α=.86 supported by factor analysis).[79] *Commitment to trying / changing practices:* New items assessing whether they are implementing and (if not) could in the next 3 months implement the AP Standards by: providing more than 60 min/day of outdoor time, 120 min/day of active play and daily FMS activities and by limiting screen time to 30 min/day or less and limiting prolonged sitting to 60 min at a time.
- **Characteristics of childcare settings – Structural Support:** *Organizational climate:* Using a 10-item semantic differential scale based on Hoy's measure (collegial, supportive, conciliatory, friendly, warm, open, welcoming, accepting of change, accommodating, trusting) (α=0.94).[80-83] *Organizational culture:* New items assessing whether innovation and change is encouraged/rewarded. *Organizational capacity:* structural resources for implementation [e.g., equipment, space, staff] and *Institutional policies and practices* with the EPAO-SR tool.[77] *Commitment to support change:* New items assessing both leadership support for changing policies and practices and organizational readiness (not open, hesitant, planning, and taken steps to make changes; partially implementing and fully implementing the changes). *Environmental factors:* New items measuring organizational size and SES of the community.
- **Characteristics of childcare settings – Processes to support implementation**: *Implementation climate:* New items assessing tension to change, compatibility with role and relative priority to change. *Integration of intervention components*: a) attended training (Yes/No item); b) links to provincial support modeled after the Alberta Heart Health project 2004 linkage questions[84] based on Valente's[85] and Robinson's[86] connectedness questions; and c) use of provincial resources/programs modeled after Steckler et al.'s[87] current use items to classify providers into 5 levels of users (non-users to high users). *Level of Institutionalization*: EPAO-SR tool[77] to assess change in policies and practices and new items for plans to maintain change assessing sustainability (retraining of new staff). *Reflexive monitoring:* New items assessing the extent to which as a group they have institutionalized processes for evaluating their implementation efforts.

Qualitative semi-structured interviews (AIM 2): We will develop guiding questions based on our theoretical framework (see Figure 1) to probe about the factors that enhance or hinder implementation of the AP Standards. Interviews will be digitally recorded and take about 45 to 60 minutes.

AIM 3: ELIGIBILITY / SAMPLE SIZE / DATA COLLECTION / MEASURES

Eligibility (AIM 3): Our primary sampling frame will be the childcare facilities that completed the 2018/19 EY Surveys. Given the intense nature of this Aim, we will recruit 65 facilities from the Coast/Fraser and the southern part of Vancouver Island service delivery regions as they account for 75% of the sample and represent the diversity in BC. In addition, facilities that provide care to at least 20 children (30 months to 5 year) will be targeted. We will systematically sample facilities using implicit stratification with respect to level of implementation of the AP Standards (using responses to the 2018/19 EY surveys) and using urbanicity, % of visible minority and % of population with post-secondary education around the childcare facility based on the census data. (targeting ~8% of eligible)

Sample size (AIM 3): For a multiple linear regression model that already includes multiple covariates with a R^2 of 0.03, a sample size of 434 will have 80% power to detect at an alpha 0.05 an increase in R^2

of 0.03 due to including implementation variables to explain PA and FMS in children. Assuming 10 children are enrolled per site and an intra-facility correlation of 0.03 (based on published data[88] resulted in a DEFF = 1.27 which further increased the sample to 551 and after adjustment for missing data (15%) resulted in requiring 650 children from 65 licensed providers for AIM 3.

Data collection (AIM 3): Childcare providers will facilitate delivery of invitation packages to parents. Children, whose parents consent for their participation, will wear an accelerometer and GPS device for 4 days from the time they arrive at the facility to departure. On those same days, staff will conduct field observations. To account for weather effects, we will ensure that each facility observation set includes at least 1 non-rainy day (no rain from 9:30–11:00 am & 1:00–3:00 pm). We will collect data in units of 2 facilities at a time to match data collection days of low and high implementers. Matching will account for our stratification variables and proximity of the facilities to ensure the 2 facilities are subject to the same inclement weather. At their last visit, staff will collect the FMS data with the Test of Gross Motor Development (TGMD-2) which takes 15-20 minutes to administer. Finally, we will re-administer a shorter version of the EY Surveys to capture changes since the last cycle of data collection.

Measures (AIM 3): Child's Indoor and Outdoor Sedentary, Light and Moderate-Vigorous PA will be measured using direct measurement via wearable devices as well as field observations.

- **Wearable devices***:* Children will wear two devices: 1) the GT3X$^+$ accelerometer (Actigraph, Pensocola, FL) extensively validated to measure PA in children;[89-91]and 2) the GPS forerunner 230 watch (Garmin, KS) as GPS devices have higher accuracy to detect indoor and outdoor time of preschoolers (accuracy of .89 to detect outdoor time).[92]
  The GT3X+ accelerometer is a small (4.6cm x 3.3cm x 1.5cm; 19 grams) tri-axial monitor designed to assess human motion (0.25–2.5 Hz range) and filter non-human motion. The device will be worn under children's clothes secured on the right or left hip at the level of the iliac crest. Children will wear the device for 4 weekdays as it provides adequate ICC (>=.70) in preschool children.[93] Data will be collected in 15-second epochs and processed with preschoolers' cut-points – minutes per day of: 1) Sedentary activity = 15-s counts < 48; b) Light physical activity =15-s counts ≥48 & <420 and 2) Moderate-vigorous PA = 15-s counts ≥420.[94,95] In all analyses, wear time will be entered as a covariate to adjust for variations in time spent at the childcare.
  The GPS forerunner 230 watch records longitude and latitude with a 2-3 metre accuracy. The GPS device will be affixed to the Actigraph belt and worn on the same days as the GT3X+ is worn. Data will be collected in 1 second epoch. Indoor and outdoor time will be assessed by computing the signal-to-noise ratio (SNR or strength of the signal), where SNR >250 has been validated to assess outdoor time with 90% accuracy.[96]
  We will follow the protocol we used in our CIHR playability study (Mâsse/Brussoni), the GT3X+ and GPS data will be merged and processed with the Personal Activity and Location Measurement System software (PALMS San Diego, CA).
- **Field observations** will supplement the wearable tools and utilize the field component of the EPAO measure; integrating observations of active and sedentary opportunities (ICC = .67 and 1.00 respectively).[97] EPAO observational tool will be expanded to document location of the activities (indoor/outdoor), intensity of the activity (sedentary, light or moderate-vigorous), types of activities (teacher led or not, FMS activity or not) and whether practices are influenced by gender norms. Observations will yield both childcare and child indices.

Fundamental Movement Skills (FMS) will be assessed with the validated Test of Gross Motor Development (TGMD-2 www.proedinc.com/customer/productView.aspx?ID=1776) a norm-referenced tool for assessing 12 gross motor skills - locomotor (run, gallop, hop, leap, horizontal jump and slide) and object control (strike, dribble, kick, catch, throw, and roll a ball) (test retest r .85 to .91 and α ≥ .90). The measure takes 15-20 min per child to administer and provides standardized and percentile composite scores for the locomotor, object control, and gross motor quotient (GMQ) composites.

DATA ANALYSES

Quantitative data. To address **AIM 1**, we will use longitudinal multi-level mixed effects dichotomous and polytomous logistic regressions to model changes in policies and practices triggered by the AP Standards. These analyses will quantify implementation 1 and 2 years following our initial assessment (2016/17). A mixed effects model can account for the clustering of the data and the unbalanced nature of

the dataset (facilities and providers who completed 2016/17, 2017/18 and/or 2018/19 EY surveys). In separate analyses we will examine change in: 1) policy strength (i.e., no policy, written policy without or with specificity) and 2) practices whether they meet minimum AP Standards [≥60 min/d of outdoor active play; ≥120 min/d of active play indoor and outdoor; daily FMS, screen use ≤ 30 min/d and prolonged sitting ≤ 60 min at a time].

To address **AIM 2**, longitudinal analyses from AIM 1 will serve as the base model where all the variables hypothesized to influence change (see Figure 1) will be entered as independent variables (i.e., attributes of the AP Standards and intervention, characteristics of providers, characteristics of childcare settings include both structural support and processes to support implementation). This analysis will take into account time dependency in the independent variables. In addition, childcare demographic characteristics will be entered as covariates (e.g., census variables linked by postal code such as urbanicity, % of visible minority and % of population with post-secondary education around the facility and using childcare facilities demographic variables such as size and number of staff among others).

To address **AIM 3**, we will use hierarchical mixed models that incorporate both fixed and random effects at the childcare and child level and account for design effects (clustering of children within childcares). Several analyses will be conducted using the following as dependent variables: FMS scores and daily average (min/day) of indoor and outdoor sedentary and light and moderate-vigorous PA. The corresponding level of policy and practice variables will be used as the main independent variables as well as including child level demographic variables (age, sex) as covariates.

Qualitative analyses (AIM 2): Administrators and staff transcripts from the same facilities will be linked to enable childcare unit analyses. Techniques from constructivist grounded theory approach (see Charmaz)[98-100] will guide the initial analyses using NVivo-10 software. Our analyses will also be guided by Straus and Corbin[101] procedures and will integrate Knalf and Ayres[102] procedures to ensure the childcare unit is accounted in the analyses. Our analytical plan will first analyze the transcripts of individuals (administrators and providers individually) and then shift our focus to analyzing the childcare as a unit.[102] This sequence consists of shifting emphasis from within and across individuals to a series of constant comparative analysis within and across childcare facilities.

Mixed-method data integration: Data integration will begin after we have analyzed both the quantitative and qualitative data. This will require that both the qualitative and quantitative data be systematically integrated and contrasted in both the analysis and interpretation stages with more in-depth analyses using our theoretical framework (Figure 1) as an explicit theoretical lens. In addition, we will explore whether providers' practices and child preferences for activity types are influenced by gender norms in the childcare facilities using the social construction of gender framework.[103]

CHALLENGES & MITIGATION STRATEGIES

Impact of seasonality on study outcomes. Seasonality can influence providers' practices and child outcomes. For AIMS 1 and 2, we mitigate this by assessing both previous days' practices and typical practices and ask respondents to report previous day weather (used as confounder in analyses). In addition, we standardized the time when the EY surveys are administered (collected in the same months across years). For AIM 3, we mitigate this by: 1) collecting data from facilities in units of 2 (a low and a high implementer in the same region on the same days); and 2) requiring that one data collection is a non-rainy day; and 3) spread data collection over 1 year.

Delays in intervention delivery. The primary components of the scale up intervention were developed in 2015/16 and have undergone extensive pilot testing. It was a priority to ensure availability of the multi-component intervention when the AP Standards were fully enacted in June 2017. The expected start date for the scale up intervention is early 2017 but in real world trials implementation delays are common. Our data collection can easily accommodate a 6-month delay in start date and if longer we can shift our EY surveys by a year without implications to our study.

KNOWLEDGE TRANSLATION PLAN

We have strong relationships with government and stakeholders to undertake this study (see letters of support). We have translated research findings to inform policies (school PA policies Mâsse/Naylor) and to evaluate the effectiveness of provincial scale-up strategies (Action Schools!BC scaled to 1600 schools in BC Mâsse/Naylor).[15,79] In addition, Naylor serves on the Early Years PA and Healthy Eating

Advisory Committee of the provincial PA Strategy and the sub-committee which informed the AP Standards and the multi-component scale up intervention. As such, ongoing knowledge exchange will occur between researchers and early years stakeholders and decision-makers. As a committee member, Naylor will ensure that evaluation is a standing item on the agenda for the Early Years advisory committee that meets quarterly. We will have a direct line of communication to discuss findings from our evaluations. Regional reports will be prepared for licensing officers. Most importantly this research will produce tangible knowledge exchange opportunities. We have planned for wider involvement of decision makers in our study as shown in our timeline, where they provide input to our instrumentation and help us develop knowledge translation products. In addition, to formally sharing our research findings with the scientific community (through presentations and publications), we will go beyond academic "currency" to inform practice and policy by sharing findings using News Flashes, Community Reports, infographics, short videos, public and stakeholder symposia, panel discussions and presentations at the Ministry Health policy rounds.

3. EXPERTISE, EXPERIENCE AND RESOURCES

RESEARCHERS

Mâsse (PI): Professor, School of Population and Public Health, UBC, MSFHR Senior Scholar. I have >20y experience in leading an active prevention research program with expertise in PA, evaluation of population-based interventions including policy evaluations enacted at the state/provincial level and the hierarchy of factors that influence implementation of interventions and policies, and measurement of PA. Mâsse will oversee all scientific and administrative aspects of this project. (40% effort time)

Naylor (co-PI) Professor, School of Exercise Science, Physical & Health Education, UVic. Naylor has extensive expertise in implementation science, setting-based interventions (childcare, schools, recreational centres), knowledge exchange and scale up/capacity-building interventions related to PA. Naylor will co-lead recruitment, design of study instrument and data collection. (35% effort time)

Brussoni (Co-A) Associate Professor, Pediatrics and School of Population and Public Health, UBC MSFHR scholar. Expertise in active play, developing playground guidelines to stimulate active and risky play and mixed methodology. She will provide a key role in measuring environmental factors in the childcare settings that influence child outcomes (PA, sedentary behaviours and FMS). (5-20% effort)

Carson (Co-A) Assistant Professor, Faculty of Physical Education and Recreation, U of A. CIHR New Investigator Salary Awardee. She has expertise in PA /Sedentary behaviours in early years and is currently funded by CIHR to evaluate the Alberta accreditation standards that address PA for childcare settings. She will guide assessment of behaviours and inform instrumentation. (5-20% effort)

Faulkner (Co-A) Professor School of Kinesiology, UBC, CIHR-PHAC Chair in Applied Public Health with extensive experience in the evaluation of large-scale provincial and national PA interventions and policies. He will guide implementation tools and refinement of the evaluation model. (5-20% effort)

Lau (Co-A) CIHR post-doctoral fellow, School of Kinesiology UBC. Emerging expertise in implementation sciences and dissemination of interventions in youth serving settings. She will provide input on factors that influence implementation and indirectly impact child outcomes. (5-20% effort)

Temple (Co-A) Professor, School of Exercise Science, Physical & Health Education, UVic with expertise in FMS, PA, interventions in childcare settings and conducting longitudinal studies in young children. She will guide assessment of FMS, provide key input on instrumentation and intervention evaluation in childcare. (5-20% effort)

Wolfenden (Co-A) Associate Professor and Brawn Career Development Fellow, University of Newcastle Australia is an internationally-recognized implementation scientist and public health researcher with experience in conducting large-scale randomized controlled trials in community settings including a number of large implementation trials in the childcare setting aimed at changing PA of preschoolers. He will provide key input on instrumentation related to assessing policies and practices in the childcare settings as well as factors that influence implementation. (5-20% effort)

EXPERIENCE/EXPERTISE

This Project is founded on the extensive stakeholder partnerships, expertise and experiences of the research team. It extends the established research programs of the PI (Mâsse) and co-PI (Naylor), who

have a strong history of collaboration and research productivity – collectively Mâsse/Naylor have been awarded over $9.5M in project funds as PI/co-PI and over $19M as co-applicants. It builds on the recognized expertise and productivity of the co-applicants (Brussoni/Carson/Faulkner/Lau/Temple/ Wolfenden hold >$16M in project funds as PI). Related to this Project, we have:

- *Advanced knowledge of scale up and implementation sciences* – Mâsse/Naylor published the 1st studies which identified factors that influenced implementation of provincial initiatives after scale-up;[15,79] Mâsse/Naylor/Faulkner/Wolfenden/Temple conducted implementation studies in childcare / youth based organizations; [8,17,104-110] Mâsse/Naylor/Lau/ Wolfenden informed implementation sciences through syntheses (Cochrane review) and expert driven processes.[9,13,14,24,73,111,112]
- *Evaluated policy implementation* – Mâsse identified factors that impact policy implementation[113] – Mâsse published 1st conceptual framework for evaluating impact of state/provincial PA policies;[19] Mâsse/Naylor tracked change in state/provincial policies;[74,114] Mâsse/Naylor/Faulkner/Wolfenden assessed the impact of policies on child outcomes;[65,115-119] Wolfenden developed measured that assess policies and practices in childcare settings.[78]
- *Led other early years PA, sedentary behaviours, active play and FMS work* – Brussoni/Carson/ Faulkner contributed to PA, sedentary behaviours and active play guidelines for early years and children;[120-124] Naylor/Carson/Temple assessed prevalence of PA, sedentary behaviours and FMS in childcare[3,125-127] Naylor/Temple conducted 1st Canadian longitudinal cohort K-Gr3 that measured PA and FMS and showed object and locomotor FMS skills below norms.[7] VC conducted the 1st study in Canada that objectively measured PA and sedentary behaviours in childcare;[126] Naylor/Temple/ Wolfenden developed interventions to improve FMS and PA in childcare;[4,48,128-131] Brussoni examined environmental factors related to active play in childcare;[132,133] Naylor/Carson/Temple studied developmental and health outcomes associations with PA and FMS.[1,2,5,7,134,134]
- *Methodological expertise in:* qualitative methods (Mâsse/Naylor/Brussoni), psychometrics (Mâsse) and mixed-effects models (Mâsse/Carson/Faulkner/Wolfenden); measuring PA/sedentary behaviours (Mâsse/Naylor/Carson/Temple, Wolfenden) and FMS (Naylor/Temple).

RESOURCES

Mâsse's (nominated PI) research unit at the BC Children's Hospital Research Institute is:

- Equipped with a *Survey Laboratory* to automate collection and analysis of email, web and scannable surveys; and *Field Measurement Tools* (70 accelerometers, portable scales and stadiometers – provided in-kind with CFI funds to establish the Childhood Obesity Prevention Unit).
- Supported by a research manager (D Mount) who connects staff and trainees across research group and helps bring on-board new research staff; financial manager (M Gottenbos) who manages CIHR budgets and financial reporting; and well-established research support for ethics, grants administration, finance, communications, IMIT, facilities, Research Education, Research Development.
- Physically located in a dynamic environment that fosters collaborations and interactions among faculty, trainees and research staff; provides educational opportunities to research staff and trainees (eg, ethics application, budgeting, project management, communication skills, data management and analysis); provides travel awards for trainees ($1500/yr); has a dedicated IT research support team that offers training and support in REDCap, SPPS and STATA; and provides biostatistical services.

Naylor's (co-PI) CFI funded Chronic Disease Prevention Knowledge Exchange research unit provides:

- A dynamic environment for Faculty, research trainees and coordinators including a meeting facility equipped with presentation and video-conferencing technologies, a dedicated secure research local area network, hardware and software for on-line surveying, qualitative and quantitative analysis and analysis of accelerometry data and field measurement tools (50 accelerometers, portable scales and stadiometers, and hard drives for storing video data).
- Geographic proximity to the BC Ministries of Health and Community, Sport and Culture allowing for continuous knowledge exchange with policy-makers.

This project presents considerable opportunities for student training and mentoring of first time investigator (Lau). Trainees will also have excellent networking opportunities locally and internationally within our team and our respective networks. Trainees will be co-supervised to gain transdisciplinary knowledge to identify mechanisms or levers that accelerate change, or influence responsiveness, to new policy – an important next step to influence policies and practices of childcare settings.

BUDGET JUSTIFICATION CIHR - $960,000 (needs to be rounded to multiples of $5000)

Research staff ($507,000)
Project Coordinator: 100% FTE Yrs 1-5; UBC Social Science Researcher Level B FTE = $62,000 + 18% benefits + 2% cost of living increase: MSc with minimum of 5yrs experience in coordinating population-based research. Will be responsible for coordinating the data collection in the child care settings and have expertise in PA and assessment of Fundamental Movement Skills (FMS), knowledge of qualitative and complex quantitative methods using software such as STATA, MPlus & NVivo to oversee management of study **= $383,500**

Research Technician: 100% FTE in Yrs 3& 4; UBC Research Technician Level 4 FTE = $50,000 + 18% benefits + 2% cost of living increase): The technician will be an MSc-trained person with experience in conducting research in children. S/he will assist with child data collection (recruitment, data collection (accelerometer, GPS & FMS), data processing & data analysis **= $123,900**

Trainees ($245,000)
Support is for:

- **2 MSc students**: 2*2yr stipend **=$80,000**
- **1 PhD student**: 1*3yr stipends **= $75,000**
- **1 Post-doctoral fellow**: 1*2yr stipends = **$90,000**

Trainees will gain experience and knowledge in the following (appropriate to their level): on collecting and conducting qualitative data analyses and collecting data (physical activity, Fundamental and GPS data), processing data (set-up databases and cleaning data), and analyzing the prospective qualitative and quantitative data throughout the duration of the project. Trainees will also be involved in data collection throughout the project. The trainees will gain valuable experience in helping analyze data and write manuscripts under the mentorship of the PI and Co-PI. Trainees will also gain valuable experience in learning how to conduct complex data analyses in multiple statistical software programs (eg, Stata & MPlus).

Non-Consumables ($5,000)

- **Actigraph licenses:** 2 for processing accelerometer data $2500 each**= $5,000**

Consumables ($58,000)

- **Supplies**: $50/mth**=$3,000**
- **Computers**: 2 desktops & 2 laptops=**$7,320**
- **Software licences:** 4 STATA $3,400; 2 MPlus $2,506; 3 2 RUMM2030 $3,360; N-Vivo provided in-kind**=$9,266**
- **Digital recorders**: 2**= $199**
- **Video recorders for FMS assessment:** 2=$**1500**
- **Accelerometers and belts**: Require 30 accelerometers/week (provided in-kind) and 650 belts/child *$5=**$3,250**
- **GPS Forunner 230:** 30 units * $369.60=**$11,088**
- **Transcription**: 60 interviews (15 facilities * 2 (2017 & 2018 interviews) * 2 (administrator/staff pair at each site) *$30/hr * 5.5 hr/transcripts=**$9,900**
- **Travel to childcare facilities: [**Informant interviews in 2017/2018: 30 facilities * avg of 50 km/roundtrip * .50 cent/km=$750] + [Child data collection in 2019/2020: 65 facilities * 7 round trips (2 for recruitment + 5 days of data collection PA/GPS and FMS assessment) * avg of 50 km/ roundtrip * .50 cent/km=$11,375]= **$12,125**

Other ($101,000)
Telecommunication: conference calls $300/yr **= $1,500**
Nominal incentives are integral to these studies to compensate participants for their time and for the outcomes of the study. We include both nominal cash and draws to manage these costs. Amounts in line with what we provide in previous studies.

- BC Child Care Survey (administrator/staff incentives): [$15 * 1000 facilities * 2 (1 administrator & 1 staff) * 2 (collection in 2017 &2018) =$60,000] & [$100 cash draw * 80 draws=$8000] =**$68,000**
- Informant interviews (N=60): 30 facilities * 2 (1 administrator + 1 staff) * $50 cash incentive = **$3,000**
- Child data collection: [650 children * $25 FMS toy/game given to parent = $16,250] + [65 facilities incentive *$150 (1 hr instruction of FMS provided by a specialist) = $9,750] + [Administrator + Staff cash incentive for completing surveys 2*$25 cash * 65 facilities = $3,250] = **$29,250**

Knowledge translation ($44,000)

- **Conference travel**: 2 conferences in all years **(**Program Leader + 1 trainee) (flight $750, registration $800, accommodation 5*$200/night, 6*$50 per diem, $150 taxi cabs) **= $30,000**
- **Meeting travel** to Ministries in Victoria BC. We have a history of cultivating relationships with BC government so we budgeted to travel for meeting with the BC ministries to ensure they guide our research questions and that we address questions that matter to our stakeholders. We budgeted meetings in Years 1, 3, 4, and 5. 4 trips at $300/trip **= $1,200**
- **Publications:** We have budgeted for the cost of publishing and many of our leading journals charge about $1500 per article. We have budgeted for 8 papers **= $12,800**

BUDGET JUSTIFICATION SSHRC - $398,997

Research staff (\$227,888)
Project Coordinator: .75% FTE Yrs 1-4; UBC Social Science Researcher Level B FTE = \$62,000 + 18% benefits + 2% cost of living increase: MSc with minimum of 5yrs experience in coordinating population-based research. Will be responsible for coordinating the data collection in the childcare facilities, conduct qualitative interviews and have expertise in PA. Knowledge of qualitative and complex quantitative methods using software such as STATA, MPlus & NVivo to oversee all aspects of the study **= \$227,888**

Trainees (\$85,000)
Support is for:
- **2 MSc students**: 2*1yr stipend each \$17,500 = **\$35,000**
- **1 PhD student**: 1*2yr stipends **= \$50,000**

Trainees will gain experience and knowledge in the following (appropriate to their level): on collecting and conducting qualitative data analyses and collecting data surveillance data, processing data (set-up databases and cleaning data), and conduct longitudinal analysis to access impact of the BC Active Play Standard of Practice Guidelines. Trainees will also be involved in data collection throughout the project. The trainees will gain valuable experience in helping analyze data and write manuscripts under the mentorship of the PI. Trainees will also gain valuable experience in learning how to conduct complex data analyses in multiple statistical software programs.

Consumables (\$16,909)
- **Supplies**: \$50/mth **= \$2,400**
- **Computers**: 1 desktops = **\$1,960**
- **Software licences:** 2 STATA \$1,700; N-Vivo provided in-kind **= \$1,700**
- **Digital recorders**: 2 **= \$199**
- **Transcription**: 60 informant interviews (15 facilities * 2 (2017 & 2018 interviews) * 2 (administrator/staff pair at each site) *\$30/hr * 5.5 hr/transcripts = \$ **\$9,900**
- **Travel to childcare facilities:** Informant interviews in 2018/2019: 30 facilities * avg of 50 km roundtrip * .50 cent/km = **\$750**

Other (\$51,800)
Telecommunication: conference calls \$200/yr **= \$800**
Nominal incentives are integral to these studies to compensate participants for their time and for the outcomes of the study. We included both nominal cash and draws to manage these costs. Amounts are in line with what we provide in previous studies.
- BC Child Care Survey (administrator/staff incentives): [\$10 * 1000 facilities * 2 (1 administrator & 1 staff) * 2 (collection in 2017 &2018) =\$40,000] & [\$100 cash draw * 40 in 2017 and in 2018 draws=\$8000] =**\$48,000**
- Informant interviews (N=60): 30 facilities * 2 (1 administrator + 1 staff) * \$50 cash incentive = **\$3,000**

Knowledge translation (\$17,400)
- **Conference travel**: 1 conferences in all years **(**Program Leader + 1 trainee) (flight \$750, registration \$800, accommodation 5*\$200/night, 6*\$50 per diem, \$150 taxi cabs) **= \$12,000**
- **Meeting travel** to Ministries in Victoria BC. We have a history of cultivating relationships with BC government so we budgeted to travel for meeting with the BC ministries to ensure they guide our research questions and that we address questions that matter to our stakeholders. We budgeted meetings in Years 1, 3 and 4. 3 trips at \$300/trip **= \$900**
- **Publications:** We have budgeted for the cost of publishing and many of our leading journals charge about \$1500 per article. We have budgeted for 3 papers **= \$4,500**

Reference List

(1) Timmons BW, Leblanc AG, Carson V et al. Systematic review of physical activity and health in the early years (aged 0-4 years). *Appl Physiol Nutr Metab* 2012;37:773-792.
(2) Leblanc AG, Spence JC, Carson V et al. Systematic review of sedentary behaviour and health indicators in the early years (aged 0-4 years). *Appl Physiol Nutr Metab* 2012;37:753-772.
(3) Colley RC, Garriguet D, Adamo KB et al. Physical activity and sedentary behavior during the early years in Canada: a cross-sectional study. *Int J Behav Nutr Phys Act* 2013;10:54.
(4) Adamo KB, Wilson S, Harvey AL et al. Does Intervening in Childcare Settings Impact Fundamental Movement Skill Development? *Med Sci Sports Exerc* 2016;48:926-932.
(5) Crane JR, Naylor PJ, Cook R, Temple VA. Do Perceptions of Competence Mediate The Relationship Between Fundamental Motor Skill Proficiency and Physical Activity Levels of Children in Kindergarten? *J Phys Act Health* 2015;12:954-961.
(6) Goldfield GS, Harvey A, Grattan K, Adamo KB. Physical activity promotion in the preschool years: a critical period to intervene. *Int J Environ Res Public Health* 2012;9:1326-1342.
(7) LeGear M, Greyling L, Sloan E et al. A window of opportunity? Motor skills and perceptions of competence of children in kindergarten. *Int J Behav Nutr Phys Act* 2012;9:29.
(8) Wolfenden L, Finch M, Wyse R, Clinton-McHarg T, Yoong SL. Time to focus on implementation: the need to re-orient research on physical activity in childcare services. *Aust N Z J Public Health* 2016;40:209-210.
(9) Finch M, Jones J, Yoong S, Wiggers J, Wolfenden L. Effectiveness of centre-based childcare interventions in increasing child physical activity: a systematic review and meta-analysis for policymakers and practitioners. *Obes Rev* 2016;17:412-428.
(10) Ward DS. Physical activity in young children: the role of child care. *Med Sci Sports Exerc* 2010;42:499-501.
(11) Ward DS, Vaughn A, McWilliams C, Hales D. Interventions for increasing physical activity at child care. *Med Sci Sports Exerc* 2010;42:526-534.
(12) Kreichauf S, Wildgruber A, Krombholz H et al. Critical narrative review to identify educational strategies promoting physical activity in preschool. *Obes Rev* 2012;13 Suppl 1:96-105.
(13) Wolfenden L, Jones J, Williams CM et al. Strategies to improve the implementation of healthy eating, physical activity and obesity prevention policies, practices or programmes within childcare services. *Cochrane Database Syst Rev* 2016;10:CD011779.
(14) Naylor PJ, Nettlefold L, Race D et al. Implementation of school based physical activity interventions: a systematic review. *Prev Med* 2015;72:95-115.
(15) Naylor PJ, McKay HA, Valente M, Masse LC. A mixed-methods exploration of implementation of a comprehensive school healthy eating model one year after scale-up. *Public Health Nutr* 2016;19:924-934.
(16) Masse LC, McKay H, Valente M, Brant R, Naylor PJ. Physical activity implementation in schools: a 4-year follow-up. *Am J Prev Med* 2012;43:369-377.
(17) McKay HA, Macdonald HM, Nettlefold L, Masse LC, Day M, Naylor PJ. Action Schools! BC implementation: from efficacy to effectiveness to scale-up. *Br J Sports Med* 2015;49:210-218.
(18) Mâsse LC, Frosh MM, Chriqui JF et al. Development of a School Nutrition-Environment State Policy Classification System (SNESPCS). *American Journal of Preventive Medicine* 2007;33:S277-S291.
(19) Mâsse LC, Chriqui JF, Igoe JF et al. Development of a Physical Education-Related State Policy Classification System (PERSPCS). *American Journal of Preventive Medicine* 2007;33:S264-S276.

(20) Rogers EM. *Diffusion of Innovations*. 5th Edition ed. New York: Free Press, 2003.
(21) Bandura A. *Social foundations of thought and action: A social cognitive theory*. New Jersey: Prentice Hall, Inc., 1986.
(22) Bandura A. Health promotion by social cognitive means. *Health Educ Behav* 2004;31:143-164.
(23) Steckler A, Goodman RM, Kegler MC. Mobilizing organizations for health enhancement: Theories of organizational change. In: Glanz K, Rimer BK, Lewis BM, eds. *Health Behavior and Health Education*. 2nd ed. San Francisco: Jossey-Bass; 2002;335-360.
(24) Lau EY, Wandersman AH, Pate RR. Factors influencing implementation of youth physical activity interventions:An expert perspective. Translational Journal of the American College of Sports Medicine 1[7], 60-70. 2016.
(25) Damschroder LJ, Aron DC, Keith RE, Kirsh SR, Alexander JA, Lowery JC. Fostering implementation of health services research findings into practice: a consolidated framework for advancing implementation science. *Implement Sci* 2009;4:50.
(26) Janz KF, Kwon S, Letuchy EM et al. Sustained effect of early physical activity on body fat mass in older children. *Am J Prev Med* 2009;37:35-40.
(27) Haga M. Physical fitness in children with high motor competence is different from that in children with low motor competence. *Phys Ther* 2009;89:1089-1097.
(28) Janz KF, Gilmore JM, Levy SM, Letuchy EM, Burns TL, Beck TJ. Physical activity and femoral neck bone strength during childhood: the Iowa Bone Development Study. *Bone* 2007;41:216-222.
(29) Janz KF, Letuchy EM, Eichenberger Gilmore JM et al. Early physical activity provides sustained bone health benefits later in childhood. *Med Sci Sports Exerc* 2010;42:1072-1078.
(30) Duncan MJ, Stanley M. Functional movement is negatively associated with weight status and positively associated with physical activity in british primary school children. *J Obes* 2012;2012:697563.
(31) Graf C, Koch B, Falkowski G et al. Effects of A School-Based Intervention on BMI and Motor Abilities in Childhood. *J Sports Sci Med* 2005;4:291-299.
(32) Hardy LL, King L, Kelly B, Farrell L, Howlett S. Munch and Move: evaluation of a preschool healthy eating and movement skill program. *Int J Behav Nutr Phys Act* 2010;7:80.
(33) Barnett L, Hinkley T, Okely AD, Salmon J. Child, family and environmental correlates of children's motor skill proficiency. *J Sci Med Sport* 2013;16:332-336.
(34) Robinson LE, Stodden DF, Barnett LM et al. Motor Competence and its Effect on Positive Developmental Trajectories of Health. *Sports Med* 2015;45:1273-1284.
(35) Burdette HL, Whitaker RC. Resurrecting free play in young children: looking beyond fitness and fatness to attention, affiliation, and affect. *Arch Pediatr Adolesc Med* 2005;159:46-50.
(36) Piek JP, Baynam GB, Barrett NC. The relationship between fine and gross motor ability, self-perceptions and self-worth in children and adolescents. *Hum Mov Sci* 2006;25:65-75.
(37) Skinner RA, Piek JP. Psychosocial implications of poor motor coordination in children and adolescents. *Hum Mov Sci* 2001;20:73-94.
(38) Fedewa AL, Ahn S. The effects of physical activity and physical fitness on children's achievement and cognitive outcomes: a meta-analysis. *Res Q Exerc Sport* 2011;82:521-535.
(39) Hillman CH, Schott N. Fitness and cognitive performance in childhood. Zeitschrift Fur Sportpsychologie 20[1], 33-41. 2013.
(40) Leonard HC, Hill EL. Review: the impact of motor development on typical and atypical social cognition and language:a systematic review. Child and Adolescent Mental Health 19[3], 163-170. 2014.

(41) Carson V, Hunter S, Kuzik N et al. Systematic review of physical activity and cognitive development in early childhood. *J Sci Med Sport* 2016;19:573-578.
(42) Williams HG, Pfeiffer KA, O'Neill JR et al. Motor skill performance and physical activity in preschool children. *Obesity (Silver Spring)* 2008;16:1421-1426.
(43) Stodden D, Langendorfer S, Roberton MA. The association between motor skill competence and physical fitness in young adults. *Res Q Exerc Sport* 2009;80:223-229.
(44) Riethmuller AM, Jones R, Okely AD. Efficacy of interventions to improve motor development in young children: a systematic review. *Pediatrics* 2009;124:e782-e792.
(45) International Physical Literacy Association. Canada's Physical Literacy Consensus Statement. 2014.
(46) Hardy LL, O'Hara BJ, Rogers K, St GA, Bauman A. Contribution of organized and nonorganized activity to children's motor skills and fitness. *J Sch Health* 2014;84:690-696.
(47) Henrique RS, Re AH, Stodden DF et al. Association between sports participation, motor competence and weight status: A longitudinal study. *J Sci Med Sport* 2016;19:825-829.
(48) Temple M, Robinson JC. A systematic review of interventions to promote physical activity in the preschool setting. *J Spec Pediatr Nurs* 2014;19:274-284.
(49) Piek JP, Barrett NC, Smith LM, Rigoli D, Gasson N. Do motor skills in infancy and early childhood predict anxious and depressive symptomatology at school age? *Hum Mov Sci* 2010;29:777-786.
(50) National Cancer Institute. Evaluating ASSIST A blueprint for understanding state-level tobacco control. Stillman FA, Schmitt CL, editors. Toboacco Control Monograph No. 17. 2007. Bethesda, MD, US Department of Health and Human Services, National Institutes of Health, National Cancer Institute.
(51) Kumar JV, Moss ME. Fluorides in dental public health programs. *Dent Clin North Am* 2008;52:387-401, vii.
(52) Houston DJ, Richardson LE, Jr. Getting Americans to buckle up: the efficacy of state seat belt laws. *Accid Anal Prev* 2005;37:1114-1120.
(53) Taber DR, Chriqui JF, Perna FM, Powell LM, Slater SJ, Chaloupka FJ. Association between state physical education (PE) requirements and PE participation, physical activity, and body mass index change. *Prev Med* 2013;57:629-633.
(54) LaRowe TL, Tomayko EJ, Meinen AM, Hoiting J, Saxler C, Cullen B. Active Early: one-year policy intervention to increase physical activity among early care and education programs in Wisconsin. *BMC Public Health* 2016;16:607.
(55) Erinosho T, Hales D, Vaughn A, Mazzucca S, Ward DS. Impact of Policies on Physical Activity and Screen Time Practices in 50 Child-Care Centers in North Carolina. *J Phys Act Health* 2016;13:59-66.
(56) Kakietek J, Dunn L, O'Dell SA, Jernigan J, Kettel KL. Training and technical assistance for compliance with beverage and physical activity components of New York City's regulations for early child care centers. *Prev Chronic Dis* 2014;11:E177.
(57) Wolfenden L, Finch M, Nathan N et al. Factors associated with early childhood education and care service implementation of healthy eating and physical activity policies and practices in Australia: a cross-sectional study. *Transl Behav Med* 2015;5:327-334.
(58) Benjamin Neelon SE, Finkelstein J, Neelon B, Gillman MW. Evaluation of a Physical Activity Regulation for Child Care in Massachusetts. *Child Obes* 2016.
(59) Childcare Resource and Research Unit. Provincial/territorial requirements in regulated childcare for physical activity, outdoor space and time. 2013.

(60) Kaphingst KM, Story M. Child care as an untapped setting for obesity prevention: state child care licensing regulations related to nutrition, physical activity, and media use for preschool-aged children in the United States. *Prev Chronic Dis* 2009;6:A11.
(61) Stephens RL, Xu Y, Lesesne CA et al. Relationship between child care centers' compliance with physical activity regulations and children's physical activity, New York City, 2010. *Prev Chronic Dis* 2014;11:E179.
(62) Durlak JA, DuPre EP. Implementation matters: a review of research on the influence of implementation on program outcomes and the factors affecting implementation. *Am J Community Psychol* 2008;41:327-350.
(63) Sisson SB, Campbell JE, May KB et al. Assessment of food, nutrition, and physical activity practices in Oklahoma child-care centers. *J Acad Nutr Diet* 2012;112:1230-1240.
(64) McWilliams C, Ball SC, Benjamin SE, Hales D, Vaughn A, Ward DS. Best-practice guidelines for physical activity at child care. *Pediatrics* 2009;124:1650-1659.
(65) Wolfenden L, Neve M, Farrell L, Lecathelinais C, Sutherland R, Bell C et al. How supportive are childcare service policies and practices to child physical activity? Journal of Science and Medicine in Sport 12, e169-e170. 2010.
(66) Lessard L, Lesesne C, Kakietek J et al. Measurement of compliance with New York City's regulations on beverages, physical activity, and screen time in early child care centers. *Prev Chronic Dis* 2014;11:E183.
(67) Simmons R, Shiffman J. Scaling up health service innovations: A framework for action. In: Simmons R, Fajans P, Ghiron L, eds. *Scaling up health service delivery: From pilot innvoations to policies and programmes*. Geneva, Switzerland: World Health Organization; 2007;1-30.
(68) Damschroder LJ, Goodrich DE, Robinson CH, Fletcher CE, Lowery JC. A systematic exploration of differences in contextual factors related to implementing the MOVE! weight management program in VA: a mixed methods study. *BMC Health Serv Res* 2011;11:248.
(69) Fixsen DL, Naoom SF, Blase KA, Friedman RM, Wallace F. *Implementation Research: A Synthesis of the Literature*. University of South Florida, 2005.
(70) Fixsen DL, Blase KA, Horner R, Sugai G. Scaling up evidence-based practices in education. Brief #1. 2009. Chapel Hill, The University of North Carolina.
(71) Bronfenbrenner U. Toward an experimental ecology of human development. *American Psychologist* 1977;32:513-531.
(72) Moore GF, Moore L, Murphy S. Facilitating adherence to physical activity: exercise professionals' experiences of the National Exercise Referral Scheme in Wales: a qualitative study. *BMC Public Health* 2011;11:935.
(73) Milat AJ, Newson R, King L et al. A guide to scaling up population health interventions. *Public Health Res Pract* 2016;26:e2611604.
(74) Watts AW, Saewyc E, Naylor P.J., Mâsse LC. Impact of nutrition and physical activity policies on the school environment: Case study in British Columbia. *Int J Behav Nutr Phys Act* 2014;14:50.
(75) Naylor PJ, Temple VA. Addressing physical activity and obesity in the preschool year (0-5): A literature review and environmental scan of better practices and recommendations for an implementation strategy in BC. Report to the Early Year PA and HE Advisory Committee, editor. 2011. Victoria (British Columbia).
(76) Morse JM. Determining sample size. *Qualitative Health Research* 2000;10:3-5.
(77) Ward DS, Mazzucca S, McWilliams C, Hales D. Use of the Environment and Policy Evaluation and Observation as a Self-Report Instrument (EPAO-SR) to measure nutrition and physical activity

environments in child care settings: validity and reliability evidence. *Int J Behav Nutr Phys Act* 2015;12:124.
(78) Dodds P, Wyse R, Jones J et al. Validity of a measure to assess healthy eating and physical activity policies and practices in Australian childcare services. *BMC Public Health* 2014;14:572.
(79) Mâsse LC, McKay H, Valente M, Brant R, Naylor PJ. Physical activity implementation in schools: a 4-year follow-up. *Am J Prev Med* 2012;43:369-377.
(80) Hoy WK, Tarter CJ, Kottkamp RB. The organizational climate description questionnaire for elementary schools. *Open Schools/Healthy Schools: Measuring Organizational Climate*. Newbury Park: Sage Publications; 1991;25-45.
(81) Hoy WK, Hannum JW. Middle school climate: An empirical assessment of organizational health and student achievement. *Educational Administration Quarterly* 1997;33:290-311.
(82) Hoy WK. Measuring the Health of the School Climate: A Conceptual Framework. *NASSP bulletin* 1992;76:74.
(83) Hoy WK. The Development of the Organizational Climate Index for High Schools: Its Measure and Relationship to Faculty Trust. *High School Journal* 2002;86:38-50.
(84) Alberta Cancer Foundation. An inventory of Canadian chronic disease and population health data. 2007.
(85) Valente TW, Unger JB, Ritt-Olson A, Cen SY, Anderson JC. The interaction of curriculum type and implementation method on 1-year smoking outcomes in a school-based prevention program. *Health Educ Res* 2006;21:315-324.
(86) Robinson K, Elliott SJ, Driedger SM et al. Using linking systems to build capacity and enhance dissemination in heart health promotion: a Canadian multiple-case study. *Health Educ Res* 2005;20:499-513.
(87) Steckler A, Goodman RM, McLeroy KR, Davis S, Koch G. Measuring the diffusion of innovative health promotion programs. *Am J Health Promot* 1992;6:214-224.
(88) Murray DM, Stevens J, Hannan PJ et al. School-level intraclass correlation for physical activity in sixth grade girls. *Med Sci Sports Exerc* 2006;38:926-936.
(89) Evenson KR, Catellier DJ, Gill K, Ondrak KS, McMurray RG. Calibration of two objective measures of physical activity for children. *J Sports Sci* 2008;26:1557-1565.
(90) Trost SG, Way R, Okely AD. Predictive validity of three ActiGraph energy expenditure equations for children. *Med Sci Sports Exerc* 2006;38:380-387.
(91) Trost SG, Loprinzi PD, Moore R, Pfeiffer KA. Comparison of accelerometer cut points for predicting activity intensity in youth. *Med Sci Sports Exerc* 2011;43:1360-1368.
(92) Tandon PS, Saelens BE, Zhou C, Kerr J, Christakis DA. Indoor versus outdoor time in preschoolers at child care. *Am J Prev Med* 2013;44:85-88.
(93) Addy CL, Trilk JL, Dowda M, Byun W, Pate RR. Assessing preschool children's physical activity: how many days of accelerometry measurement. *Pediatr Exerc Sci* 2014;26:103-109.
(94) Trost SG, Fees BS, Haar SJ, Murray AD, Crowe LK. Identification and validity of accelerometer cut-points for toddlers. *Obesity (Silver Spring)* 2012;20:2317-2319.
(95) Pate RR, Almeida MJ, McIver KL, Pfeiffer KA, Dowda M. Validation and calibration of an accelerometer in preschool children. *Obesity (Silver Spring)* 2006;14:2000-2006.
(96) Kerr J, Norman GJ, Godbole S, Raab F, Demchak B, Patrick K. Validating GPS data with the PALMS system to detect different active transportation modes. *Medicine & Science in Sports & Excercise* 2012;44:S25-S29.
(97) Ward D, Hales D, Haverly K et al. An instrument to assess the obesogenic environment of child care centers. *Am J Health Behav* 2008;32:380-386.

(98) Charmaz K. *Constructing grounded theory: A practical guide through qualitative analysis*. London, England: Sage Publications, 2003.

(99) Charmaz K. Shifting the grounds: Constructivist rounded theory methods. In: Morse JM, Stern PN, Corbin J, Bowers B, Charmaz K, Clarke AE, eds. *Developing Grounded Theory: The Second Generation*. Walnut Creek: Left Coast Press; 2009;127-154.

(100) Charmaz K. *Constructing grounded theory*. 2nd edition ed. Los Angeles, CA: Sage Publications Inc., 2014.

(101) Corbin J, Strauss A. *Basics of qualitative research: Techniques and procedures for developing grounded theory*. 3rd edition ed. Thousand Oaks, CA: Sage Publications, 2008.

(102) Knalf AK, Ayres L. Managing large qualitative data sets in family research. *Jounral of Family Nursing* 1996;2:350-364.

(103) Bottorf J, Oliffe JL, Kelly MT, Chambers NA. Approaches to examining gender relations in health research. In: Greaves L, Oliffe JL, eds. *Designing and conducting gender, sex, and health research*. Thousand Oak (CA): Sage; 2011;175-188.

(104) Yoong SL, Williams CM, Finch M et al. Childcare service centers' preferences and intentions to use a web-based program to implement healthy eating and physical activity policies and practices: a cross-sectional study. *J Med Internet Res* 2015;17:e108.

(105) Finch M, Wolfenden L, Morgan PJ, Freund M, Jones J, Wiggers J. A cluster randomized trial of a multi-level intervention, delivered by service staff, to increase physical activity of children attending center-based childcare. *Prev Med* 2014;58:9-16.

(106) Finch M, Yoong SL, Thomson RJ et al. A pragmatic randomised controlled trial of an implementation intervention to increase healthy eating and physical activity-promoting policies, and practices in centre-based childcare services: study protocol. *BMJ Open* 2015;5:e006706.

(107) Naylor PJ, Macdonald HM, Zebedee JA, Reed KE, McKay HA. Lessons learned from Action Schools! BC--an 'active school' model to promote physical activity in elementary schools. *J Sci Med Sport* 2006;9:413-423.

(108) Naylor PJ, Macdonald HM, Warburton DE, Reed KE, McKay HA. An active school model to promote physical activity in elementary schools: Action Schools! BC. *Br J Sports Med* 2008;42:338-343.

(109) Naylor PJ, Temple VA. Enhancing the capacity to facilitate physical activity in home-based child care settings. *Health Promot Pract* 2013;14:30-37.

(110) Ramanathan S, Allison KR, Faulkner G, Dwyer JJ. Challenges in assessing the implementation and effectiveness of physical activity and nutrition policy interventions as natural experiments. *Health Promot Int* 2008;23:290-297.

(111) Lau EY, Saunders RP, Pate RR. Factors Influencing Implementation of a Physical Activity Intervention in Residential Children's Homes. *Prev Sci* 2016.

(112) Lau E.Y., Saunders RP, Beets WM, Cai B, Pate RR. Factors influencing implementation of a preschool-based physical activity intervention. *Health Education Research (Accepted)* 2016.

(113) Naiman DI, Leatherdale ST, Gotay C, Masse LC. School factors associated with the provision of physical education and levels of physical activity among elementary school students in Ontario. *Can J Public Health* 2015;106:e290-e296.

(114) Mâsse LC, Perna F, Agurs-Collins T, Chriqui JF. Change in school nutrition-related laws from 2003 to 2008: evidence from the school nutrition-environment state policy classification system. *Am J Public Health* 2013;103:1597-1603.

(115) Mâsse LC, de Niet JE, Watts A, Naylor PJ, Saewyc E. Associations between school food environment student's consumption and body mass index of Canadian adolescents. *International Journal of Behavioral Nutrition and Physical Activity* 2014;14:50.
(116) Perna FM, Oh A, Chriqui JF et al. The association of state law to physical education time allocation in US public schools. *Am J Public Health* 2012;102:1594-1599.
(117) Hennessy E, Agurs-Collins T, Massse LC, Moser R, Perna F. State-level school competitive food and beverage laws are associated with children's weight status. *Journal of School Health* 2014;84:609-616.
(118) Stone MR, Faulkner GE, Zeglen-Hunt L, Bonne JC. The Daily Physical Activity (DPA) policy in Ontario: is it working? an examination using accelerometry-measured physical activity data. *Can J Public Health* 2012;103:170-174.
(119) Leatherdale ST, Manske S, Faulkner G, Arbour K, Bredin C. A multi-level examination of school programs, policies and resources associated with physical activity among elementary school youth in the PLAY-ON study. *Int J Behav Nutr Phys Act* 2010;7:6.
(120) Tremblay MS, Leblanc AG, Carson V et al. Canadian Sedentary Behaviour Guidelines for the Early Years (aged 0-4 years). *Appl Physiol Nutr Metab* 2012;37:370-391.
(121) Tremblay MS, Leblanc AG, Carson V et al. Canadian Physical Activity Guidelines for the Early Years (aged 0-4 years). *Appl Physiol Nutr Metab* 2012;37:345-369.
(122) Brussoni M, Gibbons R, Gray C et al. What is the Relationship between Risky Outdoor Play and Health in Children? A Systematic Review. *Int J Environ Res Public Health* 2015;12:6423-6454.
(123) Gray C, Gibbons R, Larouche R et al. What Is the Relationship between Outdoor Time and Physical Activity, Sedentary Behaviour, and Physical Fitness in Children? A Systematic Review. *Int J Environ Res Public Health* 2015;12:6455-6474.
(124) Tremblay MS, Gray C, Babcock S et al. Position Statement on Active Outdoor Play. *Int J Environ Res Public Health* 2015;12:6475-6505.
(125) Temple VA. Child's play: Physical activity and the early years. In: Singleton E, Varpalotai A, eds. *Pedagogy in Motion: Establishing a community of inquiry for studies in human movement*. London, Ontario: The Althouse Press; 2012.
(126) Kuzik N, Clark D, Ogden N, Harber V, Carson V. Physical activity and sedentary behaviour of toddlers and preschoolers in child care centres in Alberta, Canada. *Can J Public Health* 2015;106:e178-e183.
(127) Carson V, Tremblay MS, Spence JC, Timmons BW, Janssen I. The Canadian Sedentary Behaviour Guidelines for the Early Years (zero to four years of age) and screen time among children from Kingston, Ontario. *Paediatr Child Health* 2013;18:25-28.
(128) Temple VA, O'Connor JP. Active Care. A Meaningful Movement Program for Children in Care. 2002. Melbourne, VIC.
(129) Temple VA, Preece A. Healthy Opportunities for Preschoolers. A Resource for Early Learning and Care Providers. A Reseource of LEAP BC. 2007. Vancouver, BC. 2010 Legacies Now.
(130) Temple VA, Preece A. Healthy Opportunities for Preschoolers Family Resource. A Resource of Leap BC. 2007. Vancouver, BC. 2010 Legacies Now.
(131) Bell R, Gibbons S, Temple VA. Fundamental Motor Skills: Active Start & FUNdamentals. A Handbook for Generalists and Physical Education Teachers,as well as Others Tasked with Teaching Motor Skill Development. 2008. Ottawa, ON:PHE Canada.
(132) Herrington S, Brussoni M. Beyond Physical Activity: The Importance of Play and Nature-Based Play Spaces for Children's Health and Development. *Curr Obes Rep* 2015;4:477-483.

(133) Herrington S, Brunelle S, Brussoni M. Outdoor play spaces in Canada: As if if chidlren mattered (In Press). In: Waller Tea, ed. *International handbool of outdoor play and learning*. Thousand Oak (CA): Sage; 2016.

(134) Carson V, Hunter S, Kuzik N et al. Systematic review of physical activity and cognitive development in early childhood. *J Sci Med Sport* 2016;19:573-578.
